# pdb2reaction: End-to-End Reaction-Path Elucidation from PDB Structures Using Machine-Learning Interatomic Potentials

**DOI:** 10.1021/acsomega.6c08242

**Published:** 2026-10-07

**Authors:** Takuto Ohmura, Hajime Sato, Tohru Terada

**Affiliations:** Department of Biotechnology, Graduate School of Agricultural and Life Sciences, 13143 The University of Tokyo, 1-1-1 Yayoi, Bunkyo-Ku, Tokyo 113-8657, Japan

## Abstract

Elucidating enzymatic reaction mechanisms requires a sequence of computational tasks comprising active-site extraction, minimum-energy-path search, transition-state (TS) refinement, intrinsic reaction coordinate (IRC) validation, and quasi-rigid-rotor harmonic-oscillator (QRRHO) thermochemistry—stages typically connected by ad hoc scripting and per-system tuning. We present pdb2reaction, an open-source Python command-line toolkit that automates this entire pipeline directly from a user-curated PDB using a single machine-learning interatomic potential (MLIP) backend. A GPU-accelerated pysisyphus fork is bundled to perform the Hessian-based heavy computations—Hessian, IRC, and vibrational analysis—on the same CUDA device as the MLIP for the default backend, minimizing data transfer overhead. This toolkit also implements a bond-change-driven recursive path-search algorithm, which recovered the two-step reaction mechanism of the geranyl pyrophosphate C6-methyltransferase BezA—an S_N_2-like methyl transfer followed by glutamate-mediated deprotonation via a cationic intermediate—locating both transition states and the intermediate itself without a user-supplied intermediate geometry: from a reactant-state active-site cluster and a minimal scan list, and independently from the reactant and product structures alone. On a benchmark of 23 reaction steps across six enzymes, the broadest-coverage backend, UMA-m-1.1, reproduced the literature mechanism for 19 steps, each with a single imaginary frequency at the transition state. For BezA, all five backends recovered the same two-step pathway and the same rate-limiting step. pdb2reaction enables rapid PDB-to-mechanism elucidation on a single GPU, providing a high-throughput complement to higher-cost DFT-based cluster-model calculations and full enzyme–solvent QM/MM calculations.

## Graphical abstract

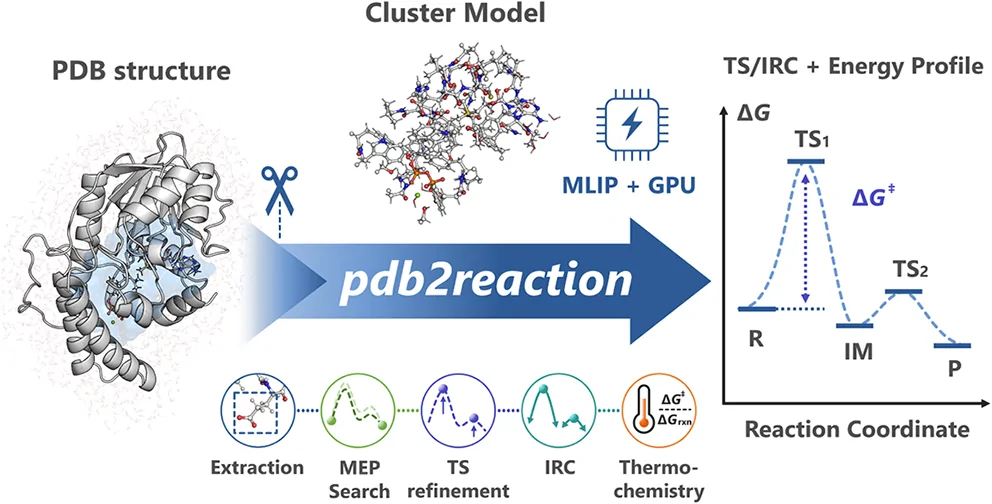

## 1 Introduction

Enzymes catalyze chemical transformations with high rates and selectivity, making mechanistic investigation central to enzyme engineering and inhibitor discovery. Structural information, typically maintained in the Protein Data Bank (PDB) format, provides the essential starting point for linking experimentally observed conformations to hypotheses about bond-making and bond-breaking events at the active site.^1^

Static quantum-chemical cluster models—constructed by extracting an active-site region and applying boundary caps—are a widely used approach for rapid mechanistic hypothesis testing.^2,3^ By truncating the system to the active site, they omit the long-range electrostatics, conformational sampling, and bulk solvation that multiscale quantum mechanics/molecular mechanics (QM/MM) schemes—including ONIOM—treat explicitly; this focus on the active site is what makes them fast enough for the iterative work of mechanistic discovery—proposing a mechanism, testing it, and revising it—and for rationalizing an experimental observation in terms of a specific bond-making and bond-breaking sequence.

The emergence of machine-learning interatomic potentials (MLIPs) has reduced the computational cost of energy and force evaluations. These models are neural networks trained on density functional theory (DFT) reference data—including energies, atomic forces, and stress tensors—to approximate the DFT potential energy surface at a fraction of the original cost. Recent foundation MLIPs,^4–8^ pretrained on diverse atomistic datasets, can now approach DFT-level accuracy for many systems at several orders of magnitude lower cost, bringing such simulations within reach of a single GPU. Recent applications to *small-molecule* reactions include rapid analysis of terpene rearrangement pathways^9^ and the ColabReaction web tool, which simplifies reaction path exploration on Google Colaboratory.^10^ Consequently, the practical bottleneck has shifted toward the preparation of chemically sensible active-site inputs and the integration of extraction, minimum-energy-path (MEP) search, transition-state (TS) refinement, and TS validation into a unified, reproducible pipeline.

Existing tools cover only fragmented parts of this pipeline. RINRUS automates cluster construction but does not perform reaction-path calculations;^11,12^ EnzyHTP targets QM/MM workflows rather than MLIP-based cluster models;^13^ and pysisyphus serves as a general path-search and TS-optimization library that, while accepting PDB inputs, lacks automated active-site extraction, residue-aware cap placement, and multistage workflow orchestration.^14^ Furthermore, while autodE^15^ is designed for small-molecule TS searches from SMILES or 2D-structure input, it lacks the PDB-native, residue-aware cluster-extraction front end—specifically cap placement and covalent-continuity safeguards—needed to transition directly from a curated PDB to a working enzyme model. Consequently, none of these tools deliver a fully automated, PDB-native, MLIP-ready workflow optimized for elucidating enzyme reaction mechanisms.

We developed pdb2reaction as a pure-MLIP cluster-model toolkit that addresses this integration gap. The software provides two key capabilities: (a) a single command-line interface (CLI) for a PDB-to-mechanism workflow that delivers intrinsic reaction coordinate (IRC)-validated TSs and quasi-rigid-rotor harmonic-oscillator (QRRHO) thermochemistry; and (b) a bundled GPU-accelerated pysisyphus fork that maintains the Hessian-based operations—Hessian construction and diagonalization, IRC propagation, and vibrational analysis—on the same CUDA device as the MLIP for the default backend. In cluster-sized systems involving hundreds of atoms, the intensive operations on 3*N* × 3*N* Hessian tensors would otherwise dominate the pipeline wall-clock time due to frequent host–device synchronization and data transfer overhead. Although segmentation of a multistep reaction path into elementary steps via bond-change inspection has precedent in tools such as autodE,^15^ pdb2reaction tightly integrates this logic with its cluster-extraction front end and the GPU-resident Hessian/IRC stack. This allows multistep segmentation to directly trigger TS refinement and IRC validation for each elementary step within a single, automated MLIP-driven pass. While the default MLIP engine is Meta’s UMA-s-1.2 ^4^ on its OMol25-trained^6^ omol task, other models such as UMA-s-1.1, UMA-m-1.1, MACE-OMOL-0 (MACE^5^ trained on OMol25 ^6^), and Orb-v3-omol^6–8^ are also supported.

We apply this toolkit to two complementary enzymatic reactions: a canonical single-step S_N_2 methyl transfer in catechol *O*-methyltransferase (COMT) and a two-step methyl transfer in geranyl pyrophosphate (GPP) C6-methyltransferase, BezA, the latter of which exercises the path-segmentation capability. Furthermore, we quantify backend reliability across a 23-step benchmark involving six different enzymes. Full details regarding installation, CLI and YAML references, implementation (including HPC scaling), application case studies, the benchmark systems, and the path-opt method and cluster-size scaling study are provided in the Supporting Information (Sections S1–S7).

## 2 Methods

### 2.1 End-to-End Workflow

pdb2reaction is a command-driven pipeline that identifies IRC-validated stationary points starting from a protein–ligand PDB file (Figure 1). The pipeline comprises several independent subcommands: extract (active-site cluster construction), opt (input preoptimization), scan (staged distance constraints), path-search or path-opt (MEP construction via the growing string method (GSM)^16,17^ or the Direct-MaxFlux (DMF) method,^18^ tsopt (TS refinement), irc (IRC validation), freq (vibrational analysis and QRRHO thermochemistry), and dft (single-point DFT calculation). These subcommands are sequentially executed within the pipeline, yet each can also be run independently: because every stage reads and writes standard structure files (XYZ/PDB), intermediate results can be inspected, edited, or replaced, and the workflow resumed from any step after manual intervention—for example, supplying a hand-refined TS guess to tsopt or an edited preoptimized cluster to path-search. While default settings are provided, they can be overridden by a YAML configuration file or command-line options, following a standard precedence model. This workflow supports three distinct input modes: (i) scan-list mode, which uses a single curated PDB file supplemented with a scan list of the interatomic distances involved in the reaction; (ii) endpoint mode, which uses reactant, product, and optionally intermediate PDB files; and (iii) TS-only mode. The execution of pdb2reaction for the BezA application is illustrated by the following command-line examples for two input modes:
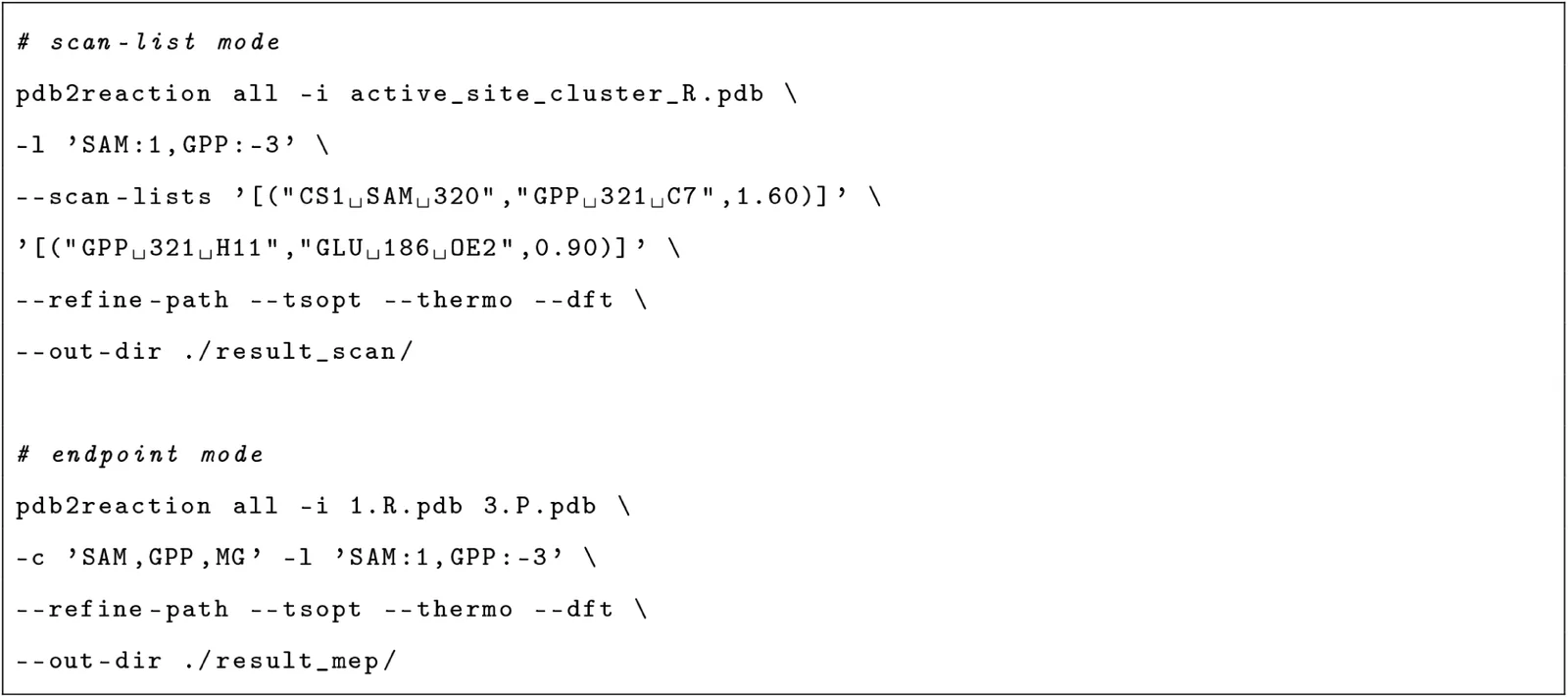


**Figure 1:**
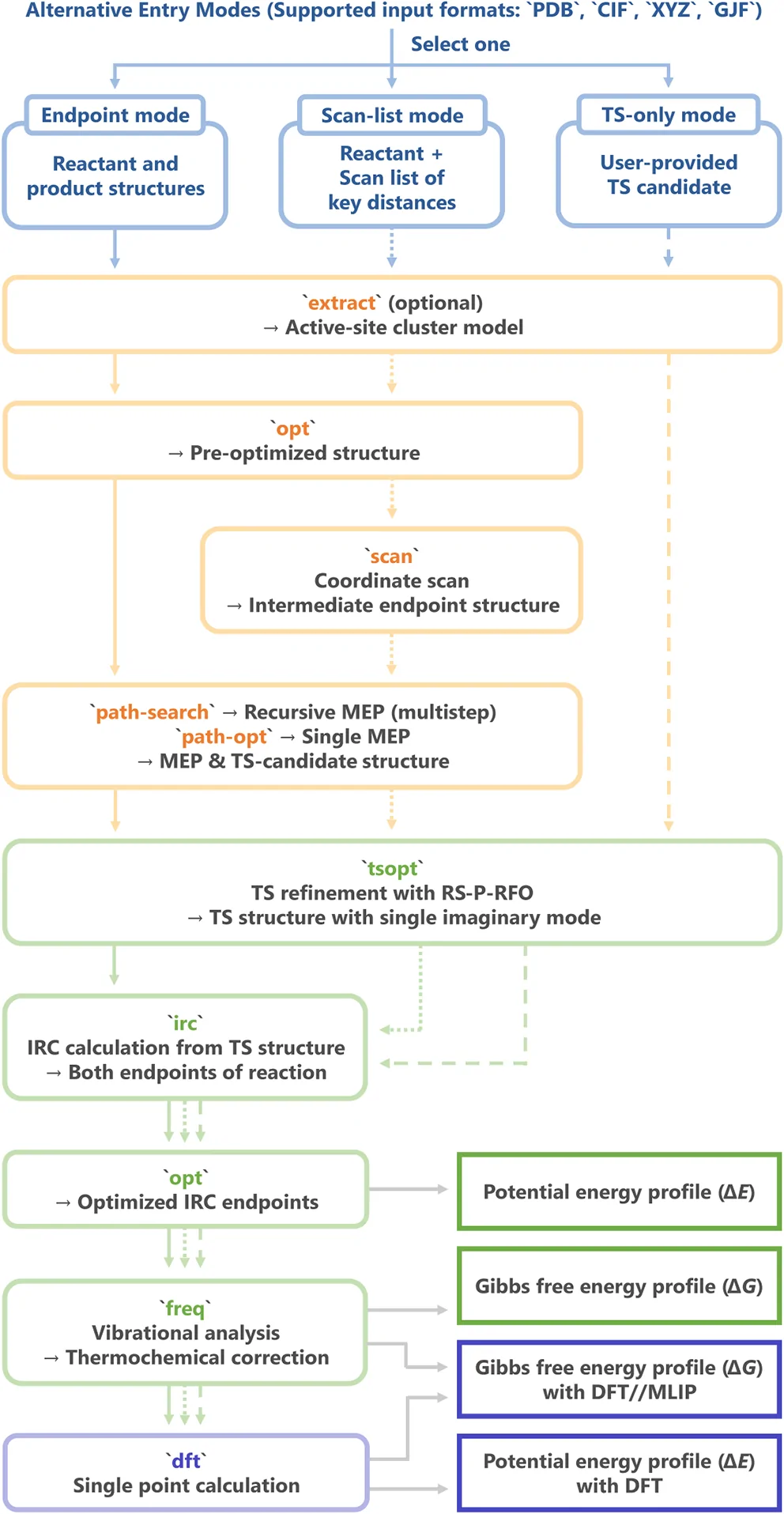
The pdb2reaction workflow. Three mutually exclusive input modes (top) enter a unified pipeline of MLIP-driven stages: reactant and product structures (*endpoint* mode), a single reactant structure plus a scan list of staged key interatomic distances and target values (*scan-list* mode), or a TS candidate (*TS-only* mode). A TS candidate is therefore required only for TS-only mode, not for the endpoint or scan-list modes. Final outputs consist of potential (Δ*E*) and Gibbs (Δ*G*) energy profiles at the MLIP level and, optionally, at the DFT//MLIP level. Here Δ*G* is the QRRHO-corrected relative free energy of the truncated cluster model, not of the full enzyme–solvent system. Stages marked “optional” can be bypassed; the pdb2reaction all command sequentially executes all required subcommands. The freq (partial-Hessian vibrational analysis, PHVA) step verifies the single-imaginary-mode structure shown as the tsopt output; runs that converge to a higher-order saddle or to a minimum are reported as such (Table 2).

Here, the -c option specifies the center residues used to seed active-site extraction, while -l sets per-residue formal charges. The --scan-lists argument encodes the reaction coordinates for each stage of the scan, and --refine-path enables the recursive path search that resolves a multistep segment into elementary steps. The optional --dft flag is included to show the complete workflow in Figure 1; it can be omitted when only MLIP-level energy and thermochemistry profiles are required. Atom selectors refer to the *PDB atom names* of the deposited ligand, which do not coincide with the IUPAC locants used in the Results: the methyl acceptor is GPP atom C7 in the PDB (IUPAC C6), and H11 is the hydrogen it carries. The scan-list example reads the 669-atom cluster produced by the endpoint-mode extraction, so that both input modes run on an identical chemical model; native single-structure extraction from 1.R.pdb traces a different 632-atom boundary of net charge +1, which was not used for any reported barrier (Section S5). Detailed descriptions of CLI options, default parameters, and the cluster-extraction algorithm are provided in Sections S2, S3, and S4, respectively.

An interactive Colab notebook provides structure upload, three-dimensional atom/residue selection, option validation, execution, and linked inspection of MEP, IRC, and stationary-point results. It uses the same CLI workflow and numerical settings.

### 2.2 MLIP Backends and the Bundled GPU pysisyphus Fork

The default backend is UMA-s-1.2 ^4^ accessed through the fairchem interface and run on its omol task, which is trained on OMol25;^6^ UMA-s-1.1, UMA-m-1.1, MACE-OMOL-0,^5,6^ and Orb-v3-omol^6–8^ are also supported and evaluated in the benchmark below. All five benchmarked backends therefore evaluate molecular systems with OMol25-trained models. Because backends are accessed through the modular ASE Calculator interface, any energy/gradient source can be supplied at run time via --calc-file—a Python file exposing a get_calculator­() factory—enabling external engines such as GFN-xTB (via tblite), DFTB+, or ORCA to be coupled without modifying the toolkit. A bundled fork of pysisyphus^14^ implements the Hessian-based operations—Hessian construction and diagonalization, the augmented-Hessian rational function optimization (RFO) and image-RFO (RS-I-RFO) eigensolvers,^19–21^ the Euler predictor–corrector IRC propagator,^22–24^ and mass-weighted PHVA^25^—as CUDA-enabled PyTorch tensor operations, so that they execute on the same device as the MLIP forward/backward pass whenever the Hessian is produced there. The default TS eigensolver, restricted-step partitioned-RFO (RS-P-RFO), diagonalizes the Hessian on the same device and assembles its restricted step from the resulting eigenpairs on the host. Hessians are calculated numerically (default) or analytically. Numerical Hessians are obtained by central finite differences of the atomic forces. In applications requiring higher precision, analytical Hessians can be computed via double backpropagation through the energy computational graph using PyTorch autograd, albeit at the cost of increased GPU VRAM consumption. With the default UMA backend, both routes are GPU-resident, so the Hessian is both constructed and diagonalized on the MLIP device; analytical Hessians are GPU-resident for the other backends as well, whereas their default finite-difference Hessians are currently assembled on the host, and the subsequent linear algebra then follows there (Section S4).

### 2.3 Bond-Change-Driven Recursive Path Search for Multistep Reactions

A single MEP search applied to a multistep reaction often fails to resolve the underlying mechanism, as it may merge consecutive elementary steps into one high-energy feature that obscures individual TSs. pdb2reaction addresses this issue with a recursive path-search algorithm. Bond-breaking and bond-forming events are detected by comparing pairwise distances between the relaxed endpoints of the highest-energy segment against scaled covalent-radius thresholds.^26^ Any reaction segment containing multiple bond-change events is recursively subdivided until each subsegment represents an elementary step—typically involving the formation or cleavage of a single bond—or until a predefined maximum recursion depth is reached (see Section S4 for defaults). Each segment then undergoes independent TS refinement and IRC validation. Finally, all segments are integrated into a continuous reaction trajectory, annotated with per-segment barriers and bond-change histories.

### 2.4 TS, IRC, and Thermochemistry

The default TS refinement algorithm is RS-P-RFO using the full Hessian. IRC validation employs the Euler predictor–corrector method in mass-weighted Cartesian coordinates, using bond-state matching with an RMSD-based fallback for endpoint assignment. Thermochemistry is calculated using the QRRHO approximation,^27,28^ which supplies standard vibrational free-energy corrections to the cluster-model electronic energy; the resulting Δ*G* values are relative energies within the truncated cluster and do not represent the free energy of the full enzyme, whose long-range environmental and conformational contributions lie outside the model’s scope. An optional DFT single-point calculation evaluates stationary-point energies via PySCF^29,30^ with GPU4PySCF^31,32^ acceleration; the default level of theory is ωB97M-V/def2-TZVPD. Additionally, Section S6.13 reports DFT//MLIP single-point results on the 23-step benchmark using the smaller def2-SVP basis set as a throughput-oriented diagnostic.

pdb2reaction automates the geometry optimization and analysis pipeline. Only -i/--input is enforced by the command-line parser; a scientifically valid run requires more. Table 1 summarizes the inputs a user must supply—structurally required or conditionally required depending on the input mode and cluster-extraction step—alongside the stages the pipeline automates once they are set. The COMT and BezA applications below illustrate the level of manual specification actually needed in practice (residue-charge mapping, scan-coordinate selection, and, for BezA, the pre-extracted cluster used to isolate the input-mode comparison). Determining protonation states, verifying active-site-boundary consistency, and assessing conformational-sampling adequacy remain the user’s responsibility and are not enforced by the pipeline (Section S4.26).

**Table 1:** Required and Automated Inputs for a pdb2reaction Run^a^

Category	Input	Flag(s)	Note
Structurally required	Input structure(s)	-i/--input	PDB/XYZ/GJF; parser-enforced
Scientifically required (mode-dependent)	Reaction hypothesis	3 input modes	scan-list/endpoint/TS-only
	Total charge	-q or -l	direct value or per-residue mapping
	Spin multiplicity	-m/--multiplicity	not inferred from the structure; falls back to 1 (singlet)
	Active-site definition	-c/--center, -r/--radius	required if extract runs; radius default 2.6 Å
Automated once the above are set	Cluster → thermochemistry	—	extraction → pre-opt → MEP → TS opt → IRC → PHVA/QRRHO (± DFT)
User judgment (verified, not enforced)	Protonation, boundaries, sampling	—	Section S4.26

a Only the first row is enforced by the CLI parser; the remaining required-input rows are scientifically necessary but are not parser-enforced. See the text above and Section S4.26 for the full description behind each entry.

## 3 Results and Discussion

We evaluated the utility and performance of pdb2reaction through three complementary applications: (i) a canonical single-step S_N_2 reaction of catechol *O*-methyltransferase (COMT); (ii) a multistep reaction in the geranyl pyrophosphate (GPP) C6-methyltransferase BezA, used to demonstrate the recursive path-search algorithm; and (iii) a 23-step benchmark involving six different enzymes to assess MLIP-backend reliability. All calculations were performed on a single CUDA-capable NVIDIA GPU using the UMA-s-1.2 backend (the pipeline default), unless otherwise specified.

### 3.1 COMT: Single-Step S_N_2 Reaction

COMT catalyzes an S_N_2 methyl transfer from *S*-adenosyl-l-methionine (SAM) to a catechol oxygen (Scheme S1A; see Section S5 for cluster protonation state and charge conventions). This application evaluated the pipeline’s ability to produce internally consistent barriers within the cluster model for a well-characterized single-step reaction and compared the two input modes—scan-list and endpoint—on a system where the reactant and product share an identical frozen-atom boundary, both being derived from the QM/MM-optimized geometries reported by Kulik et al.^33^

The extract subcommand automatically generated 670-atom cluster models (pocket radius -r 4.0 Å) for the reactant and product from their respective QM/MM-optimized structures (Figure 2A–C). Both input modes—scan-list and endpoint—were executed to completion. The pipeline successfully recovered the expected C_E_(SAM)–O_2_(catechol) bond formation (2.71 → 1.44 Å) with concomitant S­(SAM)–C_E_ cleavage (1.80 → 3.23 Å; Figure 2G). The scan-list and endpoint modes yielded Δ*G* ^‡^ values of 17.11 and 16.94 kcal mol^–1^, respectively (Figure 3A). Both TS structures converged with a single imaginary mode, and the optimized TS reproduces the QM/MM reference geometry over its reactive core (heavy-atom RMSD 0.75 Å; Section S5). IRC calculations confirmed the methyl-transfer pathway in both cases (see Figures S2 and S3 for the IRC trajectories). A DFT//MLIP single-point rescore (ωB97M-V/def2-SVP) of these UMA-s-1.2 geometries gives Δ*E* ^‡^ = 13.6 (scan-list) and 13.5 (endpoint) kcal mol^–1^ (Table S28), versus 17.06 and 17.08 at the MLIP level (same table) and 15.9 for the QM/MM literature reference. Both pipeline Δ*G* ^‡^ values lie 1.6–1.8 kcal mol^–1^ below the apparent experimental barrier of 18.7 kcal mol^–1^, obtained from the reported *k*_cat_ = 0.40 s−1 ^34^ via the Eyring equation (κ = 1) at the temperature at which it was measured (37 °C); the computed values are QRRHO free energies at 298 K, and the *k*_cat_-derived value is an apparent barrier for the rate-limiting step rather than necessarily the elementary chemical step computed here (Table S28).

**Figure 2:**
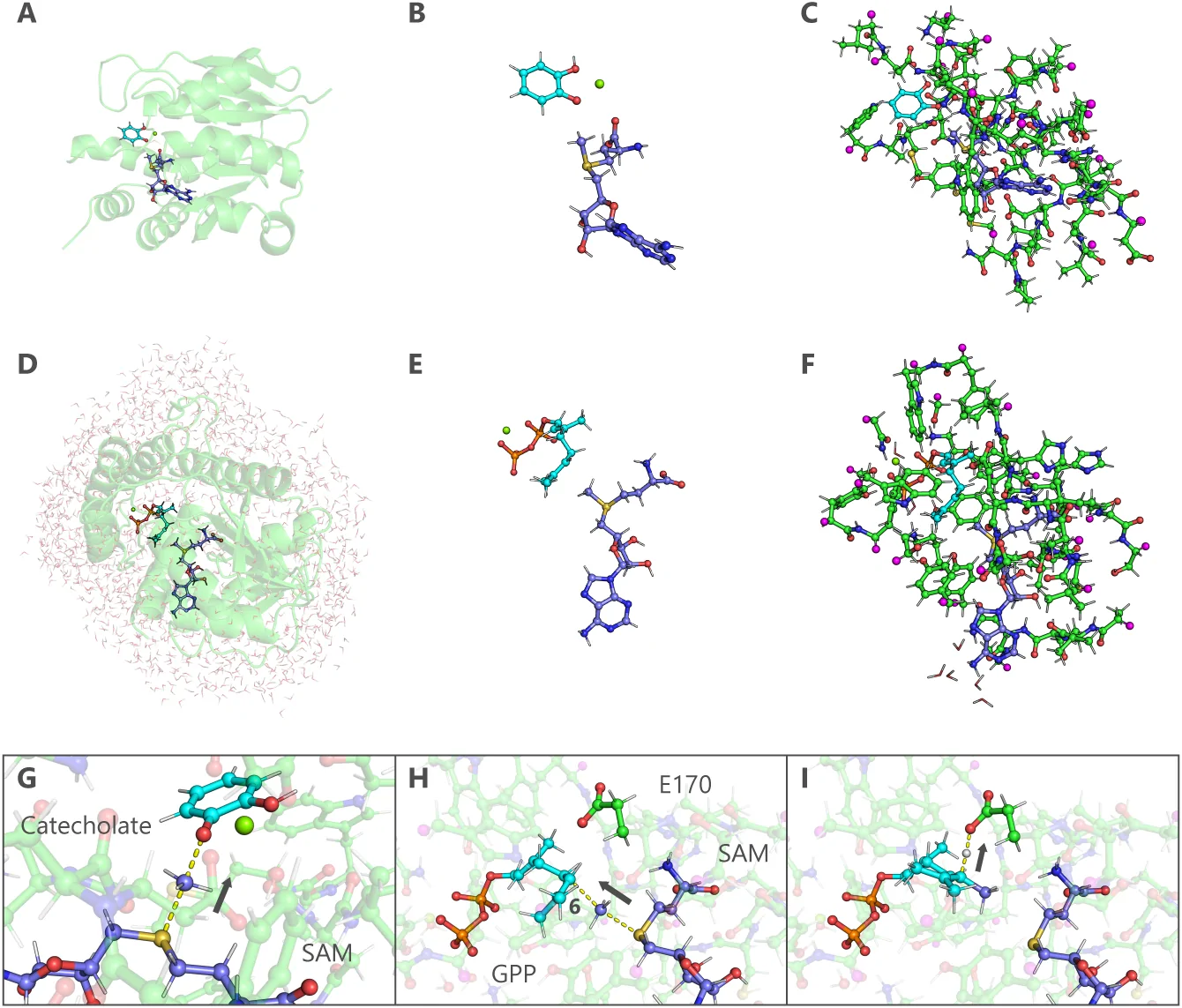
Active-site cluster extraction and TS structures (scan-list-mode models). COMT: (A) whole structure for the reactant highlighting the substrate (catecholate), SAM, and Mg^2+^ ion in a ball-and-stick representation; (B) isolated structures of catecholate, SAM, and Mg^2+^ ion; and (C) the extracted cluster model (cap H atoms in magenta; three crystallographic waters retained from Kulik et al.,^33^ one of which completes the Mg^2+^ coordination shell). BezA: (D) whole structure for the reactant, including the active-site waters contained in the reported structure of Tsutsumi et al.^35^ and used here without modification, highlighting GPP, SAM, and Mg^2+^; (E) isolated GPP, SAM, and Mg^2+^; (F) the extracted cluster model. Per-mode cluster sizes and total charges are tabulated in Table S26. Transition states: (G) COMT TS; (H) BezA TS_1_ (SAM-to-GPP methyl transfer); and (I) BezA TS_2_ (Glu170-mediated C6–H deprotonation).

**Figure 3:**
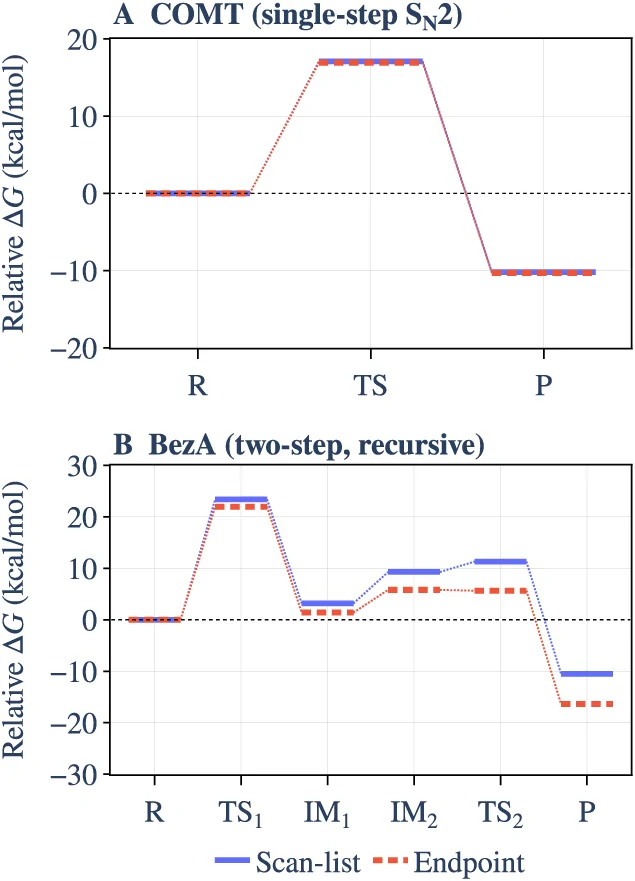
Relative Gibbs free-energy profiles at 298 K. The results from the scan-list (blue, solid) and endpoint (red, dashed) modes are compared for **(A)** COMT and **(B)** BezA. Stationary points are labeled R (reactant), TS (transition state), IM (intermediate), and P (product). In BezA, TS_1_ and TS_2_ correspond to the methyl-transfer and deprotonation transition states, respectively. IM_1_ and IM_2_ represent the same cationic intermediate, obtained by relaxing TS_1_ and TS_2_ via forward and reverse IRC calculations, respectively (they are labeled IM_1_1_ and IM_1_2_ in the Supporting Information).

### 3.2 BezA: Two-Step Reaction

BezA catalyzes a two-step methylation: an S_N_2 methyl transfer from SAM to the C6 position of the GPP C6=C7 alkene, yielding a tertiary C7 carbocation intermediate followed by C6 deprotonation by Glu170 to recover the C6=C7 double bond, forming 6-methylgeranyl pyrophosphate (Scheme S1B).^35^ Residue numbering follows the literature;^35^ e.g., Glu170 corresponds to residue 186 in the original PDB. The reactant and product PDB structures of BezA used here were kindly provided by Prof. Y. Moriwaki (Institute of Science Tokyo). This application evaluated the recursive path-search algorithm, specifically examining (i) its ability to recover the cationic intermediate without user-supplied intermediate geometries, and (ii) the consistency of the results between the two input modes on a multistep reaction where the reactant and product are derived from independent optimizations.

In the scan-list mode, using only a reactant PDB and a minimal two-stage scan list (C–C bond formation and subsequent C6–H deprotonation), we executed pdb2reaction with the --refine-path option. The extract subcommand automatically generated the active-site cluster—the catalytic Glu170 and the surrounding side chains, the active-site waters, and the Mg^2+^, GPP, and SAM substrates/cofactors, with cap hydrogens at the severed bonds (Figure 2D–F, Table S26). Each cap hydrogen replaces a covalent bond severed when the active-site cluster is excised and is placed along the original bond vector, so the number of cap hydrogens equals the number of bonds cut at the cluster boundary. To isolate the scan-list/endpoint comparison in goal (ii) above from cluster-model differences, the quantitative barriers reported below were computed on the 669-atom endpoint-mode cluster (Table S26) for both input modes, rather than on the native 632-atom scan-list cluster shown in Figure 2D–F. The recursive path-search algorithm detected two bond-change events along the scan trajectory, thereby dividing the reaction path into two elementary steps. This process automatically triggered reaction-segment refinements and identified the cationic intermediate (IM) as a relaxed stationary point between the two elementary steps, relying solely on geometry and bond-change analysis. Both transition states carry a single imaginary mode, the reactive one (|*ν̃*| = 476 cm^–1^ for TS_1_ and 724 cm^–1^ for TS_2_; Figure 2H–I), and IRC calculations confirmed that the forward path of step 1 and the reverse path of step 2 both relaxed into the same cationic IM structure (Figure S4). The independent optimization of these two relaxed structures (IM_1_ and IM_2_ in Figure 3B) allows the peripheral cluster (side chains, active-site waters) to settle into slightly different local conformers, which accounts for their nonzero relative energy offset.

The pipeline characterized the mechanism as a rate-limiting SAM-dependent methyl transfer (Δ*E* ^‡^ = 24.9, Δ*G* ^‡^ = 23.4 kcal mol^–1^) followed by a comparatively low-barrier deprotonation (Δ*E* ^‡^ = 5.5, Δ*G* ^‡^ = 2.1 kcal mol^–1^ relative to IM; Figure 3B, solid).

The literature reference (ONIOM QM/MM with B3LYP) reports lower potential-energy barriers (Δ*E* ^‡^ = 12.4 and 0.2 kcal mol^–1^), a gap of 12.5 kcal mol^–1^ on the rate-limiting step, but that comparison is between two different models rather than between two energy methods. A DFT//MLIP single-point rescore of the rate-limiting step on the same MLIP geometry gives Δ*E* ^‡^ = 25.6 kcal mol^–1^ (Table S29), within 0.7 kcal mol^–1^ of the MLIP value, so the deviation does not originate in the MLIP energies. The two models differ in composition: the reference ONIOM model treats Glu170, Phe214, Ser277, Asn281, GPP, the Mg^2+^ ion, one coordinating water, and the part of SAM around the transferring methyl group at the QM level, embedded in the MM environment of the whole ∼9,200-atom enzyme; our cluster model evaluates all 669 atoms with the MLIP, with a frozen cap-hydrogen boundary and no surrounding MM environment (Section S5).

The two models therefore let the active site respond differently along the reaction: between R and TS_1_, no heavy atom of the reference model moves by more than 0.5 Å (RMS displacement 0.05 Å), whereas in the cluster, the Asp168 carboxylate relaxes together with the transferring methyl group (0.83 Å), and the contrast is larger for the second step. The reactive cores of the two transition states nevertheless agree (heavy-atom RMSD 0.43 Å over SAM, GPP, Mg^2+^, and Glu170; Section S5). What this case study establishes is the recovery of the mechanism—both transition states and the cationic intermediate—without a user-supplied intermediate geometry, and the agreement of the transition-state structures with the reference ones over their reactive cores.

The endpoint mode was executed using only the optimized reactant and product PDB structures, with no intermediate geometry supplied. The recursive path-search algorithm successfully detected the multistep character of the reaction by identifying two distinct bond-change events along the initial GSM string, subsequently triggering independent TS optimization and IRC calculations on each elementary step (Figure 3B, dashed; see Figure S5 for the IRC trajectories). The successful recovery of the cationic intermediate and both flanking TSs solely from the reactant and product structures demonstrates that scan-list input is not strictly required for multistep reaction detection. Quantitatively, with both modes on the same UMA-s-1.2 backend, the rate-limiting methyl-transfer barrier (Δ*G* ^‡^ = 21.9 kcal mol^–1^) agreed with the scan-list value (23.4 kcal mol^–1^) to within 1.5 kcal mol^–1^, and the cationic IM free energy to within 1.8 kcal mol^–1^. The residual offset reflects the sensitivity of the energetic results to the consistency of the frozen-atom boundary between the two independently optimized input PDBs. Consequently, the scan-list mode is the safer input when reactant and product come from independent optimizations because it derives both endpoints from one structure and so freezes the boundary atoms at identical coordinates; the endpoint mode is the simpler input when the two structures already share that boundary, as in COMT.

### 3.3 Multi-Enzyme Benchmark

Beyond the two specific applications, we evaluated the pipeline on the cluster-model benchmark set by Kromann et al. (2016),^36^ which spans six enzymes comprising 23 elementary reaction steps (Table S33). The reported B3LYP literature barriers include zero-point energy (ZPE) corrections, whereas the MLIP values are electronic-energy differences on MLIP-optimized geometries; the numerical deviations below are therefore cross-method external comparisons rather than like-for-like estimates of MLIP error. All benchmark calculations were performed on a single NVIDIA GH200 120 GB GPU in double precision with deterministic kernels (--precision fp64 --deterministic), and otherwise at the pipeline’s defaults: a finite-difference Hessian and single-pass path optimization, with the recursive path search off. This is the “default” condition referred to throughout; the other eight conditions of the ablation grid are defined in Section S6.

UMA-m-1.1 yielded a clean transition state for 19 of 23 steps, the broadest coverage, and its passing set contains every step that MACE-OMOL-0 passes (18 of 23); UMA-s-1.1 and UMA-s-1.2 each reached 17 clean passes (with 1 higher-order saddle for UMA-s-1.2), and Orb-v3-omol reached 16 clean passes and 2 higher-order saddles (Table 2; see Table S35 for details). UMA-m-1.1 also provided the best coverage for Zn-containing metalloenzyme steps (6 of 7 clean passes across the 1AH7 and 1RTQ systems; Table S35), whereas Orb-v3-omol exhibited the fastest median wall-clock time (Table S42). With all 23 steps drawn from a single curated set, a spread of this size lies within the resolution of the benchmark, so we report the counts per backend rather than ranking them. Three steps are reproduced by no backend under the default condition: the tungsten-dependent 2E7Z A/step 3 (a proton transfer) and 2E7Z B, together with 4OTA A/step 2—the last of which all five backends do recover under the Direct-MaxFlux driver (Section S6).

**Table 2:** MLIP-Backend Performance on the 23-Step Benchmark under the Default Condition (Double Precision, Deterministic Kernels, Finite-Difference Hessian, Single-Pass Path Optimization)^a^

Backend	Clean/HOSP	Other/E	*N*	MAE	MSD	*r*	Time
MACE-OMOL-0	18/0	5/0	18	5.6	+4.6	0.85	8.1
UMA-s-1.1	17/0	4/2	17	5.3	+4.0	0.87	8.6
UMA-s-1.2	17/1	4/1	18	5.5	+3.8	0.85	9.3
UMA-m-1.1	**19**/0	3/1	19	5.1	+3.5	0.86	21.7
Orb-v3-omol	16/2	5/0	18	4.5	+3.6	0.91	**3.8**

a Clean and HOSP denote counts of steps reproducing the requested bond topology with one and two or more imaginary frequencies, respectively; Other and E denote counts yielding a first-order TS for another reaction and a minimum after TS optimization, respectively. *N* is the Clean+HOSP set used for numerical comparison. MAE and MSD are the mean absolute and mean signed (MLIP–literature) cross-method deviations between the MLIP electronic barriers and the reported ZPE-corrected B3LYP barriers; because *N* differs slightly, they are not directly comparable as a backend ranking. *r* is the Pearson correlation over the same paired set, and Time is the median wall-clock time per step over that set. Energies are in kcal mol^–1^ and times in min. Bold marks the highest clean-pass count and shortest time.

Across the 90 bond-topology-reproducing cases, every backend overestimates the literature barrier on average by +3.5 to +4.6 kcal mol^–1^, and removing each backend’s mean signed deviation lowers its mean absolute deviation from 4.5–5.6 to 2.8–3.7 kcal mol^–1^ (Section S6.11); the bias and the residual scatter are of comparable size, and the bias itself varies with the enzyme (per-enzyme mean deviations from −1.2 (1AH7) to +6.7 (1O9S) kcal mol^–1^). Part of this overestimation is the cross-method convention: the reference barriers are ZPE-corrected, whereas ours are electronic. The barriers nevertheless track the references across the set (Pearson *r* = 0.85–0.91 per backend), and what these numbers establish firmly is the mechanism rather than its absolute energetics—as the BezA case study shows directly: all eight clean backend/input-mode runs there return the same two-step pathway and place the methyl transfer as rate-limiting, while the Δ*G* ^‡^ of that step spans 20.6–31.1 kcal mol^–1^ across those runs (Tables S31 and S32).

On one GH200, the pipeline locates and IRC-validates a complete elementary step in a median of 9.2 min (Table S42)—about twice what the three ωB97M-V/def2-SVP single points take merely to rescore its reactant, transition state, and product on the same cluster (4.8 min), and with no DFT geometry optimization at any stage. For COMT, the eight clean runs give Δ*G* ^‡^ = 16.7–18.8 kcal mol^–1^ (Table S30), against the 18.7 kcal mol^–1^ implied by the reported *k*_cat_ at its measurement temperature (Section S5). Section S6 provides further details, including the per-backend MAE summary and common-subset analysis (Table S40 and Section S6.11), per-condition pass counts (Table S37), per-step deviation tables (Table S41), DFT//MLIP single points (Section S6.13), and per-stage timings (Table S42).

### 3.4 Computational Performance and Cluster-Size Dependence

To test whether a tractable active-site cluster preserves the reaction outcome of the full enzyme, we varied the COMT model from 457 to 3420 atoms and evaluated both MEP drivers over multiple path resolutions (Tables S46 and S47; Section S7). From 670 atoms upward, GSM and DMF recovered the intended S_N_2 bond changes—C–O formation with concomitant S–C cleavage—at every completed node count. At the recommended 50-node resolution, the 670–1117-atom clusters reproduced the basin-referenced barrier of the full 3420-atom system to within 0.2–4.0 kcal mol^–1^ across the two drivers. Thus, for COMT with UMA-s-1.1, active-site clusters of 670–1117 atoms retain the full-system mechanism and reproduce its barrier to within 4 kcal mol^–1^ at the path-optimization level, while avoiding most of the full-enzyme cost. The residual is not monotonic in cluster size (Section S7).

The timing results quantify the associated saving. GSM cost increases with path resolution and system size, becoming impractical for the full system at high resolution, whereas DMF is substantially less sensitive to the number of path images (Table S45). Together with the reaction-fidelity analysis above, these results support --max-nodes 50–100 as a practical production range: 20 nodes can mix an adjacent proton transfer into the path, while higher resolutions add substantial cost without changing the converged cluster-size conclusion.

## 4 Conclusions

We have presented pdb2reaction, an open-source toolkit that provides an integrated pipeline for cluster-model enzyme mechanism analysis—comprising PDB input, active-site extraction, MEP search, TS refinement, IRC validation, and QRRHO thermochemistry—on a single GPU. The bundled GPU-accelerated pysisyphus fork keeps the Hessian-based tensor computations—Hessian, IRC, and vibrational analysis—on the same device as the MLIP for the default backend, minimizing host–device data transfer. Furthermore, the bond-change-driven recursive path-search algorithm automatically divides a multistep reaction into elementary steps, subsequently triggering independent TS optimization and IRC calculations. By leveraging the computational efficiency of recent foundation MLIPs, our toolkit provides a high-throughput complement to more expensive DFT-based cluster-model calculations and full enzyme–solvent QM/MM calculations, all within the capacity of a single GPU.

In the BezA two-step methyltransferase, the recursive path-search recovered the reported mechanism—an S_N_2-like methyl transfer, a cationic intermediate, and Glu170-mediated deprotonation—in scan-list mode from a reactant-state active-site cluster and a two-entry scan list, and independently in endpoint mode from the reactant and product structures, requiring no user-supplied intermediate geometry in either mode. Across the 23-step benchmark (Table 2), UMA-m-1.1 reproduced the literature mechanisms for 19 of 23 steps with a single imaginary frequency at the transition state, and the other four backends reproduced 16–18 steps each. Measured against the ZPE-corrected literature references, the electronic barriers computed here deviate by 4.5–5.6 kcal mol^–1^ under that cross-method convention, but they track the references across the set (*r* = 0.85–0.91 per backend). What these numbers establish firmly is the mechanism rather than its absolute energetics, as the BezA case study shows directly: all eight clean backend/input-mode runs return the same two-step pathway and the same rate-limiting step. The transition states themselves match the QM/MM and ONIOM reference structures over their reactive cores (0.43–0.75 Å heavy-atom RMSD), so they are ready-made starting structures for a higher-level study.

This combination—PDB-native input, GPU-resident orchestration, and an end-to-end pipeline—makes pdb2reaction a practical instrument for mechanistic work: locating and IRC-validating the elementary steps of a proposed mechanism at the pace at which hypotheses are formulated and placing an experimental observation on a specific sequence of bond-making and bond-breaking events.

## Supplementary Material





## Data Availability

The Python implementation of pdb2reaction is openly available under the GPL-3.0 license at https://github.com/t-0hmura/pdb2reaction. The corresponding source code is archived on Zenodo under the concept DOI 10.5281/zenodo.19197865. All results reported here were produced with version 0.4.11 (pip install pdb2reaction==0.4.11), archived under 10.5281/zenodo.21335084. Input data (coordinates) for the benchmark and case studies are provided as a separate Supporting Data archive.

## Notes

Supporting text, tables, and figures: installation instructions, CLI and YAML reference, implementation details, extended worked examples, HPC scaling notes, application case studies, benchmark definitions, per-step benchmark tables, and additional figures (PDF)
Supporting Data: coordinates for the benchmark and case studies (ZIP)
