# Supplementary material for: pdb2reaction: End-to-End Reaction-Path Elucidation from PDB Structures Using Machine-Learning Interatomic Potentials
Source: ACS Omega. 2026 Oct 7;11(42):65454–62. doi: 10.1021/acsomega.6c08242 (PMC13717662; doi:10.1021/acsomega.6c08242)
Supplement: Supplementary file 1 [file ao6c08242_si_001.pdf]

Supporting Information

pdb2reaction: End-to-End Reaction-Path Elucidation from PDB Structures Using Machine-Learning Interatomic Potentials

Takuto Ohmura†, Hajime Sato†, and Tohru Terada†,*

†Department of Biotechnology, Graduate School of Agricultural and Life Sciences, The University of Tokyo, 1-1-1 Yayoi, Bunkyo-Ku, Tokyo 113-8657, Japan

*Email: tterada@bi.a.u-tokyo.ac.jp

This Supporting Information provides reproducibility-oriented details for `pdb2reaction`: installation, the CLI and YAML reference, implementation details, HPC scaling recipes, application case studies, and benchmark validation.

CONTENTS

S1. Installation and environment — quick-start, system requirements, and the Supporting Data archive . . . S-1
S2. CLI manual — per-subcommand options, defaults, and minimal examples . . . S-3
S3. YAML configuration reference — top-level blocks, keys, and precedence . . . S-28
S4. Implementation details — algorithms, defaults, Hessian/IRC handling, and HPC scaling . . . S-32
S5. Application case studies — COMT and BezA SAM-dependent methyltransferases . . . S-54
S6. Benchmark system details — per-enzyme references, pass criteria, and timings . . . S-64
S7. Path-opt method and cluster-size scaling — GSM vs Direct-MaxFlux timing, fidelity, and barrier convergence . . . S-89

■ S1. INSTALLATION AND ENVIRONMENT

S1.1 Quick-start (Linux + CUDA)

```
# 1. Install PyTorch with CUDA support first (match the index URL to the
#    local CUDA toolkit).
pip install torch --index-url https://download.pytorch.org/whl/cu128
pip install pdb2reaction
hf auth login           # needed once for the gated UMA checkpoint
pdb2reaction all --help  # verify the install
```

S1.2 System requirements

- **Operating system.** Linux.
- **Python** $\geq$3.11.
- **GPU (recommended).** NVIDIA GPU with CUDA 12.x.

S1.3 Core and optional dependencies

- **Core runtime:** `numpy`, `scipy`, `ase` (structure I/O), `click` (CLI framework), and `pyyaml` for configuration. `pdb2reaction` requires ASE $\geq$3.26.0, NumPy $\geq$2.0 ($<$2.5), and PyTorch $\sim$2.8.0 (i.e. $\geq$2.8.0, $<$2.9.0); `scipy` and the remaining utility packages are unpinned. Manuscript benchmarks used PyTorch 2.8.0.
- **UMA ML potential:** accessed through Meta's `fairchem` interface.
- **Orb ML potential (optional):** the Orb-v3 backend is provided by `orb-models`.
- **MACE ML potential (optional):** the MACE-OMOL-0 backend is provided by `mace-torch`.
- **Direct-MaxFlux (DMF; Ipopt solver required):** DMF runs through `pydmf`, which optimizes a B-spline path using nonlinear constrained optimization via the Interior Point Optimizer (Ipopt)/`cyipopt` (install `cyipopt` separately, e.g., `conda install -c conda-forge cyipopt`).
- **DFT library (optional):** `PySCF` is used for post hoc DFT single-point evaluation; `gpu4pyscf` (when installed) enables GPU acceleration.

S1.4 Installation

pdb2reaction is distributed via PyPI (install PyTorch with CUDA support first, as in the Quick-start above); installation from source is also supported for development or version pinning:

```
git clone https://github.com/t-0hmura/pdb2reaction.git
cd pdb2reaction
pip install -e .
```

The UMA checkpoint (default uma-s-1p2) is hosted on the gated HuggingFace repository facebook/UMA, so first-time users must accept the model license and authenticate with hf auth login (token from https://huggingface.co/settings/tokens) before fairchem-core can download the weights.

Optional extras. Alternative machine-learning interatomic potential (MLIP) backends and DFT support are installed via pip extras:

```
# ORB backend
pip install "pdb2reaction[orb]"

# DFT library (PySCF + GPU4PySCF)
pip install "pdb2reaction[dft]"

# DMF solver stack (platform dependent, via conda)
conda install -c conda-forge cyipopt
```

The canonical repository is https://github.com/t-0hmura/pdb2reaction and is archived on Zenodo under the concept DOI 10.5281/zenodo.19197865. The accompanying input data are provided as the Supporting Data archive. pdb2reaction is released under the GNU General Public License, version 3 (GPL-3.0).

An interactive Colab notebook provides structure selection, option validation, execution, and result inspection (Figure S17).

CPU-only continuous-integration tests cover the core modules (bond-change detection, extraction logic, CLI utilities).

MACE backend. The MACE-OMOL-0 foundation model is available through mace-torch. mace-torch and fairchem-core can pin incompatible versions of e3nn, so the recommended setup is a dedicated environment that does not include fairchem-core:

```
# Recommended: dedicated env without fairchem-core
conda create -n <mace-env> python=3.11
conda activate <mace-env>
pip install pdb2reaction
pip uninstall fairchem-core
pip install mace-torch
```

Alternatively, uninstall fairchem-core from an existing pdb2reaction environment before installing mace-torch.

Orb backend. The Orb-v3 backend (default checkpoint orb_v3_conservative_omol) is installed via the [orb] extra (pip install "pdb2reaction[orb]"). Orb-v3 can share an environment with UMA; its e3nn pinning is compatible with fairchem-core. The conservative OMol checkpoint provides analytically differentiable forces/Hessians and is selected automatically when the Orb backend is requested.

S1.5 MLIP backend configuration

Table S1: Default MLIP backend configurations in pdb2reaction.

Backend	Default model	Source
UMA	uma-s-1p2 (task omol)	facebook/UMA on HuggingFace via fairchem-core
MACE	MACE-OMOL-0 (extra_large)	mace-torch
Orb	orb_v3_conservative_omol	orb-models

For every backend the Hessian can be computed by finite difference (default; peak VRAM bounded by a single energy/force call) or analytically (exact second derivative; recommended for smaller clusters where VRAM permits). UMA additionally accepts explicit charge/spin multiplicity through the ASE Atoms.info dictionary.

S1.6 Data archive and verification recipe

The Supporting Data archive accompanying this document contains:

- input structures and archived stationary-point coordinates for the reported runs;

- a `run.sh` script for each archived entry, reproducing the pipeline stages of that run at the version pinned below;
- a csv-format manifest (`manifest.csv`) indexing every archived benchmark run, together with the companion tables `replicates.csv` (per-replicate values for the non-deterministic conditions), `case_study_manifest.csv` (COMT and BezA stationary-point energetics) and `timing_mep_comparison.csv` (the per-run GSM-versus-Direct-MaxFlux wall-clock comparison over the benchmark set, i.e. conditions `c04` and `c06`; this is distinct from the cluster-size/node-count matrix of Section S7) and `ts_optimizer_comparison.csv` (the per-cell imaginary-mode counts, dominant imaginary frequencies and Sella convergence/IRC flags behind Tables S43 and S44);
- the standalone `sella-ts-phva` reference implementation used as the independent TS-optimizer control (`sella_ref/`);
- the complete set of QM/MM reference stationary points for the two case studies (Section S5.1): `COMT/{1.R,TS_ref,3.P}.pdb` and `bezA/{1.R,TS1_ref,2.IM,TS2_ref,3.P}.pdb`. The reactant, intermediate and product files are the pipeline inputs; the `*_ref` transition states are reference geometries for comparison, not pipeline inputs.

Software: `pdb2reaction` v0.4.11 (`pip install pdb2reaction==0.4.11`). Hardware: a CUDA-capable NVIDIA GPU (see **System requirements**). To re-run a single benchmark or case-study entry, change into its directory and execute `bash run.sh`. The nine archived condition labels of the main benchmark (`c01`–`c09`, tabulated with their precision, determinism and MEP settings in the archive's `README.md`; `c04` is the main-text default) and their tabulated results can be reconstructed from `manifest.csv`. The TS-optimizer robustness comparison (Tables S43 and S44) was run outside that condition grid: the archive ships its per-cell outcomes as `ts_optimizer_comparison.csv`, together with the `sella_ref/` implementation; the comparison can be reproduced from that implementation with the `--opt-mode` flags of Section S2. The individual run directories are not included.

■ S2. CLI MANUAL

This section provides a compact manual for the `pdb2reaction` command-line interface (CLI). Several abbreviations are used throughout: minimum-energy-path (MEP), transition state (TS), intrinsic reaction coordinate (IRC), growing string method (GSM), Direct-MaxFlux (DMF), partial Hessian vibrational analysis (PHVA), and density-functional theory (DFT). Structure workflows accept PDB, mmCIF, XYZ, and Gaussian input where chemically applicable; mmCIF and oversized PDB files use the internal structure bridge described in the online documentation. Configuration follows the standard precedence model: built-in defaults are overridden by a YAML file passed via `--config`, which is in turn overridden by CLI options (Section S3).

S2.1 Command overview

Table S1.1: Overview of `pdb2reaction` commands.

Command	Purpose
`all`	End-to-end orchestration command (extract → scan/MEP → TSOPT/IRC → freq/thermo → optional DFT).
`add-elem-info`	Repair element symbols in PDB ATOM/HETATM records.
`extract`	Extract and cap an active-site pocket (cluster model) from PDB/mmCIF structure(s).
`scan`	One-dimensional staged distance scan on a pocket model using the MLIP backend + geometry relaxation.
`scan2d`	Two-dimensional distance scan (grid) to map a local potential energy surface region using the MLIP backend.
`scan3d`	Three-dimensional distance scan (grid) to map a local potential energy surface region using the MLIP backend.
`opt`	Geometry optimization of a single structure on the MLIP potential energy surface.
`sp`	Single-point MLIP energy and forces (optionally an analytic/finite-difference Hessian) on one structure.
`path-opt`	MEP construction between two endpoints using GSM or DMF; export HEI as TS seed.
`path-search`	Recursive multistep MEP search across ≥ 2 ordered structures; segment stitch/bridge.
`tsopt`	TS optimization (`grad` = Hessian-initialized dimer or `hess` = RS-P-RFO) with optional flattening.
`irc`	EulerPC IRC integration from a TS, forward and backward, in mass-weighted Cartesian coordinates.
`freq`	Vibrational analysis (Hessian) with optional PHVA and thermochemistry.
`dft`	Optional single-point DFT (PySCF + GPU4PySCF) on selected structures.
`bond-summary`	Report covalent bond changes between two structures.
`trj2fig`	Post-processing: convert trajectories/energies into figures (energy diagrams, etc.).
`fix-altloc`	Remove alternate-location records from PDB files, selecting one coherent conformer per residue by mean occupancy.
`energy-diagram`	Plot a state-energy diagram from a numeric energy list (no structure required).

S2.2 Common options

The options below are shared across multiple subcommands; each row's *Applies to* entry identifies which subcommands register the option. The per-command tables that follow repeat a shared option only where its default or behavior differs for that command. Several entries (`--workers`, `--show-config`, `--dry-run`) are visible only with `--help-advanced`. Per-command option tables in the rest of this section list only command-specific options. Of the options below, the helper commands `add-elem-info`, `fix-altloc`, `bond-summary`, and `energy-diagram` accept only `-v/--verbose`.

Table S1.2: Common command-line options shared across multiple subcommands.

Option	Description (*Applies to: …*)	Default
`-v, --verbose LEVEL`	Console verbosity, integer `0–3`: 0 silent, 1 milestones only (stage banners, one status line per stage, the final summary, and any error), 2 adds the per-stage detail lines, 3 adds the raw optimizer output. *Applies to:* all subcommands.	`2`
`-l, --ligand-charge TEXT`	Total charge (bare number) or per-resname mapping (e.g. `GPP:-3,SAM:1`) used when `-q` is omitted; the per-resname mapping is applied whether or not active-site extraction runs (with extraction skipped, to the full input PDB/mmCIF structure). *Applies to:* `scan`/`scan2d`/`scan3d`, `extract`, `dft`, `opt`, `sp`, `tsopt`, `irc`, `freq`, `path-opt`, `path-search`, `all`.	`None`
`--workers`, `--workers-per-node`	UMA predictor parallelism. For UMA, `workers > 1` with an explicit analytical-Hessian request is rejected rather than silently downgraded; use `workers=1` or finite differences. Other backends do not use UMA worker parallelism and warn when non-default worker values are supplied. *Applies to:* `scan`/`scan2d`/`scan3d`, `opt`, `sp`, `tsopt`, `irc`, `freq`, `path-opt`, `path-search`, `all`.	`1, 1`

Option	Description (*Applies to: …*)	Default
-b, --backend CHOICE	MLIP backend (uma, orb, mace, aimnet2). *Applies to:* scan/scan2d/scan3d, trj2fig, opt, sp, tsopt, irc, freq, path-opt, path-search, all.	uma
--precision CHOICE	MLIP numerical precision (fp32 or fp64). fp64 runs full-precision inference and a fp64 Hessian. Keep it for any Hessian-based stage (tsopt, freq, irc): at fp32 the finite-difference Hessian carries enough force noise to invent imaginary modes and so to corrupt barriers, which is why Orb and MACE default to fp64. fp32 is the faster choice for scans and path searches. AIMNet2 runs at fp32 only and rejects fp64. The setting is mapped per backend (UMA fp32/fp64; Orb float32-high/float64; MACE default_dtype). *Applies to:* scan/scan2d/scan3d, opt, sp, tsopt, irc, freq, path-opt, path-search, all (i.e. same as -b/--backend except trj2fig).	per backend (uma fp32; orb, mace fp64)
--solvent TEXT	Implicit solvent name for xTB correction (e.g. water); none disables. *Applies to:* same as -b/--backend.	none
--solvent-model CHOICE	xTB solvent model: alpb (analytical linearized Poisson–Boltzmann) or cpcmx. *Applies to:* same as -b/--backend.	alpb
--backend-model TEXT	Model variant for the selected -b/--backend (e.g. uma-s-1p2/uma-m-1p1, orb_v3_conservative_omol, MACE-OMOL-0); unset keeps the backend's built-in model. *Applies to:* same as --precision.	None
--calc-file PATH	Python file exposing get_calculator(...) returning an ASE Calculator, used as the energy/gradient backend (overrides -b/--backend; couples GFN-xTB / DFTB+ / any ASE engine; contract and example in Section S4.3). *Applies to:* same as --precision.	None
--calc-file-func-name TEXT	Name of the callable in --calc-file that returns (or is) an ASE Calculator. *Applies to:* same as --precision.	get_ calculator
--coord-type {cart|redund|dlc|tric}	Optimization coordinate system: cart (Cartesian; the default used for the published results), dlc (delocalized internals; faster for torsion-rich optimizations), or redund/tric (redundant / translation-rotation-internal coordinates). redund/tric are accepted only by the single-structure optimizers (scan/scan2d/scan3d, opt, tsopt, freq); the chain-of-states engines path-opt/path-search/all accept only cart/dlc. *Applies to:* scan/scan2d/scan3d, opt, tsopt, freq, path-opt, path-search, all.	cart
--deterministic / --no-deterministic	Request strict same-stack PyTorch determinism (deterministic algorithms + index_reduce_ shim); slower, and raises for detected unsupported operations. Identity across hardware/package versions and arbitrary custom calculators is outside this guarantee. *Applies to:* same as --precision.	no-deterministic
--allow-charge-mult-mismatch	Skip the cluster charge/multiplicity electron-parity check (logs that it was skipped). Ordinary open-shell systems require the matching multiplicity; reserve this option for an intentionally nonstandard electron count. *Applies to:* same as --precision, plus dft.	False
--print-every INT	Print optimizer status every N cycles. *Applies to:* scan/scan2d/scan3d, opt, tsopt, all.	100
--show-config / --no-show-config	Print the resolved configuration and continue execution. *Applies to:* opt, sp, tsopt, irc, freq, path-opt, path-search, dft, all.	no-show-config
--dry-run / --no-dry-run	Validate options and print the execution plan. In all, extraction is additionally run in a temporary directory when --center is given, to validate the derived charge and electron parity; no computational stage runs and no persistent output is written. *Applies to:* scan/scan2d/scan3d, opt, sp, tsopt, irc, freq, path-opt, path-search, dft, all.	no-dry-run

S2.3 `all`

`all` is the orchestration command that runs through **every pipeline stage**: pocket extraction → optional staged scan → MEP search (single-pass `path-opt` by default, or recursive `path-search` with `--refine-path`) → optional TS optimization + IRC (`tsopt`) → optional vibrational analysis (`freq`) → optional single-point DFT (`dft`). It supports three input modes: scan-list mode (single curated PDB/mmCIF structure with `--scan-lists`), endpoint mode (reactant and product structures, optionally with intermediates), and TS-only mode (`--tsopt` on a single TS guess).

Usage

```
pdb2reaction all -i INPUT1 [INPUT2 ...] [-c SUBSTRATE] [options]
```

CLI options

Table S2: Command-line options for `all`, organized by pipeline stage.

Option	Description	Default
Required I/O		
`-i, --input PATH...`	Two or more full structures in reaction order (single input allowed only with `--scan-lists` or `--tsopt`).	Required
`-c, --center TEXT`	Substrate specification (PDB/mmCIF path, residue IDs like `123,124` / `A:123,B:456`, or residue names like `GPP,MMT`).	Required for extraction
`-o, --out-dir PATH`	Top-level output directory.	`./result_all/`
Active-site extraction (`extract`)		
`-r, --radius FLOAT`	Pocket inclusion cutoff (Å).	`2.6`
`--radius-het2het FLOAT`	Independent hetero-hetero (non-C/H) cutoff (Å). A literal `0.0` is treated as a disabled sentinel and is replaced internally by `0.001` Å so that only exact coincidences are ever included; set a positive radius (e.g., a few Å) to enable hetero-hetero inclusion.	`0.0`
`--include-h2o / --no-include-h2o`	Include waters; `--no-include-h2o` drops HOH/WAT/H2O/DOD/TIP/TIP3/SOL.	`True`
`--exclude-backbone / --no-exclude-backbone`	Remove backbone atoms on non-substrate amino acids.	`False`
`--add-linkh / --no-add-linkh`	Add cap hydrogens for severed bonds (carbon-only).	`True`
`--selected-resn TEXT`	Residues to force include (comma/space separated; chain/insertion codes allowed).	`""`
`--modified-residue TEXT`	Comma-separated modified residue names treated as amino acids for backbone truncation and charge assignment; use `NAME:charge` when the residue has no single built-in nominal charge (e.g. `'HD1:0,SEP:-2'`).	`""`
`-v, --verbose LEVEL`	Console verbosity level, integer `0–3` (see Common options).	`2`
`--freeze-links / --no-freeze-links`	Freeze cap-hydrogen parents in pocket PDBs (reused by scan/tsopt/freq).	`True`
`--freeze-atoms TEXT`	Comma-separated 1-based atom indices to freeze in every stage; indices are mapped onto the extracted model when extraction runs.	`None`
Charge, multiplicity, MLIP backend		
`-l, --ligand-charge TEXT`	Total charge or residue-specific mapping for unknown residues (recommended). When `-q` is omitted, the mapping drives extract-style charge derivation whether or not `-c/--center` extraction runs—with extraction skipped it is applied to the full input complex.	`None`
`-q, --charge INT`	Force the total system charge, overriding extractor rounding / `.gjf` metadata / `--ligand-charge` (logs a warning).	None
`-m, --multiplicity INT`	Spin multiplicity forwarded to all downstream steps.	`1`

Continued on next page

Option	Description	Default
`--workers`, `--workers-per-node`	UMA predictor parallelism (workers > 1 is incompatible with an explicit analytical-Hessian request, which is rejected; other backends warn and ignore non-default worker values).	`1`, `1`
`-b, --backend CHOICE`	MLIP backend (`uma`, `orb`, `mace`, `aimnet2`).	`uma`
`--solvent TEXT`	Implicit solvent name for xTB correction (e.g. `water`). `none` to disable.	`none`
`--solvent-model CHOICE`	xTB solvent model: `alpb` (analytical linearized Poisson–Boltzmann) or `cpcmx`.	`alpb`
`--hessian-calc-mode [Analytical|FiniteDifference]`	Shared MLIP Hessian engine forwarded to tsopt and freq.	`FiniteDifference`
Path search / MEP (`path-search`, `path-opt`)		
`--max-nodes INT`	MEP internal nodes per segment (GSM string images or DMF images).	`20`
`--gsm-param {equi|energy}`	GSM node parameterization; `energy` concentrates nodes in high-energy regions.	`equi`
`--max-cycles-gsm INT`	Maximum GSM string-optimizer cycles.	`300`
`--max-cycles-dmf INT`	Maximum DMF IPOPT iterations.	`300`
`--climb / --no-climb`	Enable the climbing image for standard GSM segments; bridge segments always disable climbing.	`True`
`--refine-path / --no-refine-path`	Single-pass `path-opt` by default; pass `--refine-path` to run recursive `path-search`.	`False`
`--mep-mode {gsm|dmf}`	MEP generator selector forwarded to `path-opt`/`path-search`: GSM or DMF.	`gsm`
`--dmf-backend {cpu|gpu}`	DMF compute backend (`--mep-mode dmf` only): `gpu` (`dmf.torch`/CUDA) or `cpu` (`dmf`/NumPy); retry `cpu` on a GPU out-of-memory error.	`gpu`
`--write-ref-merge BOOL`	Write aligned `mep_w_ref`/`hei_w_ref` inspection composites in recursive path-search runs.	`False`
Optimization control		
`--opt-mode [grad|hess]`	Optimizer preset shared across `scan`, `tsopt`, and `path-search` (`grad` = L-BFGS for minima / Hessian-initialized dimer for TS; `hess` = RFO for minima / RS-P-RFO for TS).	`grad`
`--opt-mode-post [grad|hess]`	Optimizer mode for the TS optimization and the post-IRC endpoint optimizations. Left unset it follows `--opt-mode` if you passed that flag explicitly, and otherwise uses `hess`—which is *not* `--opt-mode`'s own default of `grad`, so omitting `--opt-mode` and passing `--opt-mode grad` select different TS optimizers.	`hess` (or your explicit `--opt-mode`)
`--thresh TEXT`	Convergence preset for scan relaxations and path endpoint/single-structure optimizations (`gau_loose`, `gau`, `gau_tight`, `gau_vtight`, `baker`, `never`).	None (falls back to `gau`)
`--thresh-post TEXT`	Convergence preset for TSOPT and post-IRC endpoint optimizations.	`baker`
`--thresh-gsm TEXT`	Convergence preset for the GSM string optimizer.	`gau_loose`
`--thresh-dmf TEXT`	DMF IPOPT dual-infeasibility tolerance: `tight`, `middle`, `loose`, or a positive float.	`tight`
`--preopt / --no-preopt`	Pre-optimize pocket endpoints before MEP search (also the default for scan preopt).	`True`
Stage toggles		
`--tsopt / --no-tsopt`	Run TS optimization + IRC per reactive segment, or enable TSOPT-only mode (single input).	`False`
`--thermo / --no-thermo`	Run vibrational analysis (`freq`) on R/TS/P and build MLIP Gibbs diagram.	`False`
`--dft / --no-dft`	Run single-point DFT on R/TS/P and build DFT energy + optional DFT//MLIP diagrams.	`False`

Continued on next page

Option	Description	Default
`--dft-engine [gpu|cpu]`	Preferred backend for the DFT stage.	`gpu`
Scan stage overrides (single-input runs only)		
`-s, --scan-lists TEXT...`	One or more Python-like lists describing staged scans on the extracted pocket. Each element is `(i,j,target_Å)`; a single literal runs one stage, multiple literals run sequential stages. `i`/`j` can be integer indices or PDB atom selectors naming the residue name, residue number, and atom name, separated by whitespace, a comma, or a slash, in any order (`'TYR,285,CA'` and `'GPP 321 C7'` are equivalent), and are remapped internally.	None
`--scan-one-based / --no-scan-one-based`	`--scan-one-based` forces 1-based indexing and `--no-scan-one-based` forces 0-based; leaving both unset keeps the scan default (1-based).	None
`--scan-max-step-size FLOAT`	Override scan `--max-step-size` (Å).	None
`--scan-bias-k FLOAT`	Override the harmonic bias strength `k` (eV Å^{-2}).	None
`--scan-relax-max-cycles INT`	Override scan relaxation max cycles per step.	None
`--scan-preopt / --no-scan-preopt`	Override the scan pre-optimization toggle (otherwise `--preopt` propagates).	None
`--scan-endopt / --no-scan-endopt`	Override the scan end-of-stage optimization toggle.	None
`--scan-out-dir PATH`	Custom scan subdirectory (resolved against <out-dir> when relative).	None
TSOPT overrides		
`--tsopt-max-cycles INT`	Override `tsopt --max-cycles` for each refinement.	`100000`
`--tsopt-out-dir PATH`	Custom tsopt subdirectory (resolved against <out-dir> when relative).	None
`--flatten / --no-flatten`	Enable the extra-imaginary-mode flattening loop in tsopt (grad: dimer loop, hess: post-RS-P-RFO).	`False`
`--tsopt-from-mep-tan / --no-tsopt-from-mep-tan`	Guide Hessian-based TS root selection with the HEI tangent.	`True`
`--reject-uphill / --no-reject-uphill`	Opt in to rejecting uphill RFO trials during post-IRC minimum optimization; TS optimization always leaves it disabled.	`False`
`--stop-plateau / --no-stop-plateau`	Opt-in energy-plateau stop for minimum/TS optimization; a stop is reported as `stalled`, not converged.	`False`
`--stop-plateau-thresh FLOAT, --stop-plateau-window INT`	Energy range and window used when `--stop-plateau` is enabled.	`1e-4,50`
`--irc-step-size FLOAT, --irc-max-cycles INT`	Post-TS IRC step length (Bohr) and per-branch cycle cap.	`0.10,125`
`--irc-never-stop / --no-irc-never-stop`	Bypass physical IRC endpoint criteria and trace to the cycle cap.	`False`
Freq overrides		
`--freq-out-dir PATH`	Base directory override for freq outputs.	None
`--freq-max-write INT`	Override `freq --max-write`.	None
`--freq-amplitude-ang FLOAT`	Override `freq --amplitude-ang` (Å).	None
`--freq-n-frames INT`	Override `freq --n-frames`.	None
`--freq-sort [value|abs]`	Override freq mode sorting behavior.	None
`--freq-temperature FLOAT`	Override freq thermochemistry temperature (K).	None (298.15 K)
`--freq-pressure FLOAT`	Override freq thermochemistry pressure (atm).	None (1.0 atm)
DFT overrides		
`--dft-out-dir PATH`	Base directory override for DFT outputs.	None
`--dft-func-basis TEXT`	Override `dft --func-basis`.	None

Continued on next page

Option	Description	Default
`--dft-max-cycle INT`	Override `dft --max-cycle`.	None
`--dft-conv-tol FLOAT`	Override `dft --conv-tol`.	None
`--dft-grid-level INT`	Override `dft --grid-level`.	None
Meta / I/O glue		
`--config FILE`	Base YAML applied before the explicit CLI options and forwarded to `path-search`/`path-opt`, `scan`, `tsopt`, `irc`, `freq`, and `dft`. `all` forwards a derived copy that carries the resolved `geom.freeze_atoms`.	None
`--ref-pdb PATH`	Reference PDB/mmCIF topology when `-i` provides XYZ/GJF input. Enables topology-preserving output conversion in TSOPT-only, `scan`, and `path-search` pipelines.	None
`--convert-files / --no-convert-files`	Global toggle for XYZ/TRJ → PDB/mmCIF/GJF companions when templates are available.	`True`
`--dump / --no-dump`	Dump MEP (GSM/DMF), scan, and TS-optimization trajectories. With `all --thermo`, `thermoanalysis.yaml` is always retained for composite-workflow consumption, even under `--no-dump`.	`False`
`--show-config / --no-show-config`	Print the resolved configuration and continue execution.	`no-show-config`
`--dry-run / --no-dry-run`	Validate options and print the execution plan; with `--center`, extraction is run in a temporary directory to validate the derived charge and electron parity. No computational stage runs and no persistent output is written.	`no-dry-run`

Outputs

Directory tree.

```
out_dir/ (default: ./result_all/)
|- summary.log              # formatted summary for quick inspection
|- summary.json             # JSON results
|- mep.pdb / mep.cif / mep_trj.xyz # stitched MEP; topology companions as available
|- mep_w_ref.pdb            # optional aligned full-system inspection composite (--write-
   ref-merge)
|- energy_diagram_*.png     # composite energy diagrams (MLIP / dG MLIP / DFT / dG DFT//
   MLIP)
|- segments/
|  `- seg_NN/               # per-reactive-segment deliverables (2-digit)
|     |- reactant.* ts.* product.*   # canonical R/TS/P (.pdb/.cif/.xyz/.gjf as applicable)
|     `- ts/ irc/ freq/ dft/         # per-stage working files (--tsopt / --thermo / --dft)
`- _work/                   # pipeline scratch (safe to remove)
   |- models/ scan/
   `- path_opt/             # raw MEP-engine output (path_search/ with --refine-path)
```

Console summaries (`summary.log`).

- Pocket charge resolution, YAML contents, scan stages, MEP progress (GSM/DMF), and per-stage timing.
- **[1] Global MEP overview** – image/segment counts, MEP trajectory plot paths, and the aggregate MEP energy diagram.
- **[2] Segment-level MEP summary (UMA path)** – per-segment barriers ($\Delta E^{\ddagger}$), reaction energies (ΔE), and bond-change summaries.
- **[3] Per-segment post-processing (TSOPT / Thermo / DFT)** – per-segment TS imaginary frequency checks, IRC outputs, and MLIP/thermo/DFT energy tables.
- **[4] Energy diagrams (overview)** – diagram tables for MEP/MLIP/Gibbs/DFT series and an optional cross-method summary table.
- **[5] Output directory structure** – a compact tree of generated files with inline annotations.

`summary.json` schema.

- schema_version is 2.0; downstream consumers should validate it before assuming nested keys. execution_status, scientific_status, and scientific_status_reasons distinguish computational completion from scientific usability.
- run_id, pdb2reaction_version, mlip_backend, mlip_model, mlip_precision, out_dir, n_images, and n_segments record run provenance and counts.
- segments – list of per-segment entries with index, tag, kind, barrier_kcal, delta_kcal, and bond_changes.
- post_segments – refined per-segment results. Backend-neutral electronic and thermochemical blocks are named mlip, gibbs_mlip, dft, and gibbs_dft_mlip. energy_diagrams stores the corresponding optional diagram payloads.

S2.4 add-elem-info

add-elem-info repairs the element-symbol columns (77-78) of ATOM/HETATM records in a PDB file.

Usage

```
pdb2reaction add-elem-info -i INPUT.pdb [-o OUTPUT.pdb] [--overwrite / --no-overwrite]
```

CLI options

Table S3: Command-line options for add-elem-info.

Option	Description	Default
-i, --input PATH	Input PDB file.	Required
-o, --out PATH	Output path. When set, --overwrite is ignored.	None → <input>_add_elem.pdb
--overwrite / --no-overwrite	Overwrite the input file in-place when -o/--out is omitted.	False

Outputs

- A PDB file with element symbols populated/corrected (filename per Options table).
- Console report with totals for processed/assigned atoms.

S2.5 extract

extract extracts the active-site model (binding pocket) from protein–substrate complexes. The tool applies distance-based residue selection (with heuristics for disulfides, PRO adjacency, etc.), truncates side chains/backbone segments, optionally appends cap hydrogens, and can process single structures or ensembles.

Usage

```
pdb2reaction extract -i COMPLEX.pdb [COMPLEX2.pdb ...]
                     -c SUBSTRATE_SPEC
                     [-o POCKET.pdb [POCKET2.pdb ...]]
                     [--radius ANG] [--radius-het2het ANG]
                     [--include-h2o / --no-include-h2o]
                     [--exclude-backbone / --no-exclude-backbone]
                     [--add-linkh / --no-add-linkh]
                     [--selected-resn LIST]
                     [--ligand-charge MAP_OR_NUMBER]
                     [-v LEVEL]
```

CLI options

Table S4: Command-line options for `extract`.

Option	Description	Default
`-i, --input PATH...`	One or more protein–ligand PDB/mmCIF files (identical atom ordering required).	Required
`-c, --center SPEC`	Substrate specification (PDB/mmCIF path, residue IDs, or residue names).	Required
`-o, --output PATH...`	Pocket PDB output(s). One path => multi-MODEL, N paths => per input.	Auto (`model.pdb` or `model_<basename>.pdb`)
`-r, --radius FLOAT`	Atom-atom distance cutoff (Å) for inclusion.	`2.6`
`--radius-het2het FLOAT`	Independent hetero-hetero cutoff (Å, non C/H). The default `0.0` disables hetero-hetero inclusion (replaced internally by 0.001 Å); give a positive radius to enable it.	`0.0`
`--include-h2o / --no-include-h2o`	Include HOH/WAT/H2O/DOD/TIP/TIP3/SOL waters.	`True`
`--exclude-backbone / --no-exclude-backbone`	Remove backbone atoms on non-substrate amino acids (PRO/HYP safeguards).	`False`
`--add-linkh / --no-add-linkh`	Add carbon-only cap hydrogens at 1.09 Å along severed bonds.	`True`
`--selected-resn TEXT`	Force-include residues (IDs with optional chains/insertion codes).	`""`
`-l, --ligand-charge TEXT`	Total charge or per-resname mapping (e.g., `GPP:-3,SAM:1`).	`None`
`--modified-residue TEXT`	Single comma-separated list of residue names (with optional `:charge`) to treat as amino acids for backbone truncation and charge assignment, e.g. `'HD1,HD2,HD3'` or `'HD1:0,SEP:-2'`. Passing the option more than once overrides previous values (not a click `multiple` option).	`""`
`--out-json / --no-out-json`	Write `result.json` sidecar (charge summary, atom counts for the first model) next to the output PDB.	`no-out-json`
`-v, --verbose LEVEL`	Console verbosity level, integer `0–3` (0 = silent, 2 = default, 3 = debug).	`2`

Outputs

```
<output>.pdb  # Pocket PDB(s) with optional cap hydrogens after a TER record
               # Single input --> model.pdb by default
               # Multiple inputs without -o --> model_<original_basename>.pdb per structure
               # One -o path with multiple inputs --> single multi-MODEL PDB
```

- Charge summary (protein/ligand/ion/total) is logged for model #1 when verbose mode is enabled.
- Programmatic use returns `{"outputs": [...], "counts": [...], "charge_summary": {...}}`.

S2.6 scan

`scan` performs a staged, bond-length-driven scan using the MLIP calculator and harmonic restraints. Each tuple `(i, j, target_Å)` defines a distance target. At every integration step the temporary targets are updated, the restraint wells are applied, and the entire structure is relaxed with L-BFGS (`--opt-mode` grad, default) or RFOptimizer (`--opt-mode` hess). Optional unbiased pre-optimizations relax the input before the biased walk, and unbiased post-optimizations relax each stage endpoint before writing to disk. When `--scan-lists` is supplied once, the scan runs in a single stage; supplying multiple literals runs sequential stages, each starting from the previous stage's relaxed result.

Usage

```
pdb2reaction scan -i INPUT.{pdb|xyz|trj|...} -q CHARGE [--ligand-charge <number|'RES:Q,...'
    >] [-m MULT] \
                  --scan-lists '[(i,j,target_ang), ...]' [options]
                  [--convert-files / --no-convert-files]
```

CLI options

Table S5: Command-line options for `scan`.

Option	Description	Default
`-i, --input PATH`	Structure file in any format supported by the structure loader (e.g. PDB, mmCIF, XYZ, TRJ).	Required
`-q, --charge INT`	Total charge (CLI > template). When omitted, charge can be inferred from `--ligand-charge`; explicit `-q` overrides any derived value.	Required unless a `.gjf` template or `--ligand-charge` supplies it
`-m, --multiplicity INT`	Spin multiplicity 2S+1 (CLI > template > 1).	`.gjf` template value or `1`
`--scan-lists TEXT`	Repeatable Python literal with `(i,j,target_Å)` tuples. Each literal is one stage. `i`/`j` can be integer indices or PDB atom selectors naming the residue name, residue number, and atom name, separated by whitespace, a comma, or a slash, in any order (`'TYR,285,CA'` and `'GPP 321 C7'` are equivalent), and are remapped internally.	Required
`--one-based / --zero-based`	Interpret atom indices as 1- or 0-based.	`True`
`--max-step-size FLOAT`	Maximum change in any scanned bond per step (Å). Controls the number of integration steps.	`0.20`
`--bias-k FLOAT`	Harmonic bias strength `k` in eV $Å^{-2}$. Overrides `bias.k`.	`300`
`--relax-max-cycles INT`	Cap on optimizer cycles during preopt, each biased step, and end-of-stage cleanups. When omitted, YAML `opt.max_cycles` (or the built-in default) is used; an explicit CLI value overrides YAML.	`100000`
`--opt-mode TEXT`	Relaxation engine at each restrained step: `grad` = L-BFGS (gradient-only, the cheaper and default choice) or `hess` = RFO (Hessian-based, steadier on flat regions but far more costly).	`grad`
`--freeze-links / --no-freeze-links`	When the input has PDB/mmCIF topology, freeze the parents of cap hydrogens.	`True`
`--freeze-atoms TEXT`	Comma-separated 1-based atom indices to freeze explicitly (e.g., `'1,3,5'`); merged with `geom.freeze_atoms`.	`None`
`--dump / --no-dump`	Write per-step optimizer trajectory files; the concatenated stage trajectory `scan_trj.xyz` (with PDB/mmCIF companions via `--convert-files`) is always written regardless of this flag.	`False`
`--convert-files / --no-convert-files`	Toggle XYZ/TRJ → PDB/mmCIF/GJF companions for PDB/mmCIF/Gaussian inputs.	`True`
`--ref-pdb PATH`	Reference PDB/mmCIF topology to use when the input is XYZ/GJF (keeps XYZ coordinates).	None
`-o, --out-dir TEXT`	Output directory root.	`./result_scan/`
`--thresh TEXT`	Convergence preset override (`gau_loose`, `gau`, `gau_tight`, `gau_vtight`, `baker`, `never`).	None (falls back to `gau`)
`--config FILE`	YAML overrides for `geom`, `calc`, `opt`, `lbfgs`, `rfo`, `bias`, `bond`.	None
`--preopt / --no-preopt`	Run an unbiased optimization before scanning.	`False`
`--endopt / --no-endopt`	Run an unbiased optimization after each stage.	`False`
`--print-parsed / --no-print-parsed`	Print the parsed scan-list spec for diagnostics and continue execution.	`no-print-parsed`
`--out-json / --no-out-json`	Write `result.json` summary to `--out-dir`.	`no-out-json`

Outputs

```
out_dir/ (default: ./result_scan/)
|- preopt/                    # Present when --preopt is True
|  |- result.xyz
|  |- result.pdb / result.cif # topology companions when conversion is enabled
|  `- result.gjf              # When a Gaussian template exists and conversion is enabled
`- stage_XX/                  # One folder per stage
```

```
    |- result.xyz
    |- result.pdb / result.cif # topology companions of the final structure
    |- result.gjf             # Gaussian companion when templates exist and conversion is
   enabled
    |- scan_trj.xyz           # Stitched scan trajectory (always written)
    `- scan.pdb / scan.cif    # trajectory companions when conversion is enabled
```

- Console summaries of the resolved `geom`, `calc`, `opt`, `bias`, `bond`, and optimizer blocks, and per-stage bond-change reports.

S2.7 `scan2d`

`scan2d` performs a two-distance (d1, d2) grid scan using harmonic restraints. A single `--scan-lists` literal with two quadruples is supplied as `(i, j, lowÅ, highÅ)`; the tool constructs linear grids for both ranges using `--max-step-size`, then visits each new grid point starting from the previously optimized structure nearest to it. Each grid point is relaxed and written alongside a CSV/figure bundle. Energies reported in `surface.csv` are always evaluated without bias for direct comparison across grid points.

Usage

```
pdb2reaction scan2d -i INPUT.{pdb|xyz|trj|...} -q CHARGE [--ligand-charge <number|'RES:Q
    ,...'>] [-m MULT] \
                    --scan-lists '[(i,j,low_A,high_A), (i,j,low_A,high_A)]' [options]
                    [--convert-files / --no-convert-files]
```

CLI options

Table S6: Command-line options for `scan2d`.

Option	Description	Default
`-i, --input PATH`	Structure file in any format supported by the structure loader (e.g. PDB, mmCIF, XYZ, TRJ).	Required
`-q, --charge INT`	Total charge (CLI > template). Overrides `--ligand-charge` when both are set; unused and recorded as null in `--csv` plot-only mode.	Required unless a template or `--ligand-charge` supplies it, or `--csv` is given
`-m, --multiplicity INT`	Spin multiplicity 2S+1 (CLI > template > 1); unused and recorded as null in `--csv` plot-only mode.	`.gjf` template value or `1`; null with `--csv`
`-s, --scan-lists TEXT`	**Single** Python literal with two quadruples `(i,j,lowÅ,highÅ)`. `i`/`j` can be integer indices or PDB atom selectors naming the residue name, residue number, and atom name, separated by whitespace, a comma, or a slash, in any order (`'TYR,285,CA'` and `'GPP 321 C7'` are equivalent), and are remapped internally.	Required
`--one-based / --zero-based`	Interpret `(i, j)` indices as 1- or 0-based.	`True`
`--max-step-size FLOAT`	Maximum change allowed for either distance per increment (Å). Determines the grid density.	`0.20`
`--bias-k FLOAT`	Harmonic bias strength `k` in eV Å^{-2}. Overrides `bias.k`.	`300`
`--relax-max-cycles INT`	Maximum optimizer cycles during each biased relaxation. When omitted, YAML `opt.max_cycles` (or the built-in default) is used; an explicit CLI value overrides YAML.	`100000`
`--opt-mode TEXT`	Relaxation engine at each restrained step: `grad` = L-BFGS (gradient-only, the cheaper and default choice) or `hess` = RFO (Hessian-based, steadier on flat regions but far more costly).	`grad`
`--freeze-links / --no-freeze-links`	When the input has PDB/mmCIF topology, freeze parents of cap hydrogens.	`True`
`--freeze-atoms TEXT`	Comma-separated 1-based atom indices to freeze explicitly (e.g., `'1,3,5'`); merged with `geom.freeze_atoms`.	`None`
`--dump / --no-dump`	Write `inner_path_d1_###_trj.xyz` for each outer step.	`False`

Continued on next page

Option	Description	Default
`--convert-files / --no-convert-files`	Toggle XYZ/TRJ → PDB/mmCIF/GJF companions for PDB/mmCIF/Gaussian inputs.	`True`
`--ref-pdb PATH`	Reference PDB/mmCIF topology to use when the input is XYZ/GJF (keeps XYZ coordinates).	None
`-o, --out-dir TEXT`	Output directory root for grids and plots.	`./result_scan2d/`
`--thresh TEXT`	Convergence preset override (`gau_loose`, `gau`, `gau_tight`, `gau_vtight`, `baker`, `never`).	`baker`
`--config FILE`	YAML overrides for `geom`, `calc`, `opt`, `lbfgs`, `rfo`, `bias`.	None
`--preopt / --no-preopt`	Run an unbiased optimization before scanning.	`False`
`--baseline {min,first}`	Shift kcal/mol energies so the global min or first grid point is zero.	`min`
`--zmin FLOAT`, `--zmax FLOAT`	Manual limits for the contour/surface color scale (kcal/mol).	Autoscaled
`--print-parsed / --no-print-parsed`	Print the parsed scan-list spec for diagnostics and continue execution.	`no-print-parsed`
`--out-json / --no-out-json`	Write `result.json` summary to `--out-dir`.	`no-out-json`

Outputs

```
out_dir/ (default: ./result_scan2d/)
|- surface.csv                 # Structured grid table
|- scan2d_map.png              # 2D contour (requires Kaleido; the run stops if PNG export
   fails)
|- scan2d_landscape.html       # 3D surface visualization
|- grid/preopt_i###_j###.xyz  # Unbiased relaxation of the initial structure (one file,
   tagged by its
|                              #   own measured distances; written when --preopt is True)
|- grid/point_i###_j###.xyz   # Relaxed geometries for every (i, j) pair (Ang x 100 tags)
|- grid/point_i###_j###.{pdb,cif} # topology companions when templates exist
|- grid/point_i###_j###.gjf   # Gaussian companions when templates exist and conversion is
   enabled
`- grid/inner_path_d1_###_trj.xyz # Present only when --dump is True (topology companions
   as available)
```

S2.8 `scan3d`

`scan3d` performs a three-distance grid scan with harmonic restraints using the MLIP calculator. The input is a single `--scan-lists` literal containing three quadruples `(i, j, lowÅ, highÅ)`. The tool builds linear grids for each distance with `--max-step-size`, then visits each new grid point starting from the previously optimized structure nearest to it (loops nested outer d1, middle d2, inner d3). Each grid point is relaxed with the appropriate restraints active; unbiased energies are recorded for direct comparison of grid points. A precomputed `surface.csv` can also be visualized without rerunning the scan.

Usage

```
pdb2reaction scan3d -i INPUT.{pdb|xyz|trj|...} -q CHARGE [--ligand-charge <number|'RES:Q
   ,...'>] [-m MULT] \
                    --scan-lists '[(i,j,low_A,high_A), (i,j,low_A,high_A), (i,j,low_A,
   high_A)]' [options] \
                    [--convert-files / --no-convert-files]
```

CLI options

Table S7: Command-line options for `scan3d`.

Option	Description	Default
`-i, --input PATH`	Structure file in any format supported by the structure loader (e.g. PDB, mmCIF, XYZ, TRJ).	Required unless `--csv` is given

Continued on next page

Option	Description	Default
`-q, --charge INT`	Total charge (CLI > template). Overrides `--ligand-charge` when both are set; unused and recorded as null in `--csv` plot-only mode.	Required unless a template or `--ligand-charge` supplies it, or `--csv` is given
`-m, --multiplicity INT`	Spin multiplicity 2S+1 (CLI > template > 1); unused and recorded as null in `--csv` plot-only mode.	`.gjf` template value or `1`; null with `--csv`
`-s, --scan-lists TEXT`	**Single** Python literal with three quadruples `(i,j,lowÅ,highÅ)`. `i`/`j` can be integer indices or PDB atom selectors naming the residue name, residue number, and atom name, separated by whitespace, a comma, or a slash, in any order (`'TYR,285,CA'` and `'GPP 321 C7'` are equivalent), and are remapped internally.	Required unless `--csv` is given
`--one-based / --zero-based`	Interpret `(i, j)` indices as 1- or 0-based.	`True`
`--max-step-size FLOAT`	Maximum change allowed per distance increment (Å). Controls grid density.	`0.20`
`--bias-k FLOAT`	Harmonic bias strength `k` in eV Å^{-2}. Overrides `bias.k`.	`300`
`--relax-max-cycles INT`	Maximum optimizer cycles during each biased relaxation. When omitted, YAML `opt.max_cycles` (or the built-in default) is used; an explicit CLI value overrides YAML.	`100000`
`--opt-mode TEXT`	Relaxation engine at each restrained step: `grad` = L-BFGS (gradient-only, the cheaper and default choice) or `hess` = RFO (Hessian-based, steadier on flat regions but far more costly).	`grad`
`--freeze-links / --no-freeze-links`	When the input has PDB/mmCIF topology, freeze parents of cap hydrogens.	`True`
`--freeze-atoms TEXT`	Comma-separated 1-based atom indices to freeze explicitly (e.g., `'1,3,5'`); merged with `geom.freeze_atoms`.	`None`
`--dump / --no-dump`	Write `inner_path_d1_###_d2_###_trj.xyz` for each (d1, d2).	`False`
`--convert-files / --no-convert-files`	Toggle XYZ/TRJ → PDB/mmCIF/GJF companions for PDB/mmCIF/Gaussian inputs.	`True`
`--ref-pdb PATH`	Reference PDB/mmCIF topology to use when the input is XYZ/GJF (keeps XYZ coordinates).	None
`-o, --out-dir TEXT`	Output directory root for grids and plots.	`./result_scan3d/`
`--csv PATH`	Load an existing `surface.csv` and only plot it (no new scan).	None
`--thresh TEXT`	Convergence preset override (`gau_loose`, `gau`, `gau_tight`, `gau_vtight`, `baker`, `never`).	`baker`
`--config FILE`	YAML overrides for `geom`, `calc`, `opt`, `lbfgs`, `rfo`, `bias`.	None
`--preopt / --no-preopt`	Run an unbiased optimization before scanning.	`False`
`--baseline {min,first}`	Shift kcal/mol energies so the global min or `(i,j,k)=(0,0,0)` is zero.	`min`
`--zmin FLOAT, --zmax FLOAT`	Manual limits for the isosurface color bands (kcal/mol).	Autoscaled
`--print-parsed / --no-print-parsed`	Print the parsed scan-list spec for diagnostics and continue execution.	`no-print-parsed`
`--out-json / --no-out-json`	Write `result.json` summary to `--out-dir`.	`no-out-json`

Outputs

```
out_dir/ (default: ./result_scan3d/)
|- surface.csv                      # Grid metadata; may include a reference row (i=j=k=-1)
|- scan3d_density.html              # 3D energy isosurface visualization
|- grid/point_i###_j###_k###.xyz    # Relaxed geometry for each grid point (Ang x 100 tags)
|- grid/point_i###_j###_k###.{pdb,cif} # topology companions when templates exist
|- grid/point_i###_j###_k###.gjf    # Gaussian companions when templates exist and
   conversion is enabled
|- grid/preopt_i###_j###_k###.xyz   # Starting structure saved before scanning (preoptimized
    when --preopt is True)
`- grid/inner_path_d1_###_d2_###_trj.xyz # Present only when --dump is True (mirrored to .
   pdb with conversion)
```

S2.9 opt

`opt` performs a single-structure geometry optimization with the pysisyphus L-BFGS (`grad`) or RFO (`hess`) engines while the MLIP backend provides energies, gradients, and Hessians. Input structures can be `.pdb`, `.xyz`, `.trj`, or any format supported by the structure loader.

Usage

```
pdb2reaction opt -i INPUT.{pdb|xyz|trj|...} -q CHARGE [--ligand-charge <number|'RES:Q,...'
    >] -m MULT \
                  [--opt-mode grad|hess] [--freeze-links / --no-freeze-links] \
                  [--dist-freeze '[(i,j,target_A), ...]'] [--one-based / --zero-based] \
                  [--bias-k K_eV_per_A2] [--dump / --no-dump] [--out-dir DIR] \
                  [--max-cycles N] [--thresh PRESET] [--reject-uphill] [--stop-plateau] [--
    config FILE] \
                  [--convert-files / --no-convert-files]
```

CLI options

Table S8: Command-line options for `opt`.

Option	Description	Default
`-i, --input PATH`	Input structure in any format supported by the structure loader (e.g. PDB, mmCIF, XYZ, TRJ).	Required
`-q, --charge INT`	Total charge. Required for non-`.gjf` inputs unless `--ligand-charge` supplies it (a `.gjf` template already encodes charge). Overrides `--ligand-charge` when both are set.	Required unless derivable
`-m, --multiplicity INT`	Spin multiplicity (2S+1). Falls back to `.gjf` template or `1`.	Template/`1`
`--dist-freeze TEXT`	Inline Python-literal string describing `(i,j,target_A)` tuples (or a YAML/JSON spec file path) for harmonic restraints.	None
`--one-based / --zero-based`	Interpret `--dist-freeze` indices as 1-based (default) or 0-based.	`True`
`--bias-k FLOAT`	Harmonic bias strength applied to every `--dist-freeze` tuple (eV Å^{-2}).	`300`
`--freeze-links / --no-freeze-links`	Toggle cap-hydrogen parent freezing (PDB/mmCIF input, or XYZ/GJF with `--ref-pdb`).	`True`
`--freeze-atoms TEXT`	Comma-separated 1-based atom indices to freeze explicitly (e.g., `'1,3,5'`). Works on any input format; complements `--freeze-links` for inputs without PDB/mmCIF topology.	`None`
`--ref-pdb PATH`	Reference PDB/mmCIF topology to use when the input is XYZ/GJF (keeps XYZ coordinates).	None
`--max-cycles INT`	Hard limit on optimization iterations (`opt.max_cycles`).	`100000`
`--opt-mode [grad|hess|lbfgs|rfo]`	Choose optimizer: `grad` / `lbfgs` = L-BFGS, `hess` / `rfo` = RFO.	`grad`
`--reject-uphill / --no-reject-uphill`	Opt in to rejecting uphill RFO trials; ignored in gradient/L-BFGS mode.	`False`
`--stop-plateau / --no-stop-plateau`	Opt-in wasted-cycle stop when the recent energy range is flat; reports `stalled`, not converged.	`False`
`--stop-plateau-thresh FLOAT, --stop-plateau-window INT`	Energy range (Hartree) and number of cycles inspected when the plateau stop is enabled.	`1e-4`, `50`
`--flatten / --no-flatten`	Enable/disable imaginary-mode flatten loop after optimization.	`False`
`--dump / --no-dump`	Emit trajectory dumps (`optimization_trj.xyz`).	`False`
`--convert-files / --no-convert-files`	Enable or disable XYZ/TRJ → PDB/mmCIF companions for PDB/mmCIF inputs and XYZ → GJF companions for Gaussian templates.	`True`

Continued on next page

Option	Description	Default
`-o, --out-dir TEXT`	Output directory for all files.	`./result_opt/`
`--thresh TEXT`	Override convergence preset (`gau_loose`, `gau`, `gau_tight`, `gau_vtight`, `baker`, `never`).	None (falls back to `gau`)
`--config FILE`	Supply YAML overrides (sections `geom`, `calc`, `opt`, `lbfgs`, `rfo`, and the shared cutoff in `freq`).	None
`--out-json / --no-out-json`	Write `result.json` summary to `--out-dir`.	`no-out-json`

Outputs

```
out_dir/
|- final_geometry.xyz          # Always written
|- final_geometry.{pdb,cif}    # Topology companions when conversion is enabled
|- final_geometry.gjf          # When a Gaussian template was detected and conversion is
    enabled
|- optimization_trj.xyz         # Only if dumping is enabled
|- optimization.{pdb,cif}       # Topology companions of the trajectory
`- restart*.yml                 # Optional restarts when opt.dump_restart is set
```

S2.10 `sp`

`sp` evaluates a single-point MLIP energy and forces on one structure, and optionally its Hessian. It performs no geometry optimization and serves as the lightweight entry point for scoring a fixed geometry (for example, an externally optimized structure) or for pre-computing a Hessian. Backend, precision, and active-site options follow the shared *Common options* described above.

Usage

```
pdb2reaction sp -i INPUT.{pdb|cif|xyz|gjf} [-q CHARGE] [--ligand-charge <number|'RES:Q,...'
    >] [-m MULT] \
                [--hess / --no-hess] [--hessian-calc-mode Analytical|FiniteDifference] \
                [--out-dir DIR] [--out-json / --no-out-json] [--config FILE]
```

CLI options

Table S9: Command-line options for `sp`.

Option	Description	Default
`-i, --input FILE`	Input structure (PDB / mmCIF / XYZ / GJF).	Required
`-q, --charge INT`	Total charge. Required for non-`.gjf` inputs unless `--ligand-charge` is provided.	Required (or .gjf/`-l`)
`-m, --multiplicity INT`	Spin multiplicity (2S+1).	`.gjf` template value or `1`
`--hess / --no-hess`	Also compute a Hessian and save it to `hessian.npy`; with frozen atoms configured this is the active-DOF block, otherwise it is the full $3N \times 3N$ matrix.	`no-hess`
`--hessian-calc-mode CHOICE`	Hessian backend when `--hess` is set (`Analytical` or `FiniteDifference`). All built-in backends support an explicit analytical request.	`FiniteDifference`
`-o, --out-dir TEXT`	Output directory.	`./result_sp/`
`--config FILE`	YAML config file with sections (`calc`, `geom`, ...).	None
`--out-json / --no-out-json`	Write `result.json` summary to `--out-dir`.	`no-out-json`

S2.11 `path-opt`

`path-opt` searches for a minimum-energy-path (MEP) between two endpoint structures using pysisyphus' growing string method (GSM) or Direct-MaxFlux (DMF) selected via `--mep-mode`. When `--convert-files` is enabled (default), outputs are mirrored to `.pdb`/`.cif` or multi-geometry `.gjf` companions when the endpoints originate from PDB/mmCIF or Gaussian templates.

Usage

```
pdb2reaction path-opt -i REACTANT.{pdb|cif|xyz} PRODUCT.{pdb|cif|xyz} [-q CHARGE] [--ligand
    -charge <number|'RES:Q,...'>] -m MULT \
                       [--mep-mode {gsm|dmf}] [--freeze-links / --no-freeze-links] [--max-
    nodes N] \
                       [--max-cycles-gsm N] [--max-cycles-dmf N] \
                       [--climb / --no-climb] [--dump / --no-dump] [--thresh PRESET] \
                       [--thresh-gsm PRESET] [--thresh-dmf TOL] \
                       [--out-dir DIR] [--config FILE] \
                       [--convert-files / --no-convert-files]
```

CLI options

Table S10: Command-line options for `path-opt`.

Option	Description	Default
`-i, --input PATH PATH`	Reactant and product structures (PDB/mmCIF/XYZ/GJF).	Required
`-q, --charge INT`	Total charge (`calc.charge`). Required unless the input is a `.gjf` template that already encodes charge. Overrides `--ligand-charge` when both are set.	Required when not in template
`-m, --multiplicity INT`	Spin multiplicity (`calc.spin`).	Template/1
`--freeze-links / --no-freeze-links`	Requires a PDB/mmCIF topology (PDB/mmCIF input, or XYZ/GJF with `--ref-pdb`): freeze cap-hydrogen parents (merged with YAML).	`True`
`--freeze-atoms TEXT`	Comma-separated 1-based atom indices to freeze explicitly (e.g., `'1,3,5'`); merged with `geom.freeze_atoms`.	`None`
`--max-nodes INT`	Number of internal nodes (string images = `max_nodes + 2`).	`20`
`--gsm-param {equi|energy}`	GSM node parameterization after string growth.	`equi`
`--mep-mode {gsm|dmf}`	Select GSM or DMF path generator.	`gsm`
`--dmf-backend {cpu|gpu}`	DMF compute backend (`--mep-mode dmf` only): `gpu` (`dmf.torch`/CUDA) or `cpu` (`dmf`/NumPy); retry `cpu` on a GPU out-of-memory error.	`gpu`
`--max-cycles-gsm INT`	GSM string-optimizer cycle cap (`stopt.max_cycles`).	`300`
`--max-cycles-dmf INT`	DMF IPOPT iteration cap (`dmf.max_cycles`).	`300`
`--climb / --no-climb`	Enable climbing-image refinement (and Lanczos tangent).	`True`
`--dump / --no-dump`	Dump MEP trajectories/restarts (GSM/DMF).	`False`
`--opt-mode TEXT`	Single-structure optimizer for endpoint pre-optimization (`grad` = L-BFGS, `hess` = RFO).	`grad`
`--convert-files / --no-convert-files`	Toggle XYZ/TRJ → PDB/mmCIF/GJF companions for PDB/mmCIF/Gaussian inputs.	`True`
`--ref-pdb PATH`	Reference PDB/mmCIF topology to use when the input is XYZ/GJF (keeps XYZ coordinates).	None
`-o, --out-dir TEXT`	Output directory.	`./result_path_opt/`
`--thresh TEXT`	Override convergence preset for endpoint pre-optimization only (`gau_loose`, `gau`, `gau_tight`, `gau_vtight`, `baker`, `never`).	None (falls back to `gau`)
`--thresh-gsm TEXT`	Convergence preset for the GSM string optimizer.	`gau_loose`
`--thresh-dmf TEXT`	DMF IPOPT dual-infeasibility tolerance: `tight`, `middle`, `loose`, or a positive float.	`tight`
`--config FILE`	YAML overrides; see Section S3.	None
`--preopt / --no-preopt`	Pre-optimize each endpoint with the selected single-structure optimizer before alignment/MEP search (GSM/DMF).	`True`
`--preopt-max-cycles INT`	Cap for endpoint pre-optimization cycles.	`100000`
`--fix-ends / --no-fix-ends`	Keep the endpoint geometries fixed during GSM growth/refinement.	`--fix-ends`
`--out-json / --no-out-json`	Write `result.json` summary to `--out-dir`.	`no-out-json`

Outputs

```
out_dir/
|- final_geometries_trj.xyz     # XYZ path; comment line holds energies when provided
|- final_geometries.{pdb,cif}  # Topology companions when conversion is enabled
|- hei.xyz                      # Highest-energy image with its energy on the comment line
|- hei.{pdb,cif}                # HEI topology companions when conversion is enabled
|- hei.gjf                      # HEI written using a detected Gaussian template (conversion
     enabled)
|- align_refine/               # Intermediate files from the rigid alignment/refinement stage
    (created only when scan output exists)
`- <optimizer dumps/restarts>  # Present when dumping is enabled
```

S2.12 `path-search`

`path-search` constructs a continuous minimum-energy-path (MEP) across two or more structures ordered along a reaction coordinate by chaining GSM or DMF segments (selected via `--mep-mode`; GSM is the default) and recursively refining segments with covalent changes. Format-aware conversions mirror trajectories and HEI snapshots into `.pdb`/`.cif` or multi-geometry `.gjf` companions when `--convert-files` is enabled (default) and matching templates exist.

Usage

```
pdb2reaction path-search -i R.pdb [I.pdb ...] P.pdb [-q CHARGE] [--ligand-charge <number|'
    RES:Q,...'>] [--multiplicity 2S+1]
                         [--mep-mode {gsm|dmf}] [--freeze-links / --no-freeze-links] [--
    thresh PRESET]
                         [--thresh-gsm PRESET] [--thresh-dmf TOL]
                         [--refine-mode {peak|minima}]
                         [--max-nodes N] [--max-cycles-gsm N] [--max-cycles-dmf N] [--climb
     / --no-climb]
                         [--opt-mode grad|hess] [--dump / --no-dump]
                         [--out-dir DIR] [--preopt / --no-preopt]
                         [--align / --no-align] [--ref-pdb FILE ...]
                         [--convert-files / --no-convert-files]
                         [--config FILE]
```

CLI options

Table S11: Command-line options for `path-search`.

Option	Description	Default
`-i, --input PATH...`	Two or more structures in reaction order (reactant → product). Repeat `-i` or pass multiple paths after one flag.	Required
`-q, --charge INT`	Total charge. Required unless a `.gjf` input supplies charge metadata or `--ligand-charge` derives it from PDB/mmCIF inputs. Overrides `--ligand-charge` when both are set.	Required unless derivable
`-m, --multiplicity INT`	Spin multiplicity (2S+1).	`.gjf` template value or `1`
`--freeze-links / --no-freeze-links`	When loading PDB/mmCIF pockets, freeze the parent atoms of cap hydrogens.	`True`
`--freeze-atoms TEXT`	Comma-separated 1-based atom indices to freeze explicitly (e.g., `'1,3,5'`); merged with `geom.freeze_atoms`.	`None`
`--max-nodes INT`	Internal nodes per MEP segment (GSM string images or DMF images).	`20`
`--gsm-param {equi|energy}`	GSM node parameterization after string growth.	`equi`
`--max-cycles-gsm INT`	GSM string-optimizer cycle cap (`stopt.max_cycles`).	`300`
`--max-cycles-dmf INT`	DMF IPOPT iteration cap (`dmf.max_cycles`).	`300`
`--climb / --no-climb`	Enable the climbing image for standard GSM segments; bridge segments always disable climbing.	`True`
`--opt-mode TEXT`	Single-structure optimizer for HEI±1/kink nodes. `grad` maps to L-BFGS; `hess` maps to RFO.	`grad`

Continued on next page

Option	Description	Default
`--mep-mode {gsm|dmf}`	Segment generator (GSM or DMF).	`gsm`
`--dmf-backend {cpu|gpu}`	DMF compute backend (`--mep-mode dmf` only): `gpu` (`dmf.torch`/CUDA) or `cpu` (`dmf`/NumPy); retry `cpu` on a GPU out-of-memory error.	`gpu`
`--refine-mode {peak|minima}`	Seeds for refinement: `peak` optimizes HEI±1; `minima` searches outward from the HEI toward the nearest local minima on each side. Defaults to `peak` for GSM and `minima` for DMF when omitted.	*Auto*
`--dump / --no-dump`	Dump MEP (GSM/DMF) and single-structure trajectories/restarts.	`False`
`--convert-files / --no-convert-files`	Toggle XYZ/TRJ → PDB/mmCIF/GJF companions for PDB/mmCIF or Gaussian inputs.	`True`
`-o, --out-dir TEXT`	Output directory.	`./result_path_search/`
`--thresh TEXT`	Override convergence preset for single-structure optimizations only (`gau_loose`, `gau`, `gau_tight`, `gau_vtight`, `baker`, `never`).	None (falls back to `gau`)
`--thresh-gsm TEXT`	Convergence preset for the GSM string optimizer.	`gau_loose`
`--thresh-dmf TEXT`	DMF IPOPT dual-infeasibility tolerance: `tight`, `middle`, `loose`, or a positive float.	`tight`
`--config FILE`	YAML overrides (see below).	None
`--preopt / --no-preopt`	Pre-optimize each endpoint before MEP search.	`True`
`--align / --no-align`	Align all inputs to the first structure before searching.	`--align`
`--ref-full-pdb PATH`	Static full-size PDB/mmCIF template used for optional aligned inspection composites; use the template corresponding to the first input.	None
`--write-ref-merge / --no-write-ref-merge`	Write aligned `mep_w_ref`/`hei_w_ref` coordinate composites; requires `--align` and `--ref-full-pdb`.	`False`
`--ref-pdb PATH...`	Pocket reference PDB/mmCIF structures used for the final full-system merge. Useful when `--input` uses XYZ/GJF intermediates but topology snapshots exist for merging.	None

Outputs

```
out_dir/ (default: ./result_path_search/)
|- mep_trj.xyz              # Primary MEP trajectory
|- mep.pdb / mep.cif        # Topology companions when conversion is enabled
|- mep_w_ref.pdb            # Optional full-system inspection composite (--write-ref-merge)
|- mep_w_ref_seg_XX.pdb     # Optional per-segment composites (--write-ref-merge)
|- summary.json             # Barrier and classification summary for every recursive segment
|- mep_plot.png             # ΔE profile generated via `trj2fig` (kcal/mol, reactant
    reference)
|- energy_diagram_MEP.png   # State-energy diagram (kcal/mol, reactant reference)
`- seg_NNN[_refine|_bridge|_maxdepth]_mep/ # GSM/DMF dumps, HEI snapshots, refinement
    diagnostics per segment
```

- Console reports covering resolved configuration blocks (`geom`, `calc`, `gs`, `opt`, `stopt.*`, `bond`, `search`).

S2.13 `tsopt`

`tsopt` optimizes transition states using two complementary workflows: `grad` (Hessian-initialized dimer with L-BFGS) and `hess` (restricted-step partitioned rational function optimization, RS-P-RFO, following Banerjee; default).

Usage

```
pdb2reaction tsopt -i INPUT.{pdb|xyz|trj|...} [-q CHARGE] [--ligand-charge <number|'RES:Q
    ,...'>] [-m 2S+1] \
                    [--opt-mode grad|hess] [--ref-mode FILE] \
                    [--freeze-links / --no-freeze-links] [--max-cycles N] [--thresh PRESET]
     \
                    [--stop-plateau / --no-stop-plateau] \
                    [--dump / --no-dump] [--out-dir DIR] [--config FILE] \
                    [--hessian-calc-mode Analytical|FiniteDifference] \
```

```
                    [--convert-files / --no-convert-files]
```

CLI options

Table S12: Command-line options for `tsopt`.

Option	Description	Default
`-i, --input PATH`	Structure file in any format supported by the structure loader (e.g. PDB, mmCIF, XYZ, TRJ).	Required
`--ref-mode PATH`	Cartesian reference direction for Hessian-based TS root selection; `all` supplies the MEP tangent by default.	None
`-q, --charge INT`	Total charge. Required unless `--ligand-charge` is supplied or the input is a `.gjf` template with charge metadata. Overrides `--ligand-charge` when both are set.	Required (or `--ligand-charge` / .gjf metadata)
`-m, --multiplicity INT`	Spin multiplicity (2S+1).	`.gjf` template value or `1`
`--freeze-links / --no-freeze-links`	PDB/mmCIF input (or XYZ/GJF with `--ref-pdb`). Freeze parents of cap hydrogens (merged into `geom.freeze_atoms`).	`True`
`--freeze-atoms TEXT`	Comma-separated 1-based atom indices to freeze explicitly (e.g., `'1,3,5'`); merged with `geom.freeze_atoms`.	`None`
`--max-cycles INT`	Macro-cycle cap forwarded to `opt.max_cycles`.	`100000`
`--flatten / --no-flatten`	Enable the extra-imaginary-mode flattening loop (grad: dimer loop, hess: post-RS-P-RFO).	`False`
`--opt-mode [grad|hess|dimer|rsirfo|trim|rsprfo]`	TS optimizer: `grad` / `dimer` → Hessian-initialized dimer; `hess` / `rsprfo` → RS-P-RFO (Banerjee, default); `rsirfo` → RS-I-RFO; `trim` → TRIM (Helgaker).	`hess`
`--dump / --no-dump`	Dump trajectories.	`False`
`-o, --out-dir TEXT`	Output directory.	`./result_tsopt/`
`--thresh TEXT`	Override convergence preset (`gau_loose`, `gau`, `gau_tight`, `gau_vtight`, `baker`, `never`).	None (falls back to `baker`)
`--stop-plateau / --no-stop-plateau`	Opt-in energy-plateau stop; it reports `stalled`, retains the final geometry, and does not certify convergence.	`False`
`--stop-plateau-thresh FLOAT, --stop-plateau-window INT`	Energy range (Hartree) and window used when the plateau stop is enabled.	`1e-4, 50`
`--hessian-calc-mode CHOICE`	MLIP Hessian mode (`Analytical` or `FiniteDifference`).	`FiniteDifference`
`--convert-files / --no-convert-files`	Toggle XYZ/TRJ → PDB/mmCIF/GJF companions for PDB/mmCIF or Gaussian inputs.	`True`
`--ref-pdb PATH`	Reference PDB/mmCIF topology to use when the input is XYZ/GJF (keeps XYZ coordinates).	None
`--config FILE`	YAML overrides (`geom`, `calc`, `opt`, `hessian_dimer`, `rsirfo`, and the shared cutoff in `freq`).	None
`--out-json / --no-out-json`	Write `result.json` summary to `--out-dir`.	`no-out-json`

For Hessian-based TS optimization, terminal exact PHVA is performed only after numerical convergence. A non-converged or plateau-stalled run retains its final geometry but skips terminal PHVA and is not certified as a first-order TS; certification requires exactly one resolved mode below −`freq.zero_cutoff_cm` after convergence (default cutoff 5.0 cm^{-1}).

Outputs

```
out_dir/ (default: ./result_tsopt/)
|- final_geometry.xyz       # TS geometry (always written)
|- final_geometry.{pdb,cif} # Topology companions when conversion is enabled
|- final_geometry.gjf       # Gaussian companion when a template exists and conversion is
   enabled
|- result.json               # Structured summary (imag-freq count, status, config) when --
   out-json
`- <optimizer dumps/restarts> # Present when --dump is True
```

- Console summaries of resolved `geom`, `calc`, `opt`, `hessian_dimer` (grad) or `rsirfo` (hess) blocks and imaginary-frequency diagnostics.

S2.14 `irc`

`irc` runs EulerPC-based Intrinsic Reaction Coordinate (IRC) calculation from a TS structure.

Usage

```
pdb2reaction irc -i INPUT.{pdb|xyz|trj|...} [-q CHARGE] [--ligand-charge <number|'RES:Q,...
    '>] [-m 2S+1]
                 [--max-cycles N] [--step-size Δs] [--root k]
                 [--never-stop / --no-never-stop]
                 [--forward / --no-forward] [--backward / --no-backward]
                 [--freeze-links / --no-freeze-links]
                 [--out-dir DIR]
                 [--convert-files / --no-convert-files]
                 [--hessian-calc-mode Analytical|FiniteDifference]
                 [--config FILE]
```

CLI options

Table S13: Command-line options for `irc`.

Option	Description	Default
`-i, --input PATH`	Transition-state structure in any format supported by the structure loader (e.g. PDB, mmCIF, XYZ).	Required
`-q, --charge INT`	Total charge; overrides `calc.charge`. Required for non-`.gjf` inputs unless `--ligand-charge` supplies it. Overrides `--ligand-charge` when both are set.	Required unless derivable
`-m, --multiplicity INT`	Spin multiplicity (2S+1); overrides `calc.spin`.	`.gjf` template value or `1`
`--max-cycles INT`	Maximum IRC steps (`irc.max_cycles`).	`125`
`--step-size FLOAT`	Step length in Bohr along unweighted Cartesians (`irc.step_length`).	`0.10`
`--never-stop / --no-never-stop`	Ignore physical gradient and energy endpoint criteria and trace to `--max-cycles`; numerical failures and interruption still stop the branch.	`False`
`--root INT`	Imaginary-mode index for the initial displacement (`irc.root`).	`0`
`--irc-pos-def / --no-irc-pos-def`	Require a positive-definite Hessian at IRC convergence (blocks shoulder false-convergence); applied only when set, otherwise the integrator's own convergence criterion is used.	unset
`--forward / --no-forward`	Run forward branch (`irc.forward`).	`--forward`
`--backward / --no-backward`	Run backward branch (`irc.backward`).	`--backward`
`--freeze-links / --no-freeze-links`	For PDB/mmCIF inputs, freeze cap-hydrogen parents (merged with `geom.freeze_atoms`).	`True`
`--freeze-atoms TEXT`	Comma-separated 1-based atom indices to freeze explicitly (e.g., `'1,3,5'`); merged with `geom.freeze_atoms`.	`None`
`-o, --out-dir TEXT`	Output directory (`irc.out_dir`).	`./result_irc/`
`--convert-files / --no-convert-files`	Toggle XYZ/TRJ → PDB/mmCIF companions for PDB/mmCIF inputs.	`True`
`--ref-pdb PATH`	Reference PDB/mmCIF topology to use when the input is XYZ/GJF (keeps XYZ coordinates).	None
`--hessian-calc-mode CHOICE`	MLIP Hessian mode (`calc.hessian_calc_mode`).	`FiniteDifference`
`--config FILE`	YAML overrides (see below).	None
`--out-json / --no-out-json`	Write `result.json` summary to `--out-dir`.	`no-out-json`

Outputs

```
out_dir/ (default: ./result_irc/)
|- <prefix>finished_irc_trj.xyz   # Concatenated forward+backward IRC trajectory
|- <prefix>forward_irc_trj.xyz     # Present when the forward branch runs
|- <prefix>backward_irc_trj.xyz    # Present when the backward branch runs
`- *.{pdb,cif}                  # Trajectory companions when conversion is enabled
```

- Console summaries of resolved `geom`, `calc`, and `irc` configurations plus wall-clock timing.

S2.15 freq

`freq` performs vibrational analysis, honoring any frozen atoms via partial Hessian vibrational analysis (PHVA). It exports normal modes as `.trj`/`.pdb` animations, prints a Gaussian-style thermochemistry summary when the optional `thermoanalysis` package is installed, and can emit a YAML summary (`thermoanalysis.yaml`) when `--dump` is enabled.

Usage

```
pdb2reaction freq -i INPUT.{pdb|xyz|trj|...} [-q CHARGE] [--ligand-charge <number|'RES:Q
    ,...'>] [-m 2S+1] \
                   [--freeze-links / --no-freeze-links] \
                   [--max-write N] [--amplitude-ang ANG] [--n-frames N] \
                   [--sort value|abs] [--out-dir DIR] [--config FILE] \
                   [--temperature K] [--pressure atm] [--dump / --no-dump] \
                   [--hessian-calc-mode Analytical|FiniteDifference] \
                   [--convert-files / --no-convert-files]
```

CLI options

Table S14: Command-line options for `freq`.

Option	Description	Default
`-i, --input PATH`	Structure file in any format supported by the structure loader (e.g. PDB, mmCIF, XYZ, TRJ).	Required
`-q, --charge INT`	Total charge. When omitted, charge can be inferred from `--ligand-charge`; explicit `-q` overrides any derived value.	Required unless a `.gjf` template or `--ligand-charge` supplies it
`-m, --multiplicity INT`	Spin multiplicity (2S+1).	`.gjf` template value or `1`
`--freeze-links / --no-freeze-links`	Freeze parents of cap hydrogens and merge with `geom.freeze_atoms`. Requires PDB/mmCIF topology (PDB/mmCIF input, or XYZ/GJF with `--ref-pdb`).	`True`
`--freeze-atoms TEXT`	Comma-separated 1-based atom indices to freeze explicitly (e.g., `'1,3,5'`); merged with `geom.freeze_atoms`.	`None`
`--max-write INT`	Number of modes to export.	`10`
`--amplitude-ang FLOAT`	Mode animation amplitude (Å).	`0.8`
`--n-frames INT`	Frames per mode animation.	`20`
`--sort CHOICE`	Mode ordering: `value` (cm^{-1}) or `abs`.	`value`
`-o, --out-dir TEXT`	Output directory.	`./result_freq/`
`--temperature FLOAT`	Thermochemistry temperature (K).	`298.15`
`--pressure FLOAT`	Thermochemistry pressure (atm).	`1.0`
`--dump / --no-dump`	Write `thermoanalysis.yaml`.	`False`
`--hessian-calc-mode CHOICE`	MLIP Hessian mode (`Analytical` or `FiniteDifference`).	`FiniteDifference`
`--convert-files / --no-convert-files`	Toggle XYZ/TRJ → PDB/mmCIF companions when a PDB/mmCIF template is available (GJF is not written).	`True`
`--ref-pdb PATH`	Reference PDB/mmCIF topology to use when the input is XYZ/GJF (keeps XYZ coordinates).	None
`--config FILE`	YAML overrides (sections: `geom`, `calc`, `freq`, `thermo`).	None
`--out-json / --no-out-json`	Write `result.json` summary to `--out-dir`.	`no-out-json`

When thermochemistry is requested, the molecular point group and external rotational symmetry number are detected automatically. The advanced YAML key `thermo.symmetry_number` can supply an explicit integer override.

Outputs

```
out_dir/ (default: ./result_freq/)
|- mode_XXXX_±freqcm-1_trj.xyz  # Per-mode animations (XYZ trajectory)
|- mode_XXXX_±freqcm-1.{pdb,cif} # Topology companions when a template exists and
   conversion is enabled
|- frequencies_cm-1.txt     # Full frequency list using the selected sort order
`- thermoanalysis.yaml      # Present when `thermoanalysis` is importable and --dump is
   True
```

- Console blocks summarizing resolved `geom`, `calc`, `freq`, and thermochemistry settings.

S2.16 dft

`dft` runs single-point DFT calculations using PySCF (CPU) or GPU4PySCF (GPU). The backend is controlled by `--engine`: `gpu` (default) requires GPU4PySCF; `cpu` forces PySCF. In addition to total energies, the command reports Mulliken, meta-Löwdin, and IAO atomic charges/spin densities.

Usage

```
pdb2reaction dft -i INPUT.{pdb|xyz|gjf|...} -q CHARGE [--ligand-charge <number|'RES:Q,...'
   >] [-m MULTIPLICITY] \
                 --func-basis 'FUNC/BASIS' \
                 [--max-cycle N] [--conv-tol Eh] [--grid-level L] \
                 [--out-dir DIR] [--engine gpu|cpu] \
                 [--config FILE]
```

CLI options

Table S15: Command-line options for `dft`.

Option	Description	Default
`-i, --input PATH`	Structure file in any format supported by the structure loader (e.g. PDB, mmCIF, XYZ, TRJ).	Required
`-q, --charge INT`	Total charge supplied to PySCF. Required unless the input is a `.gjf` template that already stores charge, or `--ligand-charge` supplies it (PDB/mmCIF input, or XYZ/GJF with `--ref-pdb`). Overrides `--ligand-charge` when both are set.	Required unless derivable
`-m, --multiplicity INT`	Spin multiplicity (2S+1). Converted to `2S` for PySCF.	`.gjf` template value or `1`
`--func-basis TEXT`	Functional/basis pair in `FUNC/BASIS` form (quote strings with `*`).	`wb97m-v/def2-tzvpd`
`--max-cycle INT`	Maximum SCF iterations (`dft.max_cycle`).	`100`
`--conv-tol FLOAT`	SCF convergence tolerance in Hartree (`dft.conv_tol`).	`1e-9`
`--grid-level INT`	PySCF numerical integration grid level (`dft.grid_level`).	`3`
`-o, --out-dir TEXT`	Output directory (`dft.out_dir`).	`./result_dft/`
`--engine [gpu|cpu]`	Backend policy: `gpu` uses GPU4PySCF (error if unavailable); `cpu` forces CPU PySCF.	`gpu`
`--lowmem / --no-lowmem`	Use `gpu4pyscf.dft.rks_lowmem.RKS` on closed-shell GPU runs (memory-efficient direct JK; mutually exclusive with density fitting). Open-shell, CPU, or pre-`rks_lowmem` GPU4PySCF installs auto-fall back to standard `RKS`/`UKS`.	`lowmem`
`--ref-pdb PATH`	Reference PDB/mmCIF topology to use when the input is XYZ/GJF (keeps XYZ coordinates).	None
`--config FILE`	YAML overrides (see below).	None
`--out-json / --no-out-json`	Write `result.json` summary to `--out-dir`.	`no-out-json`

Outputs

```
out_dir/ (default: ./result_dft/)
|- input_geometry.xyz   # Geometry snapshot sent to PySCF
|- result.yaml          # Energy/charge/spin summaries with convergence/engine metadata
`- result.json          # Machine-readable summary (when --out-json)
```

`result.yaml` contains:

- `energy`: Hartree / kcal mol^{-1} values, convergence flag, and engine metadata (`engine`, `used_gpu`, `used_lowmem`).
- `input`: configuration snapshot (charge, multiplicity, spin (2S), functional, basis set).
- `charges [index, element, mulliken, lowdin, iao]`: one flow-style row per atom giving the Mulliken, meta-Löwdin, and IAO atomic charges (`null` when a method fails). The key carries the column header; the machine-readable `result.json` exposes the same data under the short key `charges`.
- `spin_densities [index, element, mulliken, lowdin, iao]`: the corresponding per-atom spin densities (UKS-only for spins); short key `spin_densities` in `result.json`.

S2.17 bond-summary

`bond-summary` detects covalent bond changes between two or more structures by comparing pairwise interatomic distances against scaled covalent-radius thresholds; with $N \geq 2$ inputs it compares consecutive pairs (A→B, B→C, …). It reports formed and broken bonds with their interatomic distances.

Usage

```
pdb2reaction bond-summary -i reactant.xyz product.xyz
pdb2reaction bond-summary -i R.pdb P.pdb --device cuda
```

CLI options

Table S16: Command-line options for `bond-summary`.

Option	Description	Default
`-i, --input PATH...`	Two or more structure files to compare (same atom ordering required); repeatable `-i` and/or positional `[EXTRA_INPUTS]...`, compared as consecutive pairs.	Required
`--device TEXT`	PyTorch device for distance computation (`cpu` or `cuda`).	`cpu`
`--bond-factor FLOAT`	Covalent-radius scale factor used to decide whether a pair is bonded.	`1.20`
`--one-based / --zero-based`	Report atom indices in 1-based (`--one-based`) or 0-based (`--zero-based`) form (toggle pair, no argument).	`--one-based`
`--json / --no-json`	Emit JSON summary of formed/broken bonds to stdout (replaces text output).	`no-json`

Outputs

- A console report listing formed and broken bonds between the two structures, with atom indices and interatomic distances.
- No files are written; output is printed to stdout.

S2.18 trj2fig

`trj2fig` converts an XYZ trajectory from `pdb2reaction` into an energy figure. By default it reads the Hartree energies encoded in each frame's comment line, converts them to kcal/mol or Hartree, optionally references all values to a chosen frame, and exports the resulting series as static/interactive figures with html and CSV tables. When `-q/--charge` and/or `-m/--multiplicity` are provided, every frame's energy is recomputed with the backend selected by `-b/--backend` (`uma` by default) at the given charge/spin, instead of being read from the comment lines.

Usage

```
pdb2reaction trj2fig -i TRAJECTORY.xyz [-o OUTPUTS...] [-q CHARGE] [-m MULT] [options]
```

CLI options

Table S17: Command-line options for `trj2fig`.

Option	Description	Default
`-i, --input PATH`	XYZ trajectory whose second line stores energies.	Required
`-o, --out PATH`	Repeatable output filenames; supports `.png/.jpg/.jpeg/.html/.svg/.pdf/.csv`.	`energy.png`
extra arguments	Positional filenames listed after options; merged with the `-o` list.	None
`--unit {kcal,hartree}`	Target unit for the plotted/exported values.	`kcal`
`-r, --reference TEXT`	Reference specification (`init`, `None`, or 0-based integer).	`init`
`-q, --charge INT`	Total charge; triggers energy recomputation with the backend selected by `-b/--backend` when provided.	None
`-m, --multiplicity INT`	Spin multiplicity (2S+1); triggers energy recomputation with the backend selected by `-b/--backend` when provided.	None
`--reverse-x / --no-reverse-x`	Reverse the x-axis so the last frame appears on the left (and `init` becomes the last frame).	`False`
`-b, --backend CHOICE`	MLIP backend (`uma`, `orb`, `mace`, `aimnet2`) used when `-q/--charge` or `-m/--multiplicity` triggers energy recomputation.	`uma`
`--solvent TEXT`	Implicit solvent name for xTB correction during recomputation (e.g. `water`). `none` to disable.	`none`
`--solvent-model CHOICE`	xTB solvent model: `alpb` (analytical linearized Poisson–Boltzmann) or `cpcmx`.	`alpb`
`--out-json / --no-out-json`	Write `result.json` summary alongside the figures.	`no-out-json`

Outputs

```
<output>.[png|jpg|jpeg|html|svg|pdf]  # Plotly export for every requested extension (
    defaults to energy.png)
<output>.csv                          # Optional energy table when CSV is requested
result.json                           # Machine-readable summary (when --out-json)
```

- Console diagnostics describing parsing failures or unsupported extensions.

S2.19 `fix-altloc`

`fix-altloc` removes alternate-location (altLoc) records from PDB files by selecting one coherent non-blank label per residue. The label with the highest mean occupancy across that residue's labeled atoms is retained (ties are broken by earliest appearance), blank/shared atoms are kept, and coordinates from other labels are dropped. The altLoc column (PDB column 17) is blanked without reformatting the fixed-width record, and ANISOU records are propagated only for retained atom serials.

Usage

```
pdb2reaction fix-altloc -i INPUT.pdb [-o OUTPUT.pdb]
pdb2reaction fix-altloc -i ./dir -o ./dir_clean [--recursive]
pdb2reaction fix-altloc -i ./dir --inplace [--recursive]
```

CLI options

Table S18: Command-line options for `fix-altloc`.

Option	Description	Default
`-i, --input PATH`	Input PDB file or directory.	Required

Continued on next page

Option	Description	Default
`-o, --out PATH`	Output file (when input is a file) or output directory (when input is a directory). When omitted for a file, the suffix `_clean.pdb` is appended to the stem; when omitted for a directory, a sibling directory with the suffix `_clean` is created.	None
`--recursive/--no-recursive`	When input is a directory, process `*.pdb` files in all subdirectories.	`False`
`--inplace/--no-inplace`	Overwrite each input file in place; a `.bak` copy is saved next to the original before modification.	`False`
`--overwrite/--no-overwrite`	Allow overwriting existing output files.	`False`
`--force/--no-force`	Process files even when no altLoc character is detected; by default such files are skipped, and each skip is reported on the console.	`False`

Outputs

```
<input>_clean.pdb       # Default single-file output (out-of-place, no -o given)
<out_dir>/              # Default directory output (mirrors input tree structure)
<input>.pdb.bak         # Backup created when --inplace is used
```

- Console messages report the number of processed and skipped files.
- Atom serial numbers are not renumbered; gaps may remain after altLoc removal.
- CONECT and other non-coordinate records are passed through unchanged.

S2.20 `energy-diagram`

`energy-diagram` generates a state-energy diagram directly from a list of numeric energies, without requiring any structure file. Energies are passed via `-i/--input` as a sequence of plain numbers (`-i 0 12.5 4.3`), as a Python list literal (`-i "[0, 12.5, 4.3]"`), or as repeated `-i` flags.

Usage

```
pdb2reaction energy-diagram -i 0 12.5 4.3 -o energy.png
pdb2reaction energy-diagram -i "[-205.1, -190.4, -198.7]" --label-x R TS P
pdb2reaction energy-diagram -i 0 12.5 4.3 --label-x "['R','TS','P']" --label-y "ΔG (kcal/
    mol)"
```

CLI options

Table S19: Command-line options for `energy-diagram`.

Option	Description	Default
`-i, --input TEXT`	Numeric energy values. Accepts space-separated numbers, a Python list literal, or repeated `-i` flags. At least two values are required.	Required
`-o, --output PATH`	Output image path. The extension determines the format; `.png` is used when no extension is given. Supported: `.png`, `.jpg`, `.jpeg`, `.svg`, `.pdf`.	`energy_diagram.png`
`--label-x TEXT`	State labels for the x-axis. Accepts space-separated tokens, a Python list literal, or repeated flags. Must match the number of energy values when provided.	S1, S2, … (auto)
`--label-y TEXT`	Y-axis label.	`ΔE (kcal/mol)`
`--out-json / --no-out-json`	Write `result.json` sidecar alongside the diagram image.	`no-out-json`

Outputs

```
energy_diagram.png        # Energy diagram image (-o overrides; .png/.jpg/.svg/.pdf)
result.json               # Optional sidecar with status, point count (n_points), and
    output image path (--out-json)
```

■ S3. YAML CONFIGURATION REFERENCE

This section summarizes the YAML override mechanism used throughout `pdb2reaction`. A YAML file holds key/value overrides that are deep-merged on top of the built-in defaults at run time (scalar values and lists replace defaults, nested dictionaries are merged recursively). Precedence runs defaults < `--config` YAML < CLI, with one qualification that matters in practice: a CLI flag outranks the YAML only when it is given explicitly on the command line. A flag left unset keeps its parser default out of the way, so the YAML value is the one used.

S3.1 Common top-level blocks

The YAML root must be a mapping. Validation is section-specific: unsupported top-level `dmf` keys and invalid typed or ranged values in validated blocks (including `irc` and `freq`) raise an error, whereas some unknown keys in other blocks are ignored and leave the corresponding default in force. Check a new configuration against Table S20 and the online per-key reference before trusting the numbers it produces.

Table S20: Top-level YAML configuration blocks accepted by `pdb2reaction` and the subcommands that consume each block.

Block	Purpose	Used by subcommand(s)
`geom`	Geometry handling (coordinate type, frozen atoms).	all stages
`calc`	Calculator settings (UMA model, device, parallel predictor, Hessian mode).	all MLIP stages; direct `dft` additionally reads `calc.charge` and `calc.spin`
`opt`	Common optimization controls (threshold presets, cycle caps, logging).	`opt`, `tsopt`, `scan`, `scan2d`, `scan3d`, `path-opt`, `path-search`
`lbfgs`, `rfo`	Optimizer-specific knobs for the `grad` (L-BFGS) and `hess` (RFO) minimization opt-modes.	`opt`, `scan`, `scan2d`, `scan3d`, `path-opt`, `path-search`
`gs`	GSM controls (image count, growth settings, climbing refinement).	`path-opt`, `path-search`
`stopt`	String-optimizer controls for path optimization.	`path-opt`, `path-search`
`dmf`	DMF solver controls. Top level includes `backend`, `max_cycles`, `tol`, `ipopt_options`, `correlated`, `sequential`, `fbenm_only_endpoints`, and `k_fix`. The physics knobs live in three nested sub-blocks: `dmf_options` (β, `eps_vel`, `eps_rot`), `fbenm_options` and `cfbenm_options` (bond/δ scaling, the correlation parameter `eps`).	`path-opt`, `path-search` (`--mep-mode dmf`)
`search`	Recursive path-search controls (depth limit, RMSD thresholds, bridging).	`path-search`
`bond`	Covalent bond-change detection thresholds.	`scan`, `path-search`
`bias`	Harmonic restraint force constant for the scanned interatomic distance(s).	`scan`, `scan2d`, `scan3d`
`hessian_dimer`	Hessian-initialized dimer TS optimizer (`grad`/`dimer` mode).	`tsopt`
`rsirfo`	Hess-mode RFO-family TS optimizer settings (RS-P-RFO default; also RS-I-RFO / TRIM).	`tsopt`
`irc`	IRC integrator settings (step sizes, maximum steps, Hessian update policy).	`irc`
`freq`	Frequency analysis settings, including the shared resolved-mode cutoff.	`opt`, `tsopt`, `freq`
`thermo`	Thermochemistry conditions (temperature, pressure, dump).	`freq`
`dft`	PySCF single-point settings (method string, grids, convergence controls).	`dft`

S3.2 Example YAML configuration

The following skeleton illustrates a typical end-to-end configuration file covering every block in Table S20. Every block is shown. Many keys have no CLI flag, so `--help` is not a complete reference: the per-key defaults are those of `pdb2reaction/core/defaults.py`, documented key by key in the YAML reference page of the online documentation.

```yaml
# example_args.yaml
# This YAML can be used with: pdb2reaction all -i R.pdb P.pdb -c 'LIG' --config example_args.yaml

geom:
  coord_type: cart
  freeze_atoms: []          # 1-based indices (merged with link-H parent freezes)

calc:
  charge: 0                 # total system charge
  spin: 1                   # spin multiplicity (2S+1)
  model: uma-s-1p2          # UMA pretrained model
  task_name: omol           # UMA task tag
  device: auto              # auto|cpu|cuda
  workers: 1                # UMA predictor parallelism (>1 uses FiniteDifference; explicit Analytical is rejected)
  hessian_calc_mode: FiniteDifference  # FiniteDifference (default, bounded peak VRAM) or Analytical (exact second derivative; opt-in)
  return_partial_hessian: true   # calculator API switch; Hessian-consuming CLI workflows force the active-DOF block

opt:
  thresh: gau               # convergence preset: gau_loose, gau, gau_tight, gau_vtight, baker, never
  max_cycles: 100000
  print_every: 100
  dump: false
  energy_plateau: false         # opt-in stalled stop; CLI: --stop-plateau
  energy_plateau_thresh: 1.0e-4 # Hartree; ~0.06 kcal/mol
  energy_plateau_window: 50     # number of recent steps to check

lbfgs:
  max_step: 0.30
  keep_last: 7
  reject_uphill: false      # opt-in for minimum optimization

rfo:
  trust_radius: 0.10
  trust_max: 0.10
  trust_update: true
  reject_uphill: false      # opt-in for minimum optimization
  hessian_update: bfgs
  hessian_recalc: 500

gs:
  max_nodes: 20             # maximum string nodes (internal images)
  climb: true
  climb_lanczos: true

dmf:                          # DMF solver block
  dmf_options:                # nested: knobs forwarded to the pydmf inner solver
    beta: 10.0
    remove_rotation_and_translation: false
    eps_vel: 0.01
    eps_rot: 0.01

stopt:
  thresh: gau_loose            # string optimizer convergence preset
  max_cycles: 300
```

```yaml
  stop_in_when_full: 300
  align: false
  scale_step: global
  print_every: 10

search:
  max_depth: 10
  stitch_rmsd_thresh: 1.0e-04
  bridge_rmsd_thresh: 1.0e-04
  max_nodes_segment: 20
  max_nodes_bridge: 5

bond:
  bond_factor: 1.20
  margin_fraction: 0.05
  delta_fraction: 0.05

bias:
  k: 300                       # harmonic restraint force constant (eV/A^2)

hessian_dimer:
  thresh_loose: gau_loose
  thresh: baker
  update_interval_hessian: 500
  neg_freq_thresh_cm: 5.0
  flatten_amp_ang: 0.10
  flatten_max_iter: 0       # default off; CLI --flatten enables the built-in 50-iteration
    allowance
  dimer:
    length: 0.0189
    rotation_max_cycles: 15
    rotation_method: fourier
  lbfgs:
    thresh: baker
    max_step: 0.30
    keep_last: 7

rsirfo:
  thresh: baker
  trust_radius: 0.10
  trust_max: 0.10
  hessian_update: bofill
  hessian_recalc: 500
  max_micro_cycles: 50
  track_mode_by_overlap: false  # follow the most negative eigenvalue throughout (roots:
    [0]); true = re-identify the TS mode each cycle by overlap with the previous one

irc:
  step_length: 0.10          # unweighted Cartesian step length (Bohr)
  max_cycles: 125
  hessian_update: bofill
  root: 0

freq:
  zero_cutoff_cm: 5.0       # symmetric unresolved near-zero window; TS certification uses
    the same cutoff
  amplitude_ang: 0.8
  n_frames: 20
  max_write: 10

thermo:
  temperature: 298.15
  pressure_atm: 1.0
  symmetry_number: null     # automatic point-group detection; integer overrides
```

```
dft:
  func: wb97m-v              # exchange-correlation functional
  basis: def2-tzvpd          # basis set
  conv_tol: 1.0e-09
  max_cycle: 100
  grid_level: 3
  lowmem: true               # enable low-memory GPU code path
  engine: gpu                # gpu (GPU4PySCF) or cpu (PySCF)
```

S3.3 Stage-specific notes

Convergence presets.

See Section S4.4 for the preset semantics and tolerance values.

Cycle-limit defaults by subcommand.

Because the optimizers have different inner-loop costs, the cycle-limit flags have the subcommand-specific defaults in Table S21.

Table S21: Cycle-limit defaults per `pdb2reaction` subcommand.

Subcommand	Default	Controls
`opt` (`--max-cycles`)	`100000`	Single-structure optimizer cycles (`opt.max_cycles`)
`scan` (`--relax-max-cycles`)	`100000`	Relaxation cycles per biased step (`opt.max_cycles`)
`path-opt`/`path-search` (`--max-cycles-gsm`)	`300`	GSM string-optimizer cycles (`stopt.max_cycles`)
`path-opt`/`path-search` (`--max-cycles-dmf`)	`300`	DMF IPOPT iterations (`dmf.max_cycles`)
`tsopt` (`--max-cycles`)	`100000`	TS-optimizer macro cycles (`opt.max_cycles`)
`irc`	`125`	IRC integration steps (`irc.max_cycles`)
`all` (MEP cycle flags)	`300`/`300`	`--max-cycles-gsm`/`--max-cycles-dmf`; `--tsopt-max-cycles` independently defaults to `100000`

Under `all`, `--max-cycles-gsm` and `--max-cycles-dmf` set the MEP budgets for the selected driver. Each other stage takes its cap from its own flag—`--tsopt-max-cycles`, `--scan-relax-max-cycles`, or `--irc-max-cycles`—so the budgets do not compete; a stage whose flag is unset keeps its own default from Table S21.

Distance scan.

Scan targets are given only on the command line, as one `--scan-lists` literal per stage; there is no YAML key for them. The YAML supplies what the scan shares with the other stages—the restraint strength `bias.k` and the relaxation preset `opt.thresh`—and both are single run-wide values: one `bias.k` applies to every scanned distance.

DMF block nesting.

The `dmf` block holds the wrapper's own keys (including `backend`, `max_cycles`, `tol`, `ipopt_options`, `correlated`, `sequential`, `fbenm_only_endpoints`, and `k_fix`) together with three nested sub-blocks forwarded to the solver: `dmf_options` (`beta`, `eps_vel`, `eps_rot`, `remove_rotation_and_translation`), and `fbenm_options` / `cfbenm_options` (`bond_scale`, `delta_scale`, the correlation parameter `eps`). Override a solver knob at its nested path—`dmf.dmf_options.beta`, `dmf.cfbenm_options.eps`; a misplaced top-level key such as `dmf.beta` is rejected. DMF itself is selected with `--mep-mode dmf`, not from the YAML.

String-optimizer refinement.

`stopt.stop_in_when_full` is the number of optimizer cycles the string is allowed *after* it has grown to its full image count (`gs.max_nodes`). Note that `path-opt` and `path-search` set it equal to `stopt.max_cycles` immediately before the run, so a value written for it in the YAML has no effect: the refinement stage is shortened or lengthened through `stopt.max_cycles` (equivalently `--max-cycles-gsm`, default `300`). Lowering it leaves the images less converged; raising it helps when the path keeps drifting after full growth.

Thermochemistry conditions.

`thermo.pressure_atm` is the single pressure input consumed by the thermochemistry routine; the `freq --pressure` CLI flag and the `--freq-pressure` umbrella flag both write into the same key internally.

■ S4. IMPLEMENTATION DETAILS

S4.1 Units and conversions for MLIP energies and derivatives

MLIP-native eV / eV Å^{-1} / eV Å^{-2} outputs (energy, force, Hessian) are converted to Hartree / Hartree Bohr^{-1} / Hartree Bohr^{-2} internally so that the pysisyphus optimizer stack receives atomic units throughout.

S4.2 MLIP backend architecture

`pdb2reaction` uses a pluggable backend system for machine-learning interatomic potentials (MLIPs). All backends extend an abstract base class, `MLIPCalculator`, which inherits from `pysisyphus.calculators.Calculator` and implements the three standard calculator methods consumed by pysisyphus optimizers and MEP algorithms:

- `get_energy(elem, coords)` $\to \{E\}$
- `get_forces(elem, coords)` $\to \{E, \mathbf{F}\}$
- `get_hessian(elem, coords)` $\to \{E, \mathbf{F}, \mathbf{H}\}$

Coordinates are received from pysisyphus in Bohr and converted to Å at the boundary; returned energies, forces, and Hessians are converted to the atomic units described in Section S4.1. Freeze-atom handling (zero-force masking and active-DOF sub-block extraction for partial Hessians) is implemented once in the base class and shared by all backends.

Subclasses must implement a single hook:

$$\texttt{\_compute\_energy\_forces\_ev}(\text{elem}, \text{coord\_ang}) \longrightarrow (E\ [\text{eV}],\ \mathbf{F}\ [\text{eV Å}^{-1}])$$

and may optionally override `_compute_analytical_hessian_ev` to provide an analytical Hessian.

UMA (default backend).

The UMA backend wraps the `fairchem-core` ASE calculator and its associated `pretrained_mlip` API. Model loading is handled by `pretrained_mlip.get_predict_unit()`, which downloads and caches the requested checkpoint (default: `uma-s-1p2`). System charge and spin multiplicity are passed through the ASE `Atoms.info` dictionary to the underlying model. UMA supports both analytical (autograd) and finite-difference Hessian modes. In analytical mode, for the duration of the call, a small in-place utility (i) freezes parameter gradients (`requires_grad=False`) and (ii) switches the model to `train()` mode, because the default `eval()` mode cuts the computational graph that autograd needs to traverse for the second derivative; `torch.autograd.functional.hessian` then differentiates the energy with respect to all Cartesian coordinates, returning a GPU-resident $(N\times3\times N\times3)$ tensor that is symmetrized and converted to atomic units, and the original `requires_grad`/train–eval state is restored afterward. (Every `Dropout` module is disabled once at model load, so no per-call handling is needed.) The FD path is a central-difference scheme ($\epsilon = 10^{-3}$ Å) assembled on GPU via PyTorch tensors; when frozen atoms are present, only active-DOF columns are displaced and the final Hessian is either the full $(3N\times3N)$ matrix with frozen rows/columns zeroed, or the reduced active-DOF sub-block when partial-Hessian mode is enabled.

Multi-worker parallel inference.

For path optimization stages that evaluate multiple images per iteration, UMA supports multi-worker parallelism via the `fairchem-core ParallelMLIPPredictUnit` (backed by Ray). When `workers` $>$ `1`, inference requests are distributed across GPU workers and the default finite-difference Hessian path remains available; an explicit analytical-Hessian request requires `workers=1` and otherwise raises an error. All other (non-UMA) backends are single-worker.

Orb backend.

The Orb backend wraps the `orb-models` package and supports the Orb conservative model families (e.g., `orb_v3_conservative_omol`). Only conservative models are supported, because non-conservative models do not guarantee energy-consistent forces. Analytical Hessians are computed by calling `torch.autograd.functional.hessian` on a closure that builds a fresh Orb graph and runs `model.predict`, with the same in-place autograd-prep utility used for UMA applied for the duration of the call.

MACE backend.

The MACE backend wraps `mace-torch` and resolves model specifications through a dispatch mechanism that supports foundation model aliases (`mp:`, `off:`, `omol:`), local file paths, and URLs. The default model is `MACE-OMOL-0` (`extra_large`). Analytical Hessians use `mace-torch`'s internal `models[0](..., compute_hessian=True)` path so the Hessian remains a GPU tensor; non-standard MACE calculators use the public `calc.get_hessian(atoms=...)` API.

AIMNet2 backend.

The AIMNet2 backend wraps the `aimnet2calc` package (default model `aimnet2`). Unlike the other three backends, charge and spin multiplicity are passed as entries of the input dictionary handed to the model rather than through `atoms.info`. Its analytical Hessian is *native*: the calculator returns the second derivatives directly, already on the model device, so no double backpropagation is performed. AIMNet2 runs in single precision only—`--precision fp64` is rejected, because its model inputs are cast to `float32` upstream—and it is likewise incompatible with `--deterministic`, which its custom CUDA kernels do not support.

Hessian mode selection.

All backends accept a `--hessian-calc-mode` parameter with two values: `Analytical` and `FiniteDifference` (default). `Analytical` is available for the four built-in backends in single-worker mode—by double backpropagation for UMA, Orb and MACE, and from AIMNet2's native Hessian for AIMNet2—but not for a custom calculator supplied via `--calc-file`, which always takes the finite-difference path. For the FD path (Section S4.14), the UMA backend assembles the Hessian on GPU using PyTorch tensors, while all other backends use a NumPy-based CPU implementation in the base class.

S4.3 Custom-backend contract (`--calc-file`)

Beyond the built-in MLIP backends above, `--calc-file PATH` couples `pdb2reaction` to any ASE Calculator without modifying the toolkit, selecting the `custom` backend and overriding `-b/--backend`. The Python file must expose a factory (default name `get_calculator`, overridable via `--calc-file-func-name`) that returns an ASE Calculator instance, or a module-level Calculator instance under that name. The factory receives `charge`, `spin` (multiplicity; also accepted as `mult`/`multiplicity`), and `device` when its signature declares them, or the full keyword set if it declares `**kwargs`; per-frame charge and spin are additionally set on `atoms.info` on every call, matching the built-in backends. The returned Calculator must report energies in eV and forces in eV Å^{-1}, converted internally to atomic units as in Section S4.1. The finite-difference path needs only energies and forces—exactly the ASE Calculator contract—so Hessians for the `custom` backend are always built by finite differences (Section S4.14); frozen atoms are honored as usual.

```python
# my_calc.py (minimal illustrative example)
from ase.calculators.emt import EMT

def get_calculator(charge=0, spin=1, device="auto", **kwargs):
    return EMT()
```

Swapping `EMT()` for `tblite.ase.TBLite(...)`, a DFTB+ ASE calculator, or `ase.calculators.orca.ORCA(...)` couples the pipeline to GFN-xTB, DFTB+, or ORCA (the same engines named in the main-text Methods) without any other code change. The file is passed directly on the command line, e.g. `pdb2reaction sp -i model.xyz --calc-file my_calc.py -q 0 -m 1`; it is available for `sp`, `opt`, `tsopt`, `freq`, `irc`, `scan`/`scan2d`/`scan3d`, `path-opt`, `path-search`, and `all`.

S4.4 Convergence threshold presets

Table S22: Convergence threshold presets. All values are in atomic units (Hartree for energy, Hartree/Bohr for force, Bohr for displacement).

Preset	Max Force	RMS Force	Max Step	RMS Step
`gau_loose`	2.5×10^{-3}	1.7×10^{-3}	1.0×10^{-2}	6.7×10^{-3}
`gau`	4.5×10^{-4}	3.0×10^{-4}	1.8×10^{-3}	1.2×10^{-3}
`gau_tight`	1.5×10^{-5}	1.0×10^{-5}	6.0×10^{-5}	4.0×10^{-5}
`gau_vtight`	2.0×10^{-6}	1.0×10^{-6}	6.0×10^{-6}	4.0×10^{-6}
`baker`	3.0×10^{-4}	2.0×10^{-4}	3.0×10^{-4}	2.0×10^{-4}

The default preset is `gau` (Gaussian's default optimization tolerance). For the tighter-convergence stages—the tsopt stages invoked by `pdb2reaction all` (whether Dimer with `--opt-mode-post grad` or RS-P-RFO with `--opt-mode-post hess`) and the post-IRC endpoint re-optimization—the `baker` preset is used; convergence under `baker` requires all four Cartesian-force/step criteria above and an energy change $|\Delta E| < 10^{-6}$ Hartree between consecutive cycles. The auxiliary `never` preset disables early convergence altogether and lets the optimizer run until `max_cycles` is reached; it is intended for diagnostic runs and stages handled exclusively by a downstream refinement step.

S4.5 Active degrees of freedom and frozen atoms

Cluster-boundary atoms are typically frozen to emulate the mechanical confinement imposed by the surrounding protein environment omitted from the cluster model. `pdb2reaction` represents this as a boolean mask over atoms and derives an active

DOF list (Cartesian indices) that is used consistently across geometry optimization, IRC, Hessian evaluation, and frequency analysis:

- During optimization/MEP, frozen atoms are assigned zero forces and are excluded from step updates.
- During Hessian evaluation, the active-DOF sub-block is the default output (`return_partial_hessian: True`); a full Cartesian Hessian that is subsequently reduced to the active subspace is available as a fallback.
- In GPU tensor post-processing (frequency stage), projection and eigenanalysis are performed directly in the active subspace to avoid spurious near-zero modes caused by fixed boundaries.

S4.6 Cap-hydrogen record conventions

We use *cap hydrogen* for the monovalent atoms that terminate covalent bonds severed at the cluster boundary. The corresponding code and output identifiers use "link" (PDB atom name `HL`, residue name `LKH`, CLI flags `--add-linkh`/`--freeze-links`). When cap hydrogens are inserted at the cluster boundary (see the active-site extraction algorithm, Section S4.7), they are written as contiguous `HETATM` records and placed after an explicit `TER` line to separate them from the main protein/ligand block. The exact conventions are:

- Atom name: `HL`
- Residue name: `LKH`
- Chain identifier: `L` (default)
- Residue numbers: 1 through N (one pseudo-residue per cap hydrogen)
- Serial numbers continue from the last atom serial in the extracted (truncated) main block

Downstream parsing routines identify cap-hydrogen parent atoms by searching for the nearest non-cap atom to each `HL`/`LKH` record. When the `--freeze-links` option is enabled, these parent atoms are automatically added to the freeze mask.

S4.7 Active-site extraction algorithm (`extract`)

The `extract` subcommand builds a cluster model of the enzyme active site from a full protein–substrate complex in PDB or mmCIF format. Alternate locations are normalized residue by residue: one nonblank conformer label is selected by mean parsed occupancy (ties by first appearance), while blank/shared atoms are retained. The algorithm proceeds through residue selection, backbone truncation, cap-hydrogen insertion, and charge computation.

Substrate identification.

The center of the extraction is specified via the `-c`/`--center` option, which accepts three formats: (i) a PDB/mmCIF file containing only the substrate residues (a residue matches when its name, its atom count and all of its atom names agree and every atom lies within 10^{-3} Å of its counterpart), (ii) a comma-separated residue-ID list such as `A:123,456` (with optional chain and insertion code), or (iii) a residue-name list such as `GPP,SAM` (case-insensitive; all matching residues are included). All substrate atoms are unconditionally retained in the output.

Distance-based residue selection.

Pocket residues are selected using a Biopython `NeighborSearch` spatial index over all atoms in the complex. Two independent radial cutoffs are applied:

1. **Standard cutoff** (`-r`/`--radius`, default 2.6 Å, based on sums of van der Waals radii[1,2]): a non-substrate residue is included if any of its atoms lies within the cutoff of any substrate atom. When `--exclude-backbone` is enabled, amino-acid residues require a *side-chain* atom (not backbone N, C^{α}, C, O, OXT, or their hydrogens) to satisfy the distance criterion.
2. **Heteroatom–heteroatom cutoff** (`--radius-het2het`, default 0, i.e. disabled; set to e.g. 3.5 Å to enable): an additional inclusion pass restricted to non-C/H atoms on both the substrate and neighbor sides. This captures catalytically relevant polar contacts (e.g., metal–ligand coordination, hydrogen-bond networks) that may lie beyond the standard cutoff.

Water and ion inclusion.

Water molecules (residue names HOH, WAT, H2O, DOD, TIP, TIP3, SOL) within the cutoff radii are included by default (`--include-h2o`). Metal ions and common counterions are recognized from a built-in dictionary of $\sim$65 entries spanning common $+1$ to $+4$ cations (alkali, alkaline-earth, transition metals) and the halide series (F^- through I^-); they are included when they satisfy the distance criteria. Their formal charges are automatically assigned for the total charge computation (see below).

Augmentation rules.

After the initial distance-based selection, three augmentation passes extend the residue set:

- **Disulfide partners:** if either Cys of a disulfide pair (S_γ–$S_\gamma \leq 2.5$ Å) is selected, the partner is added automatically.
- **Backbone-contact neighbors:** when `--exclude-backbone` is *not* enabled and a non-substrate residue was selected via a backbone atom contact, the immediately adjacent N-side and C-side amino-acid residues are included, provided they are connected by a peptide bond (C–N ≤ 1.9 Å). This ensures that truncation of backbone stretches leaves chemically sensible fragments.
- **Proline N-side neighbor:** if a selected proline has a peptide-bond-adjacent predecessor, that predecessor is included to preserve the pyrrolidine ring context and the associated peptide carbonyl.

Backbone exclusion.

When `--exclude-backbone` is enabled, main-chain atoms (N, C^α, C, O, OXT, and their hydrogens H/HN, H1/H2/H3, HA/HA2/HA3) are removed from all non-substrate amino-acid residues, retaining only side-chain atoms. Proline and hydroxyproline are treated specially: N, C^α, H^α, and the N-terminal hydrogens (H, H1, H2, H3 when present) are preserved to maintain the cyclic side chain. For the N-side neighbor of a proline residue, the peptide carbonyl (C, O) and C^α are also preserved.

When `--exclude-backbone` is disabled (the default), a lighter truncation is applied: the N-terminal cap (N, H) of the first residue and the C-terminal cap (C, O, OXT) of the last residue in each peptide-bond-connected segment are removed, along with C^α/H^α for isolated single-residue segments. This truncation is TER-aware: peptide-bond adjacency (C–N ≤ 1.9 Å) is checked explicitly so that chain breaks introduced by TER records are respected.

Cap-hydrogen insertion.

When backbone truncation removes atoms that were bonded to retained atoms, cap hydrogens are inserted to terminate the resulting dangling bonds. The algorithm identifies severed bonds by checking whether a retained carbon atom (the "parent") had a bonded partner that was removed by truncation (the "partner"). The inserted cap hydrogen is attached to the parent atom on the retained side and points toward the deleted partner. The specific bond patterns checked are:

- C^β–C^α (side chain severed from backbone),
- C^α–N (N-terminal truncation), and
- C^α–C (C-terminal truncation).

For proline and hydroxyproline, only the C^α–C cut is checked. A cap hydrogen is placed along the direction from the parent carbon to the removed partner at a fixed C–H bond length of 1.09 Å:

$$\mathbf{r}_{\text{H}} = \mathbf{r}_{\text{parent}} + 1.09\ \text{Å} \times \frac{\mathbf{r}_{\text{partner}} - \mathbf{r}_{\text{parent}}}{|\mathbf{r}_{\text{partner}} - \mathbf{r}_{\text{parent}}|}. \tag{S1}$$

Only carbon parent atoms produce cap hydrogens; non-carbon parents are skipped. The resulting atoms are written using the cap-hydrogen record conventions in Section S4.6.

Charge computation.

The total charge of the extracted cluster is computed additively from per-residue formal charges:

$$Q_{\text{total}} = \sum_{i \in \text{amino acids}} q_i + \sum_{j \in \text{ions}} q_j + \sum_{k \in \text{ligands}} q_k. \tag{S2}$$

Standard amino-acid charges (including protonation variants such as HIP/HID/HIE, ASH/GLH, CYM, and Amber-style N/C-terminal patches) are read from a built-in dictionary of $\sim$141 residue names. Ion charges are assigned from a separate dictionary. For non-standard ligand residues (residue names not recognized as amino acids, ions, or water), the user supplies charges via `-l`/`--ligand-charge`, which accepts either a single total charge (distributed equally across unknown substrate residues, or across all unknown residues when none is a substrate residue) or a per-residue-name mapping (e.g., `GPP:-3,SAM:1`). The `--modified-residue NAME:charge` form registers or locally overrides a modified amino-acid residue's nominal charge; a residue without a unique built-in charge requires this explicit assignment. Waters contribute zero charge. In `all` extraction mode, explicit `-q` has highest priority; an explicit `-l` participates in extraction and takes precedence over YAML charge. When neither CLI charge form is supplied, YAML `calc.charge` overrides the automatically extracted total if present; otherwise the extracted total is propagated to downstream stages.

Frozen-atom encoding.

The `extract` output PDB does not itself encode frozen-atom information in B-factor columns. Instead, frozen atoms are determined at run time by downstream stages: when the `--freeze-links` option is enabled, a dedicated utility identifies the nearest non-cap atom to each HL/LKH cap hydrogen and adds those parent-atom indices to the freeze-atom list. Users may also specify additional frozen atoms explicitly via `--freeze-atoms`.

Single-structure (scan-list) vs. multi-structure (endpoint) input.

The cluster definition—residue set, cap-hydrogen positions, and total charge—is fixed at this extraction step and propagated to every downstream stage. The scan-list and endpoint modes of `pdb2reaction all` differ only in how the extractor is invoked. In scan-list mode the cluster is extracted from a single reactant PDB/mmCIF structure and reused unchanged for all scan stages and the subsequent MEP search. In endpoint mode the extractor is given two or more input structures (typically R and P) and selects pocket residues from each input independently, then unifies the per-input selections by an OR-union over residue keys: the disulfide, backbone-contact-neighbor, and N-side-proline augmentations are likewise applied per-structure and unioned. The resulting cluster therefore contains every residue that any input would have included on its own, which can yield a larger atom count and a different total charge than the scan-list extract from the same complex when the reactant and product substrate positions select different surrounding residues.

S4.8 Geometry optimization (`opt`)

Geometry optimization in `pdb2reaction` minimizes the MLIP potential energy of a cluster model with respect to Cartesian coordinates of unfrozen atoms. The optimizer backend is provided by the `pysisyphus` library, and two modes are available via the `--opt-mode` flag (Table S23).

Table S23: Optimizer selection under the shared `--opt-mode` flag. The default value is subcommand-dependent: `grad` under the `all` pipeline and for stand-alone scan / minimum stages, `hess` for stand-alone `tsopt`.

`--opt-mode`	minima	transition state
`grad`	L-BFGS	Hessian-initialized dimer
`hess`	RFO	RS-P-RFO

L-BFGS (gradient mode, `--opt-mode grad`).

The default optimizer is the limited-memory BFGS (L-BFGS) algorithm of Liu and Nocedal,[3] which approximates the inverse Hessian using the most recent m gradient–displacement pairs (default $m = 7$). At each step k, the search direction $\mathbf{p}_k = -\mathbf{H}_k^{-1}\mathbf{g}_k$ is obtained via the two-loop recursion on the stored pairs $\{(\mathbf{s}_i, \mathbf{y}_i)\}_{i=k-m}^{k-1}$, where $\mathbf{s}_i = \mathbf{x}_{i+1} - \mathbf{x}_i$ and $\mathbf{y}_i = \mathbf{g}_{i+1} - \mathbf{g}_i$. The step is controlled by a maximum Cartesian step norm $\|\mathbf{p}_k\|_\infty \leq 0.30$ Bohr (default). A double-damping scheme is enabled by default: when the curvature condition $\mathbf{s}_k^T\mathbf{y}_k > 0$ is violated, the gradient difference is regularized to maintain a positive-definite Hessian approximation. An optional line search along the accepted direction is also available (enabled by default).

RFO (Hessian mode, `--opt-mode hess`).

When `--opt-mode hess` is selected, the Rational Function Optimization (RFO) algorithm of Banerjee et al.[4] is used. At the beginning of the optimization and periodically every N_{recalc} cycles (default $N_{\text{recalc}} = 500$), the full Cartesian Hessian is computed (see Section S4.14). Between recalculations, the Hessian is updated using the BFGS rank-2 formula. The RFO step is obtained by solving the augmented eigenvalue problem

$$\begin{pmatrix} \mathbf{H} & \mathbf{g} \\ \mathbf{g}^T & 0 \end{pmatrix} \begin{pmatrix} \mathbf{p} \\ 1 \end{pmatrix} = \lambda \begin{pmatrix} \mathbf{p} \\ 1 \end{pmatrix}, \tag{S3}$$

and the step is accepted within a trust radius (default $\Delta = 0.10$ Bohr), which is adapted based on the agreement between predicted and actual energy changes. The trust radius is bounded between $\Delta_{\text{min}} = 10^{-4}$ and $\Delta_{\text{max}} = 0.10$ Bohr. GDIIS (geometry direct inversion in the iterative subspace) is enabled by default to accelerate near-convergence behavior.

Convergence.

The default convergence preset is `gau` (Table S22), requiring simultaneous satisfaction of maximum force, RMS force, maximum displacement, and RMS displacement thresholds.

Optional energy-plateau stop.

Force convergence thresholds can become difficult to reach when the MLIP force noise floor lies above the requested threshold. The `--stop-plateau` option (YAML `opt.energy_plateau: true`) provides an opt-in wasted-cycle safeguard: if the energy range (max − min) over the last W steps is below ϵ, the run stops with status `stalled`, not `converged`. The feature is off by default; $W = 50$ and $\epsilon = 1 \times 10^{-4}$ Hartree (≈ 0.06 kcal mol^{-1}) are the default tuning values when it is enabled. The check is disabled for chain-of-states optimizers, where per-image energy arrays make a simple range test ill-defined, and `max_cycles` remains the hard run bound.

Imaginary-mode flattening.

When `--flatten` is enabled, the optimizer enters a post-optimization loop that eliminates residual imaginary frequencies. At each iteration: (i) the full Hessian is recomputed, mass-weighted, and diagonalized; (ii) all modes with $\tilde{\nu} < -5$ cm^{-1} are identified; (iii) the geometry is displaced by $\pm\delta$ along each imaginary mode (in mass-weighted Cartesian coordinates, with

$\delta = 0.10$ Å scaled by $\sqrt{m_C/m_\alpha}$ per atom α); (iv) the lower-energy displacement is accepted; and (v) the optimizer is restarted from the displaced geometry. This cycle repeats (up to 50 iterations by default) until no imaginary modes remain.

S4.9 Distance-driven scan (`scan`, `scan2d`, `scan3d`)

The scan family of subcommands drives the system along one or more interatomic distance coordinates using harmonic bias potentials. At each grid point, the remaining degrees of freedom are relaxed while the scanned distances are restrained, producing a potential energy profile (1D) or surface (2D/3D).

Harmonic bias potential.

A harmonic restraint is applied to each scanned atom pair (i, j) with a target distance r_{target}:

$$V_{\text{bias}} = \sum_{p=1}^{N_{\text{pairs}}} \frac{1}{2} k \left(r_p - r_{p,\text{target}}\right)^2 , \tag{S4}$$

where $r_p = |\mathbf{r}_{i_p} - \mathbf{r}_{j_p}|$ is the instantaneous interatomic distance (computed in Bohr) and k is the force constant (default 300 eV Å^{-2}, converted to Hartree Bohr^{-2} internally). The bias forces on the two restrained atoms are

$$\mathbf{f}_{i_p} = -k \left(r_p - r_{p,\text{target}}\right) \hat{\mathbf{r}}_{ij} , \tag{S5}$$

$$\mathbf{f}_{j_p} = -\mathbf{f}_{i_p} , \tag{S6}$$

where $\hat{\mathbf{r}}_{ij}$ is the unit vector from atom j to atom i. The total potential seen by the optimizer is $V = V_{\text{MLIP}} + V_{\text{bias}}$, and the biased gradient is used during relaxation. In all three scan variants (`scan`, `scan2d`, `scan3d`) the unbiased MLIP energy V_{MLIP} is evaluated separately after convergence at each scan step or grid point, and that unbiased value is what is recorded in the trajectory and the result JSON.

1D staged scan (`scan`).

The 1D scan proceeds in sequential stages; each stage scans one or more atom pairs from their current distances to specified target distances. Within a stage, the scan is discretized into N steps of uniform size:

$$N = \left\lceil \frac{\max_p |r_{p,\text{target}} - r_{p,0}|}{\Delta r_{\max}} \right\rceil , \tag{S7}$$

where $r_{p,0}$ is the initial distance of pair p and $\Delta r_{\max}$ is the maximum step size (default 0.20 Å, user-configurable via `--max-step-size`). At step s ($s = 1, \ldots, N$), the restraint target for each pair is set to

$$r_{p,\text{target}}^{(s)} = r_{p,0} + s \times \frac{r_{p,\text{target}} - r_{p,0}}{N} , \tag{S8}$$

and the geometry is relaxed under the bias using either L-BFGS (`--opt-mode grad`) or RFO (`--opt-mode hess`). After all steps in a stage complete, an optional unbiased geometry relaxation (`--endopt`) is performed. Multiple inline `--scan-lists` arguments define consecutive stages, enabling multistep reaction coordinate driving.

Bidirectional scans.

When a scan target is specified as a 4-tuple $(i, j, r_{\text{start}}, r_{\text{end}})$, the code generates two stages automatically: the first drives the pair from the initial geometry to r_{start}, and the second resets the geometry to the pre-scan snapshot and drives to r_{end}. The final combined trajectory is assembled as $r_{\text{start}} \to r_0 \to r_{\text{end}}$ (the first pass reversed, then the second pass appended).

Pre-optimization.

When `--preopt` is enabled, an unbiased geometry optimization is performed on the input structure before the scan begins, providing a clean starting minimum and preventing initial bias-force artifacts.

2D grid scan (`scan2d`).

The 2D scan operates on two atom pairs, (i_1, j_1) and (i_2, j_2), with respective distance ranges $[r_1^{\min}, r_1^{\max}]$ and $[r_2^{\min}, r_2^{\max}]$. Each range is discretized into evenly spaced values:

$$d_k^{(n)} = d_k^{\min} + n \times \frac{d_k^{\max} - d_k^{\min}}{N_k} , \qquad n = 0, \ldots, N_k , \tag{S9}$$

where $N_k = \lceil |d_k^{\max} - d_k^{\min}| / \Delta r_{\max} \rceil$. The grid values are reordered so that points nearest the (pre-optimized) reference distances are visited first, improving convergence by starting relaxations from geometrically similar structures.

The scan proceeds in a nested loop: for each outer-loop value of d_1, the structure is first relaxed under a single-pair bias on (i_1, j_1), then an inner loop scans d_2 by setting biases on both pairs simultaneously and relaxing at each grid point. To further

improve convergence, a nearest-neighbor initialization strategy is employed: at each grid point (d_1, d_2), the starting geometry is chosen as the previously converged structure whose (d_1, d_2) values are closest (in Euclidean distance) to the current target.

After all grid points are evaluated, the unbiased energies are collected into a `surface.csv` file and converted to relative energies (kcal/mol) with respect to either the global minimum (`--baseline min`) or the first grid point (`--baseline first`). A radial basis function (RBF, multiquadric kernel) is used to interpolate the discrete grid onto a 50×50 mesh, and both a 2D contour plot (PNG) and an interactive 3D surface plot (HTML via Plotly) are generated.

3D grid scan (`scan3d`).

The 3D scan extends the 2D procedure to three atom pairs with a triply nested loop: d_1 (outer) $\rightarrow d_2$ (middle) $\rightarrow d_3$ (inner). The same nearest-neighbor initialization strategy is applied hierarchically: at each nesting level, the starting geometry is chosen from the cache of previously converged structures at that level. Grid values for each dimension are reordered by proximity to reference distances, analogous to the 2D case.

Energy data are recorded as a `surface.csv` with columns (d_1, d_2, d_3, E). For visualization, 3D RBF interpolation (multiquadric kernel) is performed on a $50 \times 50 \times 50$ grid, and isosurfaces at eight equally spaced energy levels are rendered as an interactive Plotly HTML file. The isosurfaces use graded opacity (innermost opaque, outermost transparent) to reveal the nested energy landscape structure.

Bond-change detection.

At the conclusion of each 1D scan stage, covalent bond changes between the initial and final structures are assessed. This uses the same bond-perception machinery described in Section S4.21 (the `pysisyphus` `COVALENT_RADII` table, scaled by a user-configurable tolerance factor α and a margin fraction m). Newly formed and broken bonds are reported in the stage summary, providing an automated check of whether the intended chemical transformation occurred along the scanned coordinate.

YAML/JSON scan specification.

In addition to inline Python-literal `--scan-lists` arguments, all scan subcommands accept a YAML or JSON file path that defines scan targets, stages, and per-pair options in a structured format. File-based specifications are detected automatically by checking whether the argument is an existing file path with a recognized extension.

S4.10 Growing String Method (`path-opt`)

The `path-opt` subcommand (with default `--mep-mode gsm`) constructs a minimum-energy-path (MEP) between two end-point structures using the growing string method (GSM).[5,6]

String representation.

The path is represented as a chain of N_{img} images (geometries) connected by an arclength parametrization. Each image carries its own coordinate set, calculator, and freeze mask. The normalized arclength density is defined as

$$u_i = \frac{\sum_{j=1}^{i} \|\mathbf{q}_j - \mathbf{q}_{j-1}\|}{\sum_{j=1}^{N_{\text{img}}-1} \|\mathbf{q}_j - \mathbf{q}_{j-1}\|}, \qquad u_0 = 0,\ u_{N_{\text{img}}-1} = 1, \tag{S10}$$

where $\mathbf{q}_j$ denotes the coordinate vector of image j. The desired equal spacing is the constant $s = 1/(N_{\text{max}} + 1)$, where N_{max} is the maximum number of internal (non-endpoint) nodes.

Growing phase.

The string starts with only the two endpoint images. Frontier nodes (left and right) are grown inward by stepping along the tangent connecting the frontier to its neighbor:

$$\mathbf{q}_{\text{new}} = \mathbf{q}_{\text{frontier}} + \frac{s}{|u_{\text{frontier}} - u_{\text{n}}|} \left(\mathbf{q}_{\text{n}} - \mathbf{q}_{\text{frontier}}\right), \tag{S11}$$

where $\mathbf{q}_{\text{n}}$ is the neighbor of the frontier node on the inward side (during growth this is the opposite frontier, since the two half-strings are still adjacent), u is the normalized arclength of eq. (S10), and s is the target spacing defined above. The displacement therefore points inward from either frontier without a separate sign convention, and the new node is inserted immediately inside the frontier it grew from. A new node is inserted when the perpendicular force on the frontier node drops below a threshold:

$$\|\mathbf{F}_{\perp}^{\text{frontier}}\|_{\text{rms}} \le \epsilon_{\perp} \qquad (\text{default } \epsilon_{\perp} = 5 \times 10^{-3} \text{ Hartree Bohr}^{-1}). \tag{S12}$$

In each cycle, each frontier that satisfies the perpendicular-force criterion independently spawns a new node, so one side, both sides, or neither may grow; growth continues until the string contains $N_{\text{max}} + 2$ images total (including endpoints).

Perpendicular forces.

At each optimization cycle, only the perpendicular component of the true force is retained for each image. The tangent $\hat{\boldsymbol{\tau}}_i$ at image i is the improved upwind tangent of Henkelman and Jónsson,[7] which selects the displacement vector toward the higher-energy neighboring image and switches to an energy-weighted blend of the two neighboring displacements when image i is a local energy extremum; for frontier nodes during the growing phase, a simple forward/backward difference is used. Once climbing begins, the tangent at the highest-energy image is instead taken as the lowest-curvature mode obtained by a Lanczos iteration (`climb_lanczos`, enabled by default). The perpendicular force is

$$\mathbf{F}_{\perp,i} = \mathbf{F}_i - (\mathbf{F}_i \cdot \hat{\boldsymbol{\tau}}_i)\,\hat{\boldsymbol{\tau}}_i. \tag{S13}$$

No spring forces are applied; the desired image distribution is maintained by periodic reparametrization.

Reparametrization.

Every n_{reparam} optimization cycles (default: every cycle during growing, every cycle after full growth), images are redistributed to achieve equal spacing on the normalized arclength. In Cartesian coordinates, cubic B-spline interpolation (`scipy.interpolate.splprep`) is used: the spline is evaluated at the desired parameter values $\{s \cdot i\}_{i=0}^{N_{\text{img}}-1}$ to obtain new image coordinates. Climbing images are excluded from redistribution.

Climbing image.

After the string is fully grown, the highest-energy image (HEI) is promoted to a climbing image.[7] For the climbing image, the force component along the tangent is inverted:

$$\mathbf{F}_{\text{CI}} = \mathbf{F} - 2\,(\mathbf{F} \cdot \hat{\boldsymbol{\tau}})\,\hat{\boldsymbol{\tau}}, \tag{S14}$$

driving it uphill along the tangent toward the saddle point, while relaxing perpendicularly.

Optimization.

The string is optimized using the `StringOptimizer`, which applies an L-BFGS step once history is available, falling back to a Fletcher–Reeves conjugate-gradient correction (with $\beta \leq 1$) during the early growing phase and to plain steepest descent on the very first cycle; the resulting step is scaled globally so that the largest component does not exceed `max_step`. Convergence follows the threshold presets described in Section S4.4 (default `gau_loose` for the string optimizer). The optimizer reports the HEI energy barrier $(E_{\text{HEI}} - E_0)$ in kcal mol^{-1} and the norm of the true (unprojected) forces at the HEI at each cycle.

S4.11 Direct-MaxFlux (`path-opt --mep-mode dmf`)

When `--mep-mode dmf` is specified, the MEP is constructed using the Direct-MaxFlux (DMF) variational method of Koda and Saito.[8–10] Unlike the GSM, which optimizes a string of images under perpendicular-force constraints, DMF minimizes a variational functional built from the energies alone. Because no energy *derivative* appears in the functional itself, no Hessian is required; the potential-energy gradients (the MLIP forces at each evaluation point) do enter the gradient of that functional through the chain rule, and are evaluated at every iteration.

Initial path via FB-ENM interpolation.

An initial guess path is generated using the Flat-Bottom Elastic Network Model (FB-ENM)[9] interpolation. The FB-ENM constructs a smooth path between reactant and product by minimizing an elastic network objective subject to flat-bottom distance constraints. Key parameters include:

- `delta_scale` (default 0.2): controls the width of the flat-bottom region,
- `bond_scale` (default 1.25): scales the bond-length reference for the elastic network, and
- `fix_planes`: preserves planarity of aromatic/conjugated groups.

When `correlated=True` (default), the correlated FB-ENM (cFB-ENM)[10] variant is used, which accounts for correlated bond rearrangements and produces more physically reasonable initial paths for multistep reactions. The `sequential=True` option (default) applies FB-ENM constraints stepwise for improved convergence.

DMF optimization.

The DMF stage is delegated to the `pydmf` package;[8] `pdb2reaction` supplies the FB-ENM path, the MLIP calculator, and the settings below. The functional minimized is the MaxFlux line integral

$$\tilde{I}[\mathbf{x}] \;=\; \beta^{-1} \ln \int_0^1 |\dot{\mathbf{x}}(t)|\; e^{\beta E(\mathbf{x}(t))}\,\mathrm{d}t, \tag{S15}$$

in which the inverse-temperature parameter β is what makes the maximum energy along the path differentiable: as $\beta \to \infty$, $\tilde{I}$ approaches the highest energy on the path, so minimizing it drives the path toward the lowest barrier. The default is $\beta = 10\,\text{eV}^{-1}$,

and the integral is discretized over the --max-nodes evaluation points with the energies referenced to the lower of the two endpoints.

The optimization variables are not the image Cartesians but the coefficients of a cubic B-spline representation of the path (four segments by default), with the two endpoint coefficient rows held fixed. The only constraints handed to Ipopt[11] are equal-spacing (velocity) conditions, which keep the squared distance between neighboring evaluation points within $1 \pm$ eps_vel (eps_vel $= 0.01$) of its mean. Ipopt is run to a tight dual-infeasibility tolerance, capped at dmf.max_cycles iterations (default 300). Each image along the path is assigned an MLIP calculator; for systems with frozen atoms, harmonic constraint potentials

$$V_{\text{fix}} = \frac{1}{2} k_{\text{fix}} \sum_{i \in \mathcal{F}} |\mathbf{r}_i - \mathbf{r}_i^{\text{ref}}|^2 \tag{S16}$$

are applied via SumCalculator, where $\mathcal{F}$ is the frozen-atom index set, $\mathbf{r}_i^{\text{ref}}$ are the reference positions, and k_{fix} is the force constant (default 300 eV Å^{-2}).

Energy evaluation.

After the DMF path converges, the optimized images are evaluated with the MLIP calculator to obtain single-point energies. The highest-energy image (HEI) is identified as the TS candidate using the same local-maximum selection strategy as GSM (preferring internal local maxima over endpoint maxima).

S4.12 Recursive path search (path-search)

Unlike path-opt, which produces a single MEP, path-search detects bond-change events along an initial MEP and recurses on each elementary segment, integrating them into a continuous trajectory annotated with per-segment bond-change histories.

Algorithm overview.

The recursive procedure BuildMultistepPath(A, B) operates as follows:

1. **Initial MEP**: Run a GSM or DMF calculation between endpoints A and B with a reduced number of nodes (max_nodes_segment, default 20).

2. **HEI identification**: Locate the highest-energy image (HEI) along the resulting path.

3. **Endpoint extraction**: Extract images flanking the HEI as candidate intermediate structures. In refine_mode=peak (default for GSM), the images at HEI$\pm$1 are selected; in refine_mode=minima (default for DMF), the nearest local energy minima on either side of the HEI are selected.

4. **Endpoint optimization**: Optimize the extracted structures via single-structure optimization (L-BFGS or RFO) to obtain relaxed intermediates End_L and End_R.

5. **Refinement**: If bond changes are detected between End_L and End_R, a refined MEP (GSM or DMF) is computed for the End_L–End_R segment.

6. **Recursive decomposition**: The bond topology of the original endpoints A and B is compared against End_L and End_R. If multiple bond-change events are detected (i.e., the reaction from A to End_L or from End_R to B involves further covalent rearrangements), the algorithm recurses on those sub-segments.

7. **Integration**: All sub-paths are integrated into a continuous trajectory. Duplicate images at segment interfaces are removed when the RMSD falls below a stitching threshold (default 10^{-4} a_0).

Bridge segments.

When stitching reveals a gap between consecutive segments (RMSD above the bridge threshold, default 10^{-4} a_0), a short bridge MEP is automatically inserted. Bridge segments use a reduced node count (max_nodes_bridge, default 5) and no climbing image, serving only to provide a smooth geometric connection.

Depth control.

The maximum recursion depth is controlled by max_depth (default 10). At the maximum depth, a final MEP segment is computed without further decomposition.

Multi-pair support.

When more than two input structures are provided, path-search processes consecutive pairs $(S_0, S_1), (S_1, S_2), \ldots$ independently, each producing its own recursive decomposition. The resulting paths are concatenated with bridge segments as needed, and a unified energy profile is constructed.

Output.

The final stitched path is written as a multi-frame trajectory (`mep_trj.xyz`). A structured JSON summary (`summary.json`) records per-segment barrier heights, reaction energies, bond-change reports, and the segment decomposition hierarchy. An energy-profile plot is also generated.

Table S24: Default values for the recursive path-search parameters.

YAML key	Default	Role
`max_nodes_segment`	20	Internal nodes per initial MEP segment.
`stitch_rmsd_thresh`	10^{-4} a_0	Threshold below which adjacent segment endpoints are deduplicated.
`bridge_rmsd_thresh`	10^{-4} a_0	Threshold above which a bridge MEP is inserted between segments.
`max_nodes_bridge`	5	Reduced node count for bridge MEPs (no climbing image).
`max_depth`	10	Maximum recursion depth before falling back to a single MEP segment.

S4.13 Transition-state optimization (`tsopt`)

`pdb2reaction` exposes two TS optimization workflows through the shared `--opt-mode` switch: `grad` (Hessian-initialized dimer) and `hess` (RS-P-RFO). The standalone `tsopt` command additionally accepts the underlying algorithms by name—`rsprfo`, `rsirfo`, `trim` and `dimer`—as explicit `--opt-mode` values. `trim` is the trust-region image minimization of Helgaker:[12] the eigenvalue and the gradient component of the followed root are sign-inverted to define an image surface, on which a level-shifted Newton step $\zeta_i = -\tilde{g}_i/(\lambda_i - \mu)$ is taken with μ chosen so that the step length equals the trust radius. The three Hessian eigensolvers are the built-in settings compared in Section S6.15, alongside the independent Sella implementation; the Hessian-initialized dimer is not part of that comparison.

Hessian-initialized dimer (gradient mode, `--opt-mode grad`).

The Hessian-initialized dimer method combines an exact Hessian computation with the dimer saddle-point search[13–16] to provide a gradient-only TS optimizer that periodically updates its search direction from Hessian information. The algorithm proceeds as follows:

1. **Initial direction:** An exact Hessian is computed at the starting geometry, mass-weighted, treated using the constrained rigid-null projection described in Section S4.17 (with PHVA when frozen atoms are present), and diagonalized. The eigenvector of the r-th most negative eigenvalue ($r = 0$ by default) defines the initial Dimer orientation $\hat{\mathbf{N}}$.

2. **Loose dimer loop:** An L-BFGS optimization is run using the Dimer calculator, which modifies the forces to follow the lowest curvature mode uphill while minimizing in all perpendicular directions. The Dimer calculator computes effective forces
$$\mathbf{F}_{\text{eff}} = \mathbf{F} - 2(\mathbf{F} \cdot \hat{\mathbf{N}})\hat{\mathbf{N}}, \tag{S17}$$
where $\hat{\mathbf{N}}$ is the current orientation vector and $\mathbf{F}$ is the true gradient-derived force. The orientation $\hat{\mathbf{N}}$ is refined at each step by Fourier-interpolated rotations within the Dimer calculator. The L-BFGS uses a loose convergence threshold (`gau_loose` by default). Because the effective Dimer force is nonconservative, polynomial line search and uphill-trial rejection are disabled for these loops. Every N_{update} steps (default 500), the exact Hessian is recomputed and the Dimer direction is refreshed from the updated lowest eigenvector.

3. **Normal dimer loop:** After loose convergence, the Hessian is recomputed and the Dimer orientation is updated. A second L-BFGS loop runs with the tighter `baker` convergence criterion.

4. **Flatten loop (optional):** When `--flatten` is enabled and the converged TS has more than one imaginary mode, extra imaginary modes are flattened using the same displacement procedure as for minimum optimization (Section S4.8), but preserving the primary TS mode selected by the `root` parameter. A separation guard is available to avoid interfering with the TS direction: when a non-zero `flatten_sep_cutoff` is set, only imaginary modes whose largest-displacement atoms lie beyond that distance from those of the primary mode are flattened. The cutoff defaults to 0, i.e. the guard is inactive and every extra imaginary mode is flattened. Between flatten displacements, the Dimer loop is restarted to reconverge. An optional Bofill update (the SR1/PSB combination defined below) can be applied to the active-DOF Hessian block across flatten displacements to reduce the number of exact Hessian recomputations.

Exact PHVA and saddle-order reporting are performed only after numerical convergence. A stalled or otherwise nonconverged Dimer run retains its final geometry for diagnosis but is not certified as a transition state.

Partitioned and image RFO (`--opt-mode hess` / `rsirfo`).

The default Hessian-mode TS optimizer (`--opt-mode hess`) is the restricted-step *partitioned* RFO (RS-P-RFO) of Banerjee et al.,[4] which locates the saddle point by maximizing along the selected reaction-coordinate mode(s) and minimizing along the remaining modes, solving separate augmented rational-function subproblems for the two subspaces. Its Hessian is diagonalized on the MLIP device (`torch.linalg.eigh`); the two RFO subproblems and the restricted-step scaling are then evaluated on the host from the resulting eigenpairs, whereas RS-I-RFO forms its image Hessian and takes its step on the device.

Concretely, the gradient is transformed into the eigensystem of the Hessian, $\mathbf{g}^{\mathrm{e}} = \mathbf{V}^{\mathsf{T}}\mathbf{g}$ (with $\mathbf{V}$ the matrix of Hessian eigenvectors), and the eigenvalues are partitioned into an uphill set R—by default the single most negative eigenvalue, tracked at a fixed index unless `track_mode_by_overlap` is enabled—and the downhill complement. Each subspace is then given its own α-scaled augmented RFO problem,

$$\begin{pmatrix} \mathrm{diag}(\boldsymbol{\lambda}_S)/\alpha & \mathbf{g}^{\mathrm{e}}_S/\alpha \\ (\mathbf{g}^{\mathrm{e}}_S)^{\mathsf{T}} & 0 \end{pmatrix} \begin{pmatrix} \mathbf{s}_S \\ 1 \end{pmatrix} = \nu_S \begin{pmatrix} \mathbf{s}_S \\ 1 \end{pmatrix}, \qquad S \in \{R,\ \bar{R}\}, \tag{S18}$$

of dimension $(|R| + 1)$ for the uphill block—2×2 for the single-root default, which is why only first-order saddle points are supported—and $(N_{\mathrm{act}} - |R| + 1)$ for the downhill block. The uphill step is taken from the eigenvector of the *largest* eigenvalue of its augmented matrix and the downhill step from the *smallest*, each normalized so that its last component is unity. Both are solved by an $O(N)$ secular-equation root search, falling back to an explicit diagonalization of the augmented matrix when that search fails. The two partial steps are not mixed: each is written into its own eigenvector components, $\mathbf{s}[R] = \mathbf{s}_R$ and $\mathbf{s}[\bar{R}] = \mathbf{s}_{\bar{R}}$, giving the full partitioned-RFO step. If $\|\mathbf{s}\|$ exceeds the trust radius, α is updated from the analytic derivative $\mathrm{d}\|\mathbf{s}\|^2/\mathrm{d}\alpha$ (summed over the two subspaces) and the pair of subproblems is re-solved, up to `max_micro_cycles` times; the accepted step is finally rotated back, $\mathbf{Vs}$, and scattered over the frozen degrees of freedom. Between exact recomputations (every `hessian_recalc` cycles) the Hessian is carried forward with the Bofill update defined below.

The alternative `--opt-mode rsirfo` selects a closely related restricted-step *image* RFO (RS-I-RFO). The RS-I-RFO algorithm combines the original RFO saddle-point formulation of Banerjee et al.[4] with the BFGS-style Hessian updaters of Anglada and Bofill[17] under the automatic restricted-step scheme of Besalú and Bofill,[18] giving a trust-radius TS optimizer that transforms the saddle-point search into a pure minimization on an *image* potential energy surface by reflecting the component of the Hessian (and, correspondingly, the gradient) along each "uphill" eigenvector. The active-DOF Cartesian Hessian is diagonalized directly to define the root-following eigensystem. After numerical convergence, saddle order is assessed separately by exact Cartesian PHVA using the constrained rigid-null treatment in Section S4.17. The root index is tracked by a fixed index (`track_mode_by_overlap = False` by default) rather than by per-step eigenvector overlap, so the default is `roots` = [0] (the single most negative eigenvalue).

Following the image-Hessian construction implemented in `pysisyphus.tsoptimizers.RSIRFOptimizer`, for each root $r \in R$ with associated eigenvector $\mathbf{v}_r$ of $\mathbf{H}$, a Householder-type reflection projector is built,

$$\mathbf{P} \;=\; \mathbf{I} \;-\; 2\sum_{r\in R} \mathbf{v}_r\,\mathbf{v}_r^{\mathsf{T}}\,, \tag{S19}$$

which flips the sign of any vector component lying in the span $\{\mathbf{v}_r\}_{r\in R}$ while leaving the orthogonal complement unchanged. The image Hessian and image gradient are then obtained by applying $\mathbf{P}$ on the left,

$$\tilde{\mathbf{H}} \;=\; \mathbf{P}\,\mathbf{H}\,, \qquad \tilde{\mathbf{g}} \;=\; \mathbf{P}\,\mathbf{g}\,. \tag{S20}$$

By construction, $\tilde{\mathbf{H}}$ shares the full spectrum of $\mathbf{H}$ except that every eigenvalue in R is sign-flipped: the negative curvature directions the optimizer must climb become effective positive curvatures on the image surface. The image Hessian is symmetric and is diagonalized with `torch.linalg.eigh`, or with `numpy.linalg.eigh` when the Hessian is kept as a NumPy array (`out_hess_torch=False`); by default it remains a torch tensor on either CPU or GPU. This yields image eigenpairs $(\tilde{h}_i, \tilde{\mathbf{u}}_i)$, and the gradient is projected into the same basis, $\tilde{g}_i = \tilde{\mathbf{u}}_i^{\mathsf{T}}\tilde{\mathbf{g}}$. The step is then computed by a *single* minimization-RFO solve on the image surface via the arrowhead secular equation of Section S4.24 (eq. (S63), with $\{h_i, \tilde{g}_i\} \to \{\tilde{h}_i, \tilde{g}_i\}$ and the root chosen to the left of the smallest pole, as appropriate for a minimization), rather than by separate closed-form 2×2 maximization/minimization subproblems. This single-minimization image-RFO formulation is what distinguishes RS-I-RFO from the partitioned rational-function optimizer (P-RFO), in which distinct 2×2 blocks are solved for the uphill and downhill modes.

The resulting step $\Delta\mathbf{x}$ is scaled to satisfy a trust-radius constraint ($\Delta_{\max} = 0.10$ Bohr by default); the restricted-step scaling follows Besalú and Bofill,[18] adapting the step-scaling factor α in eq. (S63) so that $\|\Delta\mathbf{x}\| \le \Delta_{\max}$, and the trust radius itself is updated between cycles based on the predicted-vs-actual energy ratio.

Between recalculations (default every 500 steps), the Hessian is updated using the Bofill formula,[19] which takes a convex combination of the symmetric rank-one (SR1, also known as the Murtagh–Sargent or "MS" update) and the Powell–symmetric–Broyden (PSB, also known as the Powell update) formulas. Following the implementation in `pysisyphus.optimizers.hessian_updates`, the mixing parameter is

$$\phi \;=\; \frac{(\mathbf{s}^{\mathsf{T}}\boldsymbol{\xi})^2}{(\mathbf{s}^{\mathsf{T}}\mathbf{s})\,(\boldsymbol{\xi}^{\mathsf{T}}\boldsymbol{\xi})}\,, \qquad \boldsymbol{\xi} \;=\; \Delta\mathbf{g} - \mathbf{H}\mathbf{s}\,, \tag{S21}$$

and the Hessian update is

$$\Delta\mathbf{H}^{\text{Bofill}} \;=\; \phi\,\Delta\mathbf{H}^{\text{SR1}} \;+\; (1-\phi)\,\Delta\mathbf{H}^{\text{PSB}}\,, \tag{S22}$$

with $\phi \in [0, 1]$. This update preserves any existing negative eigenvalue structure of the Hessian more faithfully than the pure BFGS update used in minimum optimization, because it retains the SR1 contribution (which, unlike BFGS, does not force positive definiteness) precisely in the directions where the residual $\boldsymbol{\xi}$ is well aligned with the step $\mathbf{s}$.

The flatten loop for RS-I-RFO follows the same logic as for the Hessian-initialized dimer: extra imaginary modes are displaced, and the RS-I-RFO optimizer is restarted from the displaced geometry until at most one imaginary mode remains.

S4.14 Finite-difference Hessian construction

The Hessian matrix $\mathbf{H}$ is constructed via central finite differences of the MLIP forces. For each active Cartesian degree of freedom k (i.e., each DOF not belonging to a frozen atom), the corresponding column of the Hessian is computed as

$$H_{jk} = -\frac{F_j(\mathbf{x} + \epsilon\,\hat{\mathbf{e}}_k) - F_j(\mathbf{x} - \epsilon\,\hat{\mathbf{e}}_k)}{2\epsilon}, \tag{S23}$$

where $F_j = -\partial E/\partial x_j$ is the j-th component of the *force* vector (the leading minus sign in eq. (S23) converts the force central-difference into the energy second derivative) and $\epsilon = 10^{-3}$ Å is the displacement step size (in the MLIP's native Å coordinate space). Frozen DOFs are never displaced; their rows and columns in the full $3N \times 3N$ Hessian remain zero.

After all columns are assembled, the Hessian is symmetrized:

$$\mathbf{H} \leftarrow \frac{1}{2}\left(\mathbf{H} + \mathbf{H}^T\right), \tag{S24}$$

and converted from eV Å^{-2} to Hartree Bohr^{-2}.

When the calculator is configured with `return_partial_hessian=True` (the default for all optimization and frequency workflows), the active-DOF sub-block is extracted after symmetrization:

$$\mathbf{H}_{\text{act}} = \mathbf{H}[\mathcal{A}, \mathcal{A}], \tag{S25}$$

where $\mathcal{A}$ denotes the set of active Cartesian DOF indices. This reduces the matrix dimension from $3N \times 3N$ to $3N_{\text{act}} \times 3N_{\text{act}}$, avoiding storage and diagonalization costs that scale with the number of frozen boundary atoms.

FD vs analytical autograd tradeoff.

The FD path and the analytical autograd path both compute the second derivative of the MLIP energy. The analytical autograd path returns the exact second derivative in one backward traversal, but must hold the full backward graph over all Cartesian DOFs simultaneously, so peak VRAM scales with the model graph size. The FD path launches $6\,N_{\text{act}}$ independent force evaluations (central differences on the active Cartesian DOFs) with bounded peak VRAM (a single energy/force call at a time). FD is therefore the production default; analytical autograd is recommended for small clusters where VRAM permits (`--hessian-calc-mode Analytical`, `workers=1`). The two paths also differ in where the Hessian is built (Section S4.2): analytical Hessians are returned as GPU-resident tensors by every backend that implements them, and the UMA FD path assembles the Hessian on GPU as well, so with the default backend the Hessian never leaves the MLIP device and the downstream IRC/PHVA linear algebra runs there (Section S4.24); the FD path of the remaining backends is assembled on the host with NumPy, and the same operations then follow the NumPy code path.

S4.15 Intrinsic reaction coordinate (`irc`)

The `irc` subcommand traces the intrinsic reaction coordinate (IRC)[20,21] from a transition state (TS) using the Euler predictor–corrector (EulerPC) algorithm of Hratchian, Frisch, and Schlegel,[22] an evolution of the earlier Hessian-based predictor–corrector scheme of Hratchian and Schlegel.[23] The integration is carried out in mass-weighted Cartesian coordinates

$$\tilde{x}_i = \sqrt{m_i}\,x_i, \tag{S26}$$

where m_i is the atomic mass associated with Cartesian coordinate x_i. Frozen atoms are excluded from the active subspace throughout: Hessians, gradients, and displacements are restricted to the active degree-of-freedom (DOF) indices, and steps are expanded back to $3N$ dimensions with zero entries for frozen DOFs.

Initial displacement.

At the TS, the Hessian is computed (analytically or by finite differences) and projected with the constrained rigid-null treatment of Section S4.17: only full-system rigid motions compatible with the frozen anchors are removed from the active subspace. Writing the resulting projector as $\mathbf{P}$,

$$\tilde{\mathbf{H}}_{\text{proj}} = \mathbf{P}\,\tilde{\mathbf{H}}\,\mathbf{P}, \tag{S27}$$

where $\tilde{\mathbf{H}}$ is the mass-weighted Hessian restricted to active DOFs. Diagonalization yields eigenvectors $\{\mathbf{v}_k\}$ and eigenvalues $\{\lambda_k\}$. The transition vector $\mathbf{v}_0$ (the mode with the most negative eigenvalue; selectable via `--root`) defines the initial displacement direction.

Two displacement modes are supported:

- **Length-based** (`displ=length`): $\Delta\tilde{\mathbf{x}}_{\pm} = \pm\, d_{\text{len}}\, \mathbf{v}_0$, where d_{len} is the user-specified displacement length (default 0.1 a_0, mass-weighted).

- **Energy-based** (`displ=energy`, default): the step length d_s is determined from a quadratic model of the potential along $\mathbf{v}_0$,

$$d_s = \sqrt{\frac{2\,\Delta E}{|\lambda_0|}}, \tag{S28}$$

  where ΔE is the desired energy lowering (default 10^{-3} E_h) and λ_0 is the eigenvalue of $\mathbf{v}_0$. To prevent excessively large steps when $|\lambda_0|$ is near zero (flat saddle points), the step length is clamped:

$$d_s \leftarrow \min(d_s,\, d_{\max}), \qquad d_{\max} = 3.0\ a_0\ \text{(mass-weighted)}. \tag{S29}$$

  Forward and backward initial displacements are then $\Delta\tilde{\mathbf{x}}_{+} = d_s\, \mathbf{v}_0$ and $\Delta\tilde{\mathbf{x}}_{-} = -d_s\, \mathbf{v}_0$, respectively.

Predictor step (Euler integration).

From the current point $\tilde{\mathbf{x}}_n$, a forward Euler integration traces the steepest-descent path in mass-weighted coordinates. A conversion factor f_{conv} relates mass-weighted and unweighted gradient norms to set an appropriate Euler step size:

$$f_{\text{conv}} = \frac{\|\mathbf{g}_{\text{umw}}\|}{\|\tilde{\mathbf{g}}\|}, \qquad \delta_{\text{Euler}} = \frac{\Delta s}{N_{\text{pred}}/f_{\text{conv}}}, \tag{S30}$$

where Δs is the target step length (default 0.10 a_0) and N_{pred} is the maximum number of predictor microsteps (default 500). Note that Δs here is the unweighted Cartesian arc length in Bohr (see `EulerPC.get_integration_length_func`), not the mass-weighted convention of Hratchian, Frisch, and Schlegel[22]. At each microstep the gradient is approximated via a second-order Taylor expansion using the current mass-weighted Hessian:

$$\tilde{\mathbf{g}}(\tilde{\mathbf{x}}_n + \delta\tilde{\mathbf{x}}) \approx \tilde{\mathbf{g}}_n + \tilde{\mathbf{H}}_n\, \delta\tilde{\mathbf{x}}. \tag{S31}$$

Integration proceeds until the accumulated unweighted path length reaches Δs.

Corrector step (modified Bulirsch–Stoer).

At the predicted geometry, the true energy and gradient are evaluated, and the Hessian is updated via a Bofill update.[19] A distance-weighted interpolation (DWI)[23] surface is constructed from the accumulated (coordinates, energy, gradient, Hessian) data. The corrector then re-integrates the path on this DWI surface using a modified Bulirsch–Stoer (mBS) algorithm with Richardson extrapolation. Starting from the same initial point $\tilde{\mathbf{x}}_n$, Euler integrations with 20×2^k substeps ($k = 0, 1, \ldots$) are performed, and the Richardson table

$$R_{k,j} = \frac{2^j\, R_{k,j-1} - R_{k-1,j-1}}{2^j - 1} \tag{S32}$$

is built until the RMS error $\|R_{k,k} - R_{k,k-1}\|_{\text{RMS}} \le 10^{-5}$. The converged entry $R_{k,k}$ gives the corrected mass-weighted coordinates.

Convergence criteria.

By default, each IRC direction (forward and backward) terminates when any of the following conditions is met:

- $\text{rms}(\mathbf{g}) \le \epsilon_{\text{grad}}$ after passing an inflection point (default $\epsilon_{\text{grad}} = 10^{-3}$ $E_h\, a_0^{-1}$),
- $|\Delta E| \le \epsilon_E$ between consecutive points (default $\epsilon_E = 10^{-6}$ E_h),
- the energy increases (indicating overshoot past a minimum), or
- the maximum number of steps is reached (default 125).

The opt-in `--never-stop` mode bypasses the physical gradient- and energy-based criteria above and traces to the cycle cap; numerical or integration failure and external interruption still stop the run. The forward trajectory is reversed so that the final combined IRC runs from the forward endpoint through the TS to the backward endpoint: the first frame of the combined trajectory is the forward endpoint, taken as the reactant side, and the last frame is the backward endpoint, taken as the product side. For each explicitly converged direction with a valid downhill departure, the Bofill-updated endpoint Hessian is cached to seed post-IRC RFO endpoint re-optimization; nonconverged and cycle-cap diagnostic branches are not promoted to this cache. The R/P frequency stages compute exact Hessians after endpoint optimization, whereas a compatible exact terminal TS Hessian may be reused for TS frequency analysis.

S4.16 Vibrational frequency analysis (`freq`)

The `freq` subcommand performs vibrational frequency analysis on a stationary point by constructing and diagonalizing the mass-weighted, translation/rotation-projected Hessian.

Hessian computation.

The Cartesian Hessian $\mathbf{H}$ (in Hartree Bohr^{-2}) is obtained from the MLIP calculator. Two evaluation strategies are supported: analytical second derivatives (when the backend provides them) and numerical finite differences of the gradient. The finite-difference mode is the default and is always available regardless of backend.

Mass-weighted Hessian.

Let $\mathbf{M}$ be the $3N \times 3N$ diagonal matrix of atomic masses (in amu), where each mass m_α appears three times (for x, y, z). The mass-weighted Hessian is

$$\mathbf{H}_{\text{mw}} = \mathbf{M}^{-1/2}\,\mathbf{H}\,\mathbf{M}^{-1/2}\,. \tag{S33}$$

In the implementation, mass weighting is performed in-place on the GPU tensor to minimize memory usage:

$$(H_{\text{mw}})_{ij} = \frac{H_{ij}}{\sqrt{m_{\lfloor i/3\rfloor}}\,\sqrt{m_{\lfloor j/3\rfloor}}}\,, \tag{S34}$$

where $\lfloor i/3 \rfloor$ is integer division and $m_{\lfloor i/3\rfloor}$ denotes the mass of the atom whose three Cartesian DOFs include index i.

Translation/rotation projection for an unfrozen molecule.

To eliminate spurious zero-frequency modes arising from overall translation and rotation (TR), a $3N \times 6$ basis $\mathbf{B}$ is constructed in mass-weighted coordinates. The three translational basis vectors are

$$\mathbf{T}_\alpha = \begin{pmatrix} \sqrt{m_1}\,\mathbf{e}_\alpha \\ \sqrt{m_2}\,\mathbf{e}_\alpha \\ \vdots \\ \sqrt{m_N}\,\mathbf{e}_\alpha \end{pmatrix}, \qquad \alpha = x, y, z\,, \tag{S35}$$

and the three rotational basis vectors are constructed from mass-weighted cross products of the atomic displacements from the center of mass with each Cartesian unit vector:

$$(\mathbf{R}_\alpha)_k = \sqrt{m_k}\,(\mathbf{r}_k - \mathbf{r}_{\text{COM}}) \times \mathbf{e}_\alpha\,, \qquad k = 1, \ldots, N\,. \tag{S36}$$

These six vectors are collected into $\mathbf{B} = [\mathbf{T}_x, \mathbf{T}_y, \mathbf{T}_z, \mathbf{R}_x, \mathbf{R}_y, \mathbf{R}_z]$ and orthonormalized via singular value decomposition (SVD). Singular values below a relative tolerance (10^{-12} times the largest) are discarded, yielding r retained columns $\mathbf{Q} \in \mathbb{R}^{3N\times r}$ that span the TR subspace. For linear molecules, $r = 5$.

The projector $\mathbf{P} = \mathbf{I} - \mathbf{Q}\mathbf{Q}^{\mathsf{T}}$ (idempotent and symmetric since $\mathbf{Q}^{\mathsf{T}}\mathbf{Q} = \mathbf{I}_r$) removes TR components, and the projected Hessian is

$$\tilde{\mathbf{H}}_{\text{mw}} \;=\; \mathbf{P}\,\mathbf{H}_{\text{mw}}\,\mathbf{P}\,, \tag{S37}$$

which is algebraically equivalent to the expanded "dual" form

$$\tilde{\mathbf{H}}_{\text{mw}} \;=\; \mathbf{H}_{\text{mw}} \;-\; \mathbf{Q}\mathbf{Q}^{\mathsf{T}}\mathbf{H}_{\text{mw}} \;-\; \mathbf{H}_{\text{mw}}\mathbf{Q}\mathbf{Q}^{\mathsf{T}} \;+\; \mathbf{Q}\mathbf{Q}^{\mathsf{T}}\mathbf{H}_{\text{mw}}\mathbf{Q}\mathbf{Q}^{\mathsf{T}}\,, \tag{S38}$$

obtained by substituting $\mathbf{P} = \mathbf{I} - \mathbf{Q}\mathbf{Q}^{\mathsf{T}}$ on both sides; eq. (S38) makes the individual TR contributions that are subtracted out explicit. After projection, explicit symmetrization $\tilde{\mathbf{H}}_{\text{mw}} \leftarrow \frac{1}{2}(\tilde{\mathbf{H}}_{\text{mw}} + \tilde{\mathbf{H}}^{\mathsf{T}}_{\text{mw}})$ is applied to eliminate numerical asymmetry introduced by floating-point arithmetic.

Eigenvalue decomposition and frequency conversion.

The projected mass-weighted Hessian is diagonalized:

$$\tilde{\mathbf{H}}_{\text{mw}}\,\mathbf{v}_i = \lambda_i\,\mathbf{v}_i\,. \tag{S39}$$

The eigenvalues are converted to vibrational wavenumbers (cm^{-1}):

$$\tilde{\nu}_i = \frac{1}{2\pi c}\,\sqrt{|\lambda_i|}\,\times\,\eta\,, \tag{S40}$$

where c is the speed of light and η is a composite unit-conversion factor that brings $\sqrt{|\lambda_i|}$ (which is evaluated with $\mathbf{H}$ in Hartree Bohr^{-2} and atomic masses in amu) onto an angular-frequency scale in s^{-1}, so that division by $2\pi c$ (with c in cm s^{-1}) yields a wavenumber in cm^{-1}. Numerically, $\eta = \sqrt{E_{\text{h}}/(a_0^2\,m_{\text{u}})} \approx 9.68\times10^{14}$ s^{-1}, where E_{h}, a_0, and m_{u} are the Hartree, Bohr radius, and atomic mass constant, taken from `ase.units` (CODATA values) together with the atomic-unit conversion factors of `pysisyphus.constants`. This yields the familiar conversion $\tilde{\nu}$ [cm^{-1}] $\approx 5140.49\,\sqrt{\lambda}$ for λ in Hartree Bohr^{-2} amu^{-1}. Negative eigenvalues correspond to imaginary frequencies and are reported with a negative sign by convention. After conversion, modes with $|\tilde{\nu}| \leq$ `freq.zero_cutoff_cm` (default 5.0 cm^{-1}) are classified as unresolved near-zero modes and omitted from the resolved spectrum.

Partial Hessian Vibrational Analysis (PHVA).

When frozen atoms are present, vibrational analysis is restricted to the active-atom subspace (PHVA).[24] The constrained rigid-mode treatment and back-embedding procedure are given in Section S4.17.

Normal mode output.

For each mode i, the mass-weighted eigenvector $\mathbf{v}_i$ is converted to a Cartesian displacement:

$$\mathbf{d}_i = \frac{\mathbf{M}^{-1/2}\,\mathbf{v}_i}{\|\mathbf{M}^{-1/2}\,\mathbf{v}_i\|}\,. \tag{S41}$$

Mode animations are written as multi-frame XYZ trajectories with sinusoidal displacement:

$$\mathbf{R}(t) = \mathbf{R}_0 + A\,\sin\!\left(\frac{2\pi t}{T}\right)\,\mathbf{d}_i\,, \tag{S42}$$

where A is the displacement amplitude (default 0.8 Å) and T is the number of frames (default 20). When a PDB/mmCIF template is available, topology-aware companion files are generated for visualization in molecular graphics software.

S4.17 Frozen atoms and partial Hessian vibrational analysis (PHVA)

Frozen-atom specification.

Frozen atoms are selected at run time from CLI/YAML input rather than encoded in the PDB record itself. The `--freeze-atoms` option (or `geom.freeze_atoms` YAML key) accepts an explicit list of 1-based atom indices, which are converted to 0-based indices internally, and `--freeze-links` (enabled by default) automatically adds the protein-side parent atoms of each cap hydrogen to the frozen set. The two sources are merged before optimization and are propagated consistently to MEP, TSOPT, IRC, and frequency stages.

Force zeroing.

During optimization and MEP calculations, forces on frozen atoms are explicitly set to zero before the step is computed. This ensures that frozen atoms do not move, regardless of the optimizer's internal coordinate representation.

PHVA (partial Hessian vibrational analysis).

Vibrational analysis is performed in the active-atom subspace (PHVA).[24] The active-DOF block $\mathbf{H}_{\text{act}}$ is mass-weighted and symmetrized before diagonalization. A full-system rigid-motion basis is then constrained to displacements that leave every frozen atom fixed, and only the surviving rigid null modes are projected from the active space; with three or more non-collinear frozen anchors the generic surviving rank is zero. The resulting eigenvectors are back-transformed from mass-weighted to Cartesian coordinates and embedded into the full $3N$-dimensional space with frozen-atom components set to zero.

S4.18 Thermochemistry formulas (RRHO and QRRHO)

The main manuscript reports free-energy barriers assembled from stationary points with vibrational corrections. This section gives the explicit rigid-rotor harmonic-oscillator (RRHO) expressions and the quasi-rigid-rotor harmonic-oscillator (QRRHO) low-frequency treatment.

Harmonic frequencies.

Before applying the near-zero cutoff, the candidate-mode counts are $3N-6$ for an unfrozen nonlinear molecule, $3N-5$ for an unfrozen linear molecule, and $3N_{\text{active}}-r$ in PHVA, where r is the rank of the surviving constrained rigid-null basis. The resolved PHVA spectrum contains $N_{\text{resolved}} = 3N_{\text{active}} - r - N_{\text{near-zero}}$ modes (with the analogous unfrozen count), where $N_{\text{near-zero}}$ is determined by the symmetric `freq.zero_cutoff_cm` criterion above. The thermochemical sums use the positive-frequency subset of this resolved spectrum, denoted below by N_{vib}. The zero-point energy is

$$\text{ZPE} = \frac{1}{2}\sum_{i=1}^{N_{\text{vib}}}\hbar\omega_i. \tag{S43}$$

RRHO vibrational contributions.

In the RRHO approximation, vibrational internal energy and entropy contributions are

$$U_{\text{vib}}(T) = \sum_i \left[\frac{\hbar\omega_i}{2} + \frac{\hbar\omega_i}{\exp(\hbar\omega_i/k_{\text{B}}T)-1}\right], \tag{S44}$$

$$S_{\text{vib}}(T) = k_{\text{B}}\sum_i \left[\frac{\hbar\omega_i/k_{\text{B}}T}{\exp(\hbar\omega_i/k_{\text{B}}T)-1} - \ln\!\left(1-e^{-\hbar\omega_i/k_{\text{B}}T}\right)\right]. \tag{S45}$$

Translational and rotational contributions are evaluated with ideal-gas partition functions using the molecular mass and principal moments of inertia, yielding $U_{\text{trans}}(T)$, $S_{\text{trans}}(T,p)$, $U_{\text{rot}}(T)$, and $S_{\text{rot}}(T)$.

Assembly of thermodynamic potentials.

The total free energy at (T, p) is assembled as

$$G(T,p) = E_{\text{elec}} + \text{ZPE} + \Delta H_{\text{therm}}(T,p) - T\,S_{\text{therm}}(T,p), \tag{S46}$$

where ΔH_{therm} is the thermal enthalpy correction relative to 0 K (i.e., excluding ZPE, which is added explicitly), $\Delta H_{\text{therm}}(T,p) = [U_{\text{vib}}(T) - \text{ZPE}] + U_{\text{trans}}(T) + U_{\text{rot}}(T) + RT$, and $S_{\text{therm}} = S_{\text{trans}} + S_{\text{rot}} + S_{\text{vib}} + S_{\text{el}}$. In the bundled single-state treatment, $S_{\text{el}} = k_{\text{B}} \ln m$ for spin multiplicity m. Thermochemical quantities are reported for an ideal-gas reference at the user-specified pressure p (default $p = 1$ atm in `pdb2reaction`, converted to 101325 Pa) and temperature T (default 298.15 K). No additional 1 M concentration correction is applied; when a 1 M reference is desired for solution-phase interpretation, an ideal-gas concentration correction may be added,

$$\Delta G_{\text{1M}\leftarrow\text{1atm}} = RT\,\ln\left(\frac{C^\circ RT}{p^\circ}\right), \tag{S47}$$

with $C^\circ = 1$ mol L^{-1} ($= 10^3$ mol m^{-3}) and $p^\circ = 1$ atm.

QRRHO low-frequency treatment.

Low-frequency vibrational modes can produce unreliable entropic contributions in cluster models (especially when boundary atoms are frozen). To reduce this sensitivity, `pdb2reaction` ships a bundled `thermoanalysis` backend[25] and uses its default quasi-rigid-rotor harmonic-oscillator (QRRHO) approximation (kind=`qrrho`) for vibrational entropies and related thermochemical quantities. The `thermoanalysis` defaults used in `pdb2reaction` are a rotor cutoff of $\tilde{\nu}_{\text{rot}} = 100$ cm^{-1} and a damping exponent $\alpha = 4$.

Damping weights.

For a mode with positive wavenumber $\tilde{\nu}_i$ (cm^{-1}), the Grimme QRRHO damping weight[26] is used,

$$w_i = \left[1 + \left(\frac{\tilde{\nu}_{\text{rot}}}{\tilde{\nu}_i}\right)^{\alpha}\right]^{-1}, \tag{S48}$$

so that $w_i \to 1$ for stiff modes ($\tilde{\nu}_i \gg \tilde{\nu}_{\text{rot}}$) and $w_i \to 0$ for very soft modes.

Entropy interpolation.

The QRRHO vibrational entropy is evaluated as a weighted sum,

$$S_{\text{vib}}^{\text{QRRHO}}(T) = \sum_i \left[w_i\, S_i^{\text{HO}}(T) + (1 - w_i)\; S_i^{\text{FR}}(T)\right], \tag{S49}$$

where $S_i^{\text{HO}}(T)$ is the standard HO entropy and $S_i^{\text{FR}}(T)$ is the free-rotor entropy for mode i. In `thermoanalysis`, the free-rotor entropy is computed from an effective moment of inertia $I_{i,\text{eff}}$ as

$$S_i^{\text{FR}}(T) = k_{\text{B}}\left[\frac{1}{2} + \ln\left(\left(\frac{8\pi^3 I_{i,\text{eff}} k_{\text{B}} T}{h^2}\right)^{1/2}\right)\right], \tag{S50}$$

$$I_{i,\text{eff}} = \frac{I_i B_{\text{av}}}{I_i + B_{\text{av}}}, \qquad I_i = \frac{h}{8\pi^2 \nu_i}, \tag{S51}$$

where all quantities are expressed in SI base units: ν_i is the (positive) ordinary frequency in s^{-1}, related to the harmonic angular frequency by $\nu_i = \omega_i/(2\pi)$, h is Planck's constant in J s, k_{B} is in J K^{-1}, T in K, and both I_i and $I_{i,\text{eff}}$ carry units of kg m^2 so that S_i^{FR} comes out in J K^{-1}. B_{av} is a limiting inertia parameter (also in kg m^2) that caps the free-rotor effective moment of inertia $I_{i,\text{eff}}$ as $\nu_i \to 0$, preventing the free-rotor entropy from diverging at vanishing wavenumber. Following Grimme,[26] we adopt the empirical constant $B_{\text{av}} = 10^{-44}$ kg m^2 (≈ 0.028 cm^{-1} rotational constant via $B = h/(8\pi^2 Ic)$) as a hardcoded default in the `thermoanalysis` backend.

QRRHO partition function.

In addition to modifying entropies, the backend mixes HO and hindered-rotor vibrational partition functions,

$$q_{\text{vib}}^{\text{QRRHO}} = \prod_i \left(q_{i,\text{HO}}^{w_i}\, q_{i,\text{HR}}^{1-w_i}\right), \tag{S52}$$

where $q_{i,\text{HO}}$ is the standard HO partition function. The hindered-rotor term $q_{i,\text{HR}}$ follows Grimme's construction[26] and requires the average molecular moment of inertia I_{mean}. `pdb2reaction` passes the Cartesian coordinates and atomic masses of the supplied atom block to the thermochemistry backend, so I_{mean} is always available: it is evaluated as the mean eigenvalue of the inertia tensor of that block, and the full Grimme HO/hindered-rotor interpolation is used with the default rotor cutoff of 100 cm^{-1}. Setting the rotor cutoff to zero degenerates $q_{i,\text{HR}}$ to unity, in which case the vibrational partition function reduces to the damped HO product $\prod_i q_{i,\text{HO}}^{w_i}$; the entropy damping in eq. (S49) is applied regardless.

`pdb2reaction` prints the thermochemistry temperature and pressure in the `freq` stage summary, and (when enabled) writes them to `thermoanalysis.yaml` (see Section S2.15).

S4.19 Implicit solvent correction (xTB ALPB)

`pdb2reaction` supports implicit solvation through a delta-correction scheme based on the GFN2-xTB semiempirical method[27,28] with the Analytical Linearized Poisson–Boltzmann (ALPB) solvation model.[29] The correction is computed as the difference between solvated and vacuum xTB calculations at a fixed geometry:

$$\Delta E_{\text{solv}} = E_{\text{xTB}}^{\text{solv}} - E_{\text{xTB}}^{\text{vac}}, \tag{S53}$$

$$\Delta \mathbf{F}_{\text{solv}} = \mathbf{F}_{\text{xTB}}^{\text{solv}} - \mathbf{F}_{\text{xTB}}^{\text{vac}}, \tag{S54}$$

$$\Delta \mathbf{H}_{\text{solv}} = \mathbf{H}_{\text{xTB}}^{\text{solv}} - \mathbf{H}_{\text{xTB}}^{\text{vac}}, \tag{S55}$$

and the corrected MLIP quantities are

$$E_{\text{corr}} = E_{\text{MLIP}} + \Delta E_{\text{solv}}, \tag{S56}$$

$$\mathbf{F}_{\text{corr}} = \mathbf{F}_{\text{MLIP}} + \Delta \mathbf{F}_{\text{solv}}, \tag{S57}$$

$$\mathbf{H}_{\text{corr}} = \mathbf{H}_{\text{MLIP}} + \Delta \mathbf{H}_{\text{solv}}. \tag{S58}$$

Subprocess execution and parallelism.

Each xTB evaluation is performed by invoking the `xtb` command-line executable (configurable via the `xtb_cmd` YAML config key; default `xtb`) in an isolated temporary directory. The solvated and vacuum calculations for a given property level (energy, gradient, or Hessian) are executed concurrently using a `ThreadPoolExecutor` with two workers, so that the wall-clock cost is approximately that of a single xTB evaluation. Parallelism for the xTB process itself is controlled via the `OMP_NUM_THREADS` environment variable, with automatic detection from scheduler variables (`PBS_NP`, `SLURM_CPUS_PER_TASK`, `NSLOTS`).

Both worker threads inherit the same `OMP_NUM_THREADS` value, so the pair costs roughly one xTB evaluation of wall time on a node with 2 × `OMP_NUM_THREADS` free cores; `OMP_NUM_THREADS` can also be set explicitly.

Output parsing and unit conversion.

xTB writes energies and gradients in atomic units (Hartree, Hartree Bohr^{-1}) and Hessians in Hartree Bohr^{-2}. Energy-only single points parse the energy from xTB's stdout (`TOTAL ENERGY` line); gradient runs parse energies and gradients from the `.engrad` file, and Hessian runs additionally parse the `hessian` file. All quantities are converted to MLIP-compatible units (eV, eV Å^{-1}, eV Å^{-2}) using the following factors:

$$E\ [\text{eV}] = E\ [\text{E}_\text{h}] \times 27.211386, \tag{S59}$$

$$\mathbf{F}\ [\text{eV}\,\text{Å}^{-1}] = -\nabla E\ [\text{E}_\text{h}\,\text{Bohr}^{-1}] \times \frac{27.211386}{0.529177}, \tag{S60}$$

$$\mathbf{H}\ [\text{eV}\,\text{Å}^{-2}] = \mathbf{H}\ [\text{E}_\text{h}\,\text{Bohr}^{-2}] \times \frac{27.211386}{0.529177^2}. \tag{S61}$$

The delta corrections in eV-based units are then converted back to atomic units when added to the pysisyphus-facing calculator outputs.

Solvent name normalization.

Solvent names are normalized to lowercase and matched against the xTB ALPB solvent library. The special values `none`, `vac`, and `vacuum` disable the correction entirely, in which case all calls pass through to the underlying MLIP calculator unchanged. The solvation model can be selected between ALPB (`--solvent-model alpb`, default) and CPCM-X (`--solvent-model cpcmx`). Freeze-atom handling for the Hessian correction is inherited from the base class.

S4.20 DFT single-point evaluation (`dft`)

The `dft` subcommand provides single-point DFT energy evaluation with population analysis on MLIP-optimized geometries at a higher level of theory. The implementation wraps GPU4PySCF[30,31] (GPU-accelerated) and PySCF[32,33] (CPU) through a unified interface.

Engine selection.

Two engine modes are available via `--engine`:

- `gpu` (default): uses GPU4PySCF exclusively; raises an error if the GPU backend is unavailable.
- `cpu`: uses PySCF on CPU unconditionally.

For restricted closed-shell systems (multiplicity $=$ 1), RKS is used; for open-shell systems (multiplicity $>$ 1), UKS is used. Density fitting (`density_fit()`) is applied except on the closed-shell GPU `lowmem` branch (`gpu4pyscf.dft.rks_lowmem.RKS`, default for closed-shell GPU runs), which uses a memory-efficient direct JK pipeline.

Functional and basis set.

The exchange–correlation functional and basis set are specified as a combined string via `--func-basis` (e.g., `wb97m-v/def2-tzvpd`). The defaults are ωB97M-V[34]/def2-TZVPD.[35] Both can be overridden individually through YAML configuration keys `dft.func` and `dft.basis`. When a def2-family basis is selected, the matching Stuttgart effective core potential is set automatically (`mol.ecp = basis`), so that the elements for which the def2 family defines an ECP—Rb and beyond ($Z \geq 37$)—use it rather than PySCF's all-electron default. Lighter elements, including the first-row transition metals of the benchmark set, are all-electron in def2 and are unaffected.

SCF parameters.

The following SCF parameters are exposed:

- `--conv-tol`: SCF energy convergence tolerance (default: 10^{-9} Hartree).
- `--max-cycle`: maximum number of SCF iterations (default: 100).
- `--grid-level`: numerical integration grid level (PySCF `grids.level`, default: 3).

Population analysis.

After SCF convergence, three population analysis schemes are evaluated to provide atomic partial charges and (for open-shell systems) spin densities:

1. **Mulliken**: Standard Mulliken population analysis (`pyscf.scf.hf.mulliken_pop`).
2. **meta-Löwdin**: Mulliken populations in the meta-Löwdin AO basis (`mulliken_pop_meta_lowdin_ao`), which provides better basis-set stability than standard Mulliken charges.
3. **IAO (Intrinsic Atomic Orbital)**: Charges and spin densities are computed in the IAO basis of Knizia.[36] IAO orbitals are constructed from the occupied MO coefficients using the `minao` minimal basis, and the density matrix is transformed to the IAO basis for Mulliken-style analysis.

For open-shell (UKS) calculations, spin densities M_s are computed for each scheme by analyzing the spin-density matrix $\mathbf{D}^\alpha - \mathbf{D}^\beta$.

Output.

The DFT subcommand writes `result.yaml` containing the total SCF energy (Hartree and kcal mol^{-1}), convergence status, engine label, per-atom charges, and per-atom spin densities. A copy of the input geometry is saved as `input_geometry.xyz` for reproducibility.

S4.21 Bond-Change Detection

Bond-change detection determines which covalent bonds are formed or broken between two molecular structures (e.g., reactant and product). A bond between atoms i and j is considered to exist when the interatomic distance falls below a threshold defined by element-specific covalent radii scaled by a tolerance factor α and reduced by a margin fraction m:

$$b_{ij} = \begin{cases} 1 & \text{if } d(i,j) \leq \alpha\,(1-m)\left(r_{\text{cov}}^{(i)} + r_{\text{cov}}^{(j)}\right), \\ 0 & \text{otherwise}, \end{cases} \tag{S62}$$

where $d(i,j)$ is the Euclidean distance between atoms i and j, $r_{\text{cov}}^{(i)}$ is the tabulated covalent radius of the element of atom i (from the `pysisyphus` covalent-radius table, based on Cordero et al. 2008[37]), α is a global scaling factor (`bond.bond_factor`, default $\alpha = 1.20$), and m is a conservative margin fraction (`bond.margin_fraction`, default $m = 0.05$) that shrinks the cutoff to avoid borderline matches.

Given two structures with identical atom ordering—structure 1 (e.g., reactant) and structure 2 (e.g., product)—bond adjacency matrices $\mathbf{A}^{(1)}$ and $\mathbf{A}^{(2)}$ are constructed independently using eq. (S62). Only upper-triangular entries ($i < j$) are evaluated to avoid double-counting. A bond change is registered for pair (i,j) when (i) the bond exists in one structure but not the other, and (ii) the absolute distance change exceeds a minimum threshold gated by a delta fraction δ (`bond.delta_fraction`, default $\delta = 0.05$):

$$|d^{(2)}(i,j) - d^{(1)}(i,j)| \;\geq\; \delta\,\alpha\left(r_{\text{cov}}^{(i)} + r_{\text{cov}}^{(j)}\right).$$

This delta gating prevents reporting spurious bond changes when both distances are close to the cutoff boundary. The specific bond-change categories are:

- **Bond formed**: $A_{ij}^{(1)} = 0$ and $A_{ij}^{(2)} = 1$.
- **Bond broken**: $A_{ij}^{(1)} = 1$ and $A_{ij}^{(2)} = 0$.

All pairwise distance matrices are computed via `torch.cdist` in double precision on GPU when available, with transparent fallback to CPU.

S4.22 End-to-end workflow orchestration (`all`)

The `pdb2reaction all` command automates the complete PDB-to-$\Delta G^{\ddagger}$ pipeline. Given one or more input structures, it orchestrates active-site extraction, optional distance-driven scans, minimum-energy-path (MEP) search, transition-state optimization, IRC verification, endpoint optimization, vibrational frequency analysis, and an optional DFT single-point calculation. The driver produces energy diagrams and structured output files without manual stage-by-stage invocation.

Input modes.

Three input modes are supported:

1. **Endpoint mode:** Two or more structures (PDB/mmCIF/XYZ/GJF) are supplied with `-i` in reaction order (reactant, intermediates, product). Each structure undergoes pocket extraction when `-c/--center` is provided with PDB/mmCIF input, and the resulting structures serve as endpoints for the MEP search.
2. **Scan-list mode:** A single structure is provided together with `-s/--scan-lists` specifications. Sequential distance-driven scan stages generate intermediate geometries that, combined with the (optionally pre-optimized) starting structure, define the ordered series of endpoints for the subsequent MEP search. When extraction is active, atom indices from the full PDB/mmCIF structure are automatically remapped to the pocket numbering.
3. **TS-only mode:** A single structure with `--tsopt` and no `--scan-lists` enters a direct TS optimization mode. No MEP search is performed; the input is treated as an initial TS guess and refined directly via the `tsopt` algorithm. IRC and requested later stages run only when the TS continuation gate described below is satisfied.

Stage sequencing.

For the standard multi-endpoint pipeline, stages execute in the following order:

1. **Preflight** — Structure normalization and residue-coherent alternate-location resolution are applied to PDB/mmCIF inputs when needed.
2. **Extract** (Stage 1) — Active-site pocket extraction via the `extract` subcommand with configurable radius, cap-hydrogen insertion, and charge derivation. Skipped when `-c/--center` is omitted, in which case the full input structures are used as-is.
3. **Scan** (Stage 1b, optional) — Distance-driven staged scan on the single extracted pocket. Each `--scan-lists` literal defines one stage with one or more $(i, j, d_{\text{target}})$ triples. Stage endpoints accumulate to form the ordered series for MEP search.
4. **Pre-optimization** (optional) — When `--preopt` is enabled (on by default), each pocket is geometry-optimized before MEP search.
5. **MEP search** (Stage 2) — By default, a single-pass `path-opt` (GSM or DMF, selected by `--mep-mode`) is run between each adjacent pair of endpoints and the resulting segments are concatenated. With `--refine-path`, the recursive `path-search` algorithm is used instead, which identifies all reactive segments via bond-change detection and refines each with the growing string method (GSM) or Direct-MaxFlux (DMF).
6. **Post-processing** (Stage 3) — Per-segment TS optimization first retains and classifies its result. IRC, endpoint optimization, frequency analysis, and DFT single-point evaluation proceed only when both the requested flags and the TS continuation gate allow them.

Conditional execution flags.

Three boolean flags control post-processing depth:

- `--tsopt`: Run TS optimization (Dimer or RS-P-RFO) on the highest-energy image of each reactive segment. After numerical convergence and completed terminal PHVA, IRC and endpoint re-optimization continue when at least one resolved imaginary mode is available. A converged higher-order saddle may continue through warning-labelled diagnostic IRC using a selected or fallback negative mode, without first-order-TS certification; nonconvergence, failed/unavailable terminal Hessian analysis, or no resolved imaginary mode stops the pipeline after preserving the TS artifacts.
- `--thermo`: Run vibrational frequency analysis (`freq`) on the R, TS, and P structures of each segment and construct ΔG diagrams using RRHO (or quasi-RRHO) thermochemistry at the specified temperature and pressure.
- `--dft`: Run GPU-accelerated DFT single-point calculations (default: ωB97M-V/def2-TZVPD via GPU4PySCF) on R, TS, and P structures. When combined with `--thermo`, a composite DFT//MLIP Gibbs free-energy diagram is also generated by adding the MLIP thermal corrections to the DFT electronic energies.

`--thermo` and `--dft` are independently optional after `--tsopt`, but both require `--tsopt`. Thus `--tsopt --dft` without `--thermo` produces electronic energy diagrams at both the MLIP and DFT levels but omits Gibbs corrections.

Parameter forwarding.

The `all` driver forwards user-specified options to downstream stages with a layered precedence: `defaults < --config < CLI`, i.e., built-in defaults are overridden by the YAML configuration file (`--config`), then by explicit CLI options. The same ordering applies uniformly across subcommands. Key forwarded parameters include:

- `-b/--backend`: MLIP backend (`uma`, `orb`, `mace`, `aimnet2`; or a custom ASE calculator via `--calc-file`), forwarded to all computational stages.
- `--solvent / --solvent-model`: Implicit solvent correction (xTB ALPB/CPCMX), forwarded consistently.
- `--opt-mode`: Optimizer for scan and other single-structure stages (`grad`=L-BFGS, `hess`=RFO); the MEP driver is selected independently with `--mep-mode`.
- `--opt-mode-post`: Override optimizer for post-MEP stages (TSOPT, endpoint optimization). When unset it inherits an explicitly given `--opt-mode`; if neither is given it falls back to `hess` (RS-P-RFO).
- `--thresh`: Convergence preset for scan relaxations and path endpoint/single-structure optimizations. `--thresh-post` controls TSOPT and post-IRC endpoint optimizations, while `--thresh-gsm` and `--thresh-dmf` control the two MEP drivers.
- `--config`: Base YAML file for fine-grained control of calculator, optimizer, and stage-specific settings.

Multi-segment handling.

When the MEP search identifies multiple reactive segments (i.e., multiple barrier-crossing events along the reaction coordinate), the post-processing loop visits them in pathway order. For each segment k reached by the loop: (i) the highest-energy image is extracted as the TS guess; (ii) when the TS continuation gate passes, IRC and endpoint optimization produce segment-specific R_k/TS_k/P_k structures; (iii) requested frequency analysis and DFT evaluation are performed on these three stationary points; (iv) per-segment energy diagrams are written. If a TS continuation gate fails, the pipeline records the stop after preserving that segment's TS artifacts and leaves later segments unprocessed. When every segment completes, a composite energy diagram spanning the full reaction coordinate is assembled by concatenating segment energies in pathway order. GPU memory is released between segments via `torch.cuda.empty_cache()`.

Output structure.

The output directory layout under `--out-dir` (default: `result_all/`) is organized as:

- **Top level** — `summary.json`, `summary.log`, the promoted MEP deliverables (`mep.pdb`/`mep.cif` as applicable and `mep_trj.xyz`), and `energy_diagram_*.png` (MLIP, ΔG MLIP, DFT, ΔG DFT//MLIP). The aligned `mep_w_ref.{pdb,cif}` inspection composite is optional and requires `--write-ref-merge`, recursive `--refine-path`, extraction via `--center`, and PDB/mmCIF inputs.
- `segments/seg_NN/` — Per-reactive-segment deliverables: canonical `reactant`/`ts`/`product` (`.pdb`/`.cif`/`.xyz`/`.gjf`, as applicable) plus the stage subdirectories `ts/`, `irc/`, `freq/` (R/TS/P), and `dft/` (R/TS/P), gated by `--tsopt`/`--thermo`/`--dft` and the TS continuation decision.
- `_work/` — Pipeline scratch (safe to `rm -rf`): `models/`, `scan/`, and the raw MEP-engine output `path_opt/` (`path_search/` with `--refine-path`). In TSOPT-only mode `_work/path_opt/` is absent and the deliverables live under `segments/seg_01/`.

S4.23 Output file formats

Each stage writes a combination of plain-text logs, structured JSON/YAML, and figure outputs (Table S25):

Table S25: Per-stage output file formats.

Artifact	Format	Stage(s)	Purpose
`result.xyz` (or `final_geometry.xyz`)	XYZ	`scan` (one per stage, under `stage_XX/`); `opt`/`tsopt` (`final_geometry.xyz`); `dft` (`input_geometry.xyz`); the `all` driver copies the canonical final geometry into `result_all/`.	Terminal retained geometry; read JSON/log status before treating a nonconverged TS geometry as a stationary point.
`grid/point_*.xyz`	XYZ	`scan2d`/`scan3d`	Converged geometry at one grid point, named `point_iNNN_jNNN` (plus `_kNNN` in 3D), where each `NNN` is the target distance in Å as a zero-padded hundredths tag: `point_i250_j180.xyz` is the 2.50/1.80 Å point. These drivers have no stages and write no `result.xyz`.
`result.pdb` / `result.cif` / `result.gjf`	PDB / mmCIF / GJF	emitted alongside `result.xyz` when a PDB/mmCIF or Gaussian `.gjf` template is available.	Topology-aware companion of `result.xyz` for visualization or electronic-structure refinement.
`*_trj.xyz`	multi-XYZ	`scan`/`scan2d`/`scan3d`, `opt`, `tsopt`, `path-opt`, `path-search`, `irc`, `freq` (per-mode animations)	Trajectory along the scanned, optimized, MEP, IRC, or vibrational-mode path.
`result.json`	JSON	Leaf commands when `--out-json` is enabled; written together with an identical `summary.json`.	Schema-2.0 result, status, provenance, and output paths (Section S2); `all` consumes leaf results to assemble backend-neutral segment records.
`summary.json`	JSON	`path-search` and `all` (with per-segment copies under `segments/seg_NN/`)	Energies, convergence status, file paths.
`summary.log`	text	`path-search` and `all`	Human-readable summary for quick inspection.
`thermoanalysis.yaml`	YAML	`freq` (`--dump`)	RRHO/QRRHO thermochemistry output.

The effective configuration after the `defaults` → YAML → CLI merge can be echoed to stdout via `--show-config`.

S4.24 GPU acceleration in pysisyphus

Performance-critical linear algebra operations in the pysisyphus optimizer, TS optimizer, and IRC modules have been ported to CUDA-enabled PyTorch. This keeps Hessian-based operations on the same GPU device as the MLIP, which is essential for the several-hundred-atom cluster models considered here. All GPU-accelerated code paths maintain a dual implementation: when the Hessian or gradient is received as a `torch.Tensor` (residing on GPU), GPU kernels are used; when a NumPy array is received, the original CPU code path is followed.

Cartesian coordinates by default.

The coordinate system is selected with `--coord-type` (`cart`, `redund`, `dlc`, `tric`; chain-of-states methods accept `cart` and `dlc`). Cartesian coordinates are the default, and every result reported here was obtained with them: the GPU-accelerated linear algebra of the bundled fork operates on Cartesian tensors, so the Hessian and each step construction stay resident on

the MLIP device, whereas the primitive-coordinate and B-matrix evaluations are NumPy routines on the host and add a host round-trip to every cycle.

Secular equation RFO solver.

The Rational Function Optimization (RFO) step requires solving an augmented eigenvalue problem (Section S4.8). A direct approach involves diagonalizing the $(N+1) \times (N+1)$ augmented Hessian matrix at $\mathcal{O}(N^3)$ cost, where N is the number of active degrees of freedom. Because the augmented Hessian has arrowhead structure (a diagonal block plus a single bordering row/column), its eigenvalue problem reduces to the secular equation

$$f(\mu) = \sum_{i=1}^{N} \frac{\tilde{g}_i^2}{\alpha\mu - h_i} - \mu = 0, \tag{S63}$$

where μ is the level-shift parameter (following Besalú and Bofill[18]), h_i are the Hessian eigenvalues, $\tilde{g}_i$ are the projected gradient components, and α is the RFO step-scaling factor (initialized at 1 and adapted in the restricted-step micro-cycles until the step norm satisfies the trust-radius constraint). This scalar equation is solved in $\mathcal{O}(N)$ time via Newton–Raphson iteration,

$$\mu^{(k+1)} = \mu^{(k)} - \frac{f(\mu^{(k)})}{f'(\mu^{(k)})}, \tag{S64}$$

with bisection safeguards and using the analytical derivative

$$f'(\mu) = -\alpha \sum_{i=1}^{N} \frac{\tilde{g}_i^2}{(\alpha\mu - h_i)^2} - 1. \tag{S65}$$

The initial iterate $\mu^{(0)}$ is bracketed to the left of h_1/α (the leftmost pole of f), ensuring that the relevant root lies to the left of the entire pole spectrum; a bisection fallback is taken whenever a Newton iterate would leave the current bracket. Convergence is declared when $|f(\mu)| < 10^{-12}$, typically requiring fewer than 10 iterations. The secular solver is attempted first at each trust-radius microcycle; if it fails (e.g., due to near-degenerate eigenvalues or bracketing failure), the full $\mathcal{O}(N^3)$ eigendecomposition of the augmented Hessian is used as a fallback.

GPU eigendecomposition.

The Hessian eigendecomposition $\mathbf{H} = \mathbf{U}\boldsymbol{\Lambda}\mathbf{U}^{\mathrm{T}}$, which is required at each optimization cycle to transform the gradient into the eigenvector basis, is performed on GPU via `torch.linalg.eigh`. For symmetric matrices, `eigh` exploits the divide-and-conquer algorithm on GPU, providing substantial speedups over CPU `numpy.linalg.eigh` for matrices with $N \gtrsim 500$. The resulting eigenvalues and eigenvectors remain on GPU, so subsequent operations (gradient projection, RFO step construction, back-transformation) proceed without device transfers.

GPU GDIIS.

The Geometry Direct Inversion of the Iterative Subspace (GDIIS) accelerator, which is enabled by default near convergence, stores the m most recent error vectors (coordinate–force residuals) and solves for interpolation coefficients that minimize the residual norm. On GPU, the overlap matrix $A_{ij} = \sum_k e_{ik}\, e_{jk}$ is assembled via `torch.einsum("ij,kj->ik", ...)` and the linear system $\mathbf{Ac} = \mathbf{1}$ is solved via `torch.linalg.solve`. The interpolated coordinate and force vectors are computed as GPU tensor reductions. For the GEDIIS variant (which incorporates energy and Hessian information), the inverse Hessian is computed on GPU via `torch.linalg.pinv` and the quadratic energy model terms $\mathbf{g}_k^{\mathrm{T}}\mathbf{H}^{-1}\mathbf{g}_k$ are evaluated via `torch.einsum("ki,ji,ki->k", ...)`, avoiding transfer of the full Hessian to CPU.

RS-I-RFO GPU operations.

The Restricted-Step Image RFO transition-state optimizer (Section S4.13) constructs the image Hessian $\mathbf{H}^* = \mathbf{PH}$ via a reflection projector $\mathbf{P} = \mathbf{I} - 2\mathbf{v}_r\mathbf{v}_r^{\mathrm{T}}$, where $\mathbf{v}_r$ is the r-th Hessian eigenvector (the TS mode). On GPU, the outer product $\mathbf{v}_r\mathbf{v}_r^{\mathrm{T}}$ is computed via `torch.outer`, the matrix–matrix product $\mathbf{PH}$ via standard tensor contraction, and the eigendecomposition of $\mathbf{H}^*$ via `torch.linalg.eigh`. The projected gradient $\mathbf{g}^* = \mathbf{Pg}$ and the subsequent restricted-step RFO solve (including the secular equation solver) all operate on GPU tensors. The final step vector is transferred to CPU only at the end of the optimization cycle for coordinate update.

IRC GPU operations.

The intrinsic reaction coordinate integrator (Section S4.15) performs several linear algebra operations on the mass-weighted Hessian restricted to active degrees of freedom. The mass weighting $\tilde{\mathbf{H}} = \mathbf{M}^{-1/2}\mathbf{H}_{\mathrm{act}}\mathbf{M}^{-1/2}$, the Eckart projection $\tilde{\mathbf{H}}_{\mathrm{proj}} = \mathbf{P}\tilde{\mathbf{H}}\mathbf{P}$, and the subsequent eigendecomposition for initial displacement and Hessian updates are all performed on GPU when the calculator returns a `torch.Tensor` Hessian. The mass-weighting diagonal matrix $\mathbf{M}^{-1/2}$ is lazily promoted to a GPU tensor on first use. Eigenvectors are back-transformed to Cartesian displacements on GPU before expansion to the full $3N$-dimensional space via `torch.Tensor.index_copy_`.

S4.25 Multi-GPU and HPC scaling

MLIP inference parallelism.

MLIP inference is the dominant cost for many workflows (MEP refinement, repeated local optimizations during scans, and Hessian-based stages). `pdb2reaction` exposes MLIP parallelism via two calculator keywords: `workers` (total number of predictor workers) and `workers_per_node` (workers placed on each node), supplied through the CLI (`--workers`, `--workers-per-node`) or YAML (`calc.workers`, `calc.workers_per_node`). FD Hessians (the default at every stage) parallelize across workers; analytical autograd Hessians require `workers=1`.

Multi-GPU and multi-node execution.

On a single node with k visible GPUs (`CUDA_VISIBLE_DEVICES`), set `--workers` k `--workers-per-node` k. For multi-node execution, `pdb2reaction` distributes predictor workers across a Ray cluster: launch `ray start --head` on the first node, `ray start --address=...` on the remaining nodes, export `RAY_ADDRESS`, and invoke `pdb2reaction` with `--workers` equal to the total worker count and `--workers-per-node` equal to the per-node GPU count. Concrete bootstrap (PBS / SLURM module loads, NCCL/MPI networking, port selection, teardown) is scheduler- and site-specific; representative scripts are maintained in the online documentation.

Batch throughput vs. single-run latency.

Multi-worker MLIP inference benefits workflows that evaluate energies and forces many times (scanning, GSM image optimization, DMF path refinement). Workflows dominated by independent energy/force evaluations (GSM/DMF, FD-Hessian stages) admit the largest speedups, while sequential single-structure optimizations show diminishing returns as worker count grows.

S4.26 Limitations and required user judgment

The following three chemistry-level decisions are not automated and must be handled by the user before trusting the resulting barrier heights or mechanism assignments:

- **Long-range electrostatics.** Cluster models do not capture long-range protein electrostatics.[38–40] Verify convergence by comparing at least two pocket radii. An optional xTB[27,28] ALPB[29] implicit-solvent delta correction is available (Section S4.19); it does not compensate for explicit environmental preorganization absent from the cluster model.
- **Ligand charge, protonation state, and spin multiplicity.** Ligand charges (supplied via `-l`/`--ligand-charge`), protonation variants of titratable residues (read from PDB residue names such as HIP/HID/HIE, ASH/GLH, CYM), and spin multiplicities for open-shell metal centers must be specified by the user. Incorrect protonation or spin can shift barriers by several to tens of kcal mol^{-1} or alter the predicted mechanism. Preprocess with pK_a prediction tools (e.g., PROPKA[41], H++[42]) and manually assign spin states for radicals and transition-metal complexes before mechanistic interpretation.
- **Conformational sampling.** The pipeline does not perform conformational sampling, so results may depend on the chosen crystal conformation. IRC connectivity confirms only that the optimized TS joins the intended minima; it does *not* guarantee that the identified path is the lowest-energy route on the conformational landscape.

■ S5. APPLICATION CASE STUDIES

The two case-study reactions are catalyzed by S-adenosyl-L-methionine (SAM)-dependent methyltransferases: catechol *O*-methyltransferase (COMT) and the geranyl pyrophosphate (GPP) C6-methyltransferase BezA. Scheme S1 summarizes both mechanisms; the atom-resolved active-site structural evolution along the IRC-validated paths is shown in Figure S1.

BezA residue numbering.

The catalytic glutamate is referred to throughout the main text as Glu170, following the literature numbering of Tsutsumi et al.[43] The corresponding residue in the input PDB shipped with this Supporting Information is GLU 186 (PDB numbering = literature numbering + 16); both labels denote the same residue.

COMT cluster charges.

For the COMT calculations the CAT/SAM/MG residue charges supplied via `-l` are `CAT:-1, SAM:0, MG:2`; the reactive catechol oxygen is deprotonated (catecholate, -1) in the input geometry. The scan-list and endpoint modes both yield a 670-atom cluster with total charge -2, because the reactant and product PDBs originate from the same QM/MM optimization and the multi-structure extractor (Section S4.7) finds the same residue set from each input.

BezA cluster charges.

For the BezA calculations the SAM/GPP residue charges supplied via `-l` are `SAM:1, GPP:-3` (Mg^{2+} inferred from residue type, Glu170 deprotonated). The quantitative barriers reported for BezA (main text and Table S29) use the 669-atom, net-charge-0 cluster for *both* input modes, so that the scan-list/endpoint comparison is isolated from cluster-model differences; this cluster is fed to the scan-list run as a pre-extracted `active_site_cluster_R.pdb` rather than obtained from the native

single-structure extraction. The native scan-list-mode extraction (main-text Figure 2D–F, shown for illustration only) instead traces a smaller 632-atom, net-charge-$+1$ boundary; the larger atom count and shifted total charge of the 669-atom cluster follow from the per-input residue selection and OR-union behavior of the multi-structure extractor (see “Single-structure (scan-list) vs. multi-structure (endpoint) input” in Section S4.7).

Table S26: Per-mode active-site cluster sizes, total charges, spin multiplicities, and frozen-boundary atom counts for the two case studies (main-text Figure 2). Atom counts include cap hydrogens. Both systems are treated as closed-shell singlets (multiplicity 1, the default; no `-m` is passed in the archived commands). The frozen set follows the default `--freeze-links` rule (Section S4.6): the parent atoms of the cap hydrogens are frozen—18 of them for the COMT cluster (18 cap hydrogens) and 26 for the BezA cluster (26 cap hydrogens)—while the cap hydrogens themselves and all remaining atoms stay active.

Case study	Input mode	Cluster size (atoms)	Total charge	Multiplicity	Frozen atoms
COMT	scan-list	670	-2	1	18
COMT	endpoint	670	-2	1	18
BezA	scan-list*	669	0	1	26
BezA	endpoint	669	0	1	26

* Fed as the pre-extracted 669-atom endpoint-mode cluster (`active_site_cluster_R.pdb`), not obtained from native scan-list extraction. The native single-structure scan-list extraction traces a smaller 632-atom, net-charge-$+1$ cluster (shown in main-text Figure 2D–F for illustration of the extraction pipeline) and was not used for any reported barrier.

BezA segment indexing.

Segment indices are assigned sequentially (1-based) along the stitched final MEP and count the non-reactive bridge segments as well, whereas per-segment deliverables are archived only for the reactive segments. For BezA the intervening segment is a bridge, so the two archived reactive segments are labeled `seg_01` and `seg_03` rather than 01/02; both correspond to the chemically reactive elementary steps (methyl transfer and C6–H deprotonation).

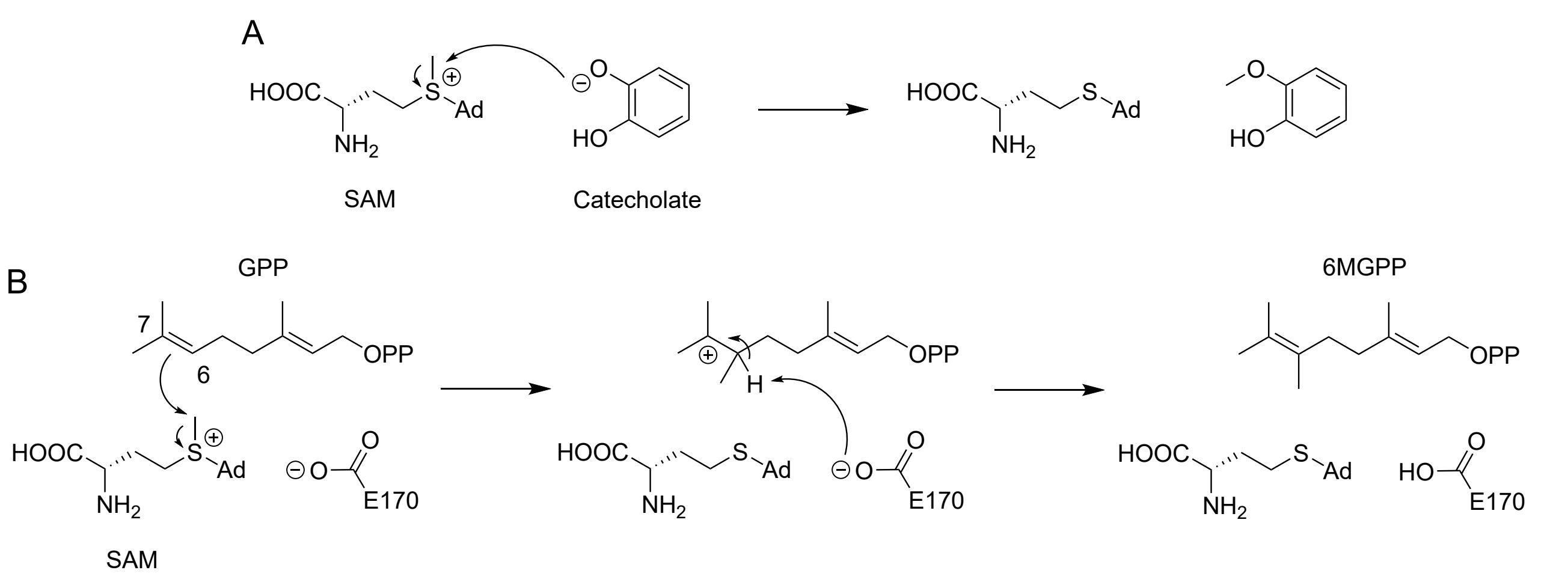

Scheme S1: Reaction mechanisms of the two application case studies. **(A)** COMT: single-step S_N2 methyl transfer from SAM to the catechol anion, forming the methylated catechol and S-adenosyl-L-homocysteine (SAH, the demethylated SAM cofactor). **(B)** BezA:[43] S_N2 methyl transfer from SAM to the geranyl pyrophosphate (GPP) C6 forms a C7 carbocation intermediate, which Glu170 deprotonates at C6 to yield 6-methylgeranyl pyrophosphate (6MGPP).

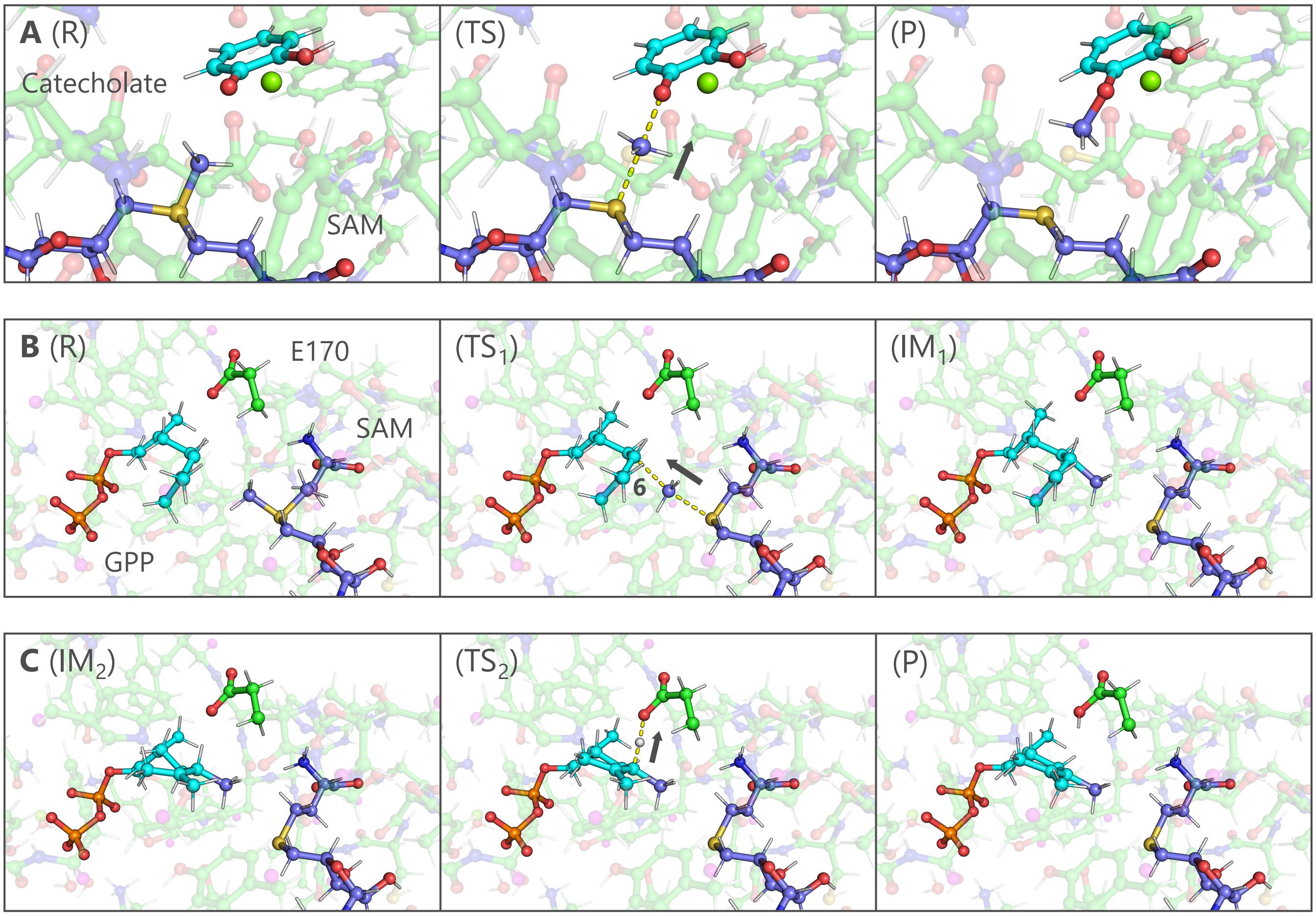

Figure S1: Active-site structural evolution along the IRC-validated paths (scan-list mode). **(A)** COMT: R → TS → P single-step S_N2 methyl transfer; the dashed yellow line in TS marks the C_E(SAM)–O_2(catechol) forming bond, and the arrow indicates the methyl trajectory. **(B)** BezA step 1: R → TS_1 ("6" marks the GPP C6 acceptor) → C7 carbocation IM_{1_1}. **(C)** BezA step 2: IM_{1_2} → TS_2 → 6-methylgeranyl pyrophosphate product (P). IM_{1_1} and IM_{1_2} differ only by conformational relaxation from the two IRC endpoints.

S5.1 Reference QM/MM models and how they differ from the cluster models used here

Both case studies start from published QM/MM stationary points, and the literature barriers we quote alongside our results were obtained with those QM/MM models—not with a cluster model. The two protocols therefore describe different systems, and the comparison is a cross-model one. The complete set of reference stationary points is shipped with the Supporting Data (Section S1.6).

COMT.

The reactant and product are the optimized geometries of model 10 of Kulik et al.[44]—the model with the largest QM region in their QM-region-convergence study—computed at ωPBEh/6-31G:AMBER (ff12SB/GAFF) in TeraChem; the corresponding transition state was located there with the nudged-elastic-band method and gives $\Delta E^{\ddagger} = 15.9$ kcal mol^{-1}. Our cluster model is a 670-atom excision from those coordinates, treated in full at the MLIP level.

BezA.

The reactant, intermediate and product are the ONIOM stationary points of Tsutsumi et al.[43] (model M1), computed with ONIOM(B3LYP/6-31++G(d,p)//B3LYP/6-31+G(d,p) : ff14SB+GAFF2/TIP3P) in Gaussian 16 on a ~9,200-atom system taken from an MD snapshot; the reported barriers are $\Delta E^{\ddagger} = 12.4$ kcal mol^{-1} (methyl transfer) and 0.2 kcal mol^{-1} (deprotonation). Their QM region contains Glu170, Phe214, Ser277, Asn281, GPP, the Mg^{2+} ion, one coordinating water, and the part of SAM around the transferring methyl group; the remainder of the ~9,200-atom system is treated at the MM level. Our BezA cluster model, by contrast, treats all 669 atoms—SAM in its entirety included—at the MLIP level, with a frozen cap-hydrogen boundary and no surrounding MM environment.

Consequences for the comparison.

The extent of the region described by a reactive potential (the QM region in the reference models; the MLIP-treated cluster here), the boundary treatment and the electrostatic environment all differ between the two protocols, so a numerical difference in the barrier is expected on model grounds alone and is not, by itself, evidence about the accuracy of the MLIP. This is why the deviations reported in Tables S28 and S29 are presented as cross-model comparisons, and why we do not treat the literature values as a ground truth against which the pipeline is scored.

S5.2 Structural comparison with the reference stationary points

To separate the geometric from the energetic content of that comparison, we superposed the pipeline's stationary points on the reference ones. The superposition uses only the frozen boundary atoms (the cap-hydrogen parents, 18 for COMT and 26 for BezA), which our runs hold at the coordinates taken from the input cluster; the reported RMSDs are therefore internal deformations of the active site rather than rigid-body offsets. All values are heavy-atom, cap hydrogens excluded (COMT: 332 atoms; BezA: 318 atoms); the "reactive core" is SAM/catecholate/Mg(II) for COMT (36 heavy atoms) and SAM/GPP/Mg(II)/Glu170 for BezA (56 heavy atoms).

Table S27: Pipeline (UMA-s-1.2, scan-list) versus QM/MM reference stationary points. *Top*: RMSD of the pipeline transition state from the corresponding reference transition state. *Bottom*: heavy-atom displacement along each elementary step within each model, i.e. how much of the active site moves between the two stationary points of that step. All values in Å.

System	Transition state	Heavy-atom RMSD	Reactive-core RMSD	Max. deviation
COMT	TS	0.91	0.75	4.57
BezA	TS_1	0.82	0.43	3.98
BezA	TS_2	0.84	0.61	4.12
Displacement along the step		Reference	Pipeline	
COMT	R → TS	0.26 (13)	0.11 (1)	
BezA	R → TS_1	0.05 (0)	0.14 (3)	
BezA	IM → TS_2	0.02 (0)	0.35 (45)	

Displacements are RMS values over the heavy atoms; the number in parentheses is how many heavy atoms move by more than 0.5 Å in that step.

The reactive cores of the transition states agree: the pipeline's TS deviates from the reference TS by 0.43–0.75 Å over the reacting fragments, while the larger whole-cluster RMSD (0.8–0.9 Å) is carried by the periphery—side chains and active-site waters that the isolated cluster relaxes but the surrounding protein holds in place in the QM/MM model.

The two systems differ in how the active site responds *along* the reaction. In BezA, the reference geometries are essentially rigid between the stationary points—no heavy atom moves by more than 0.5 Å in either step—whereas the all-MLIP cluster lets Asp168 relax together with the transferring methyl group in step 1 (0.83 Å on OD2, against 0.00 Å in the reference) and reorganizes a large part of the pocket in step 2 (45 heavy atoms beyond 0.5 Å, led by Ile185, Ser188 and Asp168). In COMT, whose reference QM region spans 940 atoms and 56 residues, the pattern is reversed: there the reference is free to respond

along the reaction (RMS displacement 0.26 Å, 13 heavy atoms beyond 0.5 Å between R and TS) while our cluster barely moves (0.11 Å, 1 atom; Table S27).

The deviation is not an error in the MLIP energy. Re-scoring the stationary points with ωB97M-V/def2-SVP reproduces the MLIP step-1 barrier of BezA to within 0.8 kcal mol^{-1} (25.6 against 24.9 in scan-list mode, 24.5 against 23.7 in endpoint mode; Table S29), while both sit some 12 kcal mol^{-1} above the ONIOM value of 12.4. A DFT energy evaluated on the same geometries therefore rules out the MLIP energies at those geometries as the source of the deviation; it cannot speak to the geometries themselves, which the MLIP surface produced. That difference is not of the same size in the two case studies: for COMT our barrier lands 1.2 kcal mol^{-1} from the reference (17.1 against 15.9), whereas for BezA it lies 12.5 kcal mol^{-1} above it. The two protocols differ in the extent of the region described by a reactive potential, in the boundary treatment and in the electrostatic environment (Section S5.1), and the present data do not resolve which of these accounts for the BezA deviation.

S5.3 Case study output

Figures S2–S5 show the three diagnostic plots written by `pdb2reaction all` for the two main-text applications (COMT scan-list and endpoint mode; BezA scan-list and endpoint mode): the MEP energy trace after `path-opt` (COMT, single elementary step) or the recursive `path-search` (BezA, `--refine-path`, two elementary steps), the IRC profile concatenated across reactive segments, and the QRRHO Gibbs free-energy diagram for the full path. Stationary-point energies are tabulated in Tables S28 (COMT) and S29 (BezA). The computed ΔG are QRRHO values at 298 K, whereas the experimental $\Delta G^{\ddagger}$ in Table S28 is evaluated at its 37 °C measurement temperature. All four runs use UMA-s-1.2 (the pipeline default backend) on a single CUDA-capable NVIDIA GPU in double precision; backend robustness of the BezA mechanism across the other MLIP backends is examined separately in Table S31. In the concatenated IRC panels, each segment's IRC starts at its TS and descends to both flanking minima, so within a segment the TS is the energy maximum. The BezA scan-list run (Figure S4) is the same run shown as the solid (scan-list) curve in main-text Figure 3B.

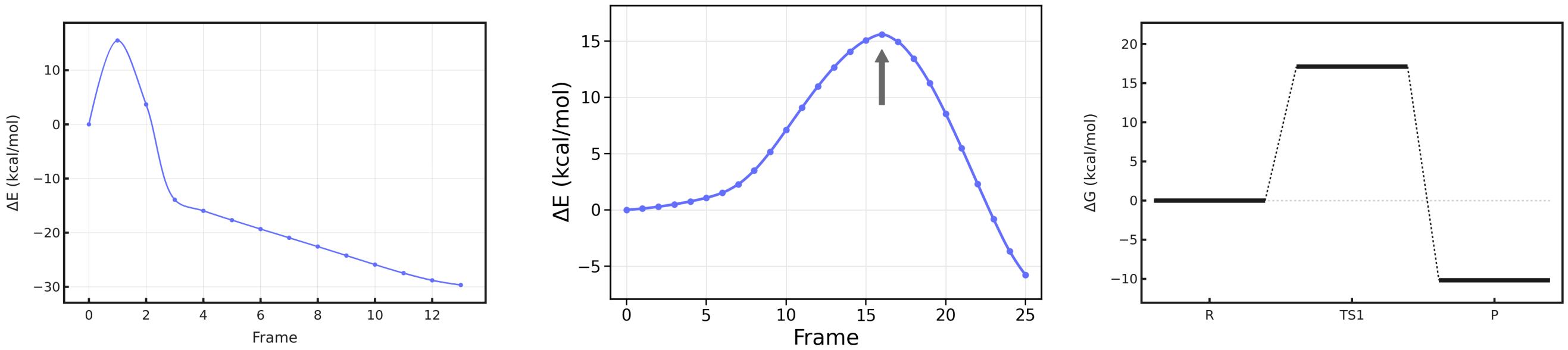

(a) Potential Energy along MEP (b) Potential Energy along IRC (c) Gibbs free-energy diagram

Figure S2: COMT SAM-dependent methyl transfer, **scan-list mode**. (a) Final MEP energy trace after `path-opt`; single peak, one elementary step. (b) Concatenated IRC profile after `tsopt`; the arrow marks the TS, which connects a well-defined R and P basin. (c) QRRHO Gibbs free-energy diagram for R/TS/P, giving $\Delta G^\ddagger = 17.11\ \mathrm{kcal\,mol^{-1}}$ (UMA-s-1.2, fp64).

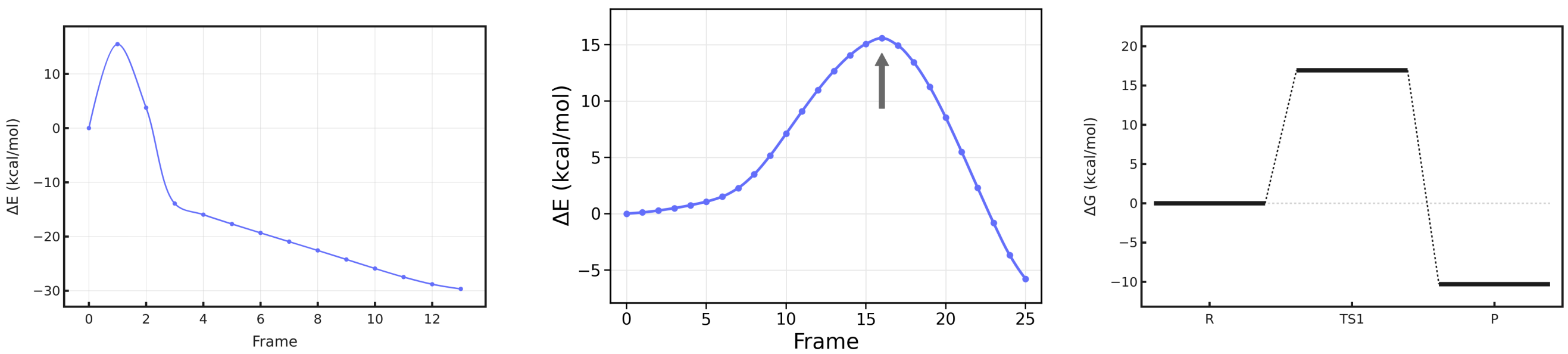

(a) Potential Energy along MEP (b) Potential Energy along IRC (c) Gibbs free-energy diagram

Figure S3: COMT SAM-dependent methyl transfer, **endpoint mode**. Same three plots as Figure S2 for the endpoint run on the same cluster (R and P inputs share a coincident freeze-atom boundary from the same QM/MM optimization). (c) gives $\Delta G^\ddagger = 16.94\ \mathrm{kcal\,mol^{-1}}$ (UMA-s-1.2, fp64).

Table S28: COMT case-study energetics (UMA-s-1.2). Stationary-point energies relative to R for the scan-list and endpoint modes (MLIP ΔE and QRRHO ΔG at 298 K), the DFT//MLIP ωB97M-V/def2-SVP single-point re-score on the same UMA-s-1.2 geometries, the QM/MM literature reference of Kulik et al.[44] (ωPBEh/6-31G:AMBER ff12SB/GAFF, optimized model with the largest QM region; transition state located via NEB), and the experimental $\Delta G^\ddagger$ obtained from $k_{\mathrm{cat}} = 24\ \mathrm{min^{-1}}$ ($0.40\ \mathrm{s^{-1}}$; measured at 37 °C) via the Eyring equation ($\kappa = 1$) evaluated at that measurement temperature; as for BezA, this is an apparent barrier for the rate-limiting step and not necessarily the elementary chemical step computed here.[45] All values in $\mathrm{kcal\,mol^{-1}}$.

Method / mode	R	TS	P
Scan-list, MLIP ΔE	0	17.06	−12.6
Scan-list, MLIP ΔG	0	17.11	−10.2
Scan-list, DFT//MLIP ΔE	0	13.6	−17.5
Endpoint, MLIP ΔE	0	17.08	−12.6
Endpoint, MLIP ΔG	0	16.94	−10.3
Endpoint, DFT//MLIP ΔE	0	13.5	−17.5
Lit. QM/MM ΔE (Kulik et al. 2016, Model 10)[44]	0	15.9	−11.2
Exp. $\Delta G^\ddagger$ (Schultz & Nissinen 1989)‡ [45]	—	18.7	—

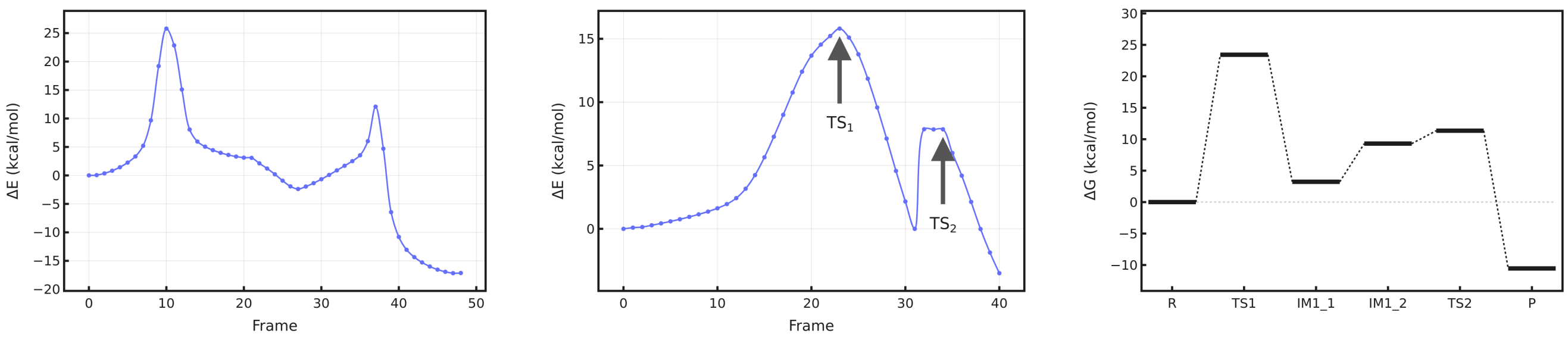

(a) Potential Energy along MEP (b) Potential Energy along IRC (c) Gibbs free-energy diagram

Figure S4: BezA two-step methyl transfer, **scan-list mode**. (a) Final MEP energy trace after recursive `path-search`; two peaks (TS_1 and TS_2) separated by the cationic intermediate basin. (b) Concatenated IRC traces from both reactive segments; the arrows mark TS_1 and TS_2. The discontinuity between frames 31 and 32 marks the segment boundary: the two IRCs are concatenated rather than joined by a continuous path. (c) QRRHO Gibbs free-energy diagram spanning R $\to$ TS_1 $\to$ IM_{1_1} and IM_{1_2} $\to$ TS_2 $\to$ P (IM_{1_1} and IM_{1_2} are the two IRC-relaxed endpoints of the same cationic intermediate, differing only by conformational relaxation). Rate-limiting $\Delta G^{\ddagger} = 23.4$ kcal mol^{-1} (UMA-s-1.2).

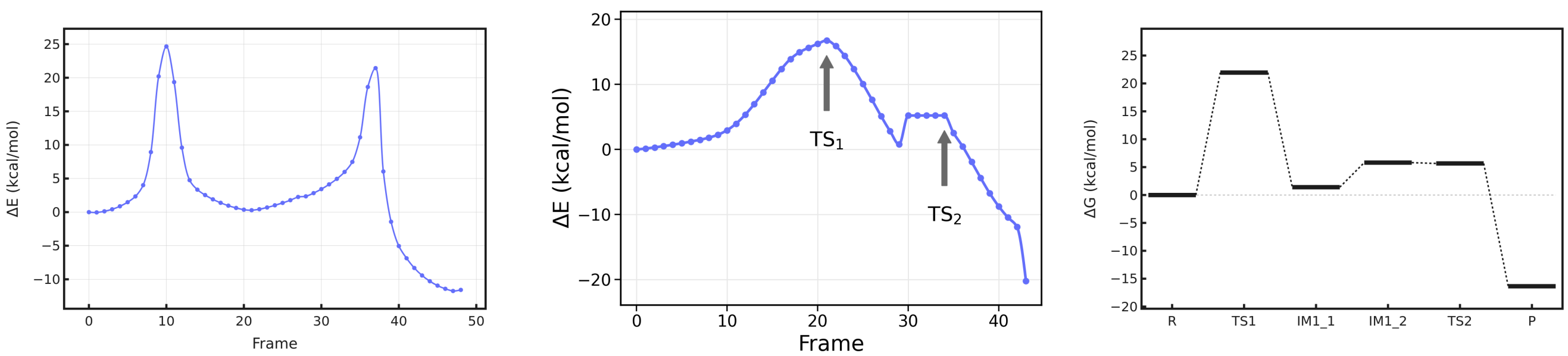

(a) Potential Energy along MEP (b) Potential Energy along IRC (c) Gibbs free-energy diagram

Figure S5: BezA two-step methyl transfer, **endpoint mode** (UMA-s-1.2). Same three plots as Figure S4 for the endpoint run (R and P from independent optimizations; freeze-atom boundaries differ slightly). (a) Two peaks flanking the cationic intermediate basin. (c) Rate-limiting $\Delta G^{\ddagger} = 21.9$ kcal mol^{-1} at TS_1; the cationic IM_{1_1} is at 1.4 kcal mol^{-1}. Stationary-point energies for both modes are tabulated in Table S29.

Table S29: BezA case-study energetics. Stationary-point energies are relative to R; each TS_2 entry gives the R-referenced energy followed in parentheses by the local step-2 barrier relative to IM_{1_2}. The rows compare scan-list and endpoint modes (UMA-s-1.2; MLIP ΔE and QRRHO ΔG at 298 K), DFT//MLIP ωB97M-V/def2-SVP single-point re-scores, and the ONIOM literature reference of Tsutsumi et al.[43] All values are in kcal mol^{-1}.

Method / mode	R	TS_1	IM_{1_1}	IM_{1_2}	TS_2	P
Scan-list (UMA-s-1.2), MLIP ΔE	0	24.9	6.5	11.4	16.9 (5.5)	−7.6
Scan-list (UMA-s-1.2), MLIP ΔG	0	23.4	3.2	9.3	11.3 (2.1)	−10.5
Scan-list (UMA-s-1.2), DFT//MLIP ΔE	0	25.6	8.8§	8.8§	15.0 (6.2)	−7.4
Endpoint (UMA-s-1.2), MLIP ΔE	0	23.7	3.6	11.1	12.2 (1.1)	−13.2
Endpoint (UMA-s-1.2), MLIP ΔG	0	21.9	1.4	5.8	5.7 (−0.2)	−16.4
Endpoint (UMA-s-1.2), DFT//MLIP ΔE	0	24.5	4.3	9.1	8.4 (−0.7)	−16.5
Lit. ONIOM ΔE (Tsutsumi et al. 2022, M1)[43]	0	12.4	—	2.9†	3.1 (0.2)†	−26.7
Apparent exp. $\Delta G^{\ddagger}$ (Tsutsumi et al. 2022)‡[43]	—	16.1	—	—	—	—

† The literature reports a single cationic intermediate (Tsutsumi et al. 2022 Figure 4f, Model M1: IM $= 2.9$ kcal mol^{-1} vs. R), with the TS_2 barrier reported as 0.2 kcal mol^{-1} vs. IM (TS_2 absolute = IM $+0.2 = 3.1$ kcal mol^{-1}). The lit. IM is mapped onto the post-relaxation IM_{1_2} column for closest correspondence with our path-search-resolved geometries.

‡ Apparent $\Delta G^{\ddagger}$ from $k_{cat}/K_m = (1.67 \pm 0.11) \times 10^{-2}$ mM^{-1} s^{-1} for GPP at 30 °C via the Eyring equation ($\kappa = 1$); not the elementary chemical-step barrier.

Parenthetical MLIP barriers are calculated from unrounded stationary-point energies before rounding to 0.1 kcal mol^{-1}; they can therefore differ by 0.1 from subtraction of the displayed values. The endpoint-mode product state is a clean minimum (no imaginary frequencies).

§ The scan-list DFT//MLIP re-score used a single representative geometry (the seg_1 IRC endpoint) for the cationic intermediate, reported under both IM_{1_1} and IM_{1_2}.

The COMT barrier is also insensitive to the choice of backend. Repeating the case study on the other MLIP backends, in both input modes, gives eight clean runs across four backends (Table S30). Every run returns the single-step S_N2 methyl transfer

with one imaginary mode, and the rate-limiting $\Delta G^\ddagger$ spans 16.7–18.8 kcal mol^{-1}, a range that contains the 18.7 kcal mol^{-1} apparent experimental barrier. The two input modes agree to within 1.0 kcal mol^{-1} for every backend.

Table S30: COMT across MLIP backends and input modes (670-atom cluster of Table S26). All runs give a clean transition state ($n_{\text{imag}} = 1$). $\Delta E^\ddagger$ and $\Delta G^\ddagger$ are the electronic and QRRHO (298 K) barriers of the methyl transfer. A separate UMA-s-1.1 run with the Direct-MaxFlux driver (50 nodes; Section S7) returned a higher-order saddle and is not part of this backend/mode grid. UMA-m-1.1 was not run for this system.

Backend	Input mode	$\Delta E^\ddagger$ (kcal mol^{-1})	$\Delta G^\ddagger$ (kcal mol^{-1})
UMA-s-1.2	scan-list	17.1	17.1
UMA-s-1.2	endpoint-MEP	17.1	16.9
UMA-s-1.1	scan-list	17.2	17.6
UMA-s-1.1	endpoint-MEP	17.2	17.6
Orb-v3-omol	scan-list	16.1	16.7
Orb-v3-omol	endpoint-MEP	16.3	17.7
MACE-OMOL-0	scan-list	19.2	18.7
MACE-OMOL-0	endpoint-MEP	19.2	18.8

To confirm that the BezA mechanism is not specific to the main-text backend, we repeated the case study on all five MLIP backends from the identical 669-atom active-site cluster, in both input modes. Every backend recovers the same two-step mechanism — a rate-limiting SAM→GPP methyl transfer followed by a comparatively low-barrier Glu170-mediated C6–H deprotonation — with each step giving a clean transition state ($n_{\text{imag}} = 1$), and each backend reaches it from at least one entry point. Each input mode covers four of the five, and the two sets are complementary rather than the same four: the endpoint-MEP runs recover the mechanism for UMA-s-1.1, UMA-s-1.2, Orb-v3-omol, and MACE-OMOL-0 (Table S31), and the scan-list runs for UMA-s-1.1, UMA-s-1.2, UMA-m-1.1, and Orb-v3-omol (Table S32), with UMA-m-1.1 locating no reactive segment in endpoint mode and MACE-OMOL-0 none in scan-list mode. The union of the two modes therefore covers every backend, and a backend that returns no reactive segment from one entry point may still be reached from the other.

Table S31: BezA two-step mechanism across MLIP backends (endpoint-MEP mode, double precision, from the shared 669-atom active-site cluster). Step 1 is the rate-limiting SAM→GPP methyl transfer and step 2 the Glu170-mediated C6–H deprotonation (barrier relative to the cationic intermediate). Per-run geometries are archived in the Supporting Data archive (`bezA/results/`). All values in kcal mol^{-1}.

	Step 1 (methyl, rate-limiting)		Step 2 (deprot.)	
Backend	$\Delta E^\ddagger$	$\Delta G^\ddagger$	$\Delta E^\ddagger$	$\Delta G^\ddagger$
UMA-s-1.1	26.4	26.8	10.7	9.5
UMA-s-1.2	23.7	21.9	1.1	−0.2*
Orb-v3-omol	27.3	21.8	2.6	1.0
MACE-OMOL-0	32.2	31.1	1.8	−0.6

* Rounded from the raw basin-referenced value (−0.15 kcal mol^{-1}); subtracting the individually-rounded stationary-point energies in Table S29 instead gives −0.1 (5.7−5.8), a round-then-subtract vs. subtract-then-round artifact at this level of precision, not a data discrepancy.

In endpoint-MEP mode the rate-limiting step-1 barrier spans $\Delta G^\ddagger = 21.8$–31.1 kcal mol^{-1} across backends, while step 2 is essentially barrierless for three of the four backends (UMA-s-1.2, Orb-v3-omol, and MACE-OMOL-0; $\Delta G^\ddagger \leq 1.0$) and 9.5 kcal mol^{-1} for UMA-s-1.1—still 17 kcal mol^{-1} below that backend's own step-1 barrier, so step 1 is rate-limiting for every backend. The endpoint-mode row of Table S29 and main-text Figure 3B both use the default UMA-s-1.2 backend.

Table S32: BezA two-step mechanism across MLIP backends in **scan-list mode** (double precision, from the same shared 669-atom active-site cluster as Table S31); steps and barrier conventions are as in that table, and both steps again give a clean TS ($n_{\text{imag}} = 1$) for every backend shown. MACE-OMOL-0 is absent because its scan-list run located no reactive segment. Per-run geometries are archived in the Supporting Data archive (`bezA/results/`). All values in kcal mol^{-1}.

	Step 1 (methyl, rate-limiting)		Step 2 (deprot.)	
Backend	$\Delta E^\ddagger$	$\Delta G^\ddagger$	$\Delta E^\ddagger$	$\Delta G^\ddagger$
UMA-s-1.1	27.4	27.9	1.0	1.2
UMA-s-1.2	24.9	23.4	5.5	2.1
UMA-m-1.1	26.6	26.7	8.2	4.6
Orb-v3-omol	28.1	20.6	3.8	2.2

The scan-list runs (Table S32) give the same picture: step 1 is rate-limiting for every backend, with $\Delta G^{\ddagger} = 20.6$–$27.9$ kcal mol^{-1}, and step 2 is low-barrier throughout ($\Delta G^{\ddagger} \leq 4.6$). UMA-m-1.1, the backend with the broadest coverage on the 23-step benchmark, sits in the upper part of that range (26.7).

■ S6. BENCHMARK SYSTEM DETAILS

This section describes the six benchmark enzyme systems (Table S33), including cluster model composition, total charge, computational methods from the original DFT studies, and the correspondence between our model labels and the labels used in the original studies. The reported literature barriers ($\Delta E^{\ddagger}$) are B3LYP single-point energies on B3LYP-optimized geometries with zero-point energy (ZPE) corrections included. The MLIP barriers reported here are electronic-energy differences on MLIP-optimized geometries. Consequently, the numerical deviations and MAEs against the literature values are cross-method external comparisons that combine differences in electronic-structure level, optimized geometry, and vibrational treatment; they are not like-for-like estimates of MLIP error. The cluster-model XYZ files were taken from the enzyme geometry collection at `https://github.com/jensengroup/db-enzymes`, accompanying the Kromann et al. (2016)[46] cluster-model barrier-height benchmark. We retained the six systems whose published XYZ coordinates have atom ordering compatible with automated bond-change detection. Input structures for all benchmark systems (per-system XYZ geometries and the YAML/shell files needed to reproduce each run), together with the BezA case-study inputs, are bundled with this Supporting Information.

The five MLIP backends benchmarked here—UMA-s-1.1,[47] UMA-s-1.2,[1] UMA-m-1.1, MACE-OMOL-0,[48,49] and Orb-v3-omol[49–51]—span three contemporary MLIP architectures, are all evaluated on OMol25-trained[49] checkpoints or tasks, and cover the OMol25-trained foundation models included in the recent accuracy and efficiency evaluation of Eastman et al.;[52] the UMA-small v1.1/v1.2 pair and the UMA small/medium split probe successive checkpoint releases and model-size effects within one architecture.

Table S33: Benchmark enzyme systems. "Atoms" gives the cluster-model size range across the variants tested for each enzyme; "Steps" is the number of elementary reaction steps modeled.

PDB	Enzyme	EC	Metal	Hetero	Atoms	Steps	Reaction type
1AH7	Phospholipase C	3.1.4.3	Zn_3	P	105–111	1–2	Phosphodiester hydrolysis
1O9S	Histone Lys methyltransferase	2.1.1.364	—	S	29–132	1	S_N2 methyl transfer
1PWZ	Haloalcohol dehalogenase	4.5.1.—	—	Cl	83–161	1	Dehalogenation
1RTQ	Aminopeptidase	3.4.11.10	Zn_2	—	82	4	Peptide hydrolysis
2E7Z	Acetylene hydratase	4.2.1.112	W	S	116–117	1–4	Acetylene hydration
4OTA	4-OT tautomerase	5.3.2.6	—	—	77–177	2	Tautomerization (proton transfer)

Literature barriers across the 23 individual reaction steps range from -2.8 to 33.4 kcal mol^{-1} and span phosphodiester hydrolysis, S_N2 methyl transfer, dehalogenation, peptide hydrolysis, tungsten-catalyzed hydration, and proton-relay tautomerization. A negative value means the transition state lies below its preceding intermediate at that level of theory; the source papers attribute these to the method and the cluster model—chiefly insufficient solvation of a charge-separated intermediate—and conclude only that the step is very fast. Table S34 lists every one of the 23 steps individually, with the elementary event it represents and the class of chemistry it belongs to; the per-system subsections below give the full mechanism and the model definitions. Three of the six systems contribute several *cluster models of the same reaction* rather than several different reactions—the four 1O9S models, the three 1PWZ models, and the two 4OTA models (which run the same two steps on a 77- and a 177-atom cluster)—so the 23 steps comprise 16 chemically distinct elementary transformations, not 23. They are nevertheless counted and scored separately, because reproducing the same reaction from clusters of very different size is exactly the robustness the pipeline has to deliver.

S6.1 1AH7 — Phospholipase C (Trinuclear Zn_3)

Reference: Liao, Yu, and Himo, *J. Phys. Chem. B* **2010**, 114, 2533–2540.[53]

Reaction: Phosphodiester hydrolysis of a dimethyl phosphate model substrate catalyzed by a trinuclear zinc cluster (Zn1, Zn2, Zn3). The favored sequential mechanism is split across Models B (step 1) and Ba (step 2): nucleophilic attack by the Zn1/Zn3-bridging hydroxide on phosphorus (Model B; barrier 20.9 kcal mol^{-1}), followed by product release via Zn2-bound hydroxide attack (Model Ba; barrier 3.9 kcal mol^{-1}). Model A represents the alternative concerted pathway (barrier 22.8 kcal mol^{-1}).

Models:

- Model A: 109 atoms, charge $+2$, singlet ($S = 0$). Corresponds to the concerted mechanism of Ref. 53.
- Model B: 111 atoms (R, TS1, IM_1), charge $+1$, singlet. Step 1 of the sequential mechanism of Ref. 53.
- Model Ba: 105 atoms (a smaller cluster distinct from Model B), step 2 of the sequential mechanism. Charge $+1$, singlet.

DFT methods: Geometry optimization: B3LYP/6-31G(d,p) for C, H, N, O; B3LYP/6-311+G(2d) for P and surrounding oxygens; B3LYP/LANL2DZ for Zn. Single-point: B3LYP/6-311+G(2d,2p) (Gaussian 03).

[1] UMA-s-1.2 is a post-preprint `fairchem`-distributed checkpoint of the UMA family and is not described in the original UMA preprint;[47] see `https://huggingface.co/facebook/UMA` for the release.

Table S34: The 23 benchmark steps, one row each: the elementary event and the class of chemistry it belongs to. "Same reaction, N-atom model" marks a step that repeats the preceding reaction on a larger cluster model rather than introducing new chemistry. $\Delta E^{\ddagger}$ is the ZPE-corrected literature B3LYP/6-311+G(2d,2p) reference barrier used throughout this section, measured from the step's own reactant. All barriers are in kcal mol^{-1}.

PDB	Model / step	Class	Elementary event	$\Delta E^{\ddagger}$
1AH7	A	Substitution at P	Alternative pathway: a terminal Zn-bound *water* attacks P as the leaving alkoxide departs	22.8
	B	Substitution at P	Favored pathway, step 1: the Zn1/Zn3-bridging *hydroxide* attacks P, concerted with departure of the leaving alcohol	20.9
	Ba	Substitution at P	Favored pathway, step 2 (turnover): the Zn2-bound hydroxide attacks P, displacing a phosphate oxygen and regenerating the bridging hydroxide	3.9
1O9S	A	S_N2 at C	In-line SAM methyl transfer S→N_ζ(Lys) with Walden inversion (29-atom model)	18.8
	B	S_N2 at C	Same reaction, 46-atom model (first shell added)	21.7
	C	S_N2 at C	Same reaction, 72-atom model (Tyr245, Tyr305 added)	15.4
	D	S_N2 at C	Same reaction, 132-atom model (full second shell)	18.9
1PWZ	A	S_N2 at C	Concerted, Tyr145-assisted intramolecular epoxide closure displacing Cl^- (83-atom model)	23.0
	B	S_N2 at C	Same reaction, 112-atom model (halide-binding residues added)	17.9
	C	S_N2 at C	Same reaction, 161-atom model (full halide-binding site)	18.2
1RTQ	step 1	Addition to C=O	μ-hydroxide attacks the peptide carbonyl C, giving the tetrahedral *gem*-diolate	12.1
	step 2	Proton transfer	Glu151 protonates the peptide nitrogen	0.1
	step 3	Proton transfer	Proton relay from the former hydroxide O back to Glu151	-0.8
	step 4	C–N cleavage	C–N scission of the tetrahedral intermediate, releasing the amine	-0.6
2E7Z	A / step 1	Addition to C≡C	Favored pathway: Asp13$^-$-assisted water attack on η^2-bound acetylene C1, giving a vinyl anion	19.8
	A / step 2	Proton transfer	Asp13–COOH protonates the vinyl anion at C2	5.5
	A / step 3	Proton transfer	Asp13$^-$ abstracts the vinyl alcohol O–H (tautomerization, part 1)	9.5
	A / step 4	Proton transfer	Asp13–COOH protonates C2, giving acetaldehyde (tautomerization, part 2)	14.7
	B	Addition to C≡C	Alternative (electrophilic) pathway: neutral Asp13 protonates C1 while the Cys141 thiolate attacks C2, concertedly; the barrier is measured from the IM1 intermediate (this model's left input), not from the free reactant	33.4
4OTA	A / step 1	Proton transfer	Pro1 abstracts the substrate C3–H (77-atom model)	12.8
	A / step 2	Proton transfer	Pro1–H^+ delivers the proton to C5, completing the 1,3-shift (77-atom model)	-2.8
	B / step 1	Proton transfer	Same as A/step 1, 177-atom model	6.9
	B / step 2	Proton transfer	Same as A/step 2, 177-atom model	4.1

S6.2 1O9S — Histone Lysine Methyltransferase (S_N2)

Reference: Georgieva and Himo, *J. Comput. Chem.* **2010**, 31, 1707–1714.[54]

Reaction: In-line S_N2 methyl transfer from the sulfonium of S-adenosylmethionine (SAM) to the ε-amino group of a target lysine residue. The S–C bond breaks and the N–C bond forms through a single transition state with Walden inversion.

Models: Four cluster models of increasing size to probe convergence:

- Model A: 29 atoms, charge $+1$. Substrate amine + SAM sulfonium and methyl group.
- Model B: 46 atoms, charge $+1$. Adds first-shell residues.
- Model C: 72 atoms, charge $+1$. Adds Tyr245 and Tyr305.
- Model D: 132 atoms, charge $+1$. Full second-shell environment (Tyr335, Asn265, Lys294, water).

Experimental $\Delta G^{\ddagger} = 20.9\ \text{kcal mol}^{-1}$ (from k_{cat}).

DFT methods: Geometry optimization: B3LYP/6-31G(d,p). Single-point: B3LYP/6-311+G(2d,2p) (Gaussian 03).

S6.3 1PWZ — Haloalcohol Dehalogenase

Reference: Hopmann and Himo, *J. Chem. Theory Comput.* **2008**, 4, 1129–1137.[55]

Reaction: Intramolecular S_N2 dehalogenation of (R)-2-chloro-1-phenylethanol to (R)-styrene oxide + Cl^-. Tyr145 (phenolate) acts as general base, abstracting the substrate hydroxyl proton while the resulting oxyanion attacks the adjacent carbon, forming the epoxide ring and displacing chloride.

Models: Three cluster models of increasing size:

- Model A: 83 atoms, charge 0, singlet. Substrate + catalytic triad (Ser132, Tyr145, Arg149).
- Model B: 112 atoms, charge 0, singlet. Adds halide-binding residues.
- Model C: 161 atoms, charge 0, singlet. Full binding pocket.

DFT methods: Geometry optimization: B3LYP/6-31G(d,p). Single-point: B3LYP/6-311+G(2d,2p) (Gaussian 03).

S6.4 1RTQ — Aminopeptidase (Binuclear Zn_2, 4-Step)

Reference: Chen, Marino, Fang, Russo, and Himo, *J. Phys. Chem. B* **2008**, 112, 2494–2500.[56]

Reaction: Four-step peptide bond hydrolysis:

1. Nucleophilic attack by the bridging μ-hydroxide on the peptide carbonyl carbon.
2. Proton transfer from Glu151 to the peptide nitrogen.
3. Proton relay from the former hydroxide oxygen back to Glu151.
4. C–N peptide bond cleavage, releasing carboxylate + amine products.

Model: 82 atoms, charge 0, singlet. Steps 1–4 use the same cluster model with sequential intermediate structures.

DFT methods: Geometry optimization: B3LYP/6-31G(d,p) for C, H, N, O; Stuttgart/Dresden ECP basis set for Zn. Single-point: B3LYP/6-311+G(2d,2p) (Gaussian 03).

S6.5 2E7Z — Acetylene Hydratase (Tungsten-Dependent, 4-Step)

Reference: Liao, Yu, and Himo, *Proc. Natl. Acad. Sci. U.S.A.* **2010**, 107, 22523–22527.[57]

Reaction (Model A, preferred mechanism): Four-step hydration of acetylene to acetaldehyde at a W(IV) active site with thiolate ligands:

1. Nucleophilic water attack on η^2-bound acetylene C1, with $Asp13^-$ as general base.
2. Protonation of the vinyl anion at C2 by Asp13-COOH.
3. Tautomerization part 1: $Asp13^-$ abstracts the vinyl alcohol O–H.
4. Tautomerization part 2: Asp13-COOH protonates C2, yielding acetaldehyde.

Reaction (Model B, alternative mechanism): Concerted electrophilic protonation of acetylene by neutral Asp13, with Cys141 thiolate attack. Corresponds to the neutral-Asp13 mechanism of Ref. 57.

Models:

- Model A: 116 atoms, charge -1, singlet. Ionized-Asp13 mechanism of Ref. 57.
- Model B: 117 atoms, charge 0, singlet. Neutral-Asp13 mechanism of Ref. 57.

DFT methods: Geometry optimization: B3LYP/6-31G(d,p) for C, N, O, H; B3LYP/6-311+G(d) for S; B3LYP/LANL2TZ(f) for W. Single-point: B3LYP/6-311+G(2d,2p) (Gaussian 03).

S6.6 4OTA — 4-Oxalocrotonate Tautomerase (2-Step)

Reference: Sevastik and Himo, *Bioorg. Chem.* **2007**, 35, 444–457.[58]

Reaction: 1,3-Allylic proton shift (tautomerization) of 2-oxo-4-hexenedioate, catalyzed by Pro1 as a proton shuttle:

1. Pro1 (N, deprotonated) abstracts a proton from C3 of the substrate (rate-limiting).
2. Protonated Pro1 donates the proton to C5, generating the conjugated isomer.

Arg39′ stabilizes the dienolate intermediate via hydrogen bonding to the C2 carbonyl oxygen.

Models:

- Model A: 77 atoms, charge $+1$, singlet. Substrate + Pro1 + 3 Arg + 2 waters.
- Model B: 177 atoms, charge $+1$, singlet. Adds Phe50′, Ile7, Leu8, Ser37, and extended side chains.

DFT methods: Geometry optimization: B3LYP/6-31G(d,p). Single-point: B3LYP/6-311+G(2d,2p) (Gaussian 03).

Literature-value note. In Model B/step 2, the prose of Sevastik and Himo[58] quotes $\Delta E^{\ddagger} = 4.0$ kcal mol^{-1} while the value tabulated in the Kromann 2016 benchmark repository and used here as the literature baseline is 4.1 kcal mol^{-1}; the 0.1 kcal mol^{-1} difference is within the rounding used in the original paper and does not affect any downstream pass/fail or mean-absolute-error (MAE) conclusion.

S6.7 Qualitative Reaction Reproduction

Table S35 summarizes the qualitative pass/fail results for all 23 reaction steps across five backends under the default pipeline settings (finite-difference Hessian, `--refine-path` off, i.e. single-pass path optimization). UMA-m-1.1 has the broadest coverage (19 of 23 steps), and its passing set is a strict superset of MACE-OMOL-0's (18 of 23): UMA-m-1.1 passes every step MACE-OMOL-0 passes, plus 1AH7 A. Adding UMA-s-1.1 or UMA-s-1.2 (17 of 23 each) does not extend the union beyond 19 of 23; adding Orb-v3-omol (16 of 23) brings the union of all five backends to 20 of 23, with three steps failing on every backend tested: 2E7Z A/step 3 and 2E7Z B (both tungsten-dependent) and 4OTA A/step 2.

The five backends differ little in TS-spectrum quality under the default condition. UMA-m-1.1, MACE-OMOL-0, and UMA-s-1.1 produced a clean transition state (single imaginary mode) for every one of their bond-topology-reproducing steps (19, 18, and 17 steps, respectively; no higher-order saddles). UMA-s-1.2 produced a clean transition state for 17 of its 18 such steps, with a higher-order saddle (multiple imaginary modes) on 1RTQ step 1, and Orb-v3-omol for 16 of its 18, with higher-order saddles on 1RTQ step 1 and 4OTA B/step 1. The diagnostic patterns are discussed in Section S6.8.

Table S35: Qualitative reaction reproduction across five MLIP backends under the default pipeline settings (finite-difference Hessian, `--refine-path` off). ✓ = all non-metal bond changes from the input R→P pair reproduced in post-IRC geometry-optimized endpoints with $n_{\text{imag}} = 1$ at the TS; ∼ = expected bond changes reproduced along the IRC, but the located stationary point is a higher-order saddle ($n_{\text{imag}} \geq 2$) rather than a clean first-order transition state; × = other TS: the pipeline located a valid transition state but of a reaction other than the one requested, so the intended bond changes were not reproduced; E = TS-optimization failure: the optimizer converged to a minimum rather than a first-order saddle, so no imaginary mode was available to start the IRC and the run produced no validated TS.

PDB	Model/Step	UMA-s-1.1	UMA-s-1.2	UMA-m-1.1	MACE-OMOL-0	Orb-v3-omol
1AH7	A	×	×	✓	×	×
	B	✓	✓	✓	✓	✓
	Ba	×	×	×	×	✓
1O9S	A (29 atoms)	✓	✓	✓	✓	✓
	B (46 atoms)	✓	✓	✓	✓	✓
	C (72 atoms)	✓	✓	✓	✓	✓
	D (132 atoms)	✓	✓	✓	✓	✓
1PWZ	A (83 atoms)	✓	✓	✓	✓	✓
	B (112 atoms)	✓	✓	✓	✓	✓
	C (161 atoms)	✓	✓	✓	✓	✓
1RTQ	step 1	E	∼	✓	✓	∼
	step 2	✓	✓	✓	✓	✓
	step 3	✓	✓	✓	✓	×
	step 4	✓	✓	✓	✓	✓
2E7Z	A/step 1	✓	✓	✓	✓	✓
	A/step 2	✓	✓	✓	✓	✓
	A/step 3	×	×	×	×	×
	A/step 4	✓	✓	✓	✓	✓
	B	E	×	×	×	×
4OTA	A/step 1	✓	✓	✓	✓	✓
	A/step 2	×	E	E	×	×
	B/step 1	✓	✓	✓	✓	∼
	B/step 2	✓	✓	✓	✓	✓
**Clean ✓ / higher-order saddle ∼** out of 23		17 / 0	17 / 1	**19 / 0**	18 / 0	16 / 2

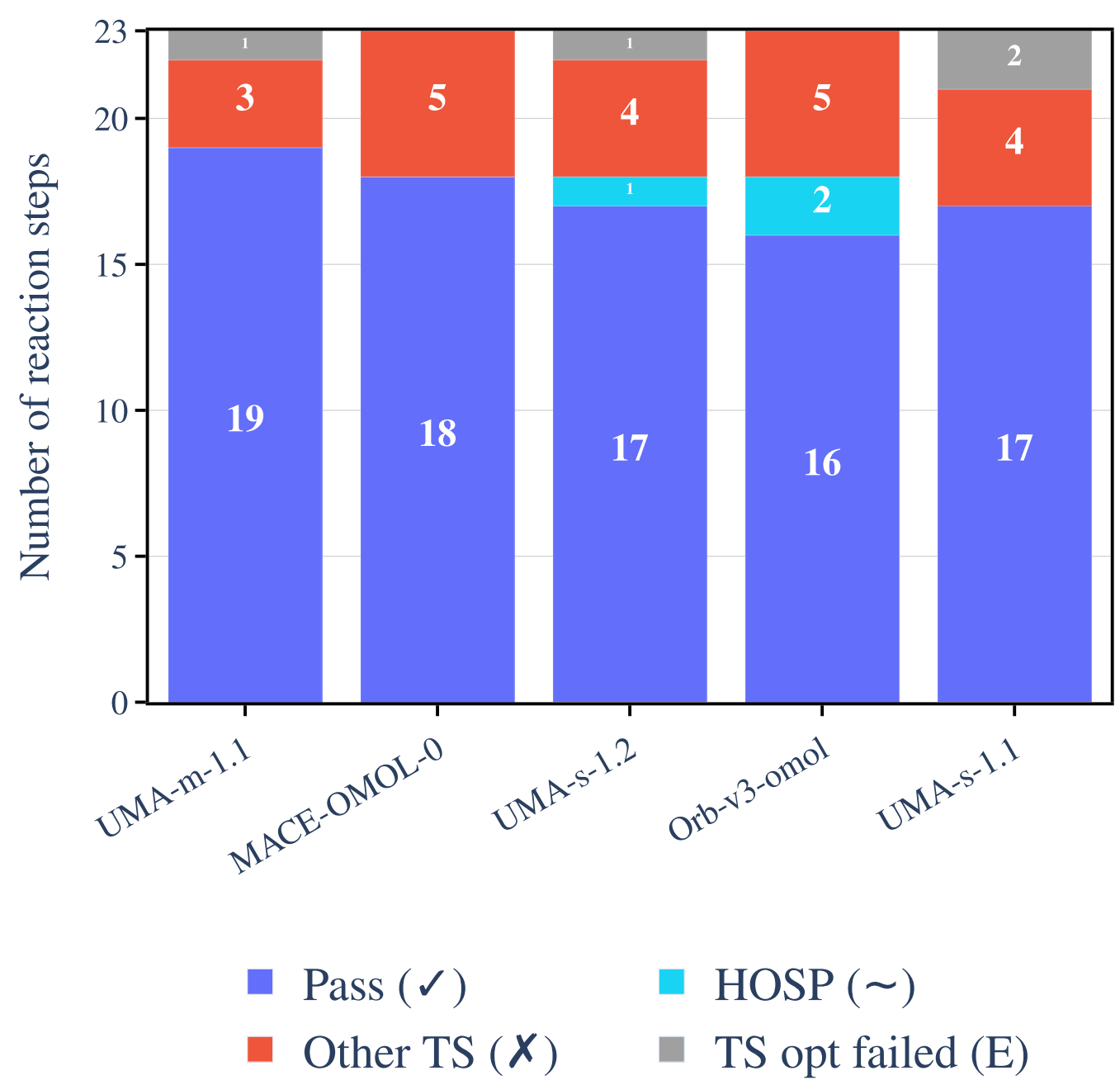

Figure S6: Qualitative reaction reproduction across five MLIP backends under the default pipeline settings. Stacked bars show clean passes (blue, ✓; bond changes reproduced with $n_{\text{imag}} = 1$), higher-order saddles (cyan, ∼; bond changes reproduced with $n_{\text{imag}} \geq 2$; not counted as passes), other TSs (red, ×; a valid saddle of a different reaction), and TS-optimization failures (gray, E), out of 23 reaction steps.

S6.8 Outcome Classification

Bond-change detector.

Bond formation and breaking are resolved by the pairwise distance / scaled covalent-radius rule defined in Section S4.21 (`pdb2reaction.domain.bond_changes.compare_structures`; default scaling $\alpha = 1.20$, margin $m = 0.05$, change gate $\delta = 0.05$).

Non-metal filter.

Before the pass/fail decision, the detected bond changes are filtered to remove any pair that involves a metal center. The metal set used throughout the benchmark is $\{Zn, W, Cu, Fe, Mg, Mn, Co, Ni, Ca\}$. MLIPs routinely show soft, reversible metal–ligand coordination drifts (e.g. a Zn–O_{water} contact oscillating across the threshold) that are usually chemically innocent within the present benchmark—where the reaction coordinate is defined by non-metal bond formations/breakings and the metal coordination sphere fluctuates only as a spectator—so they are excluded from the qualitative judgment.

Reference bond set.

For each reaction step, the expected bond changes are obtained by running the detector on the user-supplied input pair $(R_{\text{in}}, P_{\text{in}})$ – the exact two files passed to `pdb2reaction` via the `-i` flag. These are the bonds the pipeline is required to reproduce in the post-IRC geometry-optimized endpoints $(R_{\text{IRC}}, P_{\text{IRC}})$.

Display symbols.

Let $F_{\text{in}}, B_{\text{in}}$ be the non-metal formed and broken bond sets from the input pair, and $F_{\text{IRC}}, B_{\text{IRC}}$ the analogous sets from the post-IRC geometry-optimized endpoints. Each run is assigned one of:

- ✓ **Clean pass** — all expected bond changes reproduced ($F_{\text{in}} \subseteq F_{\text{IRC}}$ and $B_{\text{in}} \subseteq B_{\text{IRC}}$), with the converged TS carrying exactly one imaginary mode ($n_{\text{imag}} = 1$; modes with $|\tilde{\nu}| < 5$ cm^{-1} are filtered before counting).
- ∼ **Higher-order saddle point (HOSP)** — bond changes reproduced as above, but the converged stationary point carries $n_{\text{imag}} \geq 2$ and is therefore a higher-order saddle, not a clean first-order transition state. It is reported as a diagnostic (the "HOSP" column of main-text Table 2) and is *not* counted as a Pass; the dominant imaginary mode is nevertheless the reactive one and the IRC reproduces the expected bond changes, but we make no claim that the extra imaginary modes would vanish upon reoptimization at a higher level of theory.
- × **Other TS** — the pipeline ran to completion and located a transition state, but a *different* one from the transition state of the requested reaction: the expected bond changes were not reproduced, either not at all or only in part. All 21 × cells under the default condition carry exactly one imaginary mode: the pipeline returns a first-order transition state, but of a different reaction from the one requested.
- E **TS-optimization failure** — no transition state was obtained, so no reaction could be judged at all. In every default-condition E case the optimizer converged to a minimum ($n_{\text{imag}} = 0$) rather than to a first-order saddle, which leaves the IRC with no imaginary mode to follow (Section S6.8).
- M **Multi-segment split** — the recursive path search split the step into several segments and left no validated transition state for the requested step. This outcome arises only under the **rp** condition (`--refine-path`), where it occurs twice; it is zero in every other condition.

The labels are mutually exclusive and answer successively finer questions: was a transition state found at all (E vs. the rest), does it describe the intended reaction (× vs. the rest), and is it a clean first-order saddle (∼ vs. ✓). Extra, non-expected bond changes do not demote a label; the criterion is subset reproduction. The n_{imag} cross-tabulation is given in Table S36.

Table S36: Median and maximum of the per-TS imaginary-mode count n_{imag} across the default-condition cases that reproduce the bond topology. The N column gives that count (out of 23 reaction steps; its clean subset is the "Pass" column and its ∼ subset the "HOSP" column of main-text Table 2) so the median/max can be read with the right sample-size context. `pdb2reaction` filters out modes with $|\tilde{\nu}| < 5$ cm^{-1} before counting.

Backend	N / 23	median n_{imag}	max n_{imag}
MACE-OMOL-0	18 / 23	1	1
UMA-s-1.1	17 / 23	1	1
UMA-s-1.2	18 / 23	1	2
UMA-m-1.1	19 / 23	1	1
Orb-v3-omol	18 / 23	1	2

Observed non-pass outcomes.

The 25 default-condition runs labeled $\times$ or E fall into two categories. In 21 cases, the pipeline located a genuine first-order TS for a different reaction ($\times$, Other TS), with some or all expected bond changes absent. In the remaining four, TS optimization converged to a minimum (E; $n_{\text{imag}} = 0$), leaving no imaginary mode for the IRC. The three HOSP cases reproduce the expected bond changes and are retained separately as diagnostics; they are not included in these two failure categories and are not counted as clean passes.

(i) TS-optimization failure: every default-condition E case converged to a minimum instead of a saddle point.

The four default-condition E cases (1RTQ/step 1 and 2E7Z B on UMA-s-1.1; 4OTA A/step 2 on UMA-s-1.2 and UMA-m-1.1; Table S35) all fail through one mechanism. In each case the TS optimization converges, but to a stationary point with *zero* imaginary modes—the lowest wavenumber is $+5$ to $+28$ cm^{-1} in the four runs—that is, to a shallow minimum rather than a first-order saddle. The subsequent IRC step then cannot start, because its initial displacement needs an imaginary mode to define the descent direction; the run therefore ends without a validated TS and without a barrier. On each of the three steps involved the remaining backends do converge to a saddle point (Table S35), so the E outcome reflects that particular optimization rather than an intrinsic absence of a saddle point on the step; whether the requested reaction is then reproduced is a separate question (paragraph (ii) below). Re-running the TS search from a different initial guess is the practical remedy.

(ii) Steps failing in every scored backend–condition cell: the 2E7Z tungsten active site.

2E7Z A/step 3 (W-dependent acetylene hydratase) and the single-step model 2E7Z B are the only reaction steps that fail in every scored backend–condition cell (Section S6.9), covering the recursive-path-search, TS-only, and analytical-Hessian conditions—with the single exception that Orb-v3-omol is not scored under the analytical-Hessian condition at all (paragraph (iii) below), so no outcome exists for that cell. The failures do not share a single symptom: on 2E7Z B, UMA-s-1.1 returns a TS-optimization failure (E; the optimizer converges to a minimum, paragraph (i) above), while the other four backends return an other-TS result ($\times$). Step 4 of the same system, by contrast, is passed by all five backends under the default condition. 4OTA A/step 2, the third step that no backend passes under the default condition, is not in this category: it is recovered by all five backends under the Direct-MaxFlux driver and by four of five with the recursive path-search (Table S37, Table S39).

(iii) Orb-v3-omol is not scored under the analytical-Hessian condition.

Under the analytical-Hessian condition (`--hessian-calc-mode Analytical`), Orb-v3-omol could not be evaluated because of an incompatibility in the execution environment. It is therefore reported as "–" and excluded from the **ana** statistics rather than scored as 23 pipeline failures. For the other four backends, the analytical-Hessian condition yielded 17–18 bond-topology-reproducing steps, compared with 17–19 under **default** (Table S37), while converting one or two clean passes per backend into higher-order saddles. On this benchmark, the analytical-Hessian condition therefore affected the TS spectrum more than reaction-finding coverage.

Effect of `--refine-path`.

Table S37 compares the release default (single-pass path optimization) against **rp** (`--refine-path`, the recursive path-search enabled as an opt-in). Enabling recursion has no consistent effect on bond-topology-reproducing coverage: it changes each backend's count by at most one step. Coverage falls for MACE-OMOL-0 ($18 \to 17$) and UMA-m-1.1 ($19 \to 18$); both steps that move in the other direction do so because recursion splits them into several segments—the expected behavior when it is applied to a cluster scored one elementary step at a time, each with a single known reactive coordinate, rather than to a genuinely multistep mechanism such as BezA.

Literature baseline convention.

The reported B3LYP barrier used as the external-reference baseline for each step is shipped in the SI data bundle as the `lit_barrier_kcal` column of `manifest.csv` (one value per `pdb/model_step`, identical across backends and conditions; the per-system citations are given in the subsubsections above). For every reaction step we identify the two stationary points that correspond to the left and right `-i` input files used by `pdb2reaction`, label the left one as baseline 0 kcal mol^{-1}, and define

$$\Delta E^{\ddagger}_{\text{lit}} \;=\; \max_{k\in\text{middle}} \big[E_k - E_{\text{left}}\big], \qquad \Delta E_{\text{rxn,lit}} \;=\; E_{\text{right}} - E_{\text{left}},$$

where "middle" ranges over all stationary points strictly between the two inputs along the literature path. In single-step models (*1AH7/A, B, Ba*; all *1O9S*; all *1PWZ*) the inputs are R and P and the convention reduces to the conventional $\Delta E^{\ddagger}$ and ΔE_{rxn}. In stepwise mechanisms (*1RTQ*, *2E7Z/A*, *4OTA/A, B*) each step is scored against the literature value for its own left/right pair, so the table remains step-aligned with what the pipeline actually optimizes.

S6.9 Per-Condition Pass Counts

The main-text pass counts (Table 2) are for the *default* condition (single-pass path optimization with the recursive path-search `--refine-path` left off; finite-difference Hessian, `--opt-mode-post hess`). Table S37 gives the corresponding counts under all four pipeline conditions we tested:

- **rp**: `--refine-path`, so the recursive path-search is enabled (single-pass is the default, shown in the **default** column);
- **tsonly**: the TS optimizer is started from a user-provided TS guess (bypassing the path-search);
- **ana**: `--hessian-calc-mode Analytical`, otherwise identical to **default**.

Step 3 of 2E7Z A and the single-model system 2E7Z B fail on every backend under all four conditions tested (Section S6.8), so both are absent from every column's count.

Table S37: Bond-topology-reproducing counts (clean ✓ / higher-order saddle, HOSP, ∼; out of 23 steps) per backend and condition. **default**: finite-difference Hessian and single-pass path optimization; **rp**: recursive path-search; **tsonly**: user-supplied TS guess without path-search; **ana**: analytical Hessian, otherwise default. Bold marks the highest clean-pass count in each column (HOSP excluded); ties are bolded. [a]Orb-v3-omol was not evaluated under **ana** because of an incompatibility in the execution environment; "–" denotes no outcome and is excluded from the corresponding statistics.

Backend	default<br>Pass / HOSP	rp<br>Pass / HOSP	tsonly<br>Pass / HOSP	ana<br>Pass / HOSP
MACE-OMOL-0	18 / 0	17 / 0	7 / 0	16 / 2
UMA-s-1.1	17 / 0	17 / 1	6 / 0	16 / 1
UMA-s-1.2	17 / 1	**18 / 0**	7 / 0	16 / 2
UMA-m-1.1	**19 / 0**	**18 / 0**	**9 / 0**	**18 / 0**
Orb-v3-omol	16 / 2	17 / 2	7 / 0	–[a]

E and M cases are rare throughout: 4 E under **default** (paragraph (i)), 1 E and 2 M (M: a recursive multi-segment split left no validated TS for the requested step) under **rp**, none under **tsonly**, and 1 E under **ana** among the four backends for which that condition runs.

TS-only input: the path search is what ties the saddle to the requested reaction.

Bypassing the path search costs more coverage than any other condition: clean passes fall to 6–9 of 23 (Table S37). The TS optimization is not what fails. Seeded with the published TS geometry, it returns a single imaginary mode in 110 of the 115 cells—marginally more often than under **default** (108)—so the saddle search itself succeeds. What collapses is the reaction check: 73 cells reproduce only part of the expected bond-change set, against 12 under **default**. Given a saddle but no reactant and product to connect, the IRC descends into whichever basins that saddle happens to join. The path search is therefore not an optional preliminary to TS optimization; it is what ties the located saddle to the intended R $\rightarrow$ P transformation.

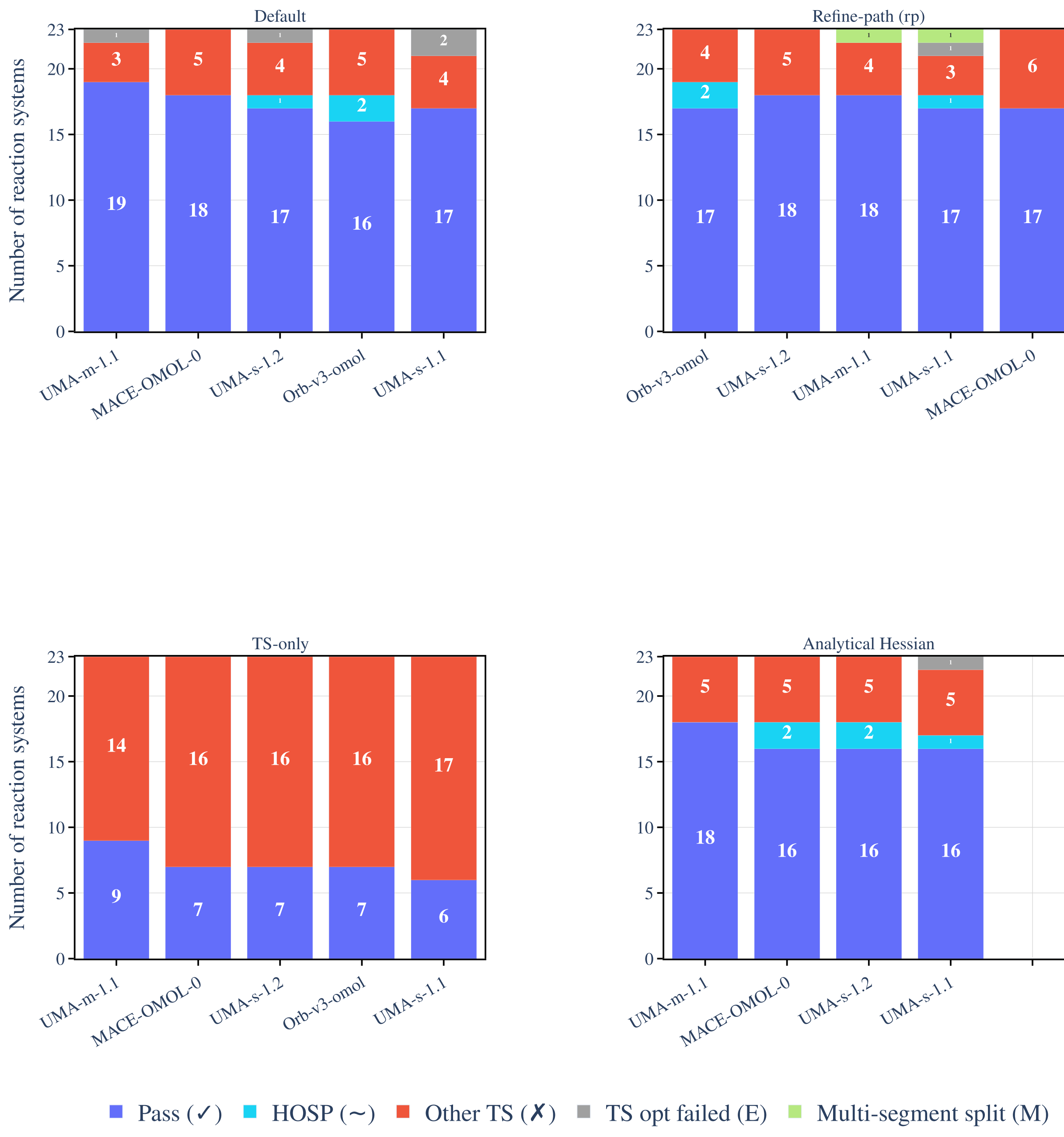

Figure S7: Per-condition pass/fail breakdown across four pipeline conditions. Segments denote clean pass (✓; $n_{\text{imag}} = 1$), higher-order saddle (∼; $n_{\text{imag}} \geq 2$; not counted as a pass), Other TS (×; a different first-order TS from the requested reaction), and E (TS optimization converged to a minimum, leaving no imaginary mode for IRC). The **rp** panel also has M (multi-segment split), where recursive path splitting left no validated TS for the requested step; M is zero elsewhere. Conditions: **default** (finite-difference Hessian, single-pass path optimization), **rp** (`--refine-path`), **tsonly** (user-supplied TS guess, no path-search), and **ana** (analytical Hessian, otherwise default). Orb-v3-omol was not evaluated under **ana** because of an incompatibility in the execution environment; the blank slot denotes no outcome and is excluded from the corresponding statistics. Each non-**ana** bar sums to 23 steps.

S6.10 Precision, Determinism, and MEP-Driver Ablations

Beyond the four pipeline-mode conditions above, we ran the 23-step benchmark under five further conditions that isolate the effect of numerical precision, run-to-run determinism, and the MEP driver, independent of the recursive path-search: **fp32-det** (single precision, deterministic kernels), **fp64-nd** and **fp32-nd** (double/single precision without the determinism flag, 2–5 replicates per cell), **flatten** (`--flatten`, the post-optimization imaginary-mode-flattening loop), and **dmf** (Direct-MaxFlux in place of the default GSM path driver; Section S7). Table S38 gives the resulting Pass/HOSP counts for the first four; the **dmf** counts are given separately in Table S39. Figure S8 shows the same outcomes as a per-condition stacked breakdown. Reproducibility (median $|\sigma|$ of the MLIP $\Delta E^{\ddagger}$ across 2–5 replicates, over all replicated cells) is 0.06 kcal mol^{-1} for fp64-nd and 0.37 kcal mol^{-1} for fp32-nd, so the median fp64 replicate spread is $\sim$6$\times$ smaller than the fp32 one even without the determinism flag; a minority of cells at both precisions show much larger replicate-to-replicate spread.

Table S38: Effect of numerical precision, determinism, and the MEP driver on bond-topology-reproducing counts (clean ✓ / higher-order saddle $\sim$, out of 23), all under single-pass path optimization (no recursive path-search). **default** = fp64, deterministic kernels (same as Table S37). **fp64-nd/fp32-nd** = non-deterministic kernels, representative replicate. **flatten** = `--flatten`. The **dmf** condition is not a column here; its counts are given separately in Table S39. Bold entries mark the highest *clean*-pass count in each column (HOSP excluded from this ranking); ties are all bolded.

Backend	default (fp64-det)	fp64-nd	fp32-det	fp32-nd	flatten
MACE-OMOL-0	18 / 0	16 / 2	5 / 13	5 / 13	18 / 0
UMA-s-1.1	17 / 0	17 / 0	15 / 2	16 / 1	17 / 0
UMA-s-1.2	17 / 1	17 / 1	17 / 1	**18 / 0**	18 / 0
UMA-m-1.1	**19 / 0**	**19 / 0**	**19 / 0**	**18 / 1**	**19 / 0**
Orb-v3-omol	16 / 2	16 / 1	1 / 12	1 / 11	17 / 0

Single precision drives higher-order saddles, overwhelmingly for MACE-OMOL-0 and Orb-v3-omol.

The clean/HOSP split under fp32 (**fp32-det** and **fp32-nd**) is substantially worse than fp64 for two of the five backends: MACE-OMOL-0 drops from 18 clean (0 HOSP) at fp64 to 5 clean (13 HOSP), and Orb-v3-omol from 16 clean (2 HOSP) to 1 clean (11–12 HOSP). The bond-topology-reproducing total (✓$+\sim$) is unchanged for MACE-OMOL-0 (18→18) but reduced for Orb-v3-omol (18→13 at fp32-det, 18→12 at fp32-nd); for MACE-OMOL-0 the entire effect is a change in TS spectrum quality rather than whether the reaction is found, while for Orb-v3-omol part of the effect (5 of 18 cases at fp32-det) is a loss of reaction-finding itself. The three UMA variants are far less sensitive: UMA-s-1.1 converts two clean passes into higher-order saddles at fp32-det (17 clean / 0 HOSP → 15 / 2), while UMA-s-1.2 and UMA-m-1.1 are unchanged. Their clean-pass counts therefore stay within 2 of the fp64-det values, against drops of 13 and 15 for MACE-OMOL-0 and Orb-v3-omol. This is consistent with the per-backend `--precision` default documented in Section S2.2 (UMA `fp32`; Orb and MACE `fp64`).

Determinism (nd vs. det) has little effect on the pass/HOSP split itself.

Comparing **fp64-nd** to **default** and **fp32-nd** to **fp32-det**, the representative-replicate Pass/HOSP counts agree to within 1 for four of the five backends; the exception is MACE-OMOL-0 at fp64, whose representative non-deterministic replicate converts two of its clean transition states into higher-order saddles (16 clean / 2 HOSP against 18 clean / 0 HOSP at default), leaving the bond-topology-reproducing total unchanged at 18. The qualitative precision effect described above is therefore not an artifact of turning the determinism flag on or off. Where determinism matters is per-replicate numerical reproducibility of the barrier value itself, not the qualitative outcome. Across the 113 replicated cells at fp64 the median barrier standard deviation is 0.06 kcal mol^{-1} (0.37 at fp32), but the distribution is heavy-tailed: 21% of cells exceed 1 kcal mol^{-1} and the 90th percentile is 5.3 kcal mol^{-1}, the tail arising when a replicate re-converges to a different stationary point. The median therefore describes the typical step rather than bounding the run-to-run variation of any single one.

Flattening extra imaginary modes recovers clean TSs at essentially no cost in coverage.

flatten (`--flatten`) does what it is designed to do: every default-condition higher-order saddle is resolved, and the flatten column contains no $\sim$ cells at all. The two backends that had HOSPs to flatten both gain: UMA-s-1.2 goes from 17 clean (1 HOSP) to 18 clean, and Orb-v3-omol from 16 clean (2 HOSP) to 17 clean. The three backends that already had no higher-order saddle are unchanged (MACE-OMOL-0 18, UMA-s-1.1 17, UMA-m-1.1 19). The bond-topology-reproducing total (✓$+\sim$) is preserved for four of the five backends and falls by one for Orb-v3-omol (18→17), whose second HOSP (1RTQ/step 1) flattens onto a different transition state rather than onto the requested one. Flattening therefore converts $\sim$ to ✓ cell-by-cell without turning a $\times$ or an E into a pass, and its only risk is that a higher-order saddle sitting close to a competing reaction channel may relax into that channel instead.

Under **dmf** the bond-topology-reproducing total (✓$+\sim$) is within 2 of **default** for every backend (MACE 18→19, UMA-s-1.1 17→19, UMA-s-1.2 18→19, UMA-m-1.1 19→20, Orb 18→17), four of the five gaining one to two cases. Because the **dmf** condition also raises `--max-nodes` to 50 against the default 20, this bounds the combined driver-and-resolution effect rather than the driver alone. DMF does raise the HOSP count for MACE-OMOL-0 and UMA-s-1.2 (0→3 and 1→3), a milder

Table S39: Direct-MaxFlux (dmf) versus the default GSM path driver (fp64, deterministic, single-pass in both; the dmf condition additionally sets `--max-nodes 50`, against the default 20 for GSM); entries are clean-pass (✓) / higher-order-saddle (∼) counts out of 23, as in Table S38. See Section S7 for the wall-clock-scaling motivation for DMF on large clusters; this table checks whether the driver choice affects reaction fidelity on the 23-step benchmark. Bold entries mark the highest *clean*-pass count in each column (HOSP excluded from this ranking); ties are all bolded.

Backend	default (GSM)	dmf
MACE-OMOL-0	18 / 0	16 / 3
UMA-s-1.1	17 / 0	17 / 2
UMA-s-1.2	17 / 1	16 / 3
UMA-m-1.1	**19 / 0**	**19 / 1**
Orb-v3-omol	16 / 2	16 / 1

form of the TS-spectrum effect seen at fp32. DMF's advantage is wall-clock scaling on large clusters (Table S45) rather than reaction finding on this small-cluster set.

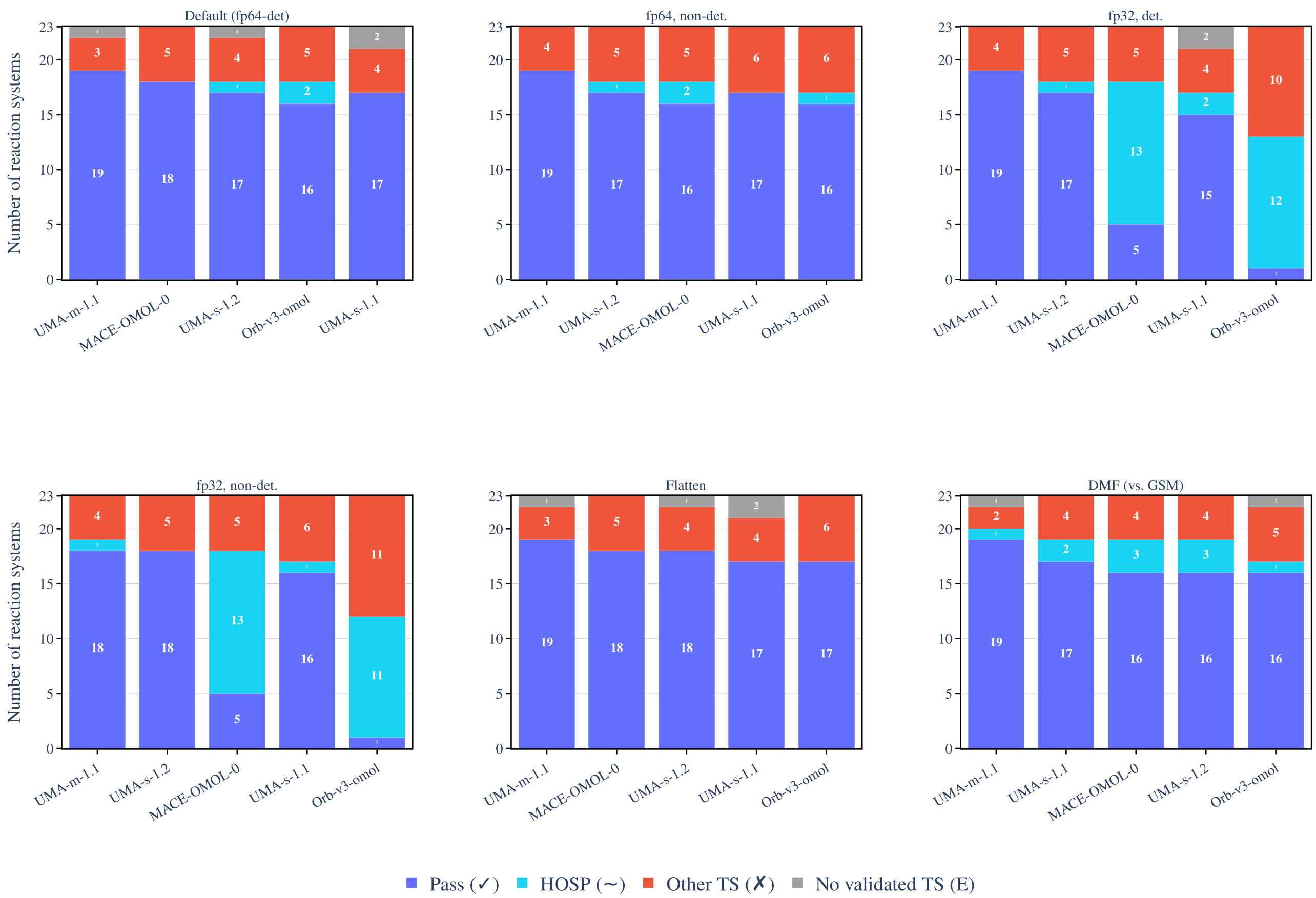

Figure S8: Per-condition pass/fail breakdown for the precision, determinism, and MEP-driver ablations (Tables S38 and S39), using the same four-symbol ontology as Figure S7. Under fp32, MACE-OMOL-0 and Orb-v3-omol shift many bond-topology-reproducing cases from clean passes to higher-order saddles; **flatten** removes all higher-order saddles, and **DMF** remains within 2 bond-topology-reproducing cases of **default** for every backend.

S6.11 MAE Summary and Literature-Reference Deviations

Table S40 collects the mean absolute deviations (MAEs) of the MLIP-only and DFT//MLIP activation energies from the reported literature B3LYP/6-311+G(2d,2p) reference for the default-condition bond-topology-reproducing cases. All are cross-method deviations in the sense set out at the start of this section. The MLIP $\Delta E^\ddagger$ column reproduces the main-text values (Table 2); the $\Delta G^\ddagger$ column gives the corresponding QRRHO free-energy deviation, reported descriptively rather than as a matched 298 K accuracy metric because the literature set does not provide a uniform finite-temperature Gibbs reference; and the DFT//MLIP column is the ωB97M-V/def2-SVP single-point re-scoring of the MLIP-optimized R/TS/P geometries (see Section S6.13). Each backend's MAE is taken over the steps it reproduces, so the subsets differ slightly ($N = 17$–19). The common-subset analysis below removes that difference by restricting to the $N = 16$ cases reproduced by all five backends.

Table S40: Mean absolute deviation (MAE) of activation energies from reported literature B3LYP/6-311+G(2d,2p) values, per backend. MLIP columns report electronic $\Delta E^\ddagger$ and QRRHO $\Delta G^\ddagger$ deviations. Because the ZPE-corrected literature values and electronic MLIP/DFT//MLIP energies use different potential-energy surfaces, geometries, and vibrational conventions, MAE is a cross-method diagnostic rather than a like-for-like model error. N is the number of bond-topology-reproducing cases (clean Pass plus HOSP); $\pm$ is the standard error of the mean. DFT//MLIP values are reported for the 90 bond-topology-reproducing default-condition cases (87 clean passes and 3 higher-order saddles) and after excluding barriers < 0 or > 150 kcal mol^{-1} ("excl. non-phys."), with retained N in parentheses. All values are in kcal mol^{-1}.

					DFT//MLIP $\Delta E^\ddagger$	
Backend	N	Pass / HOSP	MLIP $\Delta E^\ddagger$	MLIP $\Delta G^\ddagger$	all	excl. non-phys.
MACE-OMOL-0	18	18 / 0	5.6 ± 0.9	4.5	5.8 ± 1.2	5.8 ± 1.2 ($N = 18$)
UMA-s-1.1	17	17 / 0	5.3 ± 0.7	4.3	17.7 ± 6.6	8.3 ± 3.2 ($N = 13$)
UMA-s-1.2	18	17 / 1	5.5 ± 0.6	4.2	18.2 ± 4.9	13.0 ± 5.7 ($N = 11$)
UMA-m-1.1	19	19 / 0	5.1 ± 0.5	3.9	15.1 ± 5.9	9.6 ± 2.6 ($N = 18$)
Orb-v3-omol	18	16 / 2	4.5 ± 0.6	3.8	5.1 ± 1.2	5.1 ± 1.2 ($N = 18$)

Signed deviation and spread.

The five mean absolute deviations for $\Delta E^\ddagger$ span 4.5–5.6 kcal mol^{-1} over similar coverage ($N = 17$–19; Table S40). At the resolution of this benchmark the five backends are not distinguishable: the spread across them (1.1 kcal mol^{-1}) is comparable to the standard errors of the individual mean absolute deviations (0.5–0.9 kcal mol^{-1} over 17–19 paired steps). We therefore report the deviations per backend without ranking them. Deviation spreads are likewise similar (IQR 3.0–3.7 kcal mol^{-1} across the five backends; Figure S10). In Figure S9 most points lie above the $y = x$ line: the MLIP barriers are systematically higher than the literature values. The mean signed deviation (MLIP $-$ Lit.) is $+4.56$ kcal mol^{-1} for MACE-OMOL-0, $+4.04$ for UMA-s-1.1, $+3.78$ for UMA-s-1.2, $+3.45$ for UMA-m-1.1, and $+3.63$ for Orb-v3-omol; all five backends show a positive offset of roughly 3.5–4.6 kcal mol^{-1} under this cross-method convention, which cannot be assigned solely to MLIP model error. Subtracting each backend's mean signed deviation reduces its MAE from 4.5–5.6 to 2.8–3.7 kcal mol^{-1} (3.3 kcal mol^{-1} pooled), but removes only 39–51% of the mean-square deviation, and the bias is not a single constant across the set: per-enzyme mean signed deviations range from -1.2 kcal mol^{-1} (1AH7) to $+6.7$ (1O9S), and the extreme single backend–enzyme cells span -2.7 (UMA-m-1.1 on 1AH7) to $+8.1$ (MACE-OMOL-0 on 4OTA). The barriers do track the references across the set—Pearson $r = 0.85$–0.91 per backend (Spearman $\rho = 0.77$–0.90; pooled over all 90 cases, $r = 0.86$ and $\rho = 0.81$)—so the ordering of steps is reproduced better than their absolute values; note that the pooled figure repeats five correlated backends over 16 chemically distinct transformations. Per-step reaction energies (ΔE_{rxn}) show a different backend ordering (Figures S11 and S12): UMA-s-1.1 has the lowest MAE$_{\text{rxn}}$ (4.4 kcal mol^{-1}), followed by UMA-s-1.2 and UMA-m-1.1 (4.7 each), Orb-v3-omol (4.9), and MACE-OMOL-0 (6.1), underscoring that barrier and reaction-energy deviations are separate diagnostics that need not favor the same backend.

Common-subset MAE.

Restricted to the $N = 16$ bond-topology-reproducing cases shared by all five backends, the $\Delta E^\ddagger$ MAEs are 5.94 (MACE-OMOL-0), 5.48 (UMA-s-1.1), 5.76 (UMA-s-1.2), 5.44 (UMA-m-1.1), and 4.76 kcal mol^{-1} (Orb-v3-omol)—a 4.8–5.9 kcal mol^{-1} band, essentially unchanged from the 4.5–5.6 kcal mol^{-1} of the full set (Table S40). The small differences between backends are therefore not an artifact of the differing subsets each backend passes.

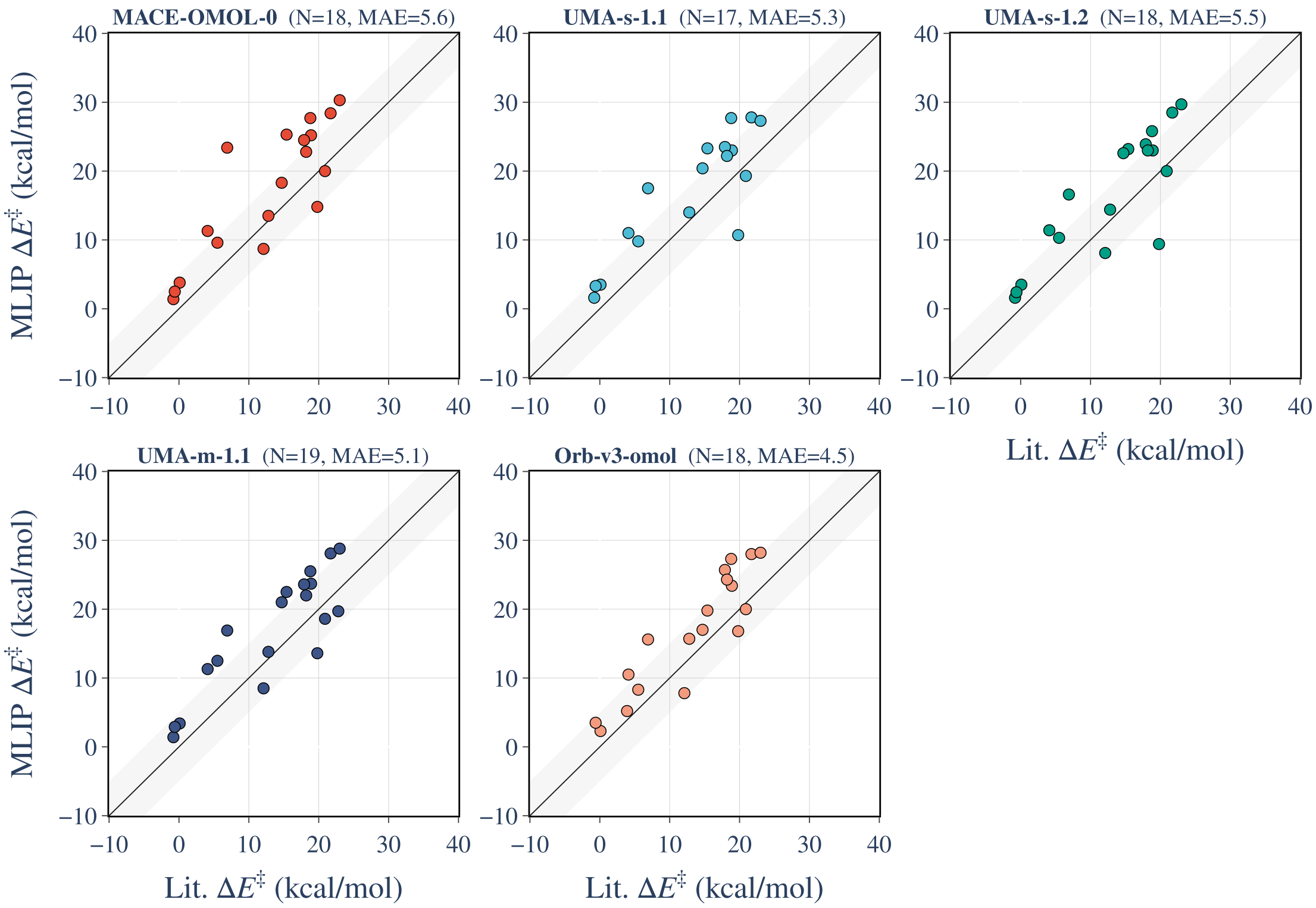

Figure S9: Correlation between MLIP and literature B3LYP activation barriers ($\Delta E^{\ddagger}$) for all bond-topology-reproducing cases. Each panel corresponds to one MLIP backend. Subplot titles report N and MAE per backend. The solid line is $y = x$; the shaded band indicates $|\text{MLIP} - \text{Lit.}| \leq 5\ \text{kcal mol}^{-1}$.

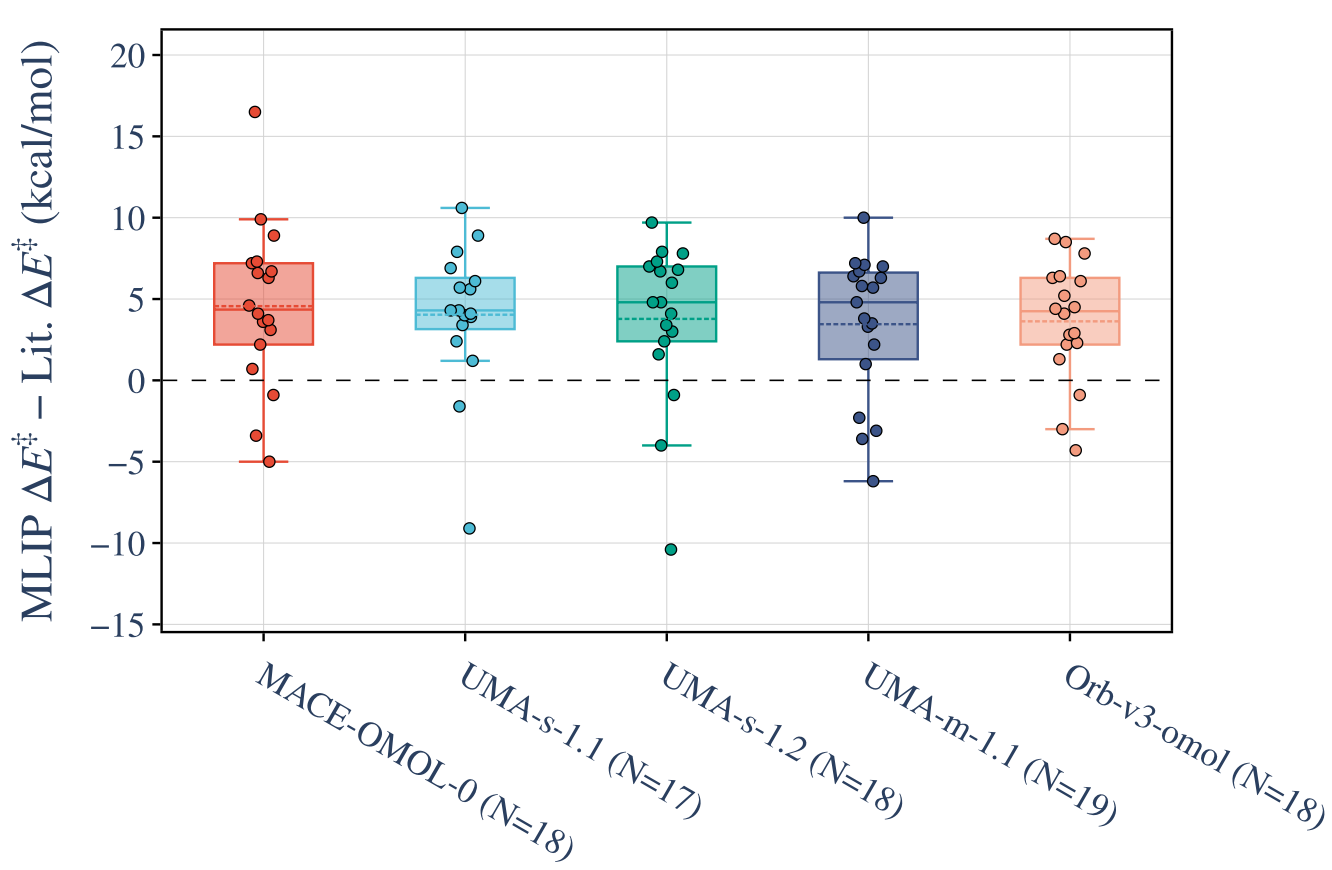

Figure S10: Distribution of barrier deviations (MLIP $\Delta E^{\ddagger}$ − Lit. $\Delta E^{\ddagger}$) per backend under the cross-method convention defined above. Box = IQR with median (solid) and mean (dashed) interior lines; individual points overlaid; the plot-wide dashed line at $y = 0$ = zero deviation.

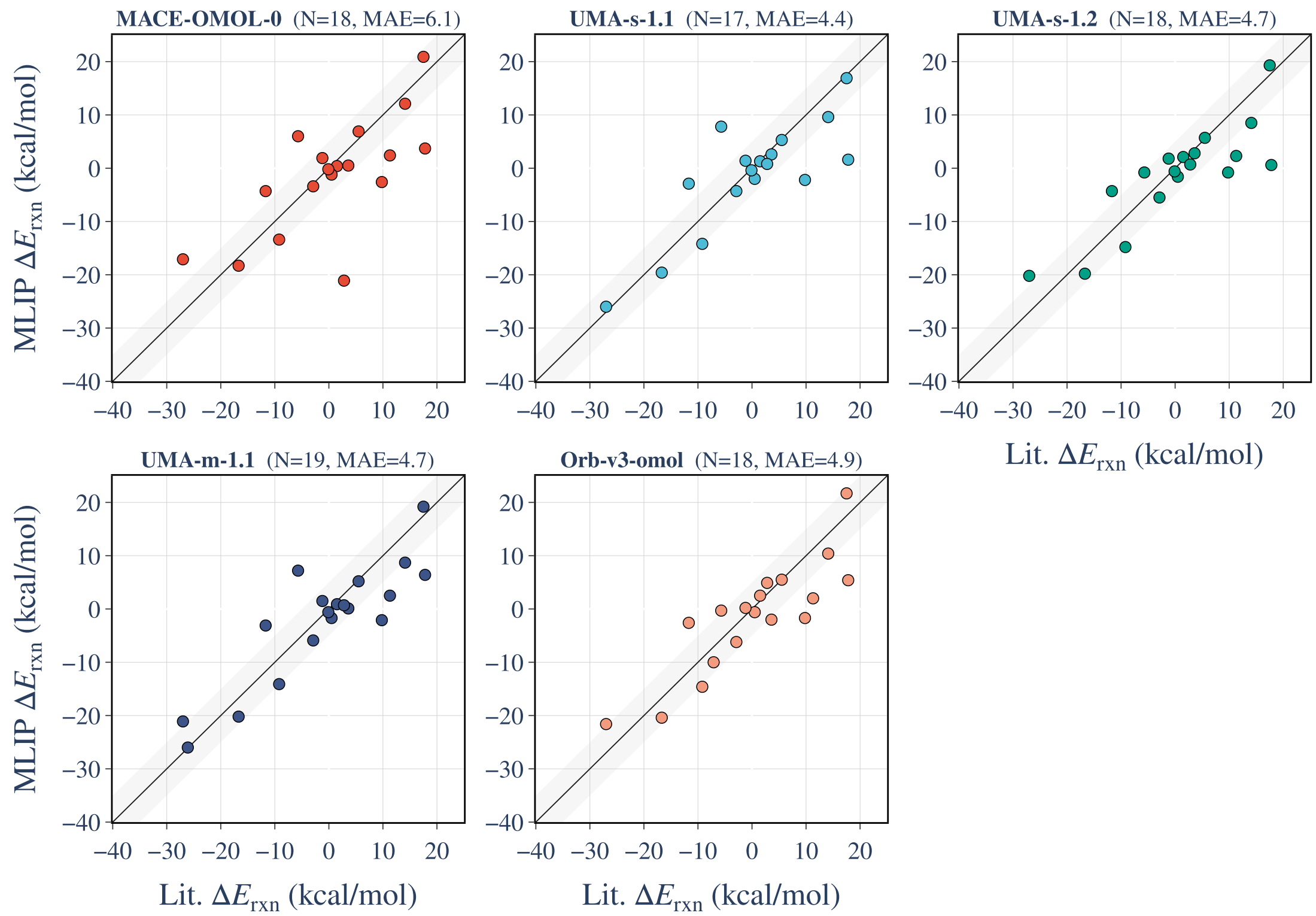

Figure S11: Correlation between MLIP and literature B3LYP reaction energies (ΔE_{rxn}) for all bond-topology-reproducing cases. Panels and conventions as in Figure S9.

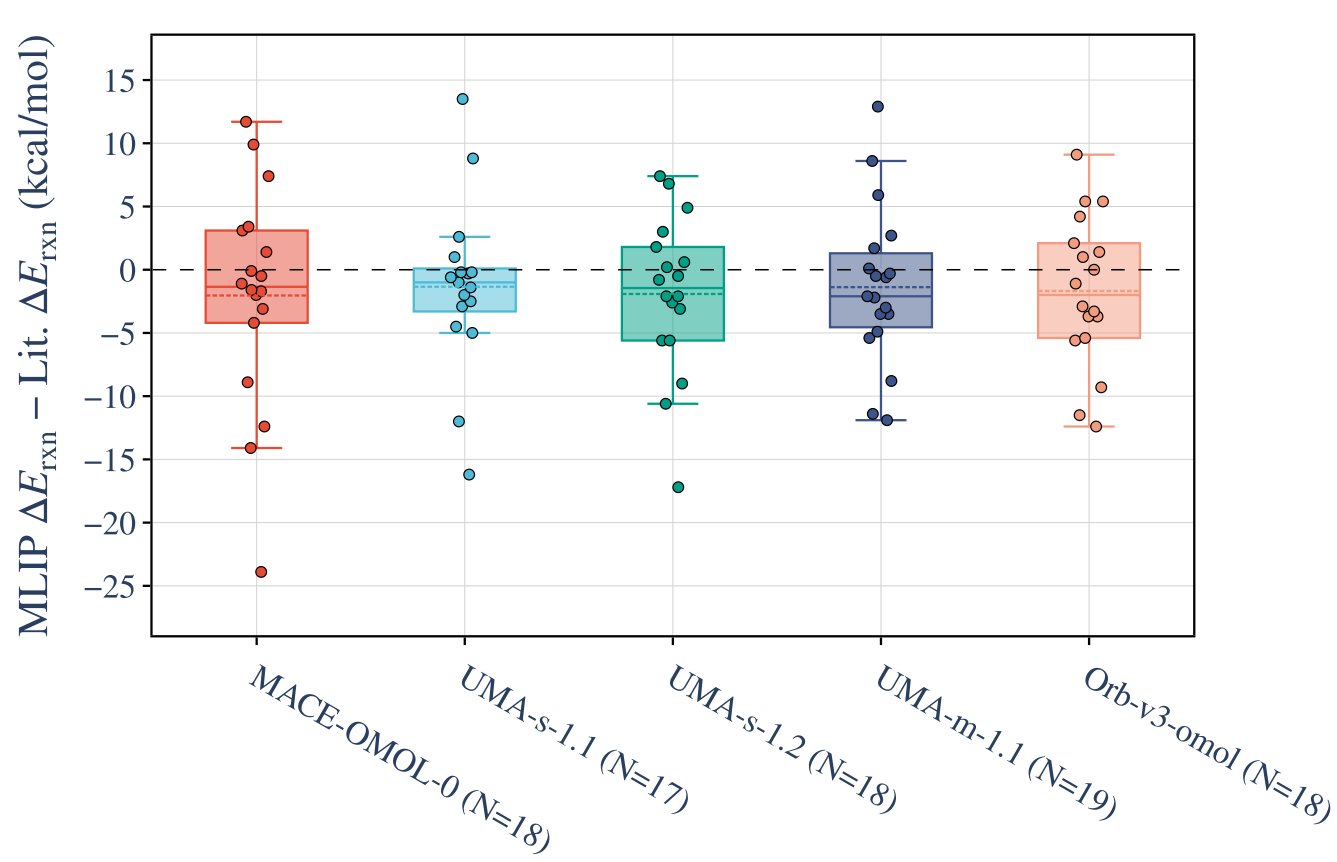

Figure S12: Distribution of reaction-energy deviations (MLIP ΔE_{rxn} − Lit. ΔE_{rxn}) by backend. Conventions as in Figure S10.

S6.12 Per-Step Barrier and Reaction-Energy Deviations

Table S41 lists individual barrier and reaction-energy deviations for every bond-topology-reproducing case (Pass plus HOSP; 90 rows: 87 clean passes and 3 higher-order saddles) across all five backends. Finite-temperature QRRHO Gibbs values are omitted because the literature set does not provide a uniform 298 K Gibbs free-energy reference.

Table S41: Per-step barrier and reaction-energy deviations for all bond-topology-reproducing cases. "Err." is MLIP − literature, or DFT//MLIP − literature for the DFT//MLIP columns. DFT//MLIP denotes ωB97M-V/def2-SVP single-point energies on MLIP-optimized R/TS/P geometries and is reported for the 90 bond-topology-reproducing cases (87 clean passes and 3 higher-order saddles). All values in kcal mol^{-1}.

			Activation barrier $\Delta E^{\ddagger}$					Reaction energy ΔE_{rxn}				
PDB	Model	Backend	Lit.	MLIP	Err.	DFT//MLIP	Err.	Lit.	MLIP	Err.	DFT//MLIP	Err.
1AH7	A	UMA-m-1.1	22.8	19.7	-3.1	6.2	-16.6	-26.1	-26.0	+0.1	-30.5	-4.4
1AH7	B	UMA-s-1.1	20.9	19.3	-1.6	13.5	-7.4	1.5	1.3	-0.2	-0.5	-2.0
		UMA-s-1.2	20.9	20.0	-0.9	11.8	-9.1	1.5	2.1	+0.6	5.7	+4.2
		UMA-m-1.1	20.9	18.6	-2.3	14.5	-6.4	1.5	0.9	-0.6	0.8	-0.7
		MACE-OMOL-0	20.9	20.0	-0.9	12.4	-8.5	1.5	0.4	-1.1	1.4	-0.1
		Orb-v3-omol	20.9	20.0	-0.9	1.5	-19.4	1.5	2.5	+1.0	1.5	-0.0
1AH7	Ba	Orb-v3-omol	3.9	5.2	+1.3	0.5	-3.4	-7.1	-10.0	-2.9	-0.1	+7.0
1O9S	A	UMA-s-1.1	18.8	27.7	+8.9	23.4	+4.6	-2.9	-4.3	-1.4	-9.5	-6.6
		UMA-s-1.2	18.8	25.8	+7.0	22.8	+4.0	-2.9	-5.5	-2.6	0.4	+3.3
		UMA-m-1.1	18.8	25.5	+6.7	20.7	+1.9	-2.9	-5.9	-3.0	-11.7	-8.8
		MACE-OMOL-0	18.8	27.7	+8.9	23.9	+5.1	-2.9	-3.4	-0.5	-9.0	-6.1
		Orb-v3-omol	18.8	27.3	+8.5	22.2	+3.4	-2.9	-6.2	-3.3	-11.0	-8.1
1O9S	B	UMA-s-1.1	21.7	27.8	+6.1	-51.5	-73.2	0.5	-2.0	-2.5	40.2	+39.7
		UMA-s-1.2	21.7	28.5	+6.8	37.9	+16.2	0.5	-1.6	-2.1	-5.8	-6.3
		UMA-m-1.1	21.7	28.1	+6.4	1.9	-19.8	0.5	-1.7	-2.2	-0.1	-0.6
		MACE-OMOL-0	21.7	28.4	+6.7	24.1	+2.4	0.5	-1.2	-1.7	-6.5	-7.0
		Orb-v3-omol	21.7	28.0	+6.3	23.7	+2.0	0.5	-0.6	-1.1	-7.9	-8.4
1O9S	C	UMA-s-1.1	15.4	23.3	+7.9	17.7	+2.3	-16.7	-19.6	-2.9	-27.4	-10.7
		UMA-s-1.2	15.4	23.2	+7.8	33.3	+17.9	-16.7	-19.8	-3.1	0.1	+16.8
		UMA-m-1.1	15.4	22.5	+7.1	17.6	+2.2	-16.7	-20.2	-3.5	-27.5	-10.8
		MACE-OMOL-0	15.4	25.3	+9.9	19.4	+4.0	-16.7	-18.3	-1.6	-25.7	-9.0
		Orb-v3-omol	15.4	19.8	+4.4	16.6	+1.2	-16.7	-20.4	-3.7	-28.5	-11.8
1O9S	D	UMA-s-1.1	18.9	23.0	+4.1	-74.9	-93.8	-9.2	-14.2	-5.0	-81.6	-72.4
		UMA-s-1.2	18.9	23.0	+4.1	-47.8	-66.7	-9.2	-14.8	-5.6	-65.6	-56.4
		UMA-m-1.1	18.9	23.7	+4.8	17.8	-1.1	-9.2	-14.1	-4.9	-21.8	-12.6
		MACE-OMOL-0	18.9	25.2	+6.3	19.3	+0.4	-9.2	-13.4	-4.2	-20.9	-11.7
		Orb-v3-omol	18.9	23.4	+4.5	18.8	-0.1	-9.2	-14.6	-5.4	-22.5	-13.3
1PWZ	A	UMA-s-1.1	23.0	27.3	+4.3	29.6	+6.6	17.5	16.9	-0.6	22.5	+5.0
		UMA-s-1.2	23.0	29.7	+6.7	-24.4	-47.4	17.5	19.3	+1.8	-28.8	-46.3

Continued on next page

			Activation barrier $\Delta E^{\ddagger}$					Reaction energy ΔE_{rxn}				
PDB	Model	Backend	Lit.	MLIP	Err.	DFT//MLIP	Err.	Lit.	MLIP	Err.	DFT//MLIP	Err.
		UMA-m-1.1	23.0	28.8	+5.8	27.2	+4.2	17.5	19.2	+1.7	12.2	-5.3
		MACE-OMOL-0	23.0	30.3	+7.3	29.8	+6.8	17.5	20.9	+3.4	22.4	+4.9
		Orb-v3-omol	23.0	28.2	+5.2	28.7	+5.7	17.5	21.7	+4.2	22.7	+5.2
1PWZ	B	UMA-s-1.1	17.9	23.5	+5.6	21.7	+3.8	14.1	9.6	-4.5	10.0	-4.1
		UMA-s-1.2	17.9	23.9	+6.0	21.6	+3.7	14.1	8.5	-5.6	7.8	-6.3
		UMA-m-1.1	17.9	23.6	+5.7	4.8	-13.1	14.1	8.7	-5.4	-0.8	-14.9
		MACE-OMOL-0	17.9	24.5	+6.6	20.7	+2.8	14.1	12.1	-2.0	7.9	-6.2
		Orb-v3-omol	17.9	25.7	+7.8	23.1	+5.2	14.1	10.4	-3.7	9.8	-4.3
1PWZ	C	UMA-s-1.1	18.2	22.2	+4.0	20.6	+2.4	5.5	5.3	-0.2	2.5	-3.0
		UMA-s-1.2	18.2	23.0	+4.8	11.7	-6.5	5.5	5.7	+0.2	0.9	-4.6
		UMA-m-1.1	18.2	22.0	+3.8	19.8	+1.6	5.5	5.2	-0.3	-3.5	-9.0
		MACE-OMOL-0	18.2	22.8	+4.6	20.9	+2.7	5.5	6.9	+1.4	3.1	-2.4
		Orb-v3-omol	18.2	24.3	+6.1	19.6	+1.4	5.5	5.5	+0.0	-0.1	-5.6
1RTQ	s1	UMA-s-1.2	12.1	8.1	-4.0	-0.2	-12.3	11.3	2.3	-9.0	0.1	-11.2
		UMA-m-1.1	12.1	8.5	-3.6	4.5	-7.6	11.3	2.5	-8.8	-1.5	-12.8
		MACE-OMOL-0	12.1	8.7	-3.4	4.6	-7.5	11.3	2.4	-8.9	-1.3	-12.6
		Orb-v3-omol	12.1	7.8	-4.3	5.6	-6.5	11.3	2.0	-9.3	0.4	-10.9
1RTQ	s2	UMA-s-1.1	0.1	3.5	+3.4	1.6	+1.5	-1.2	1.4	+2.6	0.8	+2.0
		UMA-s-1.2	0.1	3.5	+3.4	2.0	+1.9	-1.2	1.8	+3.0	0.6	+1.8
		UMA-m-1.1	0.1	3.4	+3.3	0.5	+0.4	-1.2	1.5	+2.7	1.4	+2.6
		MACE-OMOL-0	0.1	3.8	+3.7	1.5	+1.4	-1.2	1.9	+3.1	0.7	+1.9
		Orb-v3-omol	0.1	2.3	+2.2	2.5	+2.4	-1.2	0.2	+1.4	1.2	+2.4
1RTQ	s3	UMA-s-1.1	-0.8	1.6	+2.4	2.0	+2.8	-0.1	-0.4	-0.3	0.8	+0.9
		UMA-s-1.2	-0.8	1.6	+2.4	5.6	+6.4	-0.1	-0.6	-0.5	21.6	+21.7
		UMA-m-1.1	-0.8	1.4	+2.2	1.8	+2.6	-0.1	-0.6	-0.5	1.0	+1.1
		MACE-OMOL-0	-0.8	1.4	+2.2	1.9	+2.7	-0.1	-0.2	-0.1	1.1	+1.2
1RTQ	s4	UMA-s-1.1	-0.6	3.3	+3.9	2.7	+3.3	-11.7	-2.9	+8.8	1.0	+12.7
		UMA-s-1.2	-0.6	2.4	+3.0	3.3	+3.9	-11.7	-4.3	+7.4	0.9	+12.6
		UMA-m-1.1	-0.6	2.9	+3.5	-113.0	-112.4	-11.7	-3.1	+8.6	-13.0	-1.3
		MACE-OMOL-0	-0.6	2.5	+3.1	3.1	+3.7	-11.7	-4.3	+7.4	0.8	+12.5
		Orb-v3-omol	-0.6	3.5	+4.1	0.5	+1.1	-11.7	-2.6	+9.1	-0.1	+11.6
2E7Z	A/s1	UMA-s-1.1	19.8	10.7	-9.1	10.7	-9.1	17.8	1.6	-16.2	-16.4	-34.2
		UMA-s-1.2	19.8	9.4	-10.4	-1.9	-21.7	17.8	0.6	-17.2	-26.4	-44.2
		UMA-m-1.1	19.8	13.6	-6.2	23.5	+3.7	17.8	6.4	-11.4	-2.5	-20.3
		MACE-OMOL-0	19.8	14.8	-5.0	6.1	-13.7	17.8	3.7	-14.1	-3.8	-21.6
		Orb-v3-omol	19.8	16.8	-3.0	4.8	-15.0	17.8	5.4	-12.4	-5.7	-23.5
2E7Z	A/s2	UMA-s-1.1	5.5	9.8	+4.3	20.2	+14.7	-27.0	-26.0	+1.0	-15.0	+12.0
		UMA-s-1.2	5.5	10.3	+4.8	-5.1	-10.6	-27.0	-20.2	+6.8	7.1	+34.1

Continued on next page

PDB	Model	Backend	Activation barrier $\Delta E^{\ddagger}$					Reaction energy ΔE_{rxn}				
			Lit.	MLIP	Err.	DFT//MLIP	Err.	Lit.	MLIP	Err.	DFT//MLIP	Err.
		UMA-m-1.1	5.5	12.5	+7.0	18.4	+12.9	-27.0	-21.1	+5.9	-27.1	-0.1
		MACE-OMOL-0	5.5	9.6	+4.1	15.8	+10.3	-27.0	-17.1	+9.9	-13.1	+13.9
		Orb-v3-omol	5.5	8.3	+2.8	7.8	+2.3	-27.0	-21.6	+5.4	-24.7	+2.3
2E7Z	A/s4	UMA-s-1.1	14.7	20.4	+5.7	58.9	+44.2	3.6	2.6	-1.0	35.1	+31.5
		UMA-s-1.2	14.7	22.6	+7.9	82.7	+68.0	3.6	2.8	-0.8	40.5	+36.9
		UMA-m-1.1	14.7	21.0	+6.3	57.3	+42.6	3.6	0.1	-3.5	33.6	+30.0
		MACE-OMOL-0	14.7	18.3	+3.6	21.0	+6.3	3.6	0.5	-3.1	2.8	-0.8
		Orb-v3-omol	14.7	17.0	+2.3	18.6	+3.9	3.6	-2.0	-5.6	3.4	-0.2
4OTA	A/s1	UMA-s-1.1	12.8	14.0	+1.2	-5.1	-17.9	9.8	-2.2	-12.0	-22.0	-31.8
		UMA-s-1.2	12.8	14.4	+1.6	-1.6	-14.4	9.8	-0.8	-10.6	-1.3	-11.1
		UMA-m-1.1	12.8	13.8	+1.0	6.8	-6.0	9.8	-2.1	-11.9	-18.6	-28.4
		MACE-OMOL-0	12.8	13.5	+0.7	12.6	-0.2	9.8	-2.6	-12.4	-2.2	-12.0
		Orb-v3-omol	12.8	15.7	+2.9	0.3	-12.5	9.8	-1.7	-11.5	-0.5	-10.3
4OTA	B/s1	UMA-s-1.1	6.9	17.5	+10.6	-0.6	-7.5	-5.7	7.8	+13.5	-18.6	-12.9
		UMA-s-1.2	6.9	16.6	+9.7	-4.5	-11.4	-5.7	-0.8	+4.9	0.1	+5.8
		UMA-m-1.1	6.9	16.9	+10.0	32.0	+25.1	-5.7	7.2	+12.9	63.7	+69.4
		MACE-OMOL-0	6.9	23.4	+16.5	27.1	+20.2	-5.7	6.0	+11.7	10.6	+16.3
		Orb-v3-omol	6.9	15.6	+8.7	8.1	+1.2	-5.7	-0.3	+5.4	-5.8	-0.1
4OTA	B/s2	UMA-s-1.1	4.1	11.0	+6.9	9.4	+5.3	2.8	0.8	-2.0	1.1	-1.7
		UMA-s-1.2	4.1	11.4	+7.3	9.6	+5.5	2.8	0.7	-2.1	1.4	-1.4
		UMA-m-1.1	4.1	11.3	+7.2	9.9	+5.8	2.8	0.7	-2.1	1.1	-1.7
		MACE-OMOL-0	4.1	11.3	+7.2	9.1	+5.0	2.8	-21.1	-23.9	-29.3	-32.1
		Orb-v3-omol	4.1	10.5	+6.4	8.9	+4.8	2.8	4.9	+2.1	5.6	+2.8

S6.13 DFT//MLIP Single-Point Calculation

For each of the 90 bond-topology-reproducing default-condition cases (87 clean passes and 3 higher-order saddles), ωB97M-V/def2-SVP single-point energies were computed for the MLIP-optimized R/TS/P geometries (Table S41). In twelve cases the single point places the MLIP-optimized transition-state geometry below its own reactant, so the apparent barrier is negative; the SCF converged for R, TS, and P in every one of them, so this is a property of re-scoring a geometry optimized on a different potential-energy surface rather than a failed calculation. All twelve fall on UMA backends and they account for the large all-case DFT//MLIP deviations there, so Table S40 reports the deviation both over all cases and after excluding barriers < 0 or > 150 kcal mol^{-1}.

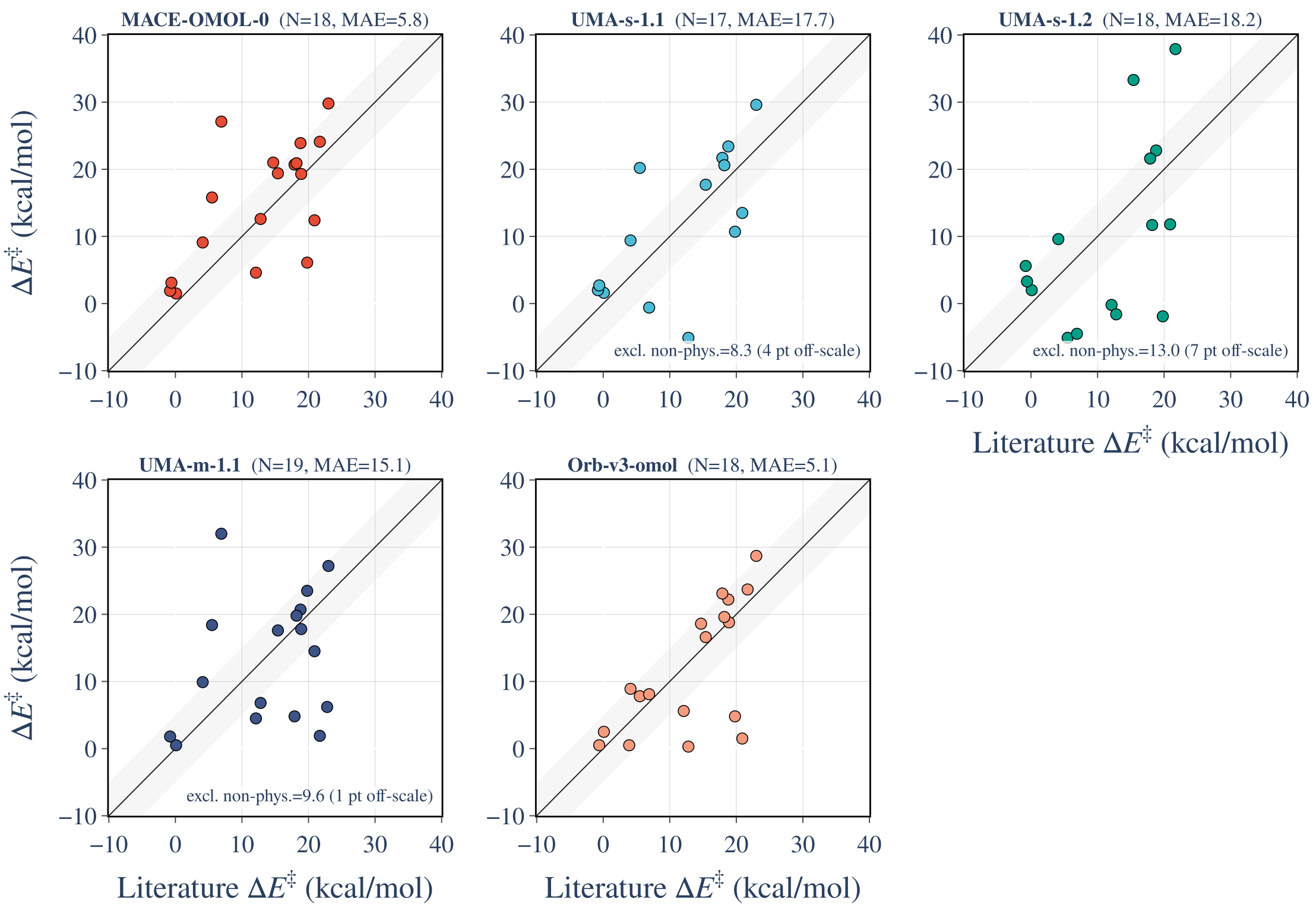

Figure S13: DFT//MLIP activation energies (ωB97M-V/def2-SVP) compared against reported literature B3LYP/6-311+G(2d,2p) values for the 90 bond-topology-reproducing default-condition cases. Filled markers show DFT//MLIP values, and open markers show the corresponding MLIP-only values. Each panel title reports N and the DFT//MLIP mean absolute deviation on the bond-topology-reproducing subset. Where applicable, the inset reports the deviation after excluding non-physical DFT//MLIP barriers and the number of off-scale points. The MLIP-only correlation is evaluated separately in Figure S9.

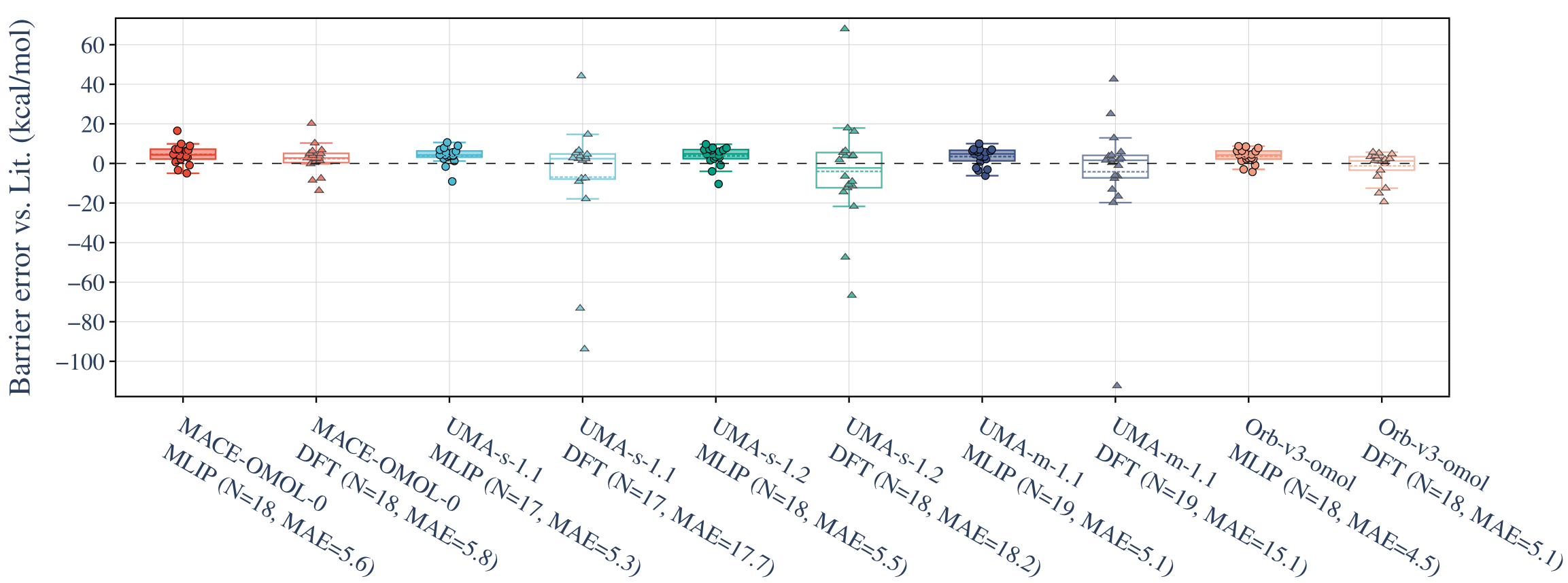

Figure S14: Barrier-deviation distribution (value − literature, kcal mol^{-1}) per backend for MLIP (circles) and DFT//MLIP (ωB97M-V/def2-SVP, triangles) on the same MLIP-optimized geometries. The plot-wide dashed line is zero; solid and dashed box lines are the median and mean. DFT//MLIP boxes and printed MAEs retain the 12 negative apparent barriers on UMA backends (Table S40).

S6.14 Computational Performance

All timings in this subsection were measured on a single NVIDIA GH200 120 GB GPU. Figure S15 shows the distribution of total pipeline wall-clock time per bond-topology-reproducing case; Table S42 and Figure S16 give the per-case and per-stage breakdowns. Across all backends the median stage costs are Path Opt 2.8, TS Opt 2.5, Freq 1.9, Endpoint Opt 0.7, and IRC 0.4 min, against a median of 4.8 min for the three DFT single points summed over R/TS/P. Pooled over the 90 cases, a complete elementary step—path optimization, TS optimization, IRC validation, the finite-difference Hessian, and QRRHO thermochemistry—takes a median of 9.2 min (interquartile range 6.2–16.9 min), i.e. about twice the wall-clock of the three ωB97M-V/def2-SVP single-point energies that re-score its reactant, transition state, and product on the same cluster. The largest MLIP-only totals arise from MEP and TS optimization, while Endpoint Opt is inexpensive in the median but has a long tail (up to 10.4 min on the largest cluster). The DFT column reports the summed R/TS/P single-point wall-clock time for the 90 bond-topology-reproducing cases and is excluded from Total. "Endpoint Opt" is the post-IRC re-optimization of the reaction endpoints, summed over both endpoints of the segment. The pipeline emits no elapsed-time line for this stage, so its wall-clock is recovered from the optimizer's own cycle table in the run log (final cycle count times the mean reported seconds-per-cycle); the per-run values are shipped as `benchmark/endpoint_opt_timings.csv` in the SI data bundle. The Total column additionally includes pre-flight and bookkeeping steps not enumerated in the breakdown columns (PDB element completion, pocket extraction, cap-hydrogen insertion, and optional pre-optimization), so the per-stage columns sum to less than Total. For most cases the shortfall is small (the breakdown columns account for a median of 95% of Total), but it is substantial for a minority of large-cluster runs—most conspicuously 4OTA B/step 2, where pre-flight dominates and the enumerated stages account for as little as 34% of Total.

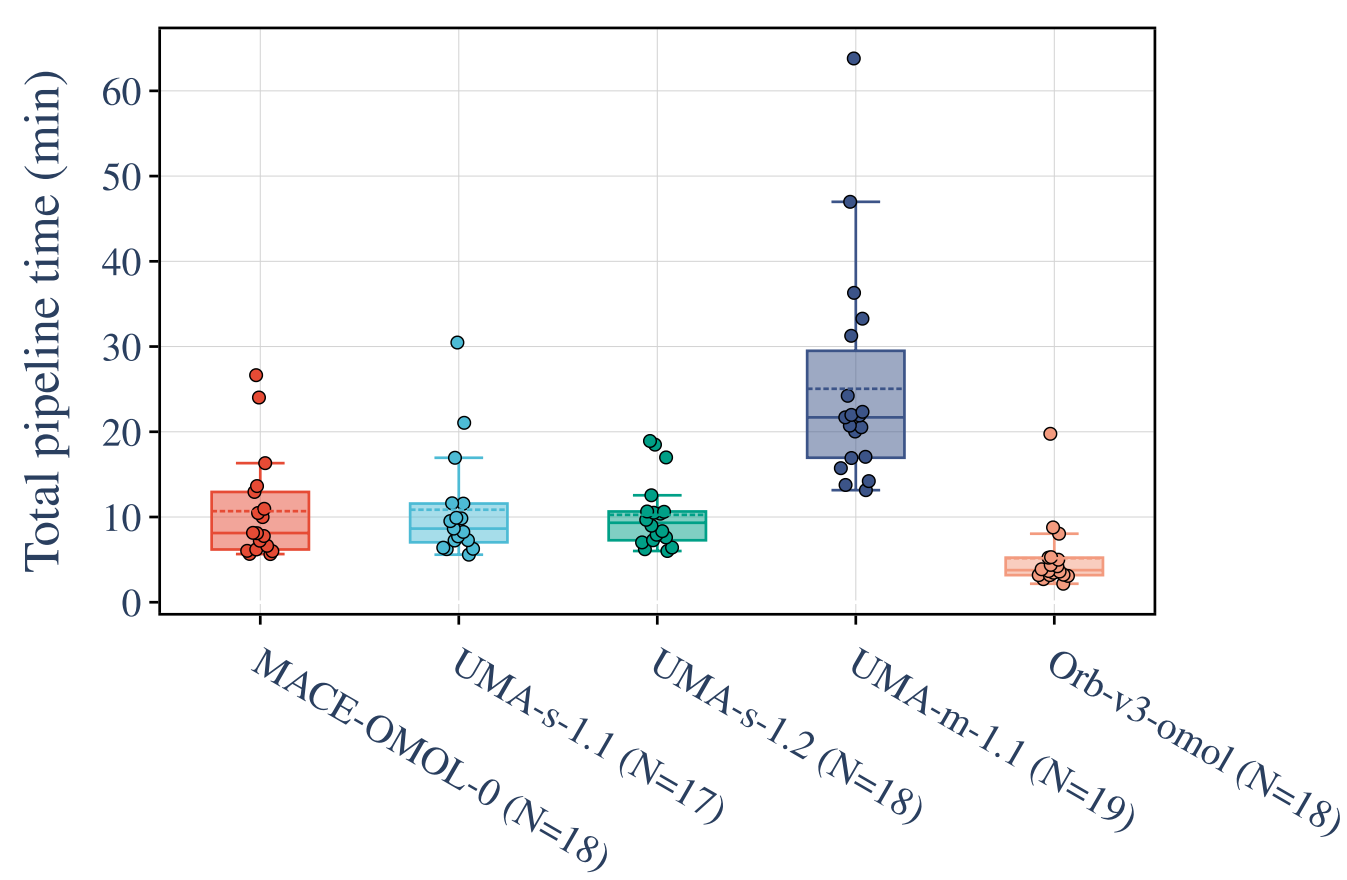

Figure S15: Distribution of total pipeline wall-clock time per bond-topology-reproducing case. Each point represents one run; boxes show median and interquartile range; the dashed bar is the within-box mean. Orb-v3-omol is the fastest of the tested backends, MACE-OMOL-0/UMA-s-1.1/UMA-s-1.2 cluster around 8–10 min, and UMA-m-1.1 is the slowest.

Table S42: Wall-clock time (minutes) per benchmark case and stage for the 90 bond-topology-reproducing default-condition cases (87 clean passes and 3 higher-order saddles). Density-functional theory (DFT) is the summed ωB97M-V/def2-SVP single-point time for R/TS/P and is excluded from Total. Endpoint Opt is post-IRC re-optimization summed over both endpoints. Total also includes pre-flight and bookkeeping steps not listed separately, so stage columns need not sum to Total.

PDB	Model	N_{atoms}	Backend	Total (min)	Path (min)	TS Opt (min)	IRC (min)	Endpoint Opt (min)	Freq (min)	DFT (min)
1AH7	A	109	UMA-m-1.1	22.0	7.5	5.6	0.7	1.7	5.3	7.6
	B	111	UMA-s-1.1	9.9	3.3	2.5	0.5	1.0	2.1	8.0
	B	111	UMA-s-1.2	10.4	3.5	2.6	0.3	1.3	2.3	7.1
	B	111	UMA-m-1.1	21.9	7.2	5.7	0.3	2.2	5.3	8.4
	B	111	MACE-OMOL-0	10.0	3.6	2.5	0.3	0.9	2.2	7.2
	B	111	Orb-v3-omol	4.2	1.5	1.0	0.2	0.5	0.8	7.1
	Ba	105	Orb-v3-omol	3.6	1.0	0.8	0.4	0.2	0.8	6.7
1O9S	A	29	UMA-s-1.1	6.3	3.1	0.8	1.0	0.5	0.7	0.7
	A	29	UMA-s-1.2	7.0	3.3	1.1	1.1	0.4	0.8	1.0
	A	29	UMA-m-1.1	15.7	7.1	2.4	1.3	2.6	1.2	0.8
	A	29	MACE-OMOL-0	5.7	2.7	0.6	1.0	0.7	0.5	0.9
	A	29	Orb-v3-omol	3.1	1.2	0.4	1.0	0.1	0.2	0.9
	B	46	UMA-s-1.1	7.7	3.6	1.4	0.8	0.7	1.0	2.3
	B	46	UMA-s-1.2	8.3	3.8	1.6	0.8	0.6	1.1	1.2
	B	46	UMA-m-1.1	17.1	7.7	3.2	1.1	1.8	2.6	1.3

Continued on next page

PDB	Model	N_{atoms}	Backend	Total (min)	Path (min)	TS Opt (min)	IRC (min)	Endpoint Opt (min)	Freq (min)	DFT (min)
	B	46	MACE-OMOL-0	6.6	3.0	1.1	0.7	0.9	0.8	1.3
	B	46	Orb-v3-omol	3.9	1.4	0.6	0.6	0.6	0.3	1.2
	C	72	UMA-s-1.1	7.3	2.6	1.7	0.6	0.6	1.5	1.8
	C	72	UMA-s-1.2	7.9	2.8	1.8	0.7	0.7	1.6	2.0
	C	72	UMA-m-1.1	33.3	6.4	4.0	0.8	5.1	2.5	2.9
	C	72	MACE-OMOL-0	7.8	2.9	1.7	0.5	1.2	1.3	1.9
	C	72	Orb-v3-omol	3.2	1.1	0.8	0.4	0.2	0.5	1.9
	D	132	UMA-s-1.1	9.5	2.3	3.1	0.5	0.6	2.6	6.1
	D	132	UMA-s-1.2	9.7	2.1	3.2	0.6	0.6	2.8	6.0
	D	132	UMA-m-1.1	20.7	4.3	7.1	0.7	1.3	6.4	7.4
	D	132	MACE-OMOL-0	11.0	3.0	3.4	0.5	0.8	2.9	5.6
	D	132	Orb-v3-omol	4.4	1.1	1.2	0.5	0.3	1.0	5.4
1PWZ	A	83	UMA-s-1.1	8.6	2.4	2.1	0.4	1.0	1.6	2.5
	A	83	UMA-s-1.2	10.6	2.5	3.6	0.4	1.0	1.8	2.7
	A	83	UMA-m-1.1	20.5	5.4	5.6	0.6	2.6	4.1	2.5
	A	83	MACE-OMOL-0	8.1	2.3	1.9	0.4	1.2	1.4	2.5
	A	83	Orb-v3-omol	3.2	0.9	0.9	0.4	0.2	0.6	2.4
	B	112	UMA-s-1.1	8.2	1.9	2.4	0.4	0.7	2.1	4.0
	B	112	UMA-s-1.2	9.0	1.9	2.6	0.4	0.9	2.3	3.8
	B	112	UMA-m-1.1	20.0	4.3	5.7	0.6	2.2	5.5	4.0
	B	112	MACE-OMOL-0	8.1	1.9	2.5	0.4	0.6	2.0	4.0
	B	112	Orb-v3-omol	3.5	0.8	0.9	0.4	0.3	0.8	4.0
	C	161	UMA-s-1.1	9.8	1.9	3.3	0.3	0.5	3.1	7.9
	C	161	UMA-s-1.2	10.5	1.9	3.5	0.5	0.6	3.4	8.1
	C	161	UMA-m-1.1	24.2	4.9	8.1	0.6	1.2	8.0	7.9
	C	161	MACE-OMOL-0	13.6	3.0	4.6	0.4	0.8	4.3	8.1
	C	161	Orb-v3-omol	5.0	1.2	1.5	0.4	0.3	1.3	7.8
1RTQ	s1	82	UMA-s-1.2	7.6	2.7	1.9	0.4	0.5	1.8	4.7
	s1	82	UMA-m-1.1	16.9	5.9	4.4	0.6	1.1	4.2	4.6
	s1	82	MACE-OMOL-0	7.2	2.6	1.8	0.3	0.7	1.5	4.8
	s1	82	Orb-v3-omol	3.2	1.1	0.7	0.5	0.2	0.6	4.8
	s2	82	UMA-s-1.1	5.6	1.3	1.7	0.1	0.5	1.7	4.8
	s2	82	UMA-s-1.2	6.0	1.4	1.8	0.2	0.5	1.8	4.8
	s2	82	UMA-m-1.1	13.2	3.0	4.0	0.2	1.1	4.2	4.9
	s2	82	MACE-OMOL-0	5.7	1.5	1.6	0.1	0.7	1.6	4.8
	s2	82	Orb-v3-omol	2.2	0.5	0.6	0.1	0.2	0.6	4.8
	s3	82	UMA-s-1.1	6.2	1.6	2.0	0.2	0.4	1.7	4.8
	s3	82	UMA-s-1.2	6.2	1.6	1.9	0.2	0.4	1.7	4.8
	s3	82	UMA-m-1.1	13.8	3.4	4.3	0.2	0.9	4.1	4.6
	s3	82	MACE-OMOL-0	6.0	1.6	1.8	0.2	0.6	1.5	4.8
	s4	82	UMA-s-1.1	6.4	1.6	1.8	0.6	0.4	1.6	4.8
	s4	82	UMA-s-1.2	6.4	1.8	1.8	0.4	0.4	1.7	4.6
	s4	82	UMA-m-1.1	14.2	3.7	4.1	0.6	1.0	4.0	5.6
	s4	82	MACE-OMOL-0	6.0	1.6	1.8	0.4	0.6	1.5	4.8
	s4	82	Orb-v3-omol	2.7	0.6	0.7	0.4	0.2	0.6	4.8
2E7Z	A/s1	116	UMA-s-1.1	11.6	5.7	2.5	0.4	0.4	2.3	14.4
	A/s1	116	UMA-s-1.2	12.6	6.3	2.6	0.4	0.4	2.4	22.6
	A/s1	116	UMA-m-1.1	31.3	16.8	6.1	0.7	1.2	5.7	14.2
	A/s1	116	MACE-OMOL-0	12.9	6.6	2.6	0.4	0.9	2.3	11.5
	A/s1	116	Orb-v3-omol	5.3	2.8	0.9	0.2	0.2	0.8	11.4
	A/s2	116	UMA-s-1.1	11.6	3.7	2.6	0.4	1.3	2.2	13.8
	A/s2	116	UMA-s-1.2	10.7	3.9	2.8	0.6	0.5	2.5	13.9
	A/s2	116	UMA-m-1.1	21.7	7.3	6.2	0.9	1.0	5.5	13.4
	A/s2	116	MACE-OMOL-0	10.5	4.2	2.7	0.5	0.7	2.2	12.7
	A/s2	116	Orb-v3-omol	5.2	2.5	1.0	0.2	0.4	0.9	12.7
	A/s4	116	UMA-s-1.1	16.9	10.9	2.6	0.2	0.4	2.2	13.7
	A/s4	116	UMA-s-1.2	18.5	10.8	2.8	0.7	1.0	2.5	18.6
	A/s4	116	UMA-m-1.1	36.3	21.5	5.9	0.3	1.8	5.7	13.7
	A/s4	116	MACE-OMOL-0	16.3	10.2	2.6	0.1	0.6	2.2	12.3
	A/s4	116	Orb-v3-omol	8.8	6.2	1.0	0.1	0.4	0.9	12.3
4OTA	A/s1	77	UMA-s-1.1	7.2	2.0	1.9	0.2	1.2	1.6	2.3
	A/s1	77	UMA-s-1.2	7.3	2.2	1.8	0.2	1.1	1.6	2.2
	A/s1	77	UMA-m-1.1	22.3	10.9	4.2	0.4	2.0	3.9	2.3
	A/s1	77	MACE-OMOL-0	6.2	1.6	1.6	0.2	1.1	1.4	2.1
	A/s1	77	Orb-v3-omol	3.7	1.6	0.6	0.2	0.5	0.6	2.1
	B/s1	177	UMA-s-1.1	21.1	3.3	12.1	0.9	1.0	3.5	8.7
	B/s1	177	UMA-s-1.2	18.9	3.5	6.9	0.2	2.3	3.8	9.3
	B/s1	177	UMA-m-1.1	47.0	7.4	27.5	0.6	1.8	8.9	9.7
	B/s1	177	MACE-OMOL-0	24.0	4.5	8.6	0.2	3.1	4.9	8.3
	B/s1	177	Orb-v3-omol	8.0	1.5	2.8	0.1	1.1	1.5	8.0
	B/s2	177	UMA-s-1.1	45.8	1.5	3.9	0.2	7.8	3.6	8.9
	B/s2	177	UMA-s-1.2	22.4	1.7	4.2	0.2	4.6	3.9	8.1
	B/s2	177	UMA-m-1.1	84.2	3.6	9.1	0.4	10.4	8.8	8.0

Continued on next page

PDB	Model	N_{atoms}	Backend	Total (min)	Path (min)	TS Opt (min)	IRC (min)	Endpoint Opt (min)	Freq (min)	DFT (min)
	B/s2	177	MACE-OMOL-0	26.6	2.5	5.2	0.2	6.7	4.9	20.7
	B/s2	177	Orb-v3-omol	61.8	0.8	3.0	0.3	1.8	1.4	8.5

S6.15 TS-Optimizer Robustness

To test sensitivity to the TS optimizer, we repeated all 115 backend–step combinations from identical default-condition HEI seeds using RS-P-RFO (the default), RS-I-RFO, TRIM, and the independent Sella implementation.[59] The built-in optimizers were evaluated by the pipeline frequency analysis, whereas Sella used its own partial-Hessian analysis.

Table S43: TS-optimizer robustness: distribution of the transition-state imaginary-mode count over 115 cells (5 backends × 23 steps), all started from the identical default-condition HEI. "clean" = exactly one imaginary mode (a valid TS); "HOSP" = two or more (higher-order saddle); "$n = 0$" = relaxation to a nearby minimum (no imaginary mode); "fail" = the optimizer returned no stationary point at all. The four RS-P-RFO cells that the pipeline reports as errors relaxed to a shallow minimum (lowest mode $+5$ to $+28$ cm^{-1}) and are counted under $n = 0$. Sella completed 111 of the 115 cells; its four failures are all 1O9S model D, where the Sella run aborts inside its own internal-coordinate setup, and its imaginary-mode counts come from its own partial-Hessian analysis, so its column is not strictly commensurate with the three pipeline optimizers. Bold marks the largest clean-TS count. The per-cell outcome behind every entry—imaginary-mode count and dominant imaginary frequency for each optimizer, plus the Sella convergence and IRC flags—is distributed as `ts_optimizer_comparison.csv` in the Supporting Data.

Optimizer	clean ($n = 1$)	HOSP ($n \geq 2$)	$n = 0$	fail
RS-P-RFO (default)	**108**	3	4	0
RS-I-RFO	101	7	7	0
TRIM	102	10	3	0
Sella	105	0	6	4

All four optimizers recovered a clean transition state in a similar number of the 115 cells (101–108). Sella, an independent implementation, returned no higher-order saddle at all; its four missing cells are aborts inside its own internal-coordinate code (all 1O9S model D). The counts differ by only a few cases, so on this set TS recovery is largely optimizer-independent, and we retain RS-P-RFO as the default.

Pooled median wall times differ by less than 20% (RS-P-RFO 150.0 s, RS-I-RFO 155.5 s, Sella 173.6 s, TRIM 179.6 s), while the pooled means spread further (189.6–371.4 s) because TRIM and Sella carry long tails, and the backend-level ordering varies (Table S44). In the typical case the four optimizers therefore cost about the same, and we do not read these differences as a general optimizer advantage.

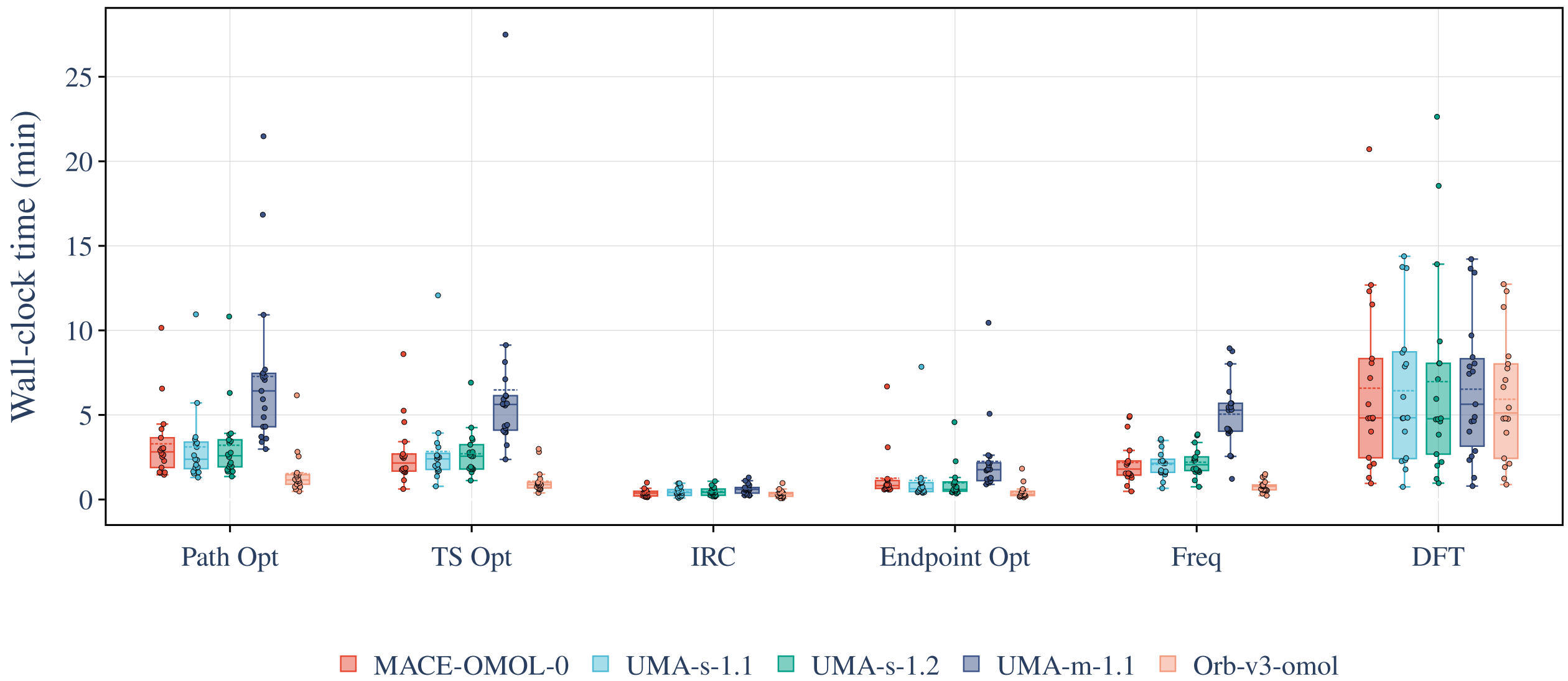

Figure S16: Distribution of wall-clock time per pipeline stage (Path Opt, TS Opt, IRC, Endpoint Opt, Freq, DFT), per MLIP backend. "Path Opt" is the single-pass MEP stage run under the default (non-recursive) condition these timings were measured under, distinct from the recursive path-search (`--refine-path`, opt-in; Section S6.9); "Endpoint Opt" is the post-IRC re-optimization of the two reaction endpoints (Table S42). The 90 bond-topology-reproducing default-condition cases are included; for every stage, $N = 18/17/18/19/18$ for MACE-OMOL-0/UMA-s-1.1/UMA-s-1.2/UMA-m-1.1/Orb-v3-omol, respectively. Each point is one run; the boxes show the median and interquartile range, and the dashed bar is the within-box mean. The DFT box is an ωB97M-V/def2-SVP single point, three per case (R/TS/P). The y-axis is on a linear scale in minutes.

Table S44: TS-optimizer wall-clock time (median seconds per case) by backend, for the same 115-cell comparison as Table S43. `pdb2reaction` optimizer times are the "Elapsed Time for TS Opt" pipeline log entry ($N = 23$ per backend); Sella times are its own `wall_ts_s` record from the 111/115 cells it completed ($N = 22$–23 per backend). Overall column reports the median/mean pooled across all five backends.

Optimizer	MACE-OMOL-0	Orb-v3-omol	UMA-s-1.1	UMA-s-1.2	UMA-m-1.1	Overall (med. / mean)
RS-P-RFO (default)	141.8	53.6	144.5	153.4	337.4	150.0 / 189.6
RS-I-RFO	154.5	60.0	151.8	161.4	335.2	155.5 / 190.7
TRIM	183.1	76.3	174.9	187.1	405.8	179.6 / 300.6
Sella	74.7	235.4	268.2	192.0	155.3	173.6 / 371.4

■ S7. PATH-OPT METHOD AND CLUSTER-SIZE SCALING

`pdb2reaction` provides two minimum-energy-path (MEP) drivers: the growing string method (GSM, default) and Direct-MaxFlux (DMF), with GPU or CPU force evaluation. We compared them for COMT methyl transfer with UMA-s-1.1 across five cluster sizes (457–3420 atoms) and four path resolutions (20/50/100/200 nodes); r2.6–r7.0 denote pocket-radius cutoffs in Å, and "full" denotes the entire enzyme. All runs used a single NVIDIA GH200 120 GB GPU with the UMA-s-1.1 backend; the GSM and DMF-GPU rows evaluate forces on that GPU, and the DMF-CPU rows differ from DMF-GPU only in the device used for the path solver.

S7.1 Wall-clock scaling

Table S45 reports the full 60-run path-opt matrix. For the full 3420-atom system, GSM increased from 83 to 714 min between 20 and 100 nodes, whereas DMF-GPU increased from 95 to 119 min and was 6.0× faster at 100 nodes. Thus the drivers are comparable for small, low-resolution clusters, while DMF is less sensitive to path resolution for large systems. The ordering reverses at benchmark scale: on the 29–177-atom cluster models of the 23-step benchmark, GSM path optimization is the cheaper driver in 113 of 115 cells (median 3.0 against 12.3 min; `timing_mep_comparison.csv` in the Supporting Data). DMF is therefore a large-cluster, high-resolution option rather than a faster default.

Table S45: Path-opt wall-clock time (minutes) across the full cluster-size × node-count matrix, UMA-s-1.1. "–": no completion time was obtained (full system, 200 nodes).

Cluster	Method	20 nodes	50 nodes	100 nodes	200 nodes
r2.6 (457)	GSM (default)	5	12	38	161
	DMF (GPU)	5	10	23	41
	DMF (CPU)	5	13	26	49
r4.0 (670)	GSM (default)	12	16	44	224
	DMF (GPU)	14	20	29	51
	DMF (CPU)	11	20	27	58
r5.5 (868)	GSM (default)	14	18	46	262
	DMF (GPU)	16	24	22	67
	DMF (CPU)	15	48	92	176
r7.0 (1117)	GSM (default)	11	22	104	344
	DMF (GPU)	13	37	65	48
	DMF (CPU)	19	37	56	137
full (3420)	GSM (default)	83	304	714	–
	DMF (GPU)	95	112	119	–
	DMF (CPU)	118	144	289	524

S7.2 Reaction fidelity and node count

The bond changes between the optimized path endpoints were computed for every completed run. From the r4.0 cluster (670 atoms) upward, the intended S_N2 event—C–O bond formation (methyl transfer to the catecholate oxygen) with concomitant S–C cleavage—was recovered by both methods at all completed node counts, and recovered as the sole bond change at 50 nodes and above.

Two measured limits set the recommended range:

- **Low path resolution (20 nodes).** At `--max-nodes 20` the coarse path occasionally couples an adjacent active-site proton transfer into the endpoint bond-change set (an extra N–H/O–H rearrangement on top of the S_N2), so the path no longer represents the target elementary step cleanly. This contamination disappears at 50 nodes and above.
- **Cluster too small.** The smallest cluster (r2.6, 457 atoms) changes the *mechanism* itself: a proton transfer is intrinsically co-located with the methyl transfer regardless of node count, so its barrier is not comparable to the larger models.

We therefore recommend `--max-nodes 50--100` for production path-opt runs. `path-opt` computes no Hessian; imaginary-mode counts are reported with the benchmark (Section S6). The driver choice itself has little effect on reaction fidelity: in the benchmark the Direct-MaxFlux condition stays within two bond-topology-reproducing steps of the default GSM condition for every backend (Table S39).

S7.3 Basin-referenced barrier definition

The MEP endpoints returned by path-opt are *not* in general the reactant and product basins: an optimized string can terminate on the shoulder of a well, so an endpoint-referenced barrier (max $E - E_{\text{endpoint}}$) underestimates the true value. The physically meaningful barrier is obtained by locating the highest-energy image (HEI) along the path and walking downhill in each direction to the first local minimum (the reactant and product basins); the barrier is then $E_{\text{HEI}} - E_{\text{R basin}}$. All barriers reported here use this basin-referenced definition. This is a path-opt–level estimate. The final reported barrier always follows the subsequent TS optimization, which path-opt only needs to seed with a good HEI guess.

S7.4 Cluster-size dependence of the barrier

At 50 nodes, the 670–1117-atom clusters reproduce the 3420-atom full-system path-opt barrier to within 0.2–4.0 kcal mol^{-1} across both drivers (Table S46), and every cluster returns the intended S_N2 bond change. The residual is non-monotonic in cluster size: for both drivers the barrier rises from r4.0 to r7.0 (DMF $11.2 \rightarrow 14.6$; GSM $15.0 \rightarrow 17.0$ kcal mol^{-1}) and then falls at the full system (12.0 and 13.0). Cluster size alone therefore does not order the deviation, and at the path-optimization level a cluster barrier is resolved only to within a few kcal mol^{-1}. The two drivers also differ systematically, GSM returning barriers 1.0–3.9 kcal mol^{-1} above DMF on the same clusters. Neither value is the production barrier: both are HEI seeds for the TS optimization that sets it. For COMT, a moderate active-site cluster (r4.0–r7.0; 670–1117 atoms) thus recovers both the mechanism and the full-system barrier to within that resolution, and the full system serves as a confirmation rather than a required model size.

Table S46: Cluster-size convergence of the basin-referenced S_N2 barrier (`--max-nodes 50`, UMA-s-1.1). The r2.6 model is included, but its mechanism differs from the larger clusters (Section S7.2) and its barrier is therefore not comparable to the clean-S_N2 rows below it.

Cluster	Atoms	$\Delta E^\ddagger$ DMF (kcal mol^{-1})	$\Delta E^\ddagger$ GSM (kcal mol^{-1})	Reaction
r2.6	457	5.6	8.0	proton-transfer-coupled
r4.0	670	11.2	15.0	clean S_N2
r5.5	868	12.2	16.1	clean S_N2
r7.0	1117	14.6	17.0	clean S_N2
full	3420	12.0	13.0	clean S_N2

Table S47 gives the full basin-referenced barrier matrix (20 node-count–cluster-size combinations × 2 drivers; 38 of the 40 cells yielded a barrier). It is what fixes the `--max-nodes 50--100` recommendation: at 20 nodes the full-system DMF profile rises monotonically with no reactant basin, and at 200 nodes two GSM profiles show ambiguous double maxima while the full system returns no barrier for either driver, whereas the 50- and 100-node columns are unflagged for every cluster whose mechanism is comparable (r4.0 and above). The 50-node column is the only one with no flagged cell at any cluster size, r2.6 included.

Table S47: Basin-referenced S_N2 barrier (kcal mol^{-1}, UMA-s-1.1) across the full node-count × cluster-size × driver matrix. "–": no barrier obtained (full system, 200 nodes: GSM did not finish within the pool job's wall-clock allocation; DMF-GPU terminated before producing a barrier). †: profile shows a monotonic energy rise with no return to a reactant-like basin within the sampled path (severe under-resolution artifact, not a physical barrier). ‡: profile shows two or more sequential local maxima of comparable height (within 1 kcal mol^{-1} of each other); the reported value is the global maximum, but no single point is an unambiguous rate-limiting barrier at this resolution.

Cluster	Driver	20 nodes	50 nodes	100 nodes	200 nodes
r2.6 (457)	DMF	5.0	5.6	5.7	5.9
	GSM	7.6	8.0	$7.1^\ddagger$	$7.7^\ddagger$
r4.0 (670)	DMF	10.9	11.2	11.2	10.9
	GSM	15.8	15.0	16.2	16.2
r5.5 (868)	DMF	11.1	12.2	11.7	12.3
	GSM	16.1	16.1	15.4	$8.3^\ddagger$
r7.0 (1117)	DMF	12.4	14.6	14.8	13.4
	GSM	16.5	17.0	17.5	17.6
full (3420)	DMF	$8.4^\dagger$	12.0	9.3	–
	GSM	19.2	13.0	13.7	–

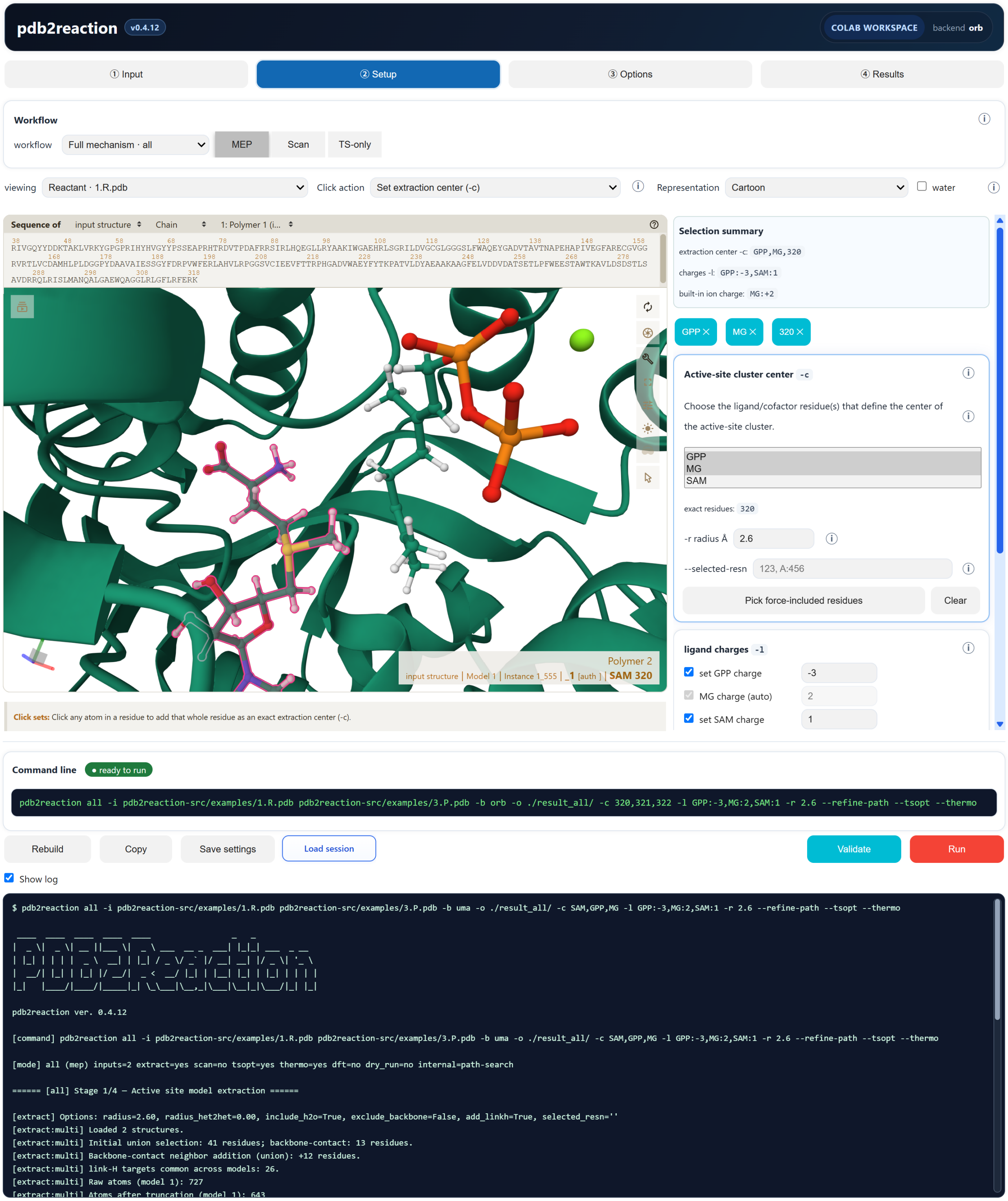

Figure S17: Interactive Colab notebook interface for `pdb2reaction`. The Setup view shows three-dimensional structure and residue selection, the generated command, validation and execution controls, and a live run log. It uses the same numerical settings as direct CLI execution.

REFERENCES

[1] Bondi, A. Van Der Waals Volumes and Radii. *J. Phys. Chem.* **1964**, *68*, 441–451.

[2] Mantina, M.; Chamberlin, A. C.; Valero, R.; Cramer, C. J.; Truhlar, D. G. Consistent van der Waals Radii for the Whole Main Group. *J. Phys. Chem. A* **2009**, *113*, 5806–5812.

[3] Liu, D. C.; Nocedal, J. On the Limited Memory BFGS Method for Large Scale Optimization. *Math. Program.* **1989**, *45*, 503–528.

[4] Banerjee, A.; Adams, N.; Simons, J.; Shepard, R. Search for Stationary Points on Surfaces. *J. Phys. Chem.* **1985**, *89*, 52–57.

[5] Peters, B.; Heyden, A.; Bell, A. T.; Chakraborty, A. A Growing String Method for Determining Transition States: Comparison to the Nudged Elastic Band and String Methods. *J. Chem. Phys.* **2004**, *120*, 7877–7886.

[6] Zimmerman, P. M. Reliable Transition State Searches Integrated with the Growing String Method. *J. Chem. Theory Comput.* **2013**, *9*, 3043–3050.

[7] Henkelman, G.; Uberuaga, B. P.; Jónsson, H. A Climbing Image Nudged Elastic Band Method for Finding Saddle Points and Minimum Energy Paths. *J. Chem. Phys.* **2000**, *113*, 9901–9904.

[8] Koda, S.; Saito, S. Locating Transition States by Variational Reaction Path Optimization with an Energy-Derivative-Free Objective Function. *J. Chem. Theory Comput.* **2024**, *20*, 2798–2811.

[9] Koda, S.; Saito, S. Flat-Bottom Elastic Network Model for Generating Improved Plausible Reaction Paths. *J. Chem. Theory Comput.* **2024**, *20*, 7176–7187.

[10] Koda, S.; Saito, S. Correlated Flat-Bottom Elastic Network Model for Improved Bond Rearrangement in Reaction Paths. *J. Chem. Theory Comput.* **2025**, *21*, 3513–3522.

[11] Wächter, A.; Biegler, L. T. On the Implementation of an Interior-Point Filter Line-Search Algorithm for Large-Scale Nonlinear Programming. *Math. Program.* **2006**, *106*, 25–57.

[12] Helgaker, T. Transition-State Optimizations by Trust-Region Image Minimization. *Chem. Phys. Lett.* **1991**, *182*, 503–510.

[13] Henkelman, G.; Jónsson, H. A Dimer Method for Finding Saddle Points on High Dimensional Potential Surfaces Using Only First Derivatives. *J. Chem. Phys.* **1999**, *111*, 7010–7022.

[14] Olsen, R. A.; Kroes, G.-J.; Henkelman, G.; Arnaldsson, A.; Jónsson, H. Comparison of Methods for Finding Saddle Points without Knowledge of the Final States. *J. Chem. Phys.* **2004**, *121*, 9776–9792.

[15] Heyden, A.; Bell, A. T.; Keil, F. J. Efficient Methods for Finding Transition States in Chemical Reactions: Comparison of Improved Dimer Method and Partitioned Rational Function Optimization Method. *J. Chem. Phys.* **2005**, *123*, 224101.

[16] Kästner, J.; Sherwood, P. Superlinearly Converging Dimer Method for Transition State Search. *J. Chem. Phys.* **2008**, *128*, 014106.

[17] Anglada, J. M.; Bofill, J. M. How Good Is a Broyden–Fletcher–Goldfarb–Shanno-Like Update Hessian Formula to Locate Transition Structures? Specific Reformulation of Broyden–Fletcher–Goldfarb–Shanno for Optimizing Saddle Points. *J. Comput. Chem.* **1998**, *19*, 349–362.

[18] Besalú, E.; Bofill, J. M. On the Automatic Restricted-Step Rational-Function-Optimization Method. *Theor. Chem. Acc.* **1998**, *100*, 265–274.

[19] Bofill, J. M. Updated Hessian Matrix and the Restricted Step Method for Locating Transition Structures. *J. Comput. Chem.* **1994**, *15*, 1–11.

[20] Fukui, K. Formulation of the Reaction Coordinate. *J. Phys. Chem.* **1970**, *74*, 4161–4163.

[21] Gonzalez, C.; Schlegel, H. B. Reaction Path Following in Mass-Weighted Internal Coordinates. *J. Phys. Chem.* **1990**, *94*, 5523–5527.

[22] Hratchian, H. P.; Frisch, M. J.; Schlegel, H. B. Steepest Descent Reaction Path Integration Using a First-Order Predictor–Corrector Method. *J. Chem. Phys.* **2010**, *133*, 224101.

[23] Hratchian, H. P.; Schlegel, H. B. Accurate Reaction Paths Using a Hessian-Based Predictor–Corrector Integrator. *J. Chem. Phys.* **2004**, *120*, 9918–9924.

[24] Li, H.; Jensen, J. H. Partial Hessian Vibrational Analysis: The Localization of the Molecular Vibrational Energy and Entropy. *Theor. Chem. Acc.* **2002**, *107*, 211–219.

[25] Steinmetzer, J. `thermoanalysis`: A Python package for thermochemistry from vibrational frequencies. GitHub repository. `https://github.com/eljost/thermoanalysis` (accessed 2026-01-30), n.d.

[26] Grimme, S. Supramolecular Binding Thermodynamics by Dispersion-Corrected Density Functional Theory. *Chem. Eur. J.* **2012**, *18*, 9955–9964.

[27] Bannwarth, C.; Ehlert, S.; Grimme, S. GFN2-xTB—An Accurate and Broadly Parametrized Self-Consistent Tight-Binding Quantum Chemical Method with Multipole Electrostatics and Density-Dependent Dispersion Contributions. *J. Chem. Theory Comput.* **2019**, *15*, 1652–1671.

[28] Bannwarth, C.; Caldeweyher, E.; Ehlert, S.; Hansen, A.; Pracht, P.; Seibert, J.; Spicher, S.; Grimme, S. Extended Tight-Binding Quantum Chemistry Methods. *WIREs Comput. Mol. Sci.* **2021**, *11*, e1493.

[29] Ehlert, S.; Stahn, M.; Spicher, S.; Grimme, S. Robust and Efficient Implicit Solvation Model for Fast Semiempirical Methods. *J. Chem. Theory Comput.* **2021**, *17*, 4250–4261.

[30] Li, R.; Sun, Q.; Zhang, X.; Chan, G. K.-L. Introducing GPU Acceleration into the Python-Based Simulations of Chemistry Framework. *J. Phys. Chem. A* **2025**, *129*, 1459–1468.

[31] Li, C.; Chan, G. K.-L. Accurate QM/MM Molecular Dynamics for Periodic Systems in GPU4PySCF with Applications to Enzyme Catalysis. *J. Chem. Theory Comput.* **2025**, *21*, 803–816.

[32] Sun, Q.; Berkelbach, T. C.; Blunt, N. S.; Booth, G. H.; Guo, S.; Li, Z.; Liu, J.; McClain, J. D.; Sayfutyarova, E. R.; Sharma, S.; Wouters, S.; Chan, G. K.-L. PySCF: The Python-Based Simulations of Chemistry Framework. *WIREs Comput. Mol. Sci.* **2018**, *8*, e1340.

[33] Sun, Q. et al. Recent Developments in the PySCF Program Package. *J. Chem. Phys.* **2020**, *153*, 024109.

[34] Mardirossian, N.; Head-Gordon, M. ωB97M-V: A Combinatorially Optimized, Range-Separated Hybrid, Meta-GGA Density Functional with VV10 Nonlocal Correlation. *J. Chem. Phys.* **2016**, *144*, 214110.

[35] Rappoport, D.; Furche, F. Property-Optimized Gaussian Basis Sets for Molecular Response Calculations. *J. Chem. Phys.* **2010**, *133*, 134105.

[36] Knizia, G. Intrinsic Atomic Orbitals: An Unbiased Bridge between Quantum Theory and Chemical Concepts. *J. Chem. Theory Comput.* **2013**, *9*, 4834–4843.

[37] Cordero, B.; Gómez, V.; Platero-Prats, A. E.; Revés, M.; Echeverría, J.; Cremades, E.; Barragán, F.; Alvarez, S. Covalent Radii Revisited. *Dalton Trans.* **2008**, 2832–2838.

[38] Warshel, A.; Levitt, M. Theoretical Studies of Enzymic Reactions: Dielectric, Electrostatic and Steric Stabilization of the Carbonium Ion in the Reaction of Lysozyme. *J. Mol. Biol.* **1976**, *103*, 227–249.

[39] Chung, L. W.; Sameera, W. M. C.; Ramozzi, R.; Page, A. J.; Hatanaka, M.; Petrova, G. P.; Harris, T. V.; Li, X.; Ke, Z.; Liu, F.; Li, H.-B.; Ding, L.; Morokuma, K. The ONIOM Method and Its Applications. *Chem. Rev.* **2015**, *115*, 5678–5796.

[40] Kim, D.; Wang, X.; Vargas, S.; Zhong, P.; King, D. S.; Jaffrelot Inizan, T.; Cheng, B. A Universal Augmentation Framework for Long-Range Electrostatics in Machine Learning Interatomic Potentials. *J. Chem. Theory Comput.* **2025**, *21*, 12709–12724.

[41] Olsson, M. H. M.; Søndergaard, C. R.; Rostkowski, M.; Jensen, J. H. PROPKA3: Consistent Treatment of Internal and Surface Residues in Empirical pK_a Predictions. *J. Chem. Theory Comput.* **2011**, *7*, 525–537.

[42] Anandakrishnan, R.; Aguilar, B.; Onufriev, A. V. H++ 3.0: Automating pK Prediction and the Preparation of Biomolecular Structures for Atomistic Molecular Modeling and Simulations. *Nucleic Acids Res.* **2012**, *40*, W537–W541.

[43] Tsutsumi, H.; Moriwaki, Y.; Terada, T.; Shimizu, K.; Shin-ya, K.; Katsuyama, Y.; Ohnishi, Y. Structural and Molecular Basis of the Catalytic Mechanism of Geranyl Pyrophosphate C6-Methyltransferase: Creation of an Unprecedented Farnesyl Pyrophosphate C6-Methyltransferase. *Angew. Chem. Int. Ed.* **2022**, *61*, e202111217.

[44] Kulik, H. J.; Zhang, J.; Klinman, J. P.; Martínez, T. J. How Large Should the QM Region Be in QM/MM Calculations? The Case of Catechol O-Methyltransferase. *J. Phys. Chem. B* **2016**, *120*, 11381–11394.

[45] Schultz, E.; Nissinen, E. Inhibition of Rat Liver and Duodenum Soluble Catechol-O-Methyltransferase by a Tight-Binding Inhibitor OR-462. *Biochem. Pharmacol.* **1989**, *38*, 3953–3956.

[46] Kromann, J. C.; Christensen, A. S.; Cui, Q.; Jensen, J. H. Towards a Barrier Height Benchmark Set for Biologically Relevant Systems. *PeerJ* **2016**, *4*, e1994.

[47] Wood, B. M. et al. UMA: A Family of Universal Models for Atoms. *arXiv* **2025**, arXiv:2506.23971, `https://doi.org/10.48550/arXiv.2506.23971`.

[48] Batatia, I.; Kovács, D. P.; Simm, G. N. C.; Ortner, C.; Csányi, G. MACE: Higher Order Equivariant Message Passing Neural Networks for Fast and Accurate Force Fields. Advances in Neural Information Processing Systems 35 (NeurIPS 2022). 2022; pp 11423–11436.

[49] Levine, D. S. et al. The Open Molecules 2025 (OMol25) Dataset, Evaluations, and Models. *arXiv* **2025**, arXiv:2505.08762, `https://doi.org/10.48550/arXiv.2505.08762`.

[50] Neumann, M.; Gin, J.; Rhodes, B.; Bennett, S.; Li, Z.; Choubisa, H.; Hussey, A.; Godwin, J. Orb: A Fast, Scalable Neural Network Potential. *arXiv* **2024**, arXiv:2410.22570, `https://doi.org/10.48550/arXiv.2410.22570`.

[51] Rhodes, B.; Vandenhaute, S.; Šimkus, V.; Gin, J.; Godwin, J.; Duignan, T.; Neumann, M. Orb-v3: Atomistic Simulation at Scale. *arXiv* **2025**, arXiv:2504.06231, `https://doi.org/10.48550/arXiv.2504.06231`.

[52] Eastman, P.; Pretti, E.; Markland, T. E. Accuracy and Efficiency Benchmarks of Pretrained Machine Learning Potentials for Molecular Simulations. *arXiv* **2026**, arXiv:2601.16331, `https://doi.org/10.48550/arXiv.2601.16331`.

[53] Liao, R.-Z.; Yu, J.-G.; Himo, F. Reaction Mechanism of the Trinuclear Zinc Enzyme Phospholipase C: A Density Functional Theory Study. *J. Phys. Chem. B* **2010**, *114*, 2533–2540.

[54] Georgieva, P.; Himo, F. Quantum Chemical Modeling of Enzymatic Reactions: The Case of Histone Lysine Methyltransferase. *J. Comput. Chem.* **2010**, *31*, 1707–1714.

[55] Hopmann, K. H.; Himo, F. Quantum Chemical Modeling of the Dehalogenation Reaction of Haloalcohol Dehalogenase. *J. Chem. Theory Comput.* **2008**, *4*, 1129–1137.

[56] Chen, S.-L.; Marino, T.; Fang, W.-H.; Russo, N.; Himo, F. Peptide Hydrolysis by the Binuclear Zinc Enzyme Aminopeptidase from *Aeromonas proteolytica*: A Density Functional Theory Study. *J. Phys. Chem. B* **2008**, *112*, 2494–2500.

[57] Liao, R.-Z.; Yu, J.-G.; Himo, F. Mechanism of Tungsten-Dependent Acetylene Hydratase from Quantum Chemical Calculations. *Proc. Natl. Acad. Sci. U.S.A.* **2010**, *107*, 22523–22527.

[58] Sevastik, R.; Himo, F. Quantum Chemical Modeling of Enzymatic Reactions: The Case of 4-Oxalocrotonate Tautomerase. *Bioorg. Chem.* **2007**, *35*, 444–457.

[59] Hermes, E. D.; Sargsyan, K.; Najm, H. N.; Zádor, J. Sella, an Open-Source Automation-Friendly Molecular Saddle Point Optimizer. *J. Chem. Theory Comput.* **2022**, *18*, 6974–6988.
